# Supplementary figures and images for: Regulated degradation of the inner nuclear membrane protein SUN2 maintains nuclear envelope architecture and function (part 1 of 2)
Source: eLife. 2022 Nov 1;11:e81573. doi: 10.7554/eLife.81573 (PMC9662817; doi:10.7554/eLife.81573)

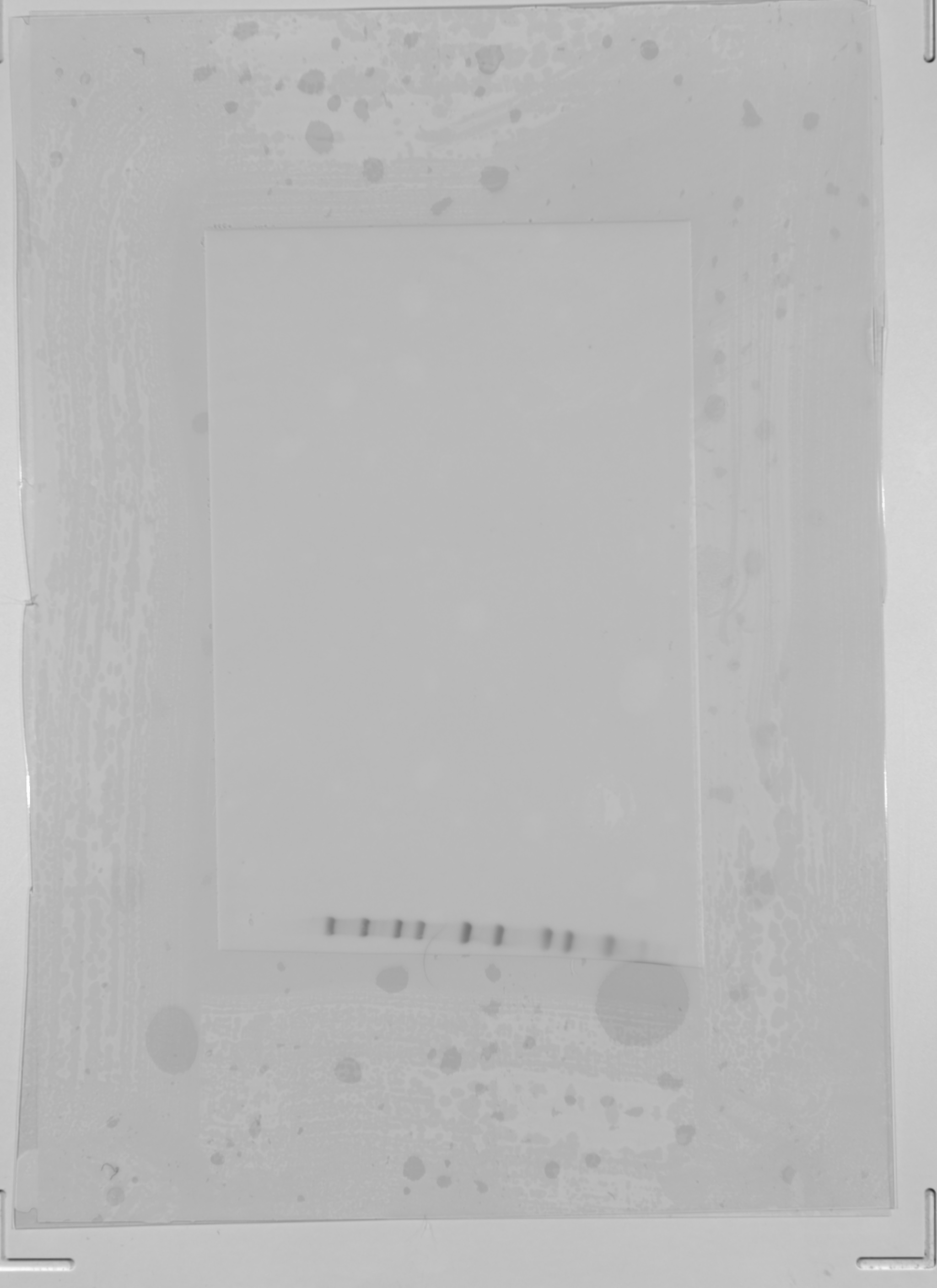

Supplement: Figure 1—source data 1. [file elife-81573-fig1-data1.zip › Figure 1-source data 1/Figure 1-source data 1_raw files/Figure 1E/SUN2 CHX2 SUN2 end 2022.04.13_16.58.09_Ch v SUN2/SUN2 CHX2 SUN2 end 2022.04.13_16.58.09_Ch-Marker.tif]

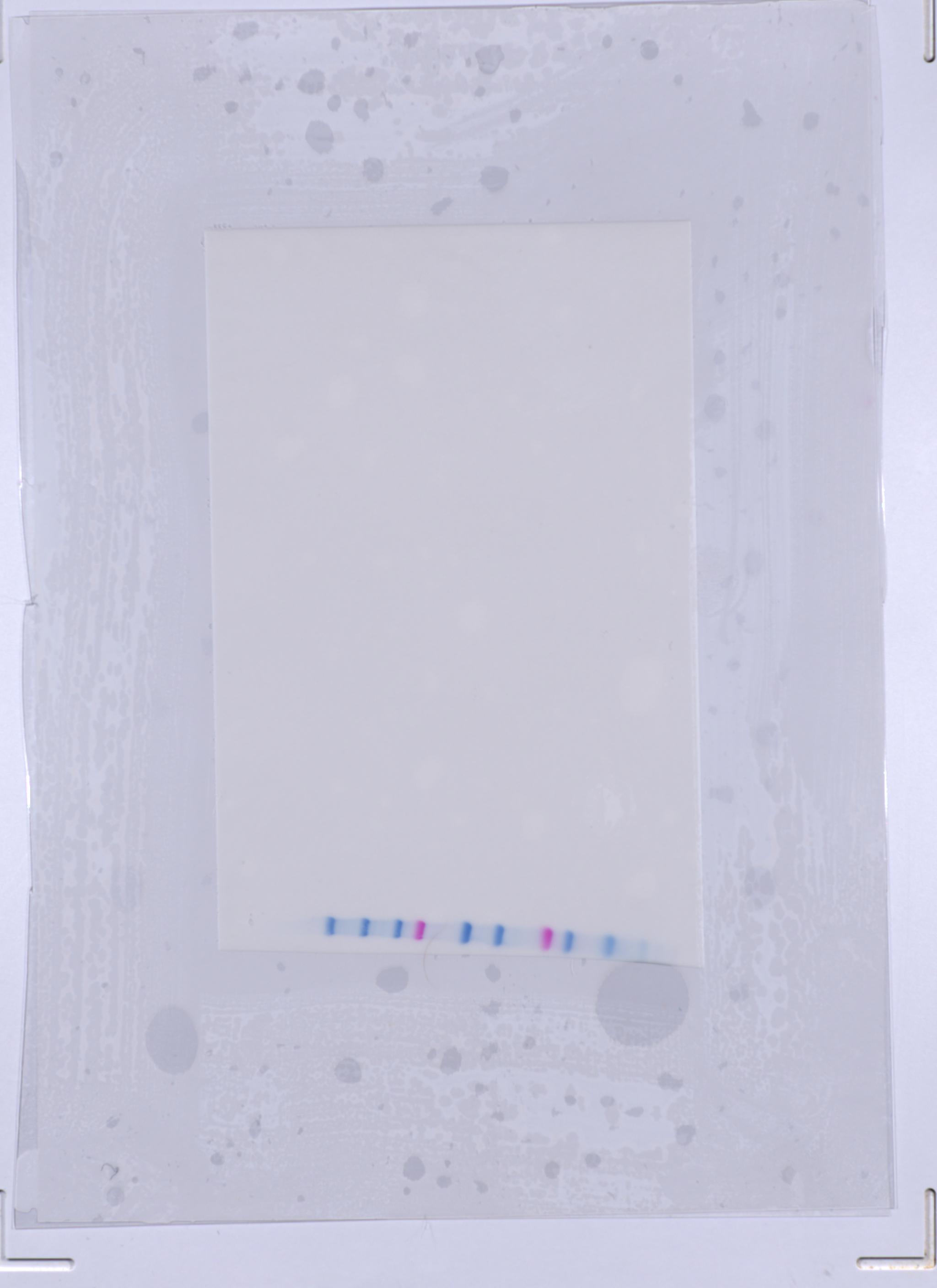

Supplement: Figure 1—source data 1. [file elife-81573-fig1-data1.zip › Figure 1-source data 1/Figure 1-source data 1_raw files/Figure 1E/SUN2 CHX2 SUN2 end 2022.04.13_16.58.09_Ch v SUN2/SUN2 CHX2 SUN2 end 2022.04.13_16.58.09_Ch-Marker.jpg]

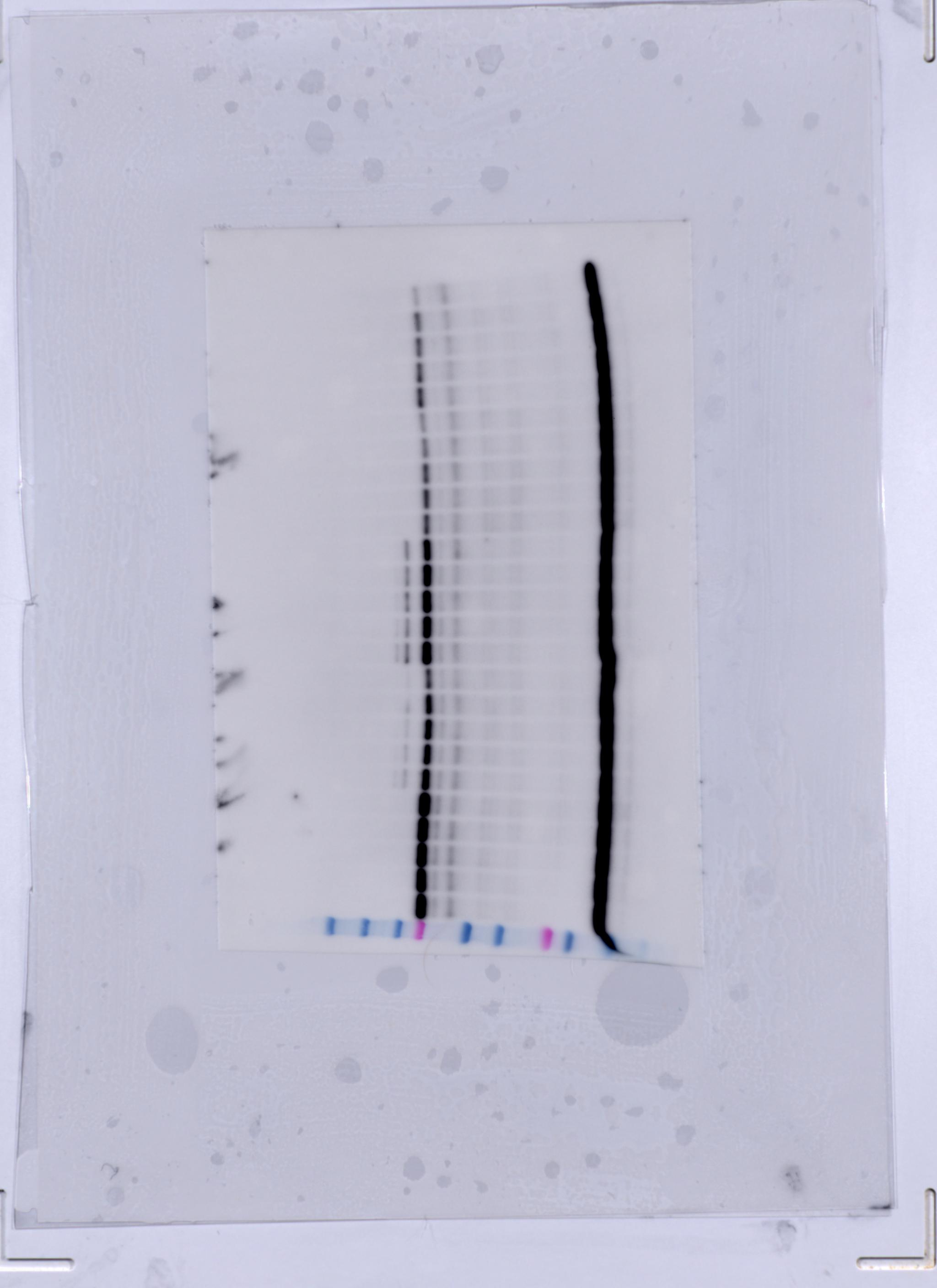

Supplement: Figure 1—source data 1. [file elife-81573-fig1-data1.zip › Figure 1-source data 1/Figure 1-source data 1_raw files/Figure 1E/SUN2 CHX2 SUN2 end 2022.04.13_16.58.09_Ch v SUN2/SUN2 CHX2 SUN2 end 2022.04.13_16.58.09_Ch+Marker.jpg]

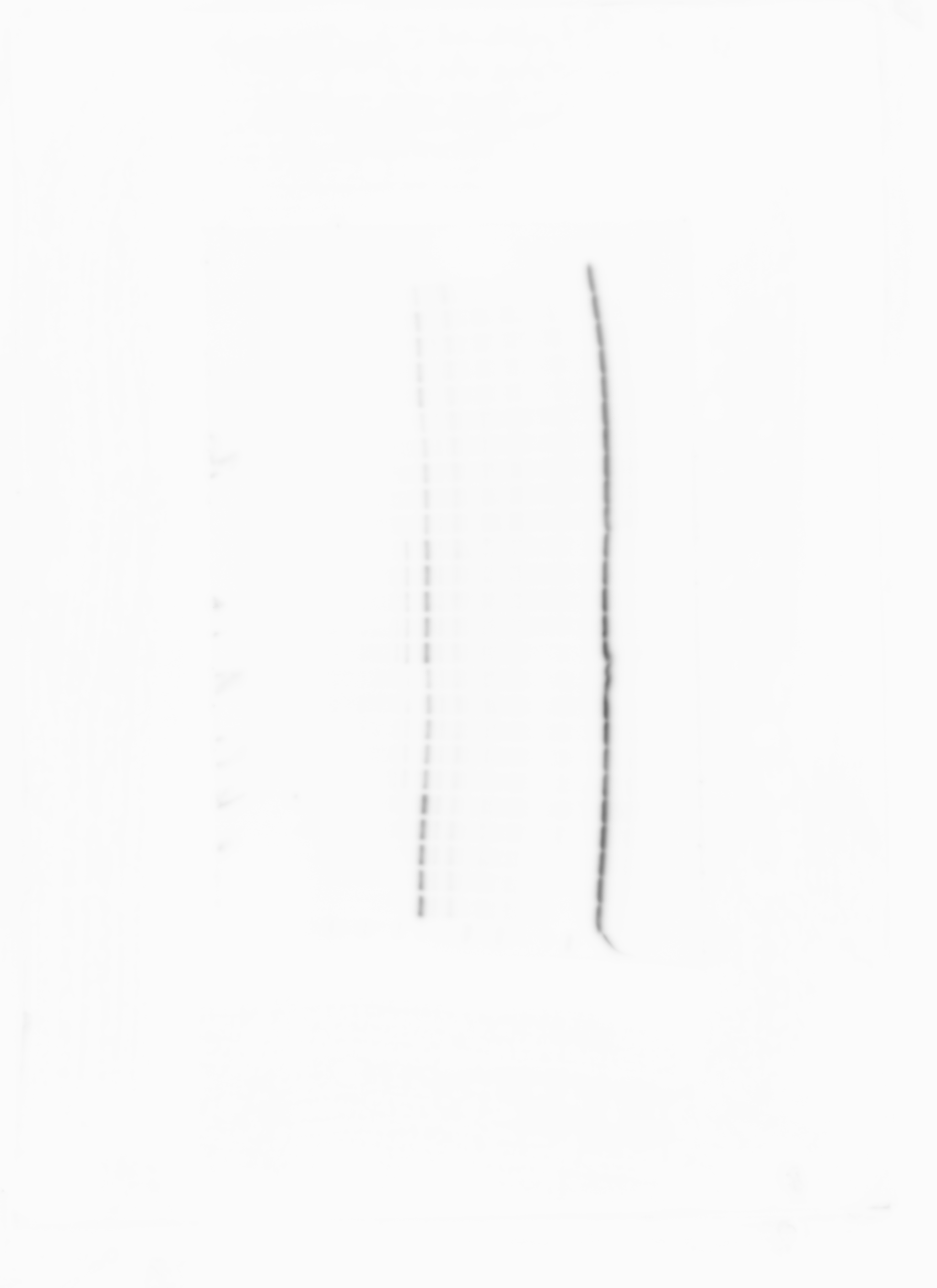

Supplement: Figure 1—source data 1. [file elife-81573-fig1-data1.zip › Figure 1-source data 1/Figure 1-source data 1_raw files/Figure 1E/SUN2 CHX2 SUN2 end 2022.04.13_16.58.09_Ch v SUN2/SUN2 CHX2 SUN2 end 2022.04.13_16.58.09_Ch.tif]

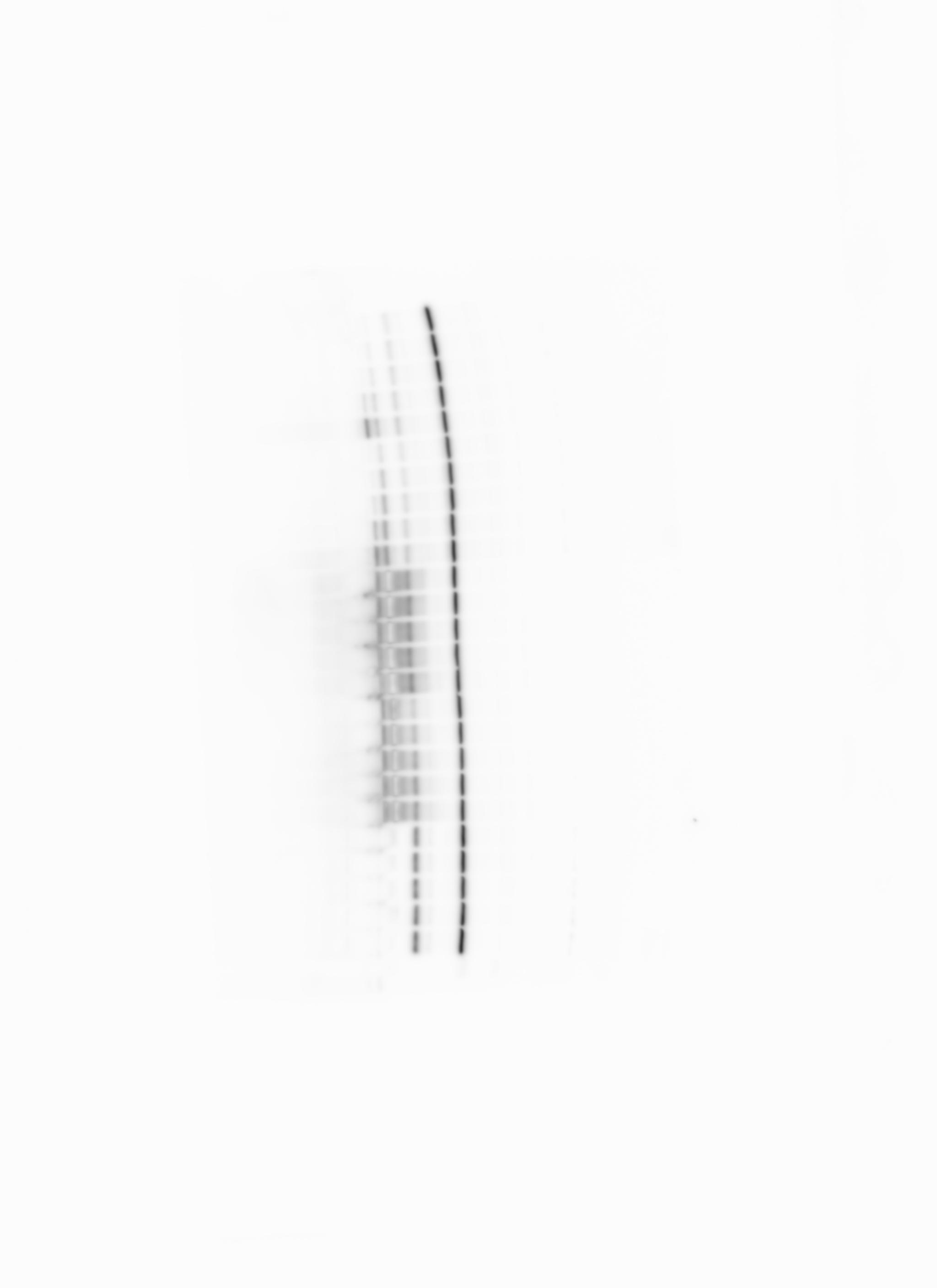

Supplement: Figure 1—source data 1. [file elife-81573-fig1-data1.zip › Figure 1-source data 1/Figure 1-source data 1_raw files/Figure 1E/SUN2 CHX chase S2HPA 2022.03.04_18.18.41-02_Ch v Tub/SUN2 CHX chase S2HPA 2022.03.04_18.18.41-02_Ch.jpg]

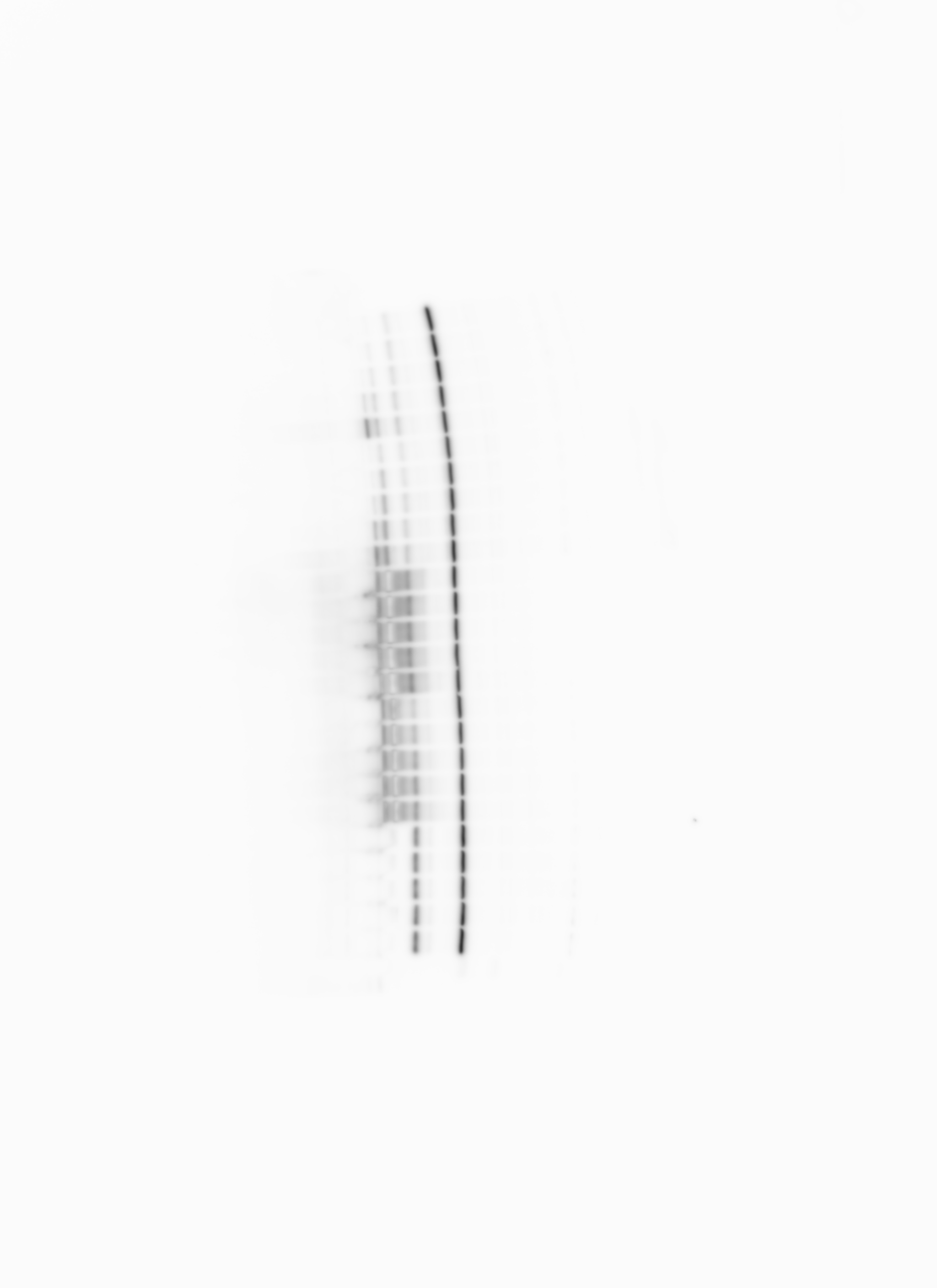

Supplement: Figure 1—source data 1. [file elife-81573-fig1-data1.zip › Figure 1-source data 1/Figure 1-source data 1_raw files/Figure 1E/SUN2 CHX chase S2HPA 2022.03.04_18.18.41-02_Ch v Tub/SUN2 CHX chase S2HPA 2022.03.04_18.18.41-02_Ch.tif]

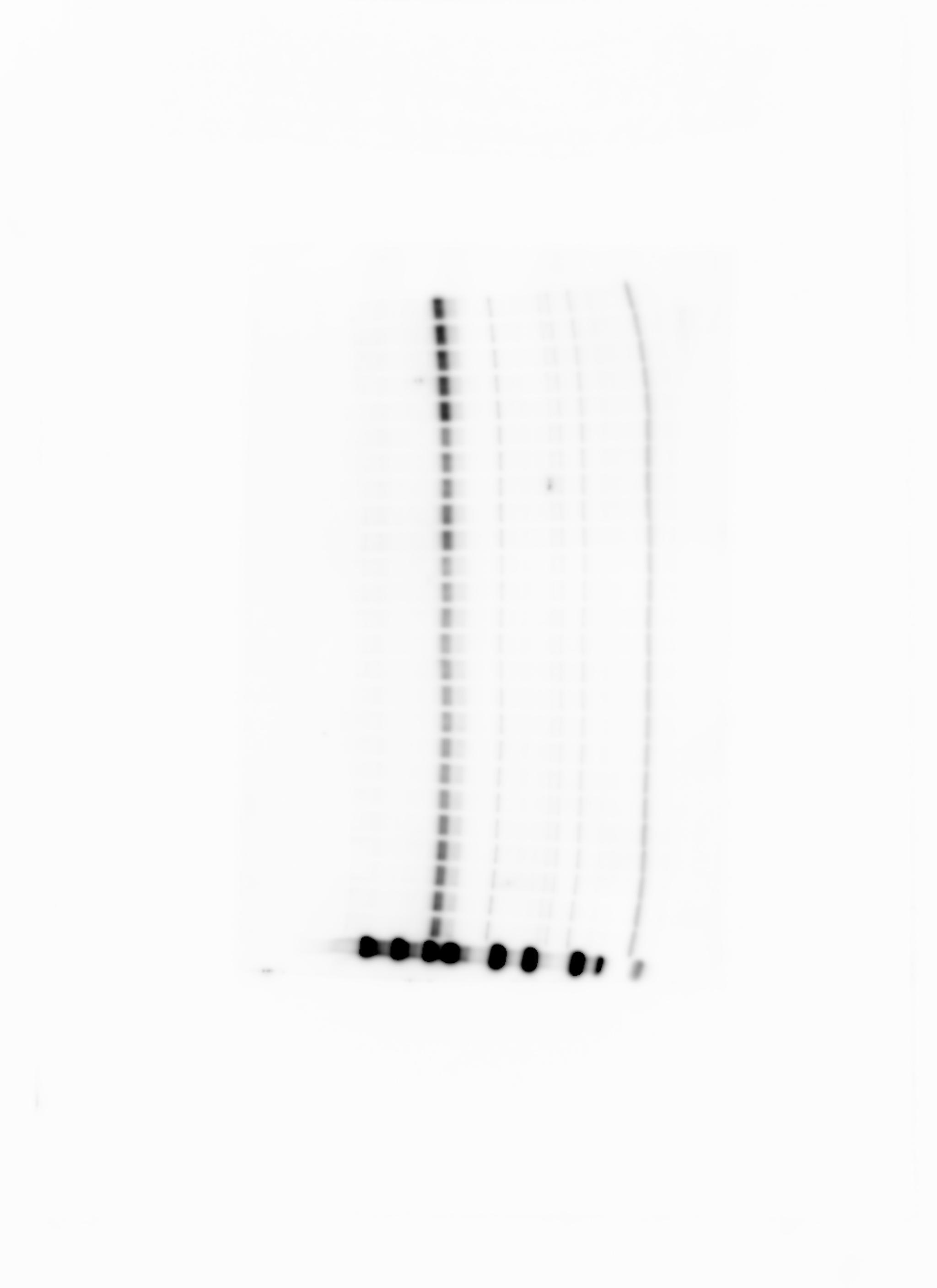

Supplement: Figure 1—source data 1. [file elife-81573-fig1-data1.zip › Figure 1-source data 1/Figure 1-source data 1_raw files/Figure 1E/SUN2 CHX chase SUN1 2022.03.03_19.07.18-06_Ch v SUN1/SUN2 CHX chase SUN1 2022.03.03_19.07.18-06_Ch.jpg]

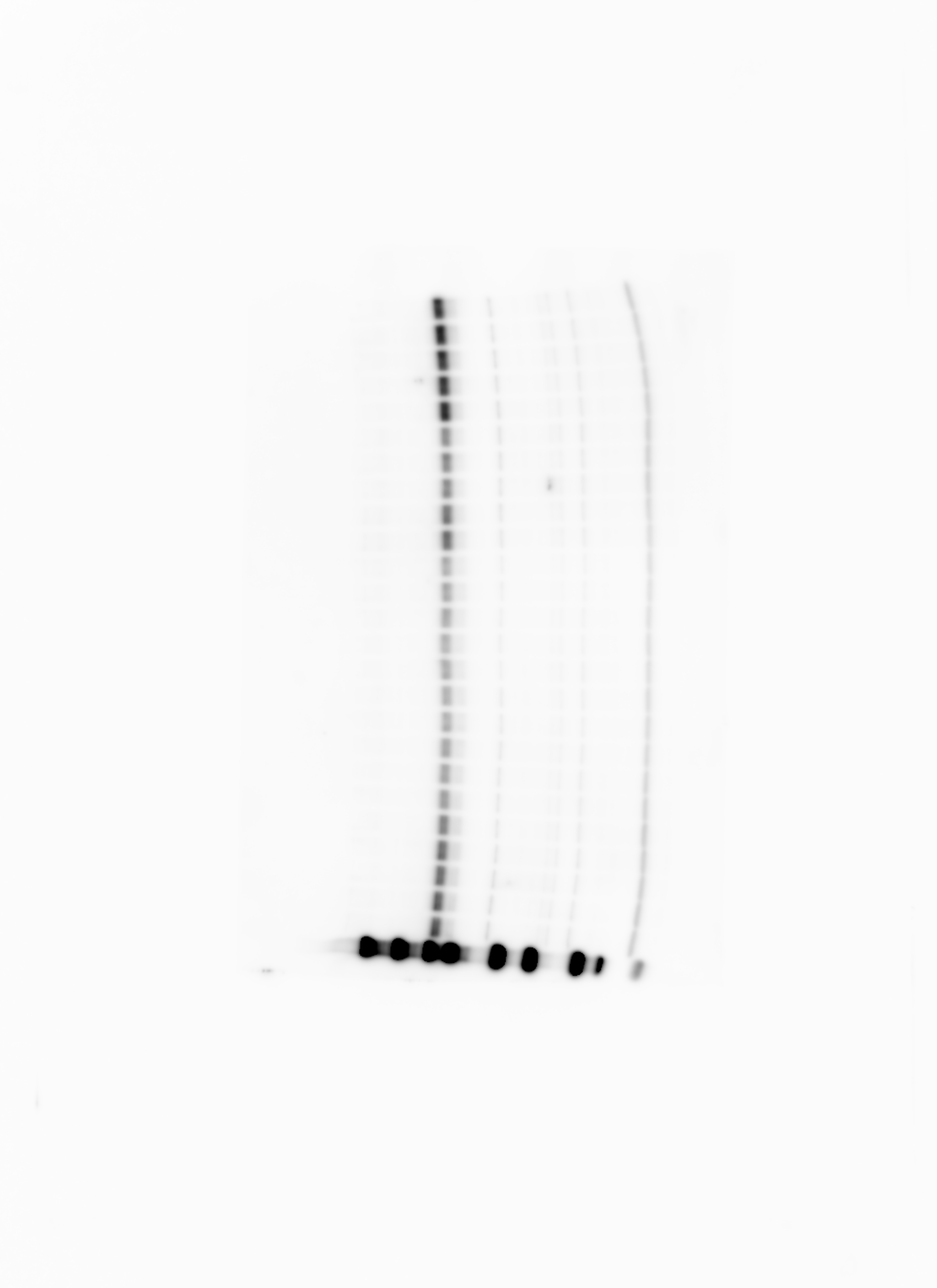

Supplement: Figure 1—source data 1. [file elife-81573-fig1-data1.zip › Figure 1-source data 1/Figure 1-source data 1_raw files/Figure 1E/SUN2 CHX chase SUN1 2022.03.03_19.07.18-06_Ch v SUN1/SUN2 CHX chase SUN1 2022.03.03_19.07.18-06_Ch.tif]

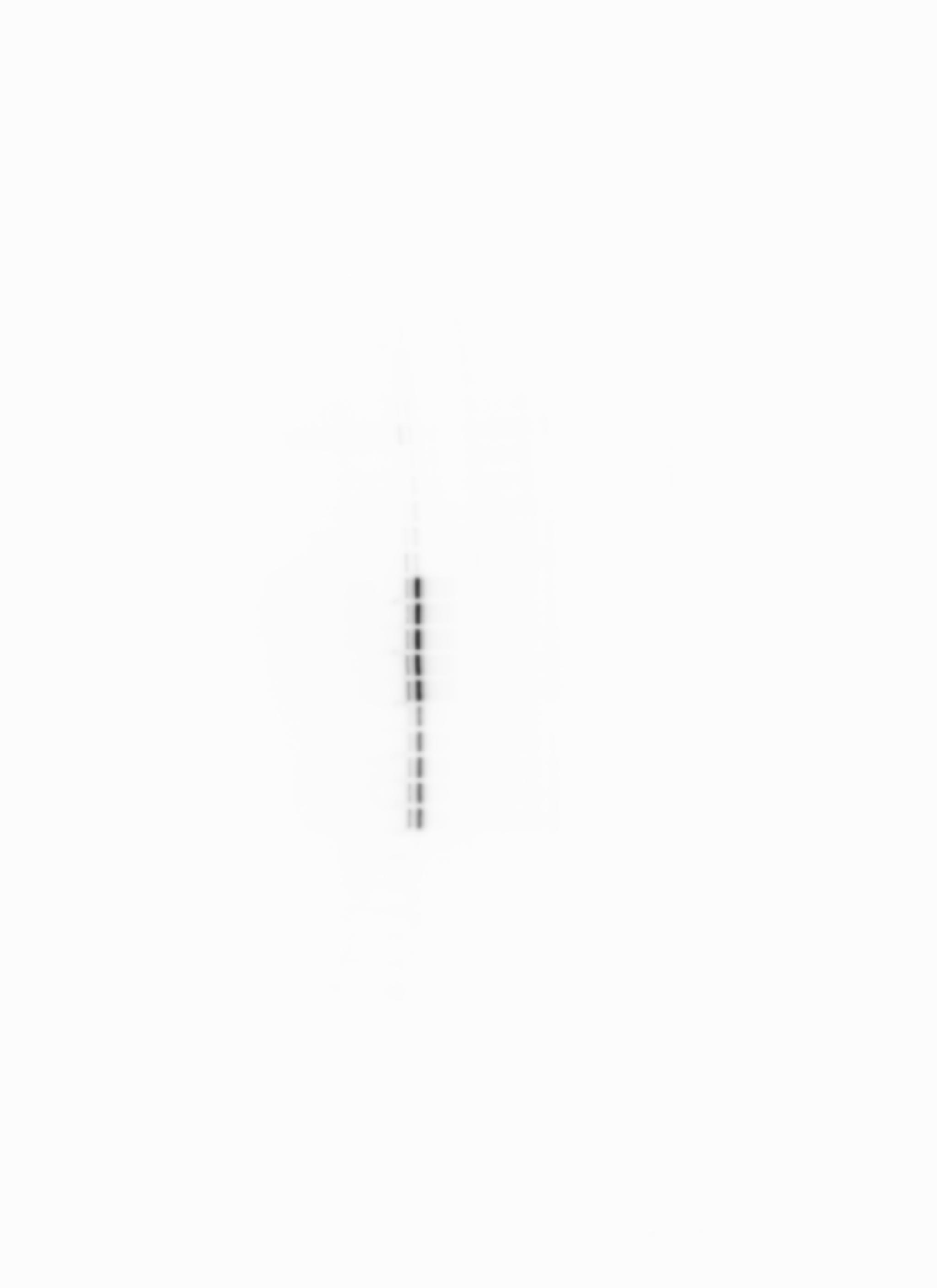

Supplement: Figure 1—source data 1. [file elife-81573-fig1-data1.zip › Figure 1-source data 1/Figure 1-source data 1_raw files/Figure 1E/SUN2 CHX chase 2022.03.02_19.40.39-01_Ch v HA short/SUN2 CHX chase 2022.03.02_19.40.39-01_Ch.jpg]

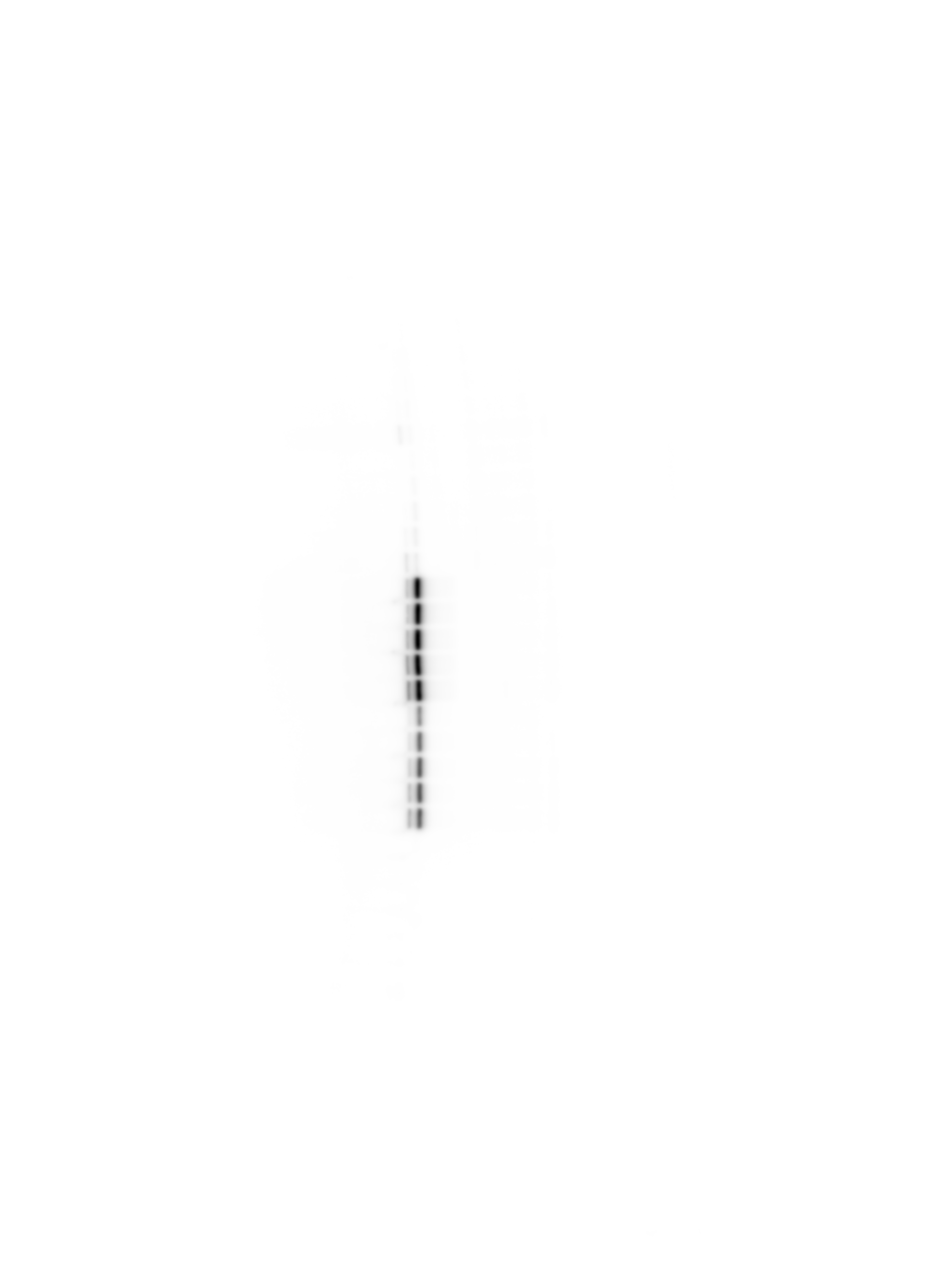

Supplement: Figure 1—source data 1. [file elife-81573-fig1-data1.zip › Figure 1-source data 1/Figure 1-source data 1_raw files/Figure 1E/SUN2 CHX chase 2022.03.02_19.40.39-01_Ch v HA short/SUN2 CHX chase 2022.03.02_19.40.39-01_Ch.tif]

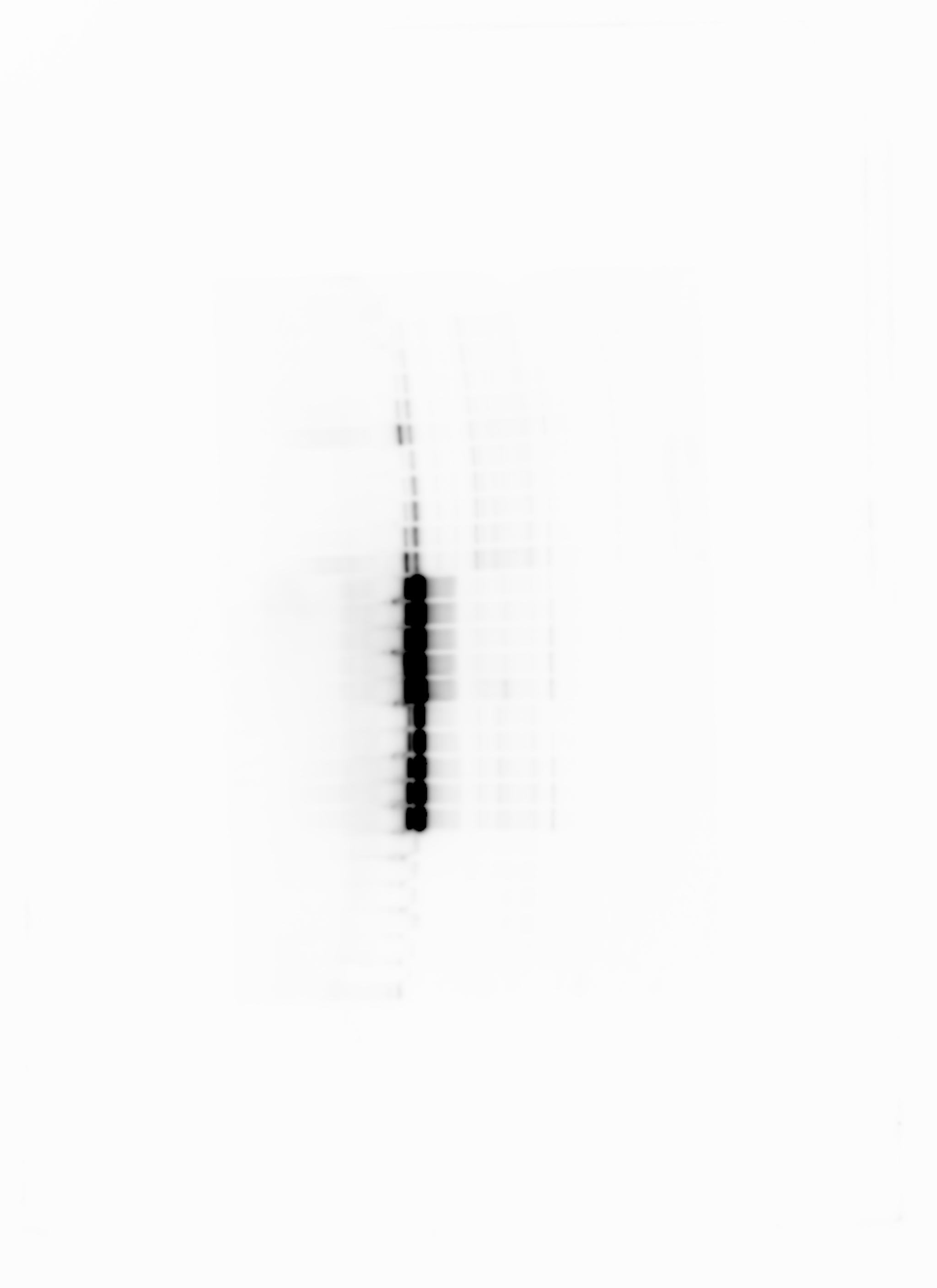

Supplement: Figure 1—source data 1. [file elife-81573-fig1-data1.zip › Figure 1-source data 1/Figure 1-source data 1_raw files/Figure 1E/SUN2 CHX chase 2022.03.02_19.46.10-06_Ch v HA long/SUN2 CHX chase 2022.03.02_19.46.10-06_Ch.jpg]

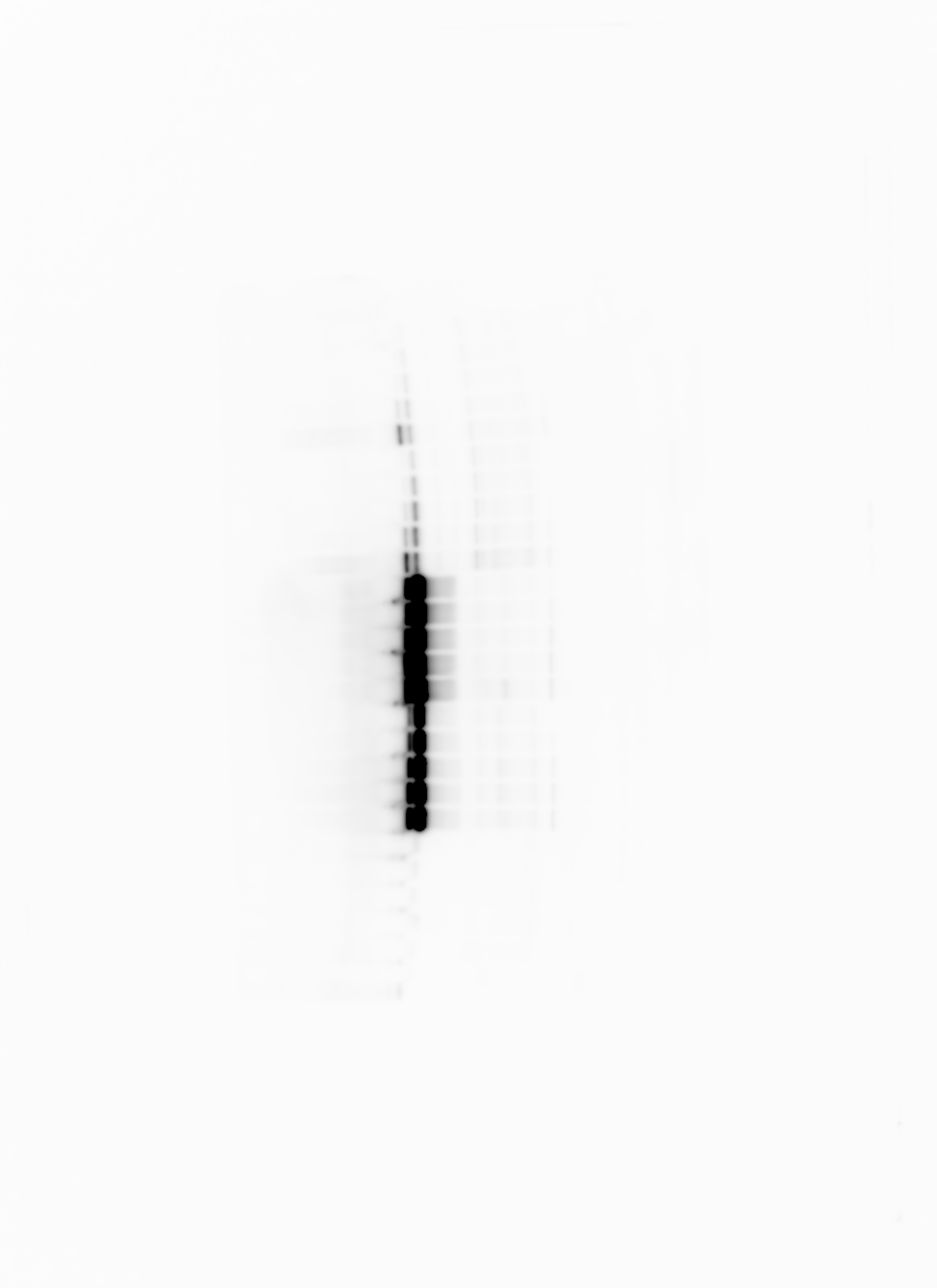

Supplement: Figure 1—source data 1. [file elife-81573-fig1-data1.zip › Figure 1-source data 1/Figure 1-source data 1_raw files/Figure 1E/SUN2 CHX chase 2022.03.02_19.46.10-06_Ch v HA long/SUN2 CHX chase 2022.03.02_19.46.10-06_Ch.tif]

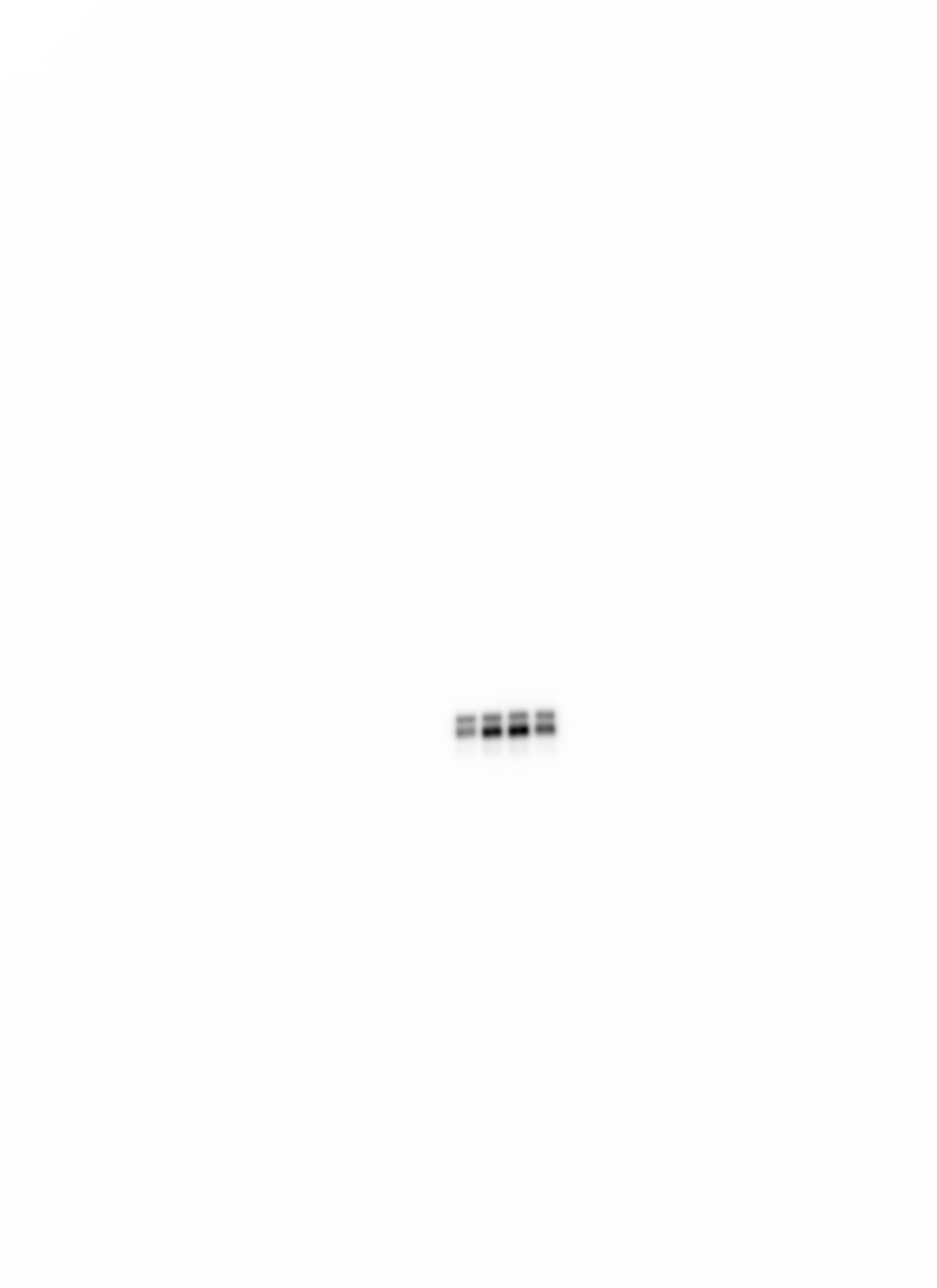

Supplement: Figure 2—source data 1. [file elife-81573-fig2-data1.zip › Figure 2-source data 1/Figure 2B/Figure 2-source data 1_raw files/LK220707 Fig2B HA 2022.07.07_22.17.50-05_Ch.tif]

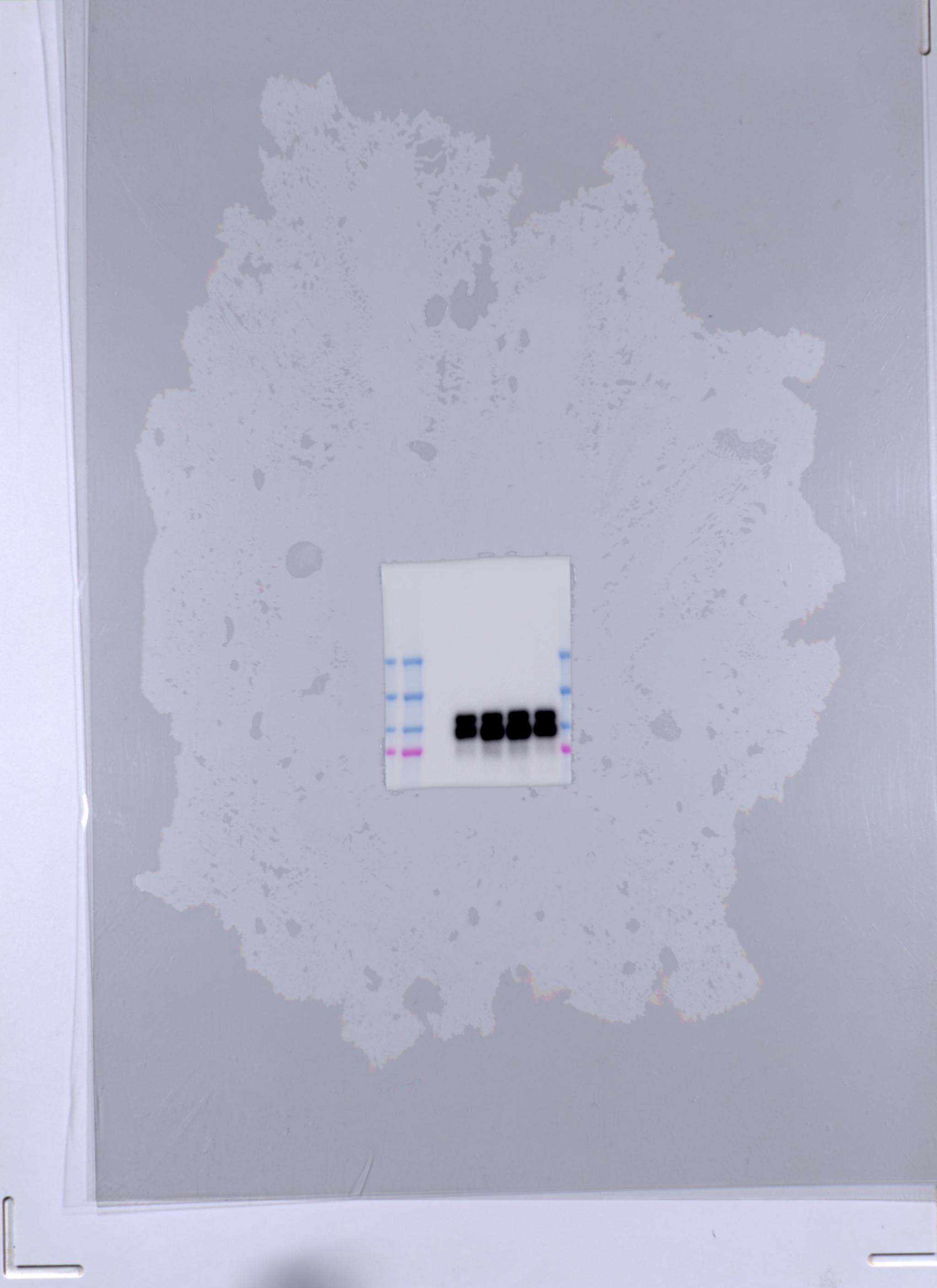

Supplement: Figure 2—source data 1. [file elife-81573-fig2-data1.zip › Figure 2-source data 1/Figure 2B/Figure 2-source data 1_raw files/LK220707 Fig2B HA 2022.07.07_22.22.02_Ch+Marker.jpg]

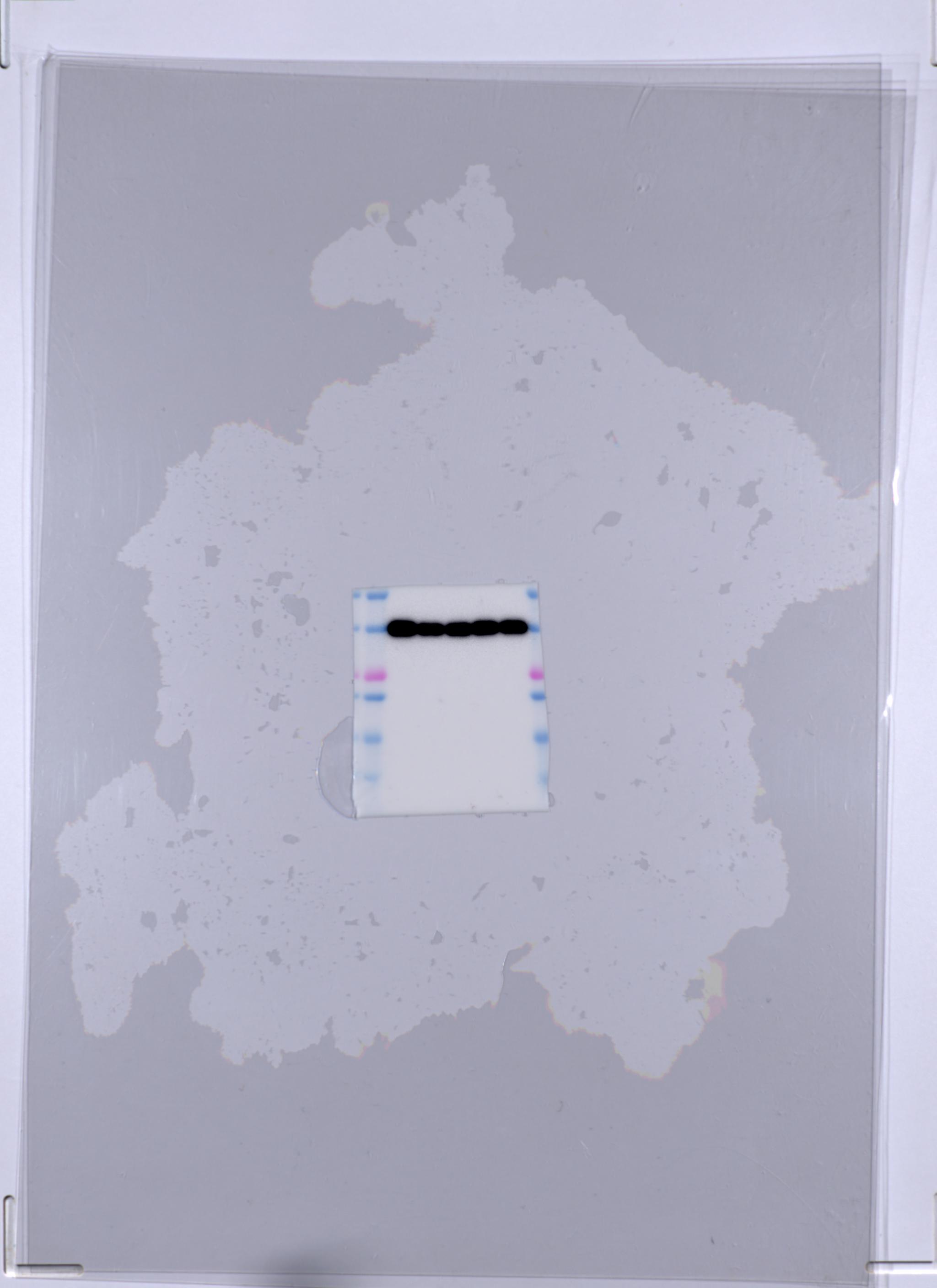

Supplement: Figure 2—source data 1. [file elife-81573-fig2-data1.zip › Figure 2-source data 1/Figure 2B/Figure 2-source data 1_raw files/LK220707 Fig2B Gpdh 2022.07.07_22.08.36_Ch+Marker.jpg]

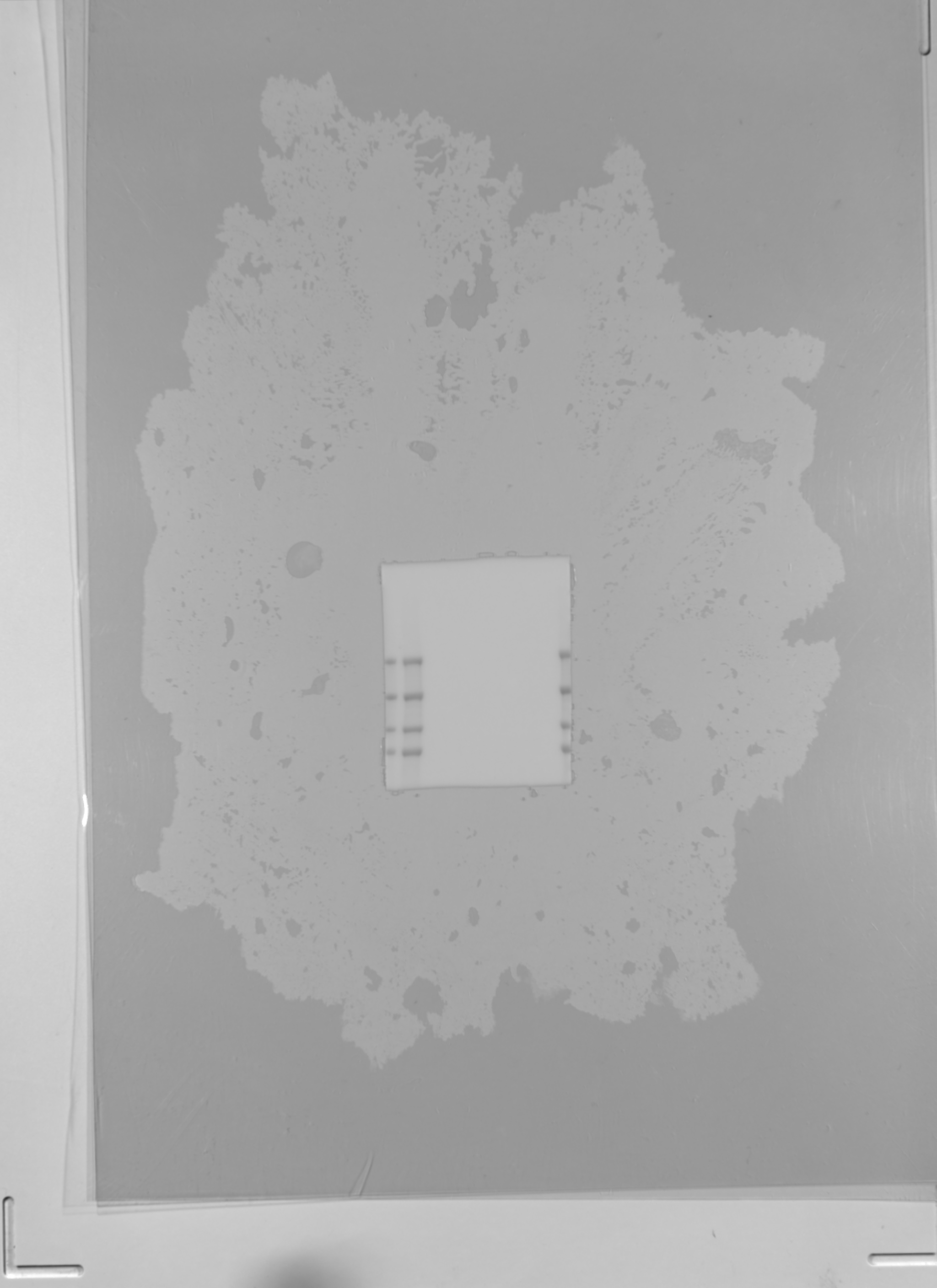

Supplement: Figure 2—source data 1. [file elife-81573-fig2-data1.zip › Figure 2-source data 1/Figure 2B/Figure 2-source data 1_raw files/LK220707 Fig2B HA 2022.07.07_22.22.02_Ch-Marker.tif]

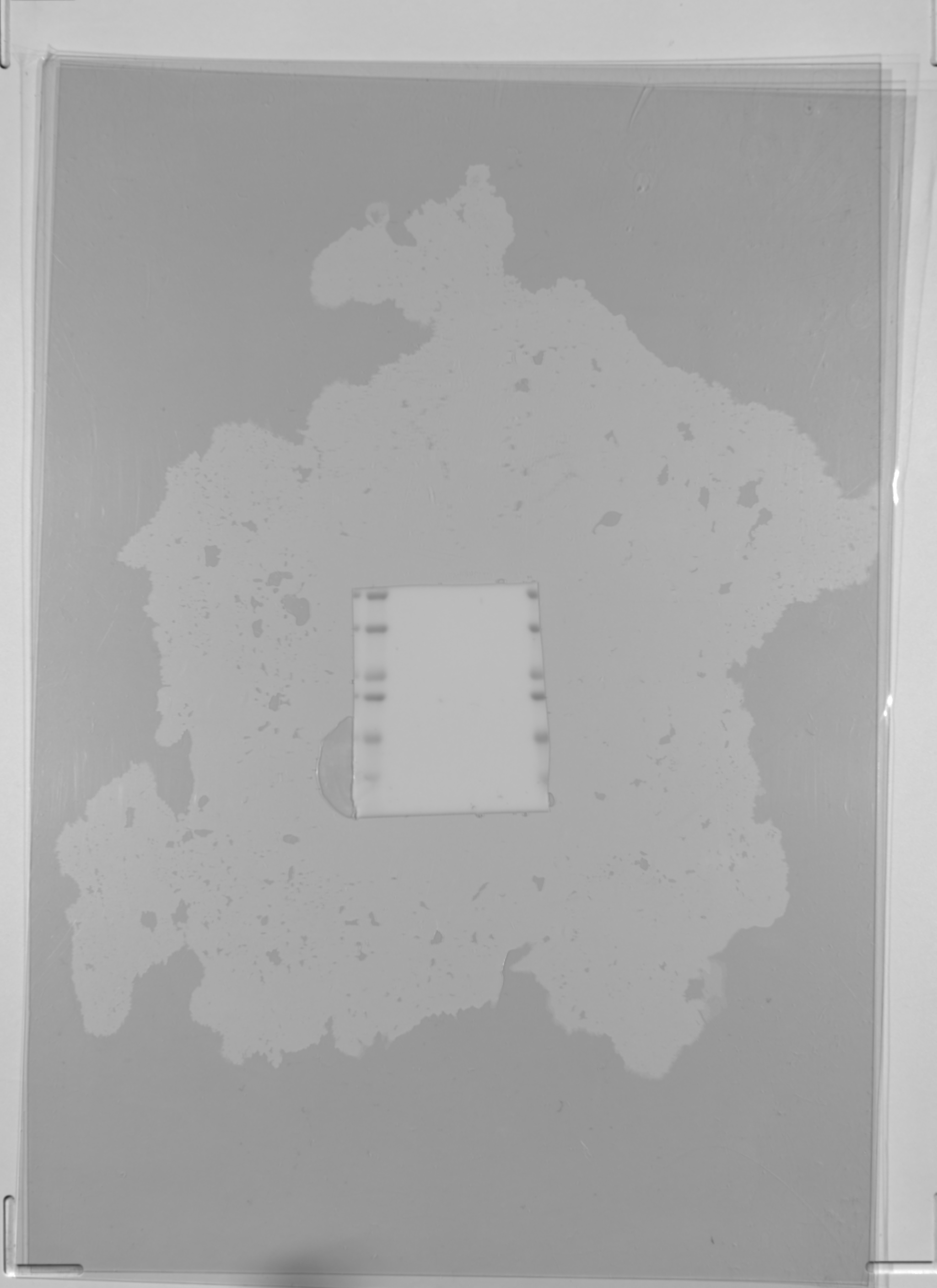

Supplement: Figure 2—source data 1. [file elife-81573-fig2-data1.zip › Figure 2-source data 1/Figure 2B/Figure 2-source data 1_raw files/LK220707 Fig2B Gpdh 2022.07.07_22.08.36_Ch-Marker.tif]

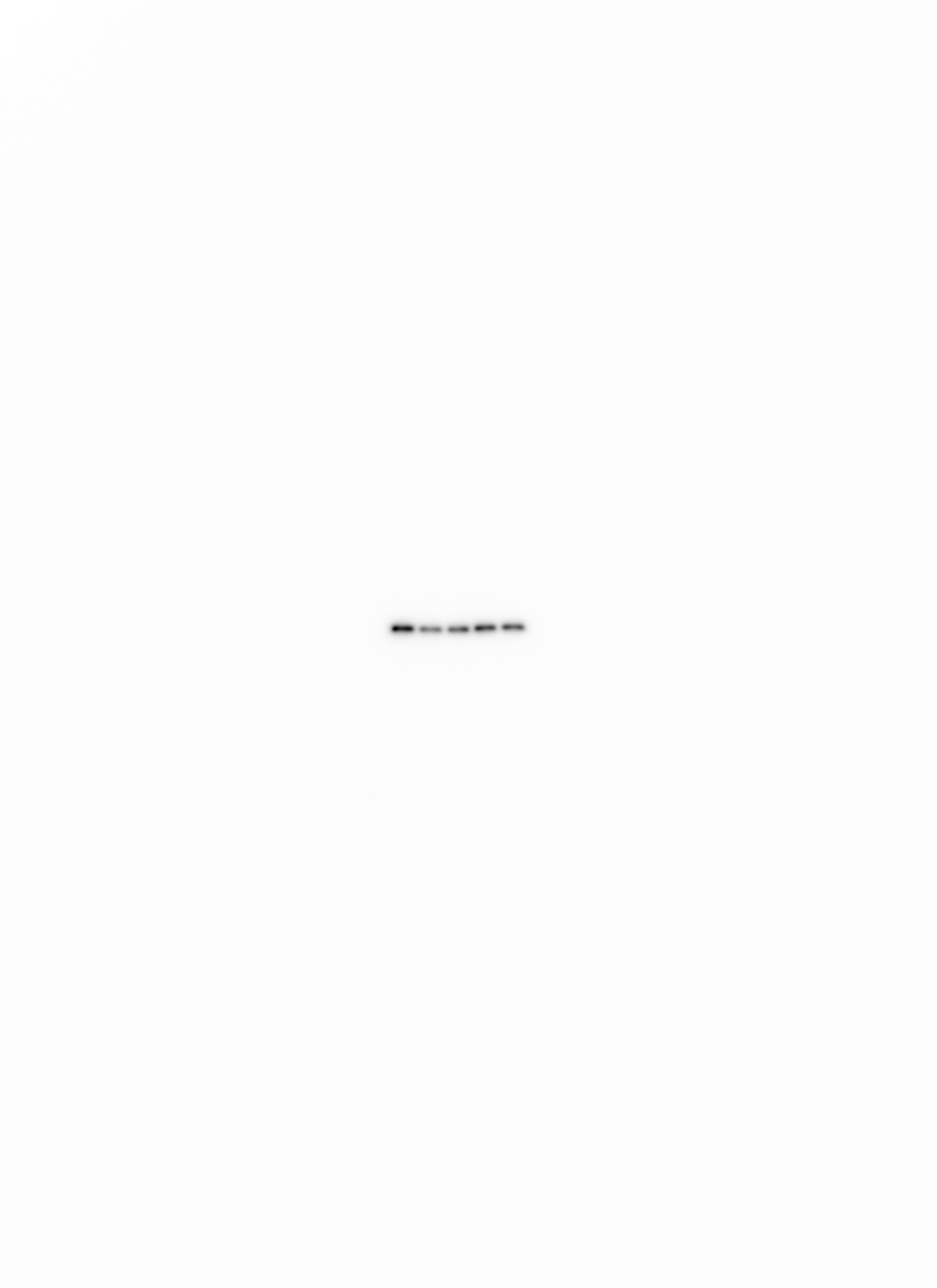

Supplement: Figure 2—source data 1. [file elife-81573-fig2-data1.zip › Figure 2-source data 1/Figure 2B/Figure 2-source data 1_raw files/LK220707 Fig2B Gpdh 2022.07.07_22.02.55-05_Ch.tif]

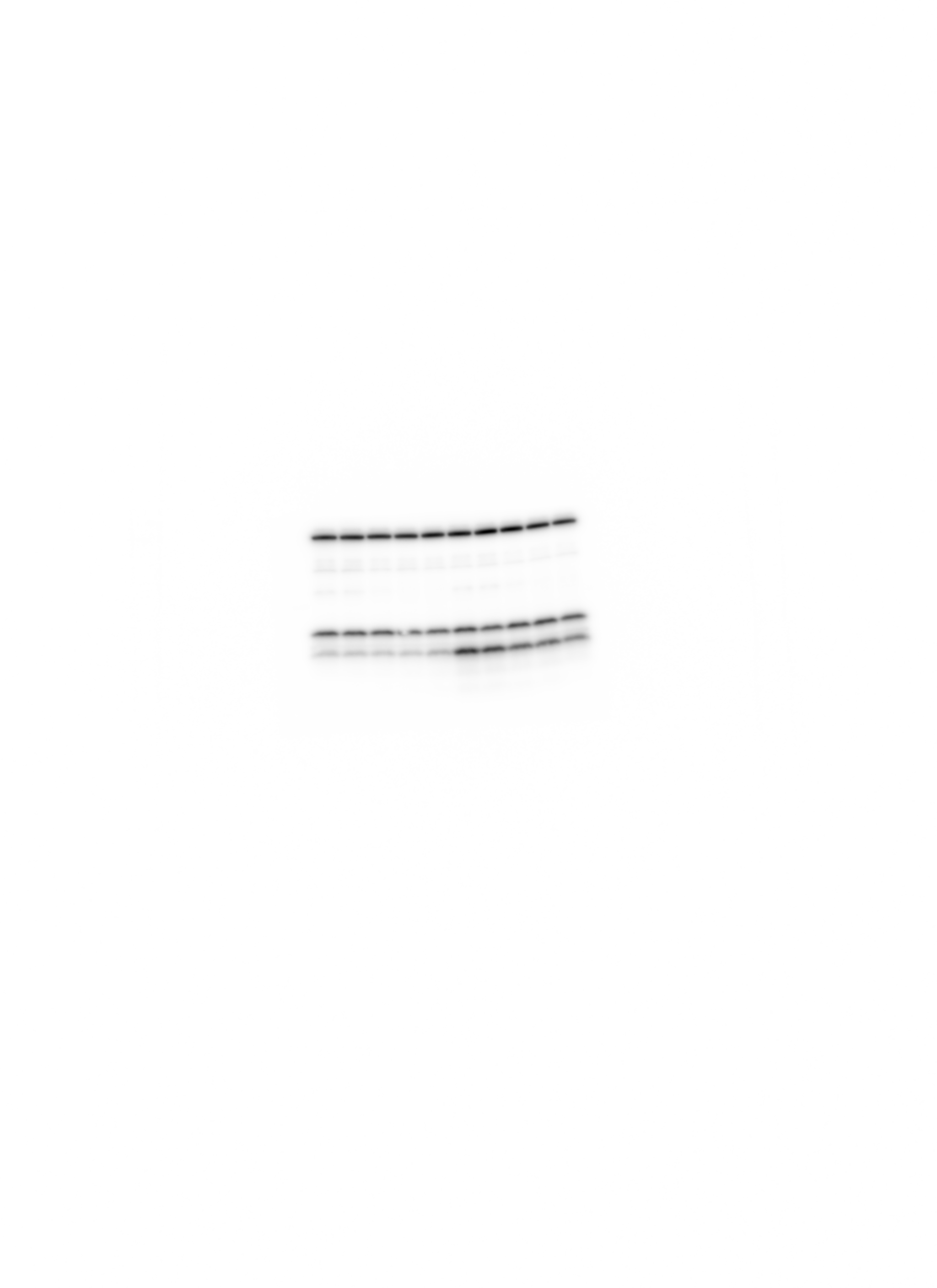

Supplement: Figure 2—source data 2. [file elife-81573-fig2-data2.zip › Figure 2-source data 2/Figure 2C/Figure 2-source data 2_raw files/LK220309 BafA1 LC3B 2022.03.09_15.39.50-01_Ch.tif]

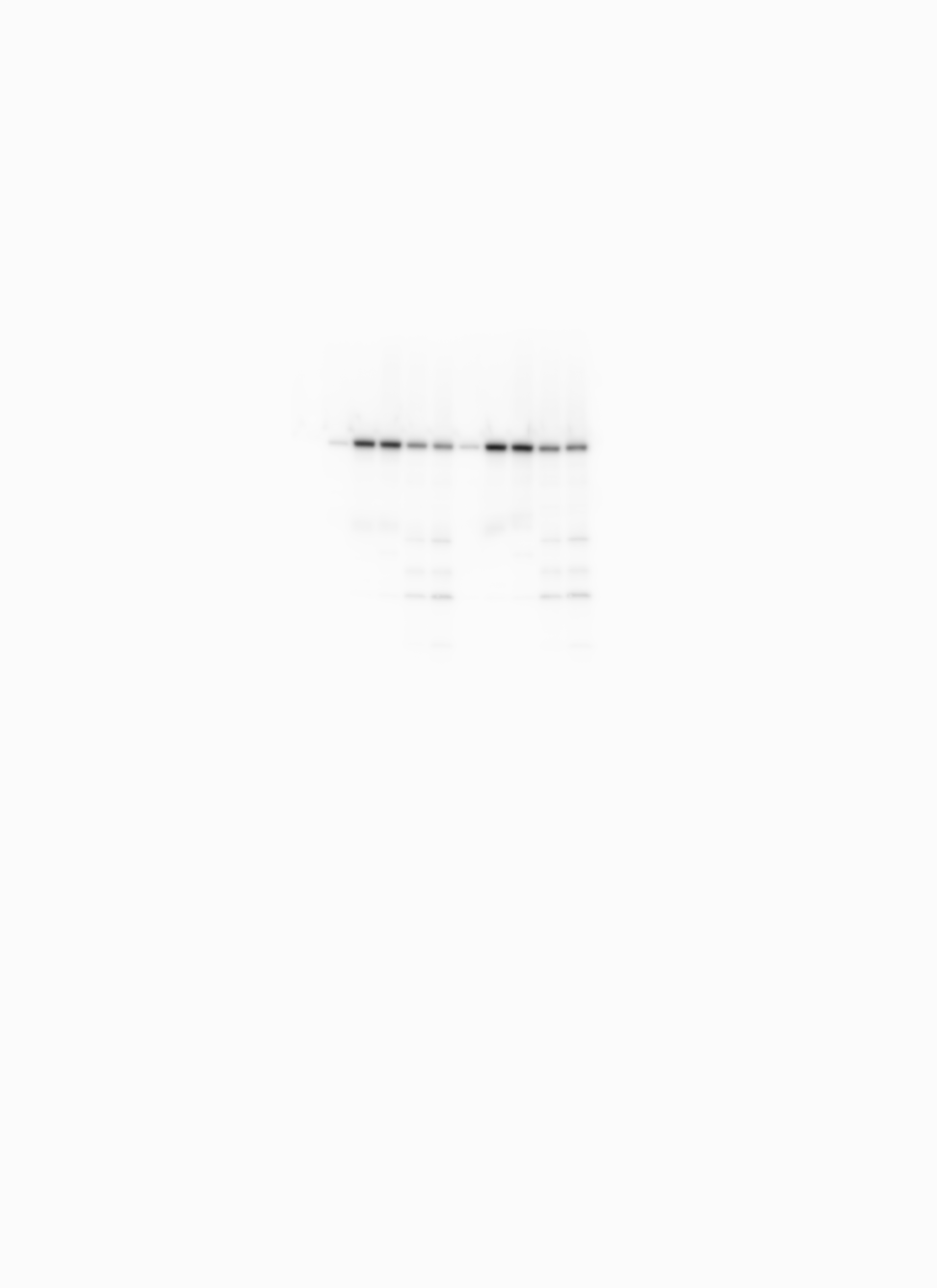

Supplement: Figure 2—source data 2. [file elife-81573-fig2-data2.zip › Figure 2-source data 2/Figure 2C/Figure 2-source data 2_raw files/LK220303 AspBaf HA 2022.03.03_17.22.27-01_Ch.tif]

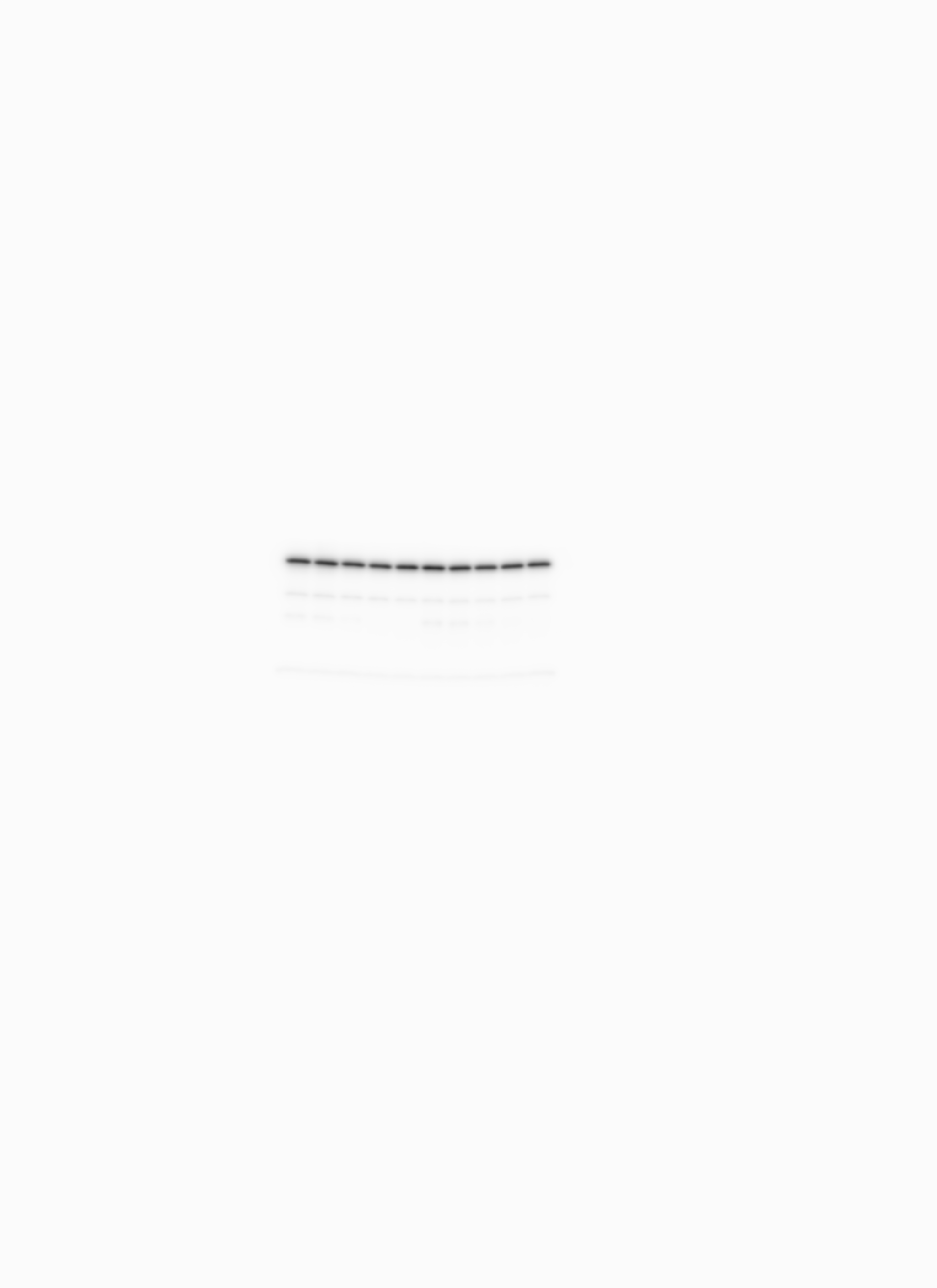

Supplement: Figure 2—source data 2. [file elife-81573-fig2-data2.zip › Figure 2-source data 2/Figure 2C/Figure 2-source data 2_raw files/LK220303 AspBaf gpdh 2022.03.03_18.08.03-01_Ch.tif]

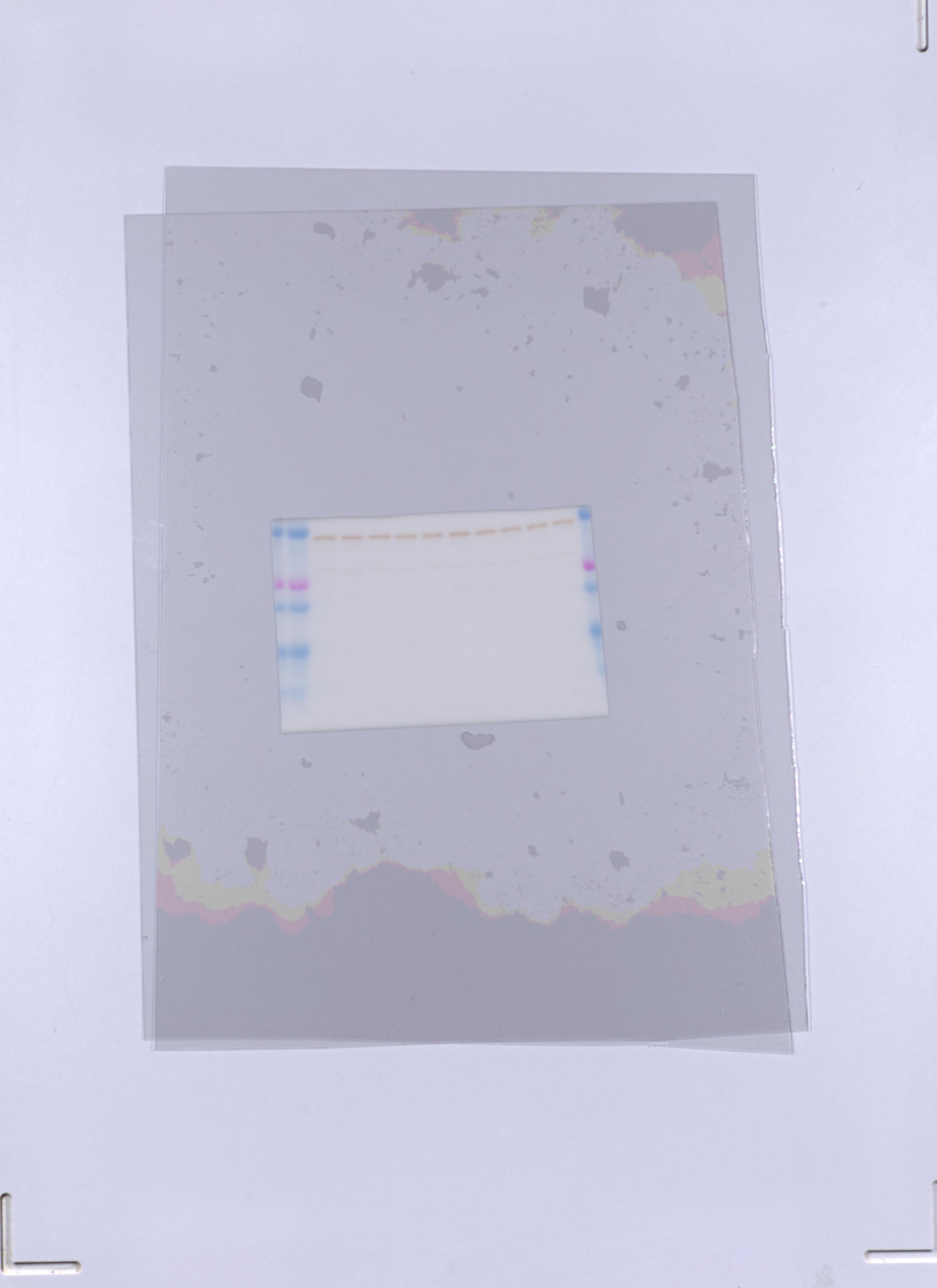

Supplement: Figure 2—source data 2. [file elife-81573-fig2-data2.zip › Figure 2-source data 2/Figure 2C/Figure 2-source data 2_raw files/LK220309 BafA1 LC3B 2022.03.09_15.37.24_Ch-Marker.jpg]

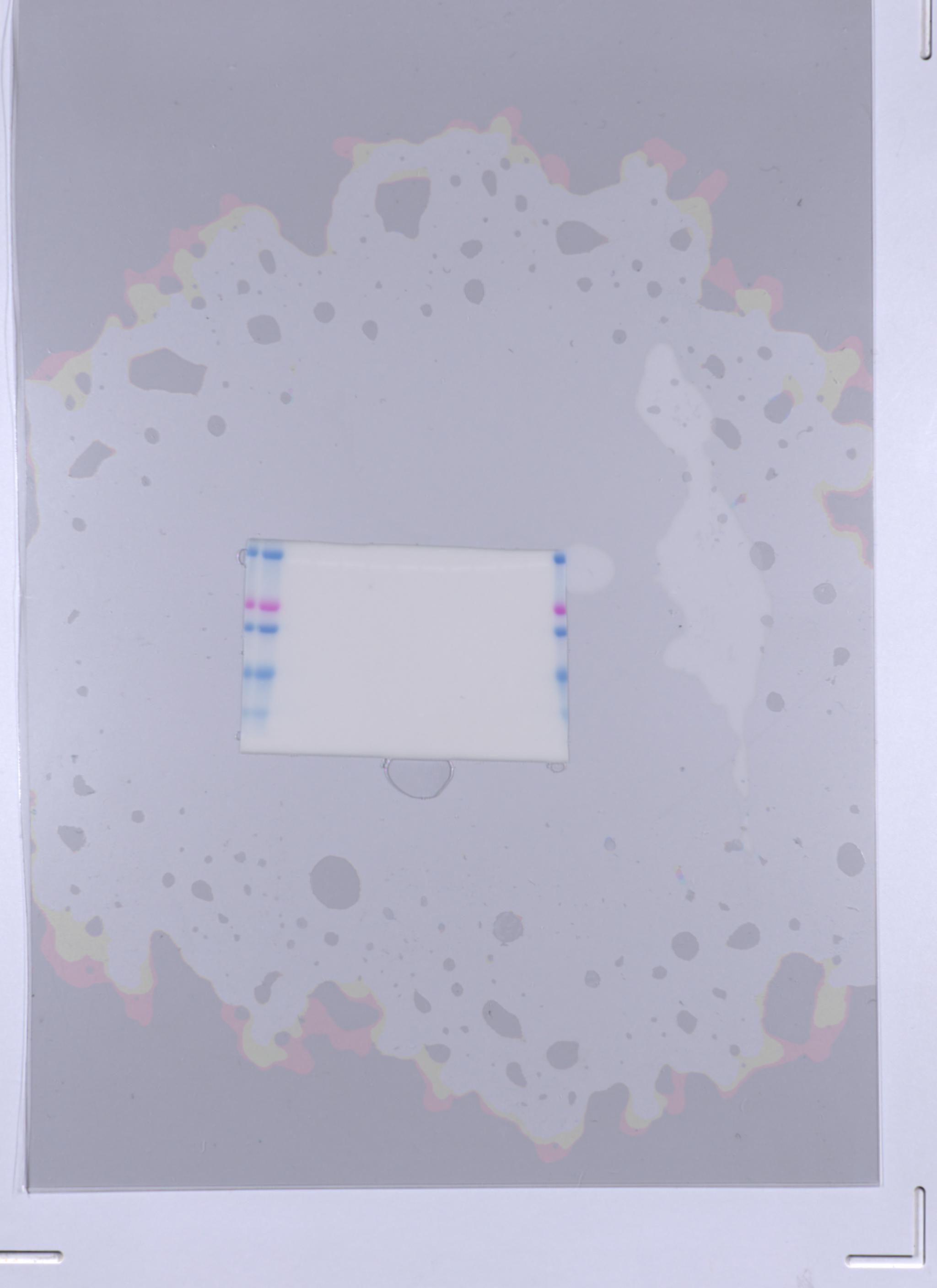

Supplement: Figure 2—source data 2. [file elife-81573-fig2-data2.zip › Figure 2-source data 2/Figure 2C/Figure 2-source data 2_raw files/LK220303 AspBaf gpdh 2022.03.03_18.06.16_Ch-Marker.jpg]

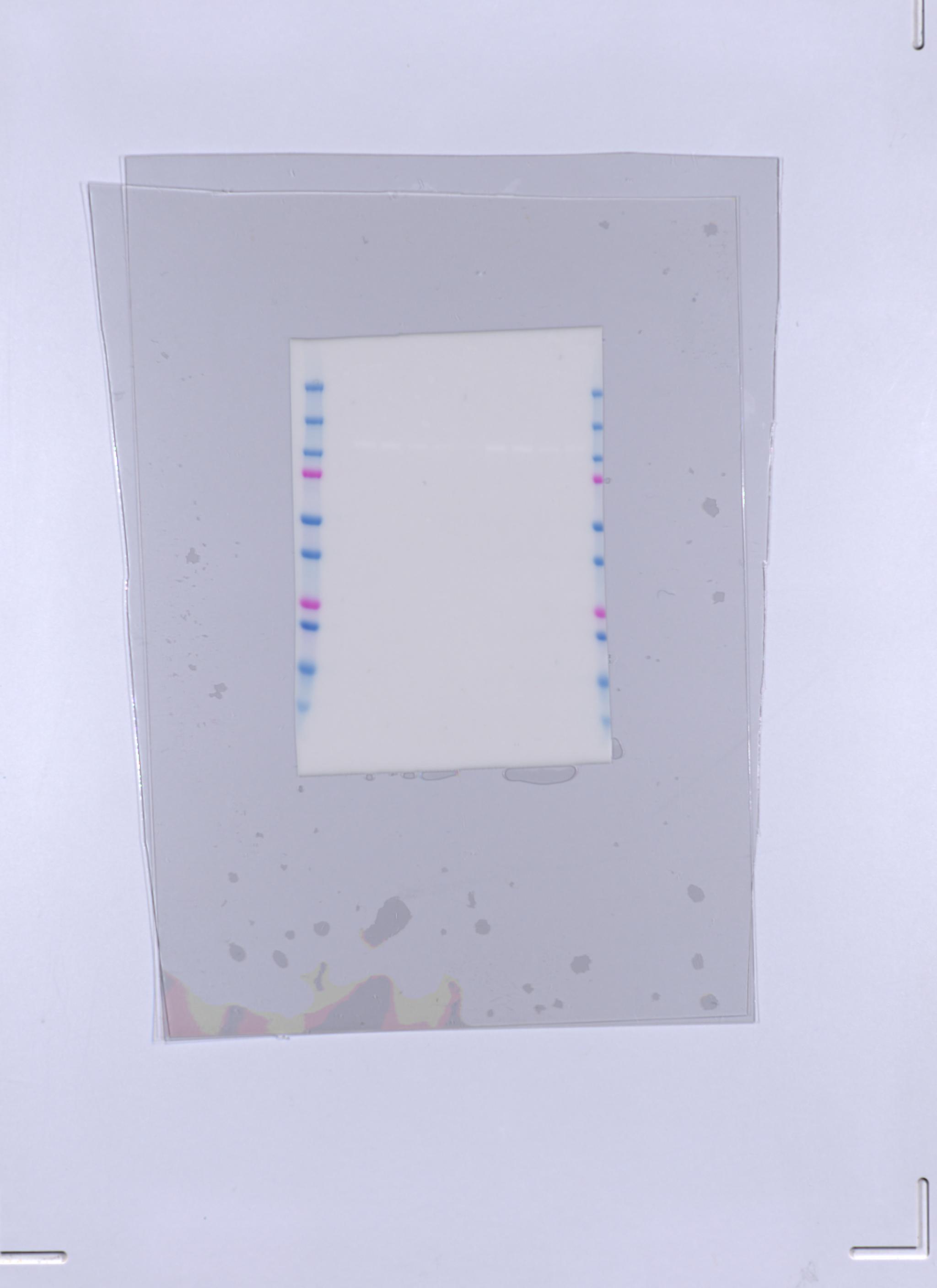

Supplement: Figure 2—source data 2. [file elife-81573-fig2-data2.zip › Figure 2-source data 2/Figure 2C/Figure 2-source data 2_raw files/LK220303 AspBaf HA 2022.03.03_17.17.07_Ch-Marker.jpg]

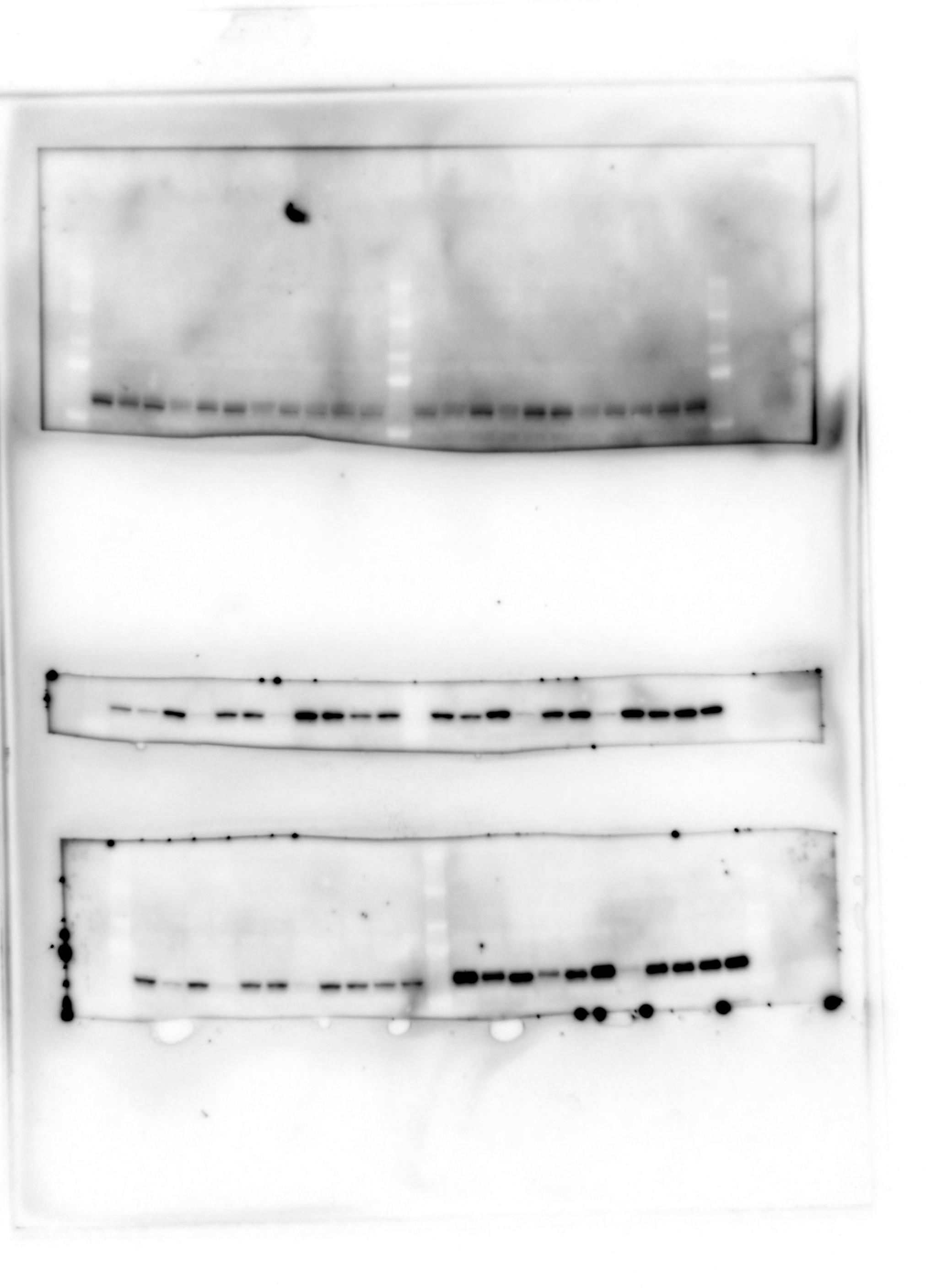

Supplement: Figure 2—figure supplement 1—source data 1. [file elife-81573-fig2-figsupp1-data1.zip › Figure 2-supplement 1-source data 1/Figure 2-supplement 1-source data 1_raw files/ws2 p+tran btrcp1 2022.09.15_15.00.39-12_Ch.jpg]

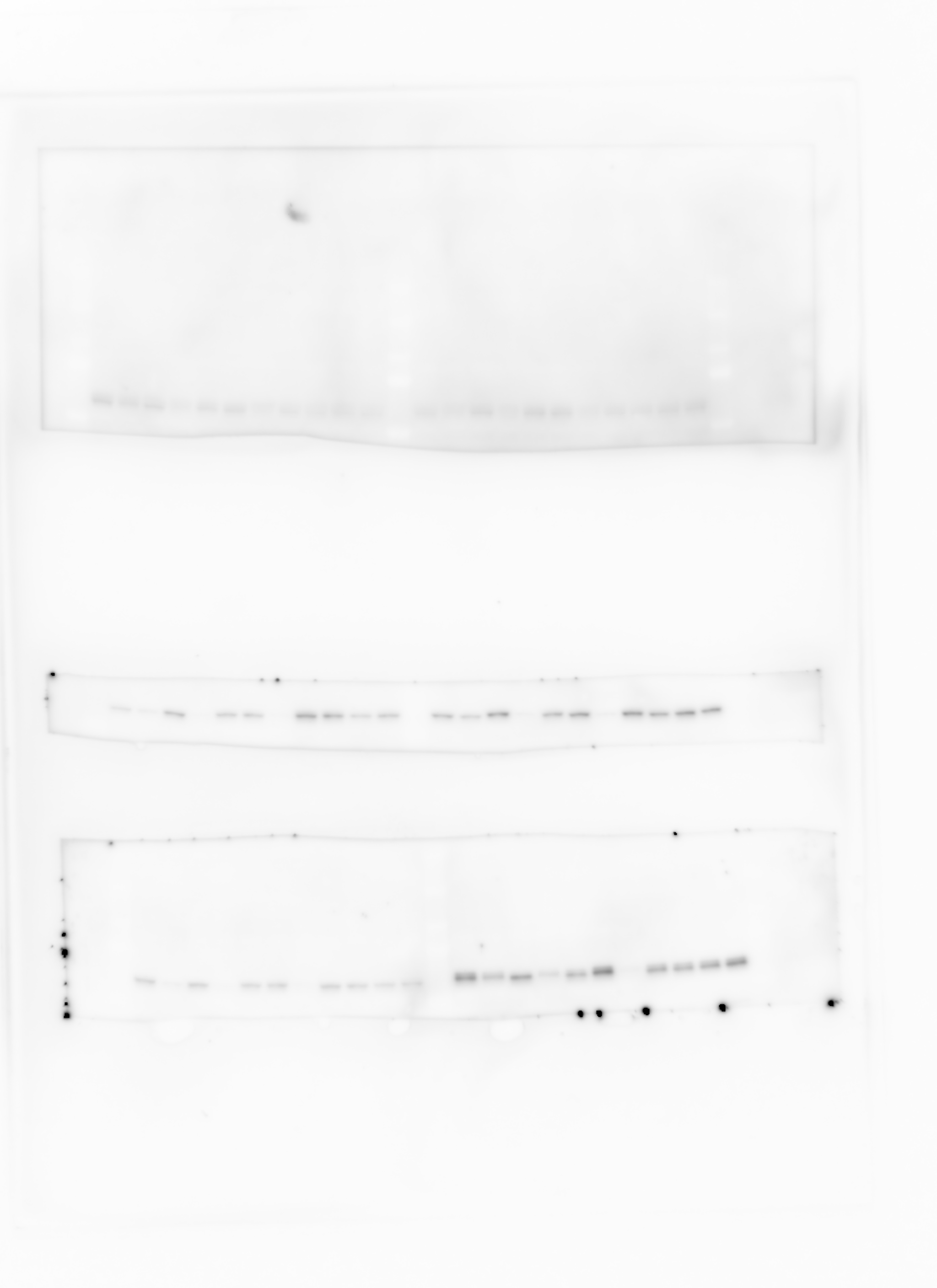

Supplement: Figure 2—figure supplement 1—source data 1. [file elife-81573-fig2-figsupp1-data1.zip › Figure 2-supplement 1-source data 1/Figure 2-supplement 1-source data 1_raw files/ws2 p+tran btrcp1 2022.09.15_15.00.39-12_Ch.tif]

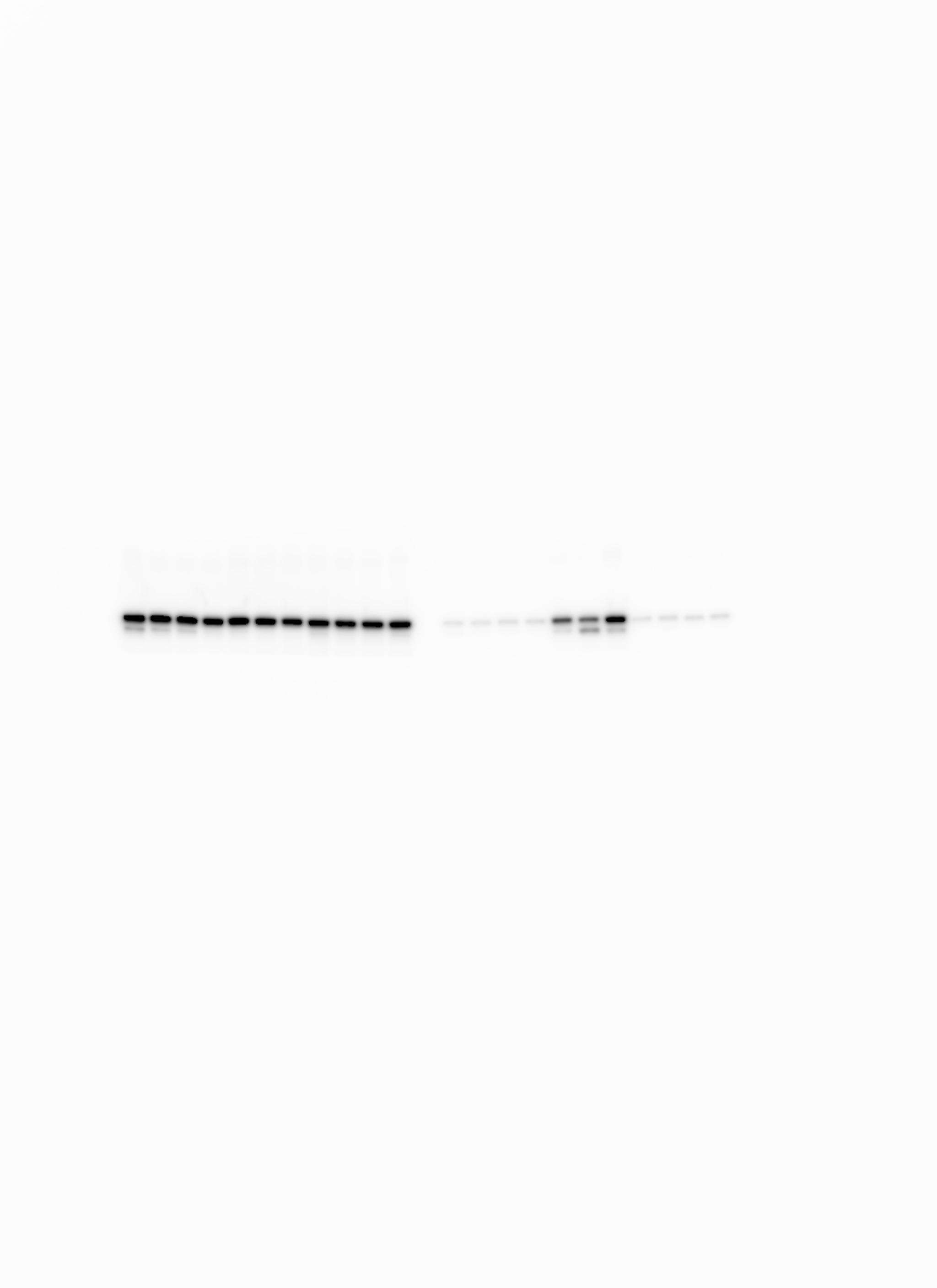

Supplement: Figure 2—figure supplement 1—source data 1. [file elife-81573-fig2-figsupp1-data1.zip › Figure 2-supplement 1-source data 1/Figure 2-supplement 1-source data 1_raw files/ws2 sun2gfp HA 2022.09.14_16.44.22-04_Ch/ws2 sun2gfp HA 2022.09.14_16.44.22-04_Ch.jpg]

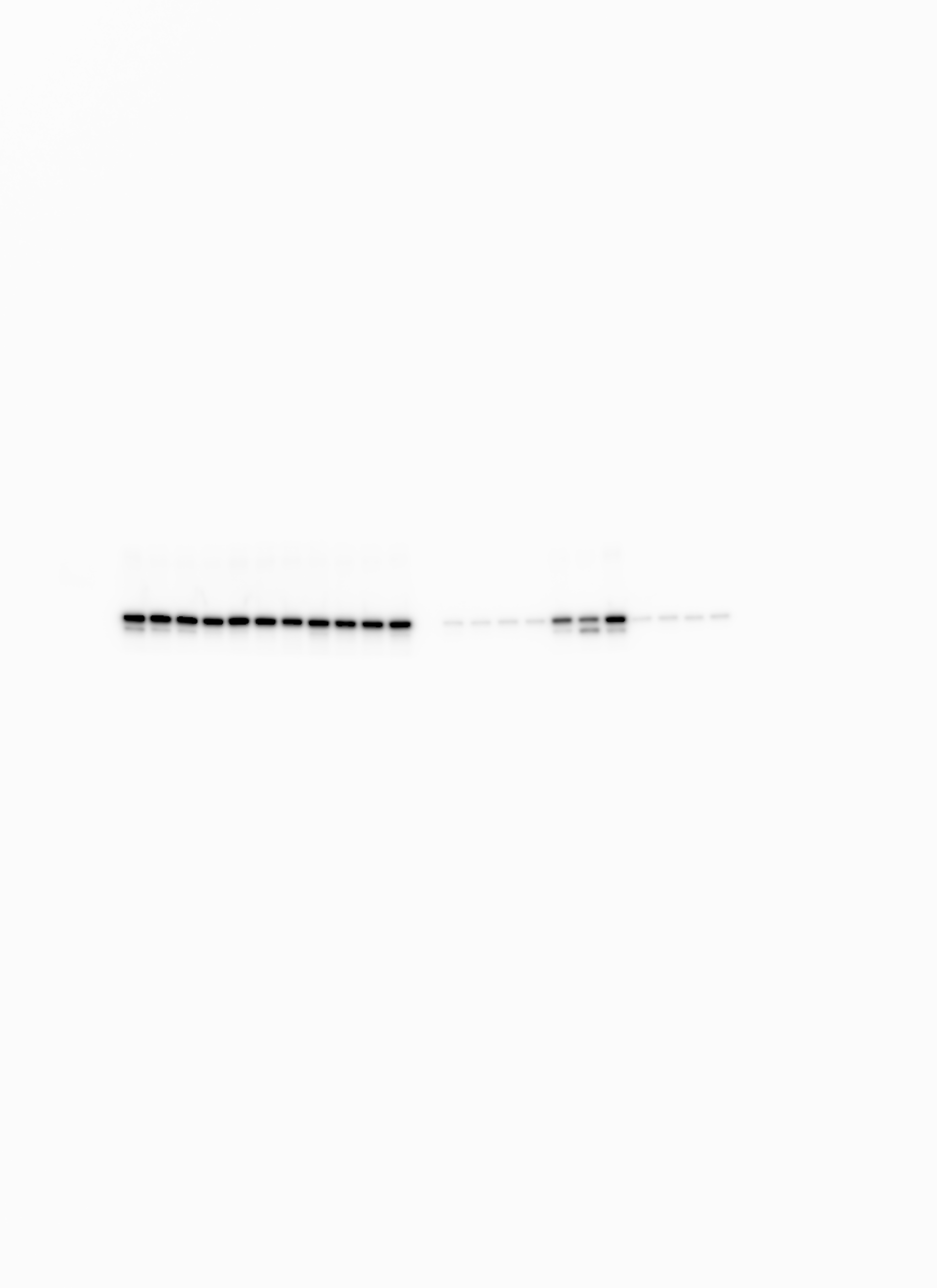

Supplement: Figure 2—figure supplement 1—source data 1. [file elife-81573-fig2-figsupp1-data1.zip › Figure 2-supplement 1-source data 1/Figure 2-supplement 1-source data 1_raw files/ws2 sun2gfp HA 2022.09.14_16.44.22-04_Ch/ws2 sun2gfp HA 2022.09.14_16.44.22-04_Ch.tif]

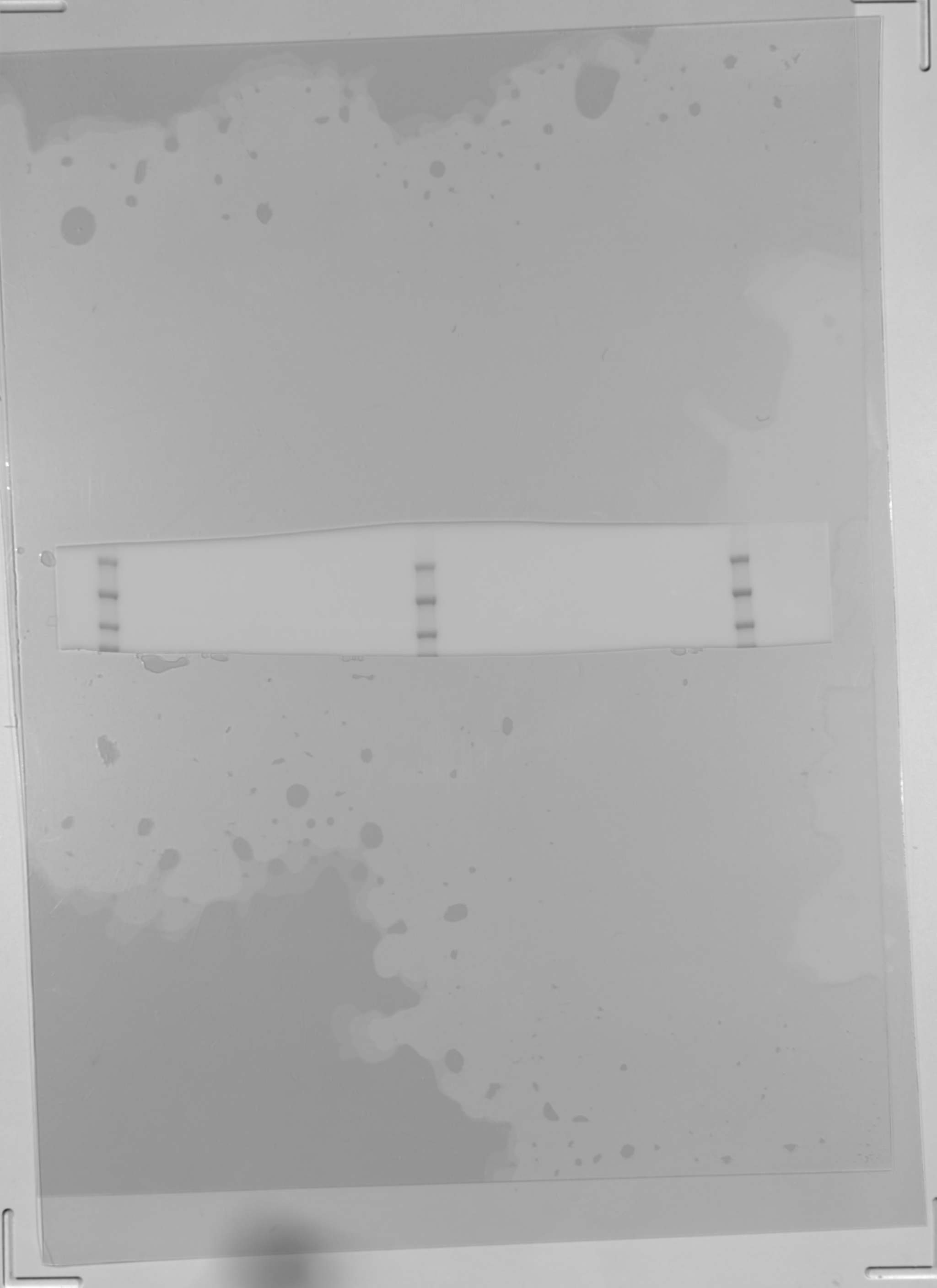

Supplement: Figure 2—figure supplement 1—source data 1. [file elife-81573-fig2-figsupp1-data1.zip › Figure 2-supplement 1-source data 1/Figure 2-supplement 1-source data 1_raw files/ws2 sun2gfp HA 2022.09.14_16.42.33_Ch/ws2 sun2gfp HA 2022.09.14_16.42.33_Ch-Marker.tif]

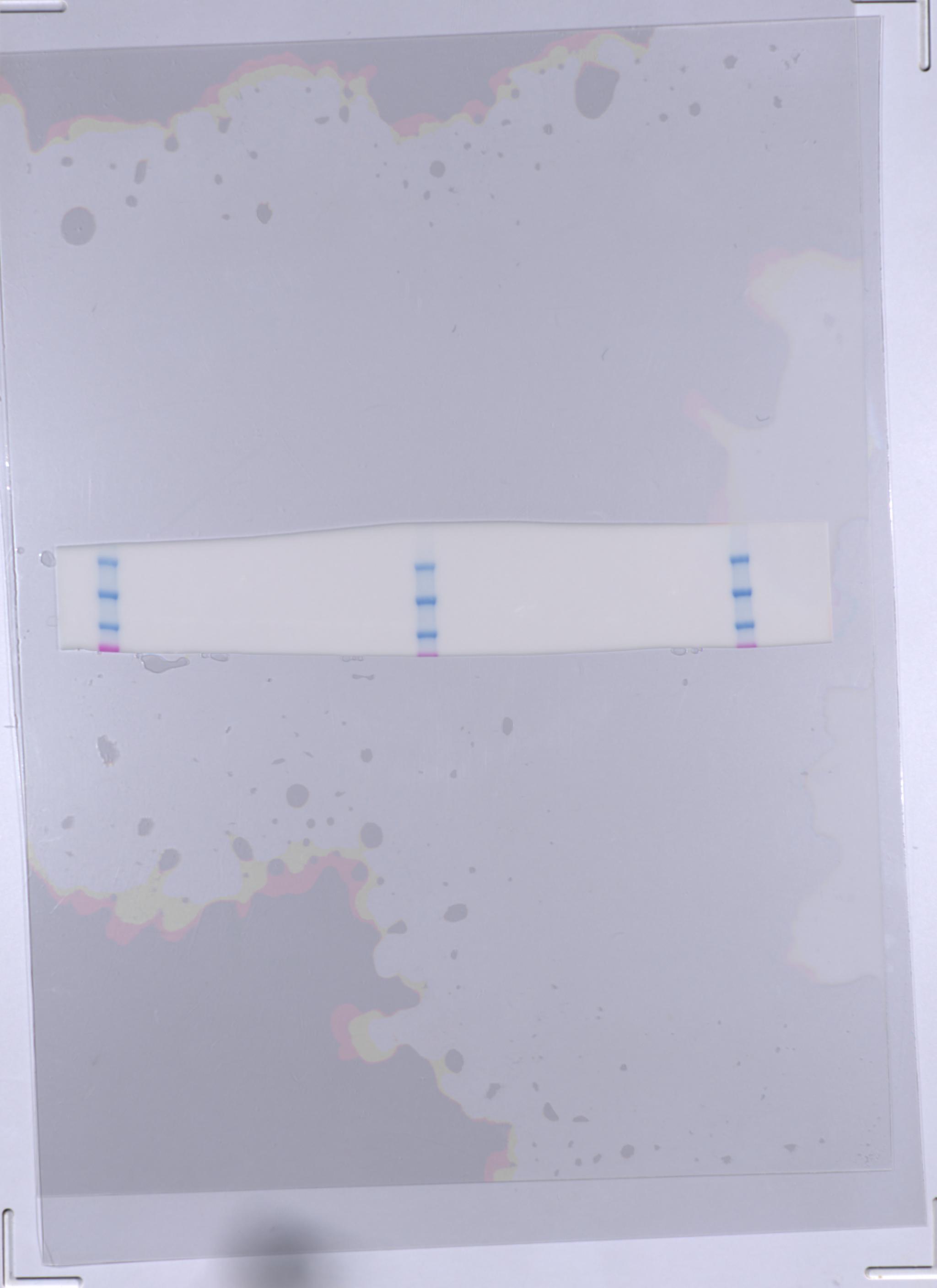

Supplement: Figure 2—figure supplement 1—source data 1. [file elife-81573-fig2-figsupp1-data1.zip › Figure 2-supplement 1-source data 1/Figure 2-supplement 1-source data 1_raw files/ws2 sun2gfp HA 2022.09.14_16.42.33_Ch/ws2 sun2gfp HA 2022.09.14_16.42.33_Ch-Marker.jpg]

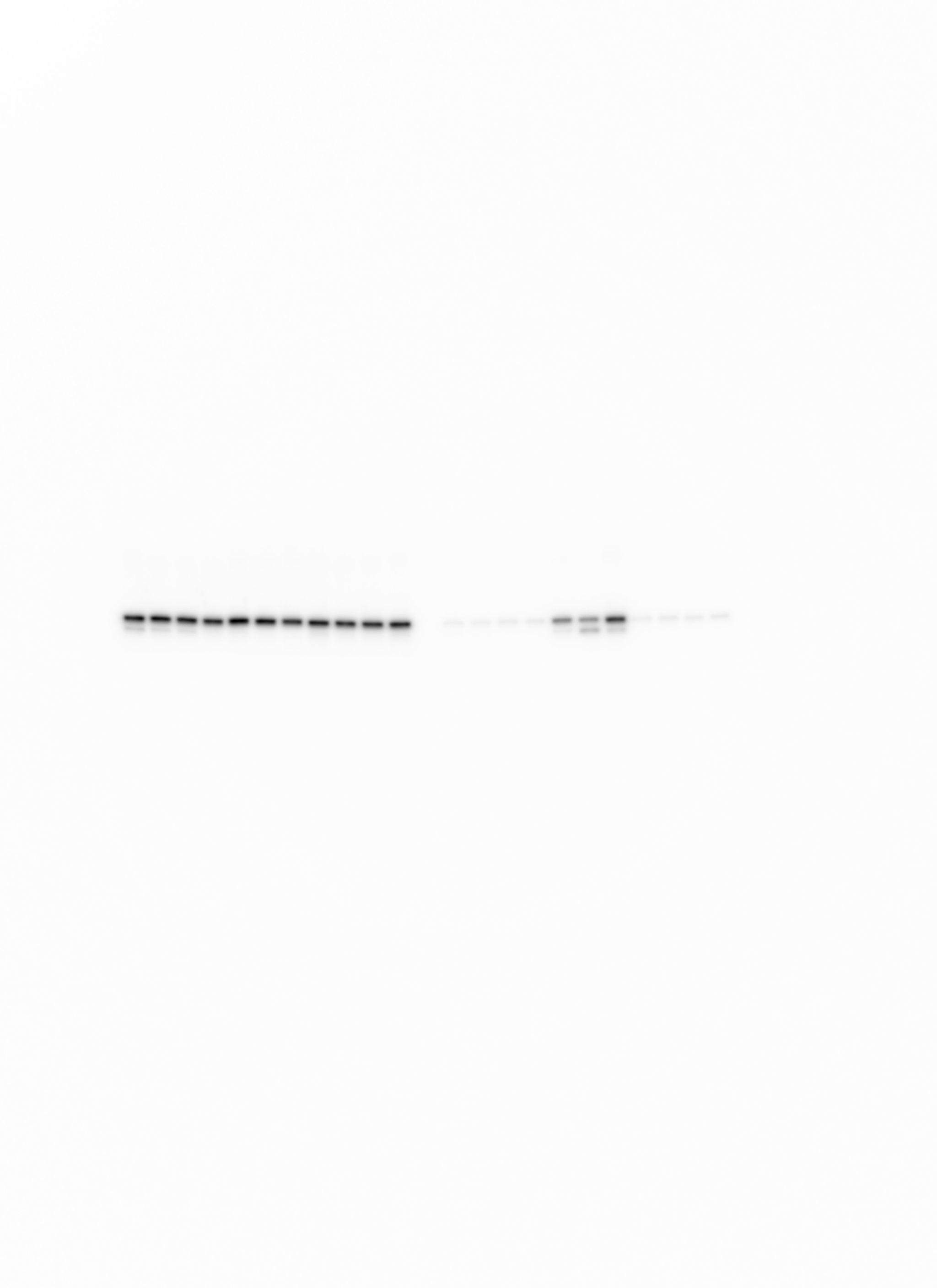

Supplement: Figure 2—figure supplement 1—source data 1. [file elife-81573-fig2-figsupp1-data1.zip › Figure 2-supplement 1-source data 1/Figure 2-supplement 1-source data 1_raw files/ws2 sun2gfp HA 2022.09.14_16.42.33_Ch/ws2 sun2gfp HA 2022.09.14_16.42.33_Ch.tif]

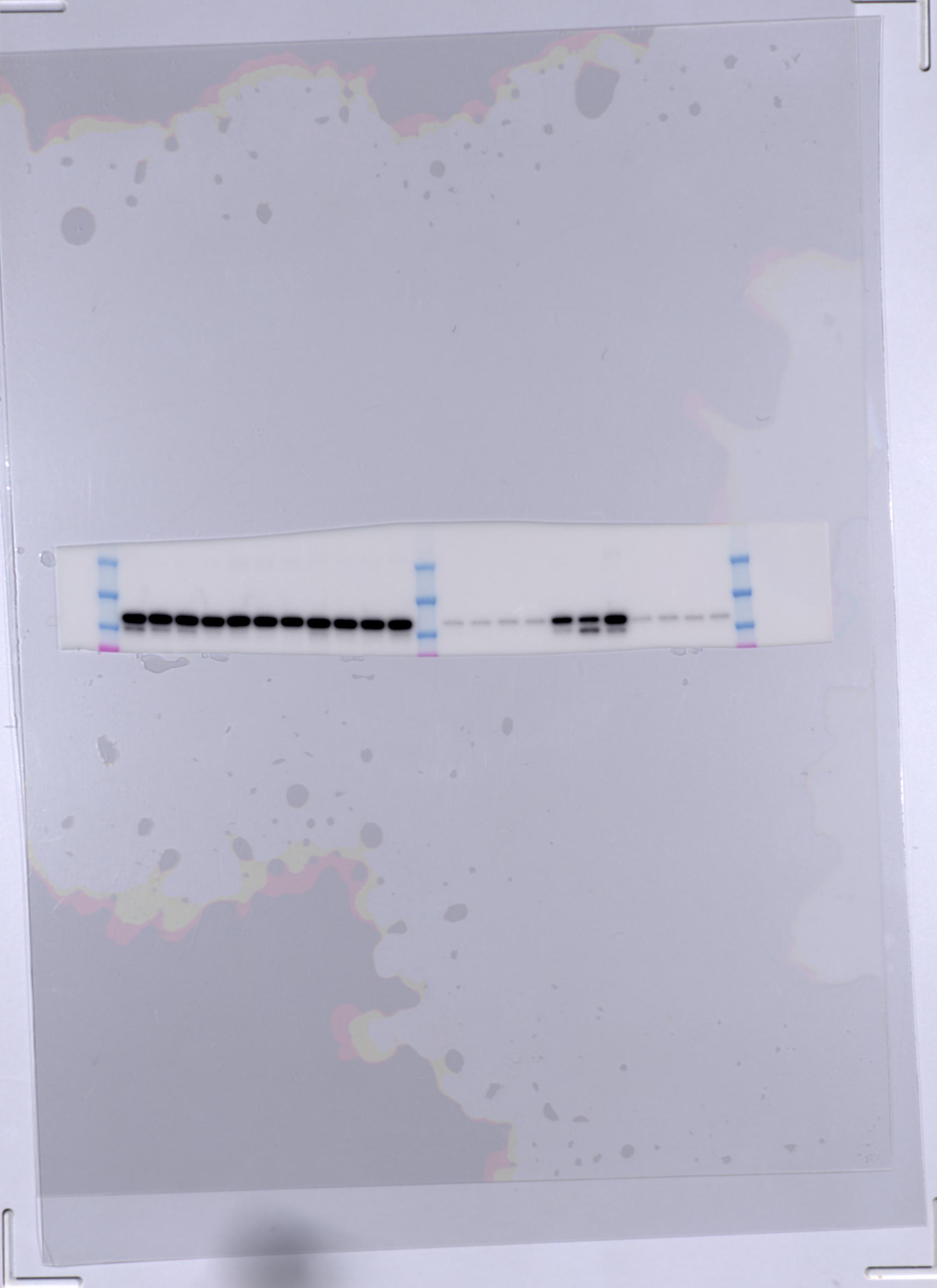

Supplement: Figure 2—figure supplement 1—source data 1. [file elife-81573-fig2-figsupp1-data1.zip › Figure 2-supplement 1-source data 1/Figure 2-supplement 1-source data 1_raw files/ws2 sun2gfp HA 2022.09.14_16.42.33_Ch/ws2 sun2gfp HA 2022.09.14_16.42.33_Ch+Marker.jpg]

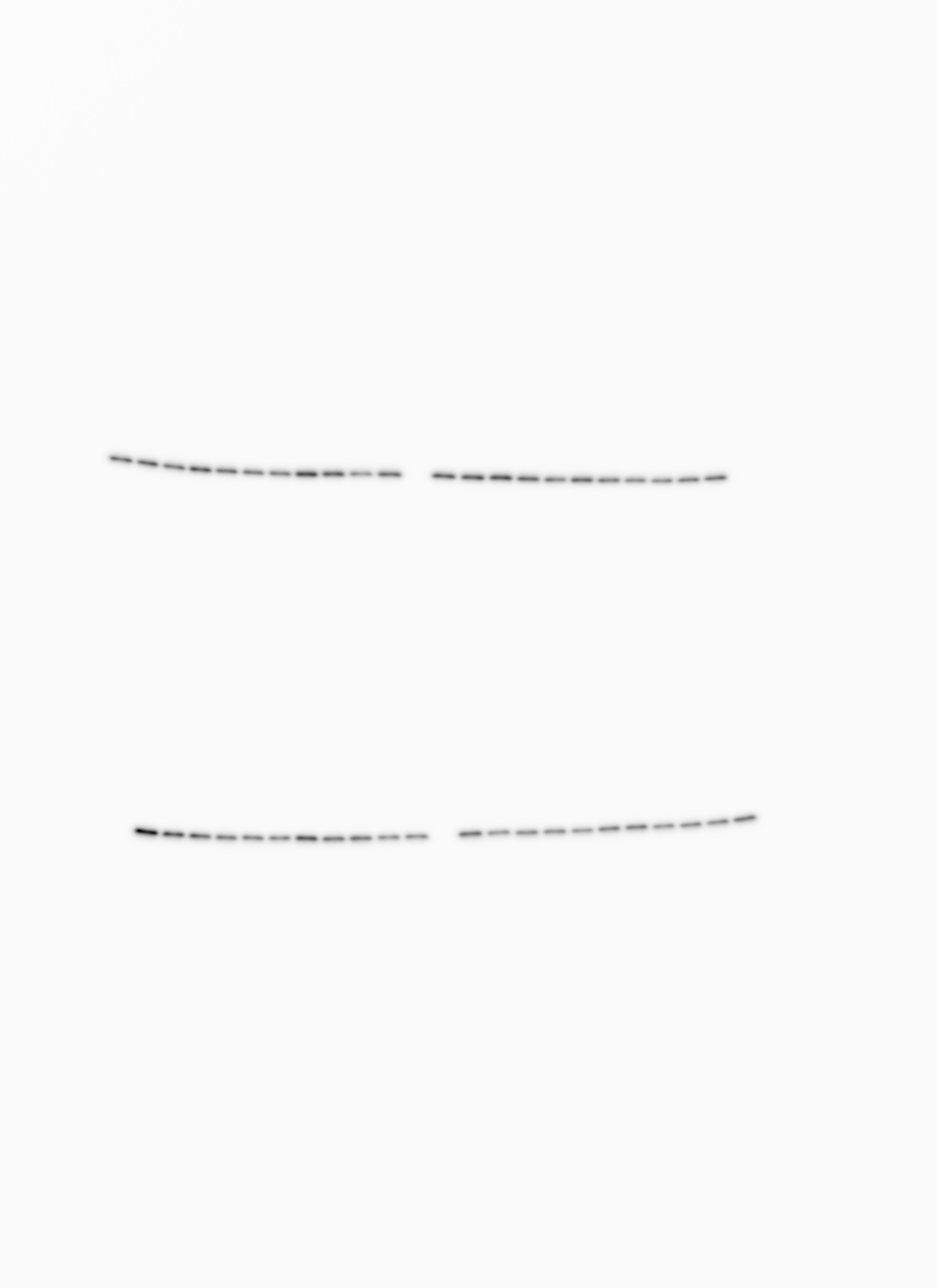

Supplement: Figure 2—figure supplement 1—source data 1. [file elife-81573-fig2-figsupp1-data1.zip › Figure 2-supplement 1-source data 1/Figure 2-supplement 1-source data 1_raw files/ws2 sun2gfp gapdh 2022.09.15_14.50.23-04_Ch/ws2 p+tran btr gapdh 2022.09.15_14.50.23-04_Ch.tif]

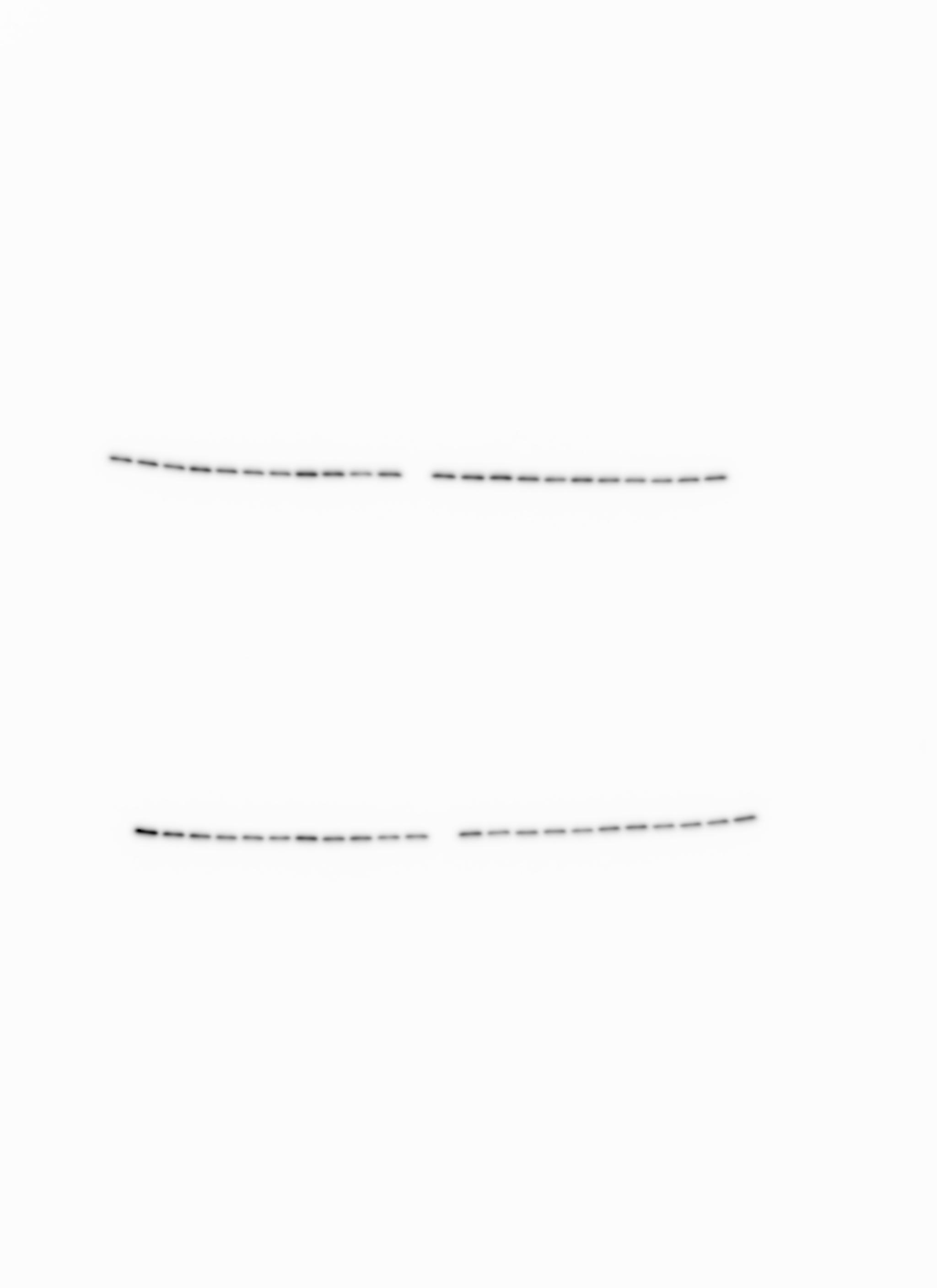

Supplement: Figure 2—figure supplement 1—source data 1. [file elife-81573-fig2-figsupp1-data1.zip › Figure 2-supplement 1-source data 1/Figure 2-supplement 1-source data 1_raw files/ws2 sun2gfp gapdh 2022.09.15_14.50.23-04_Ch/ws2 p+tran btr gapdh 2022.09.15_14.50.23-04_Ch.jpg]

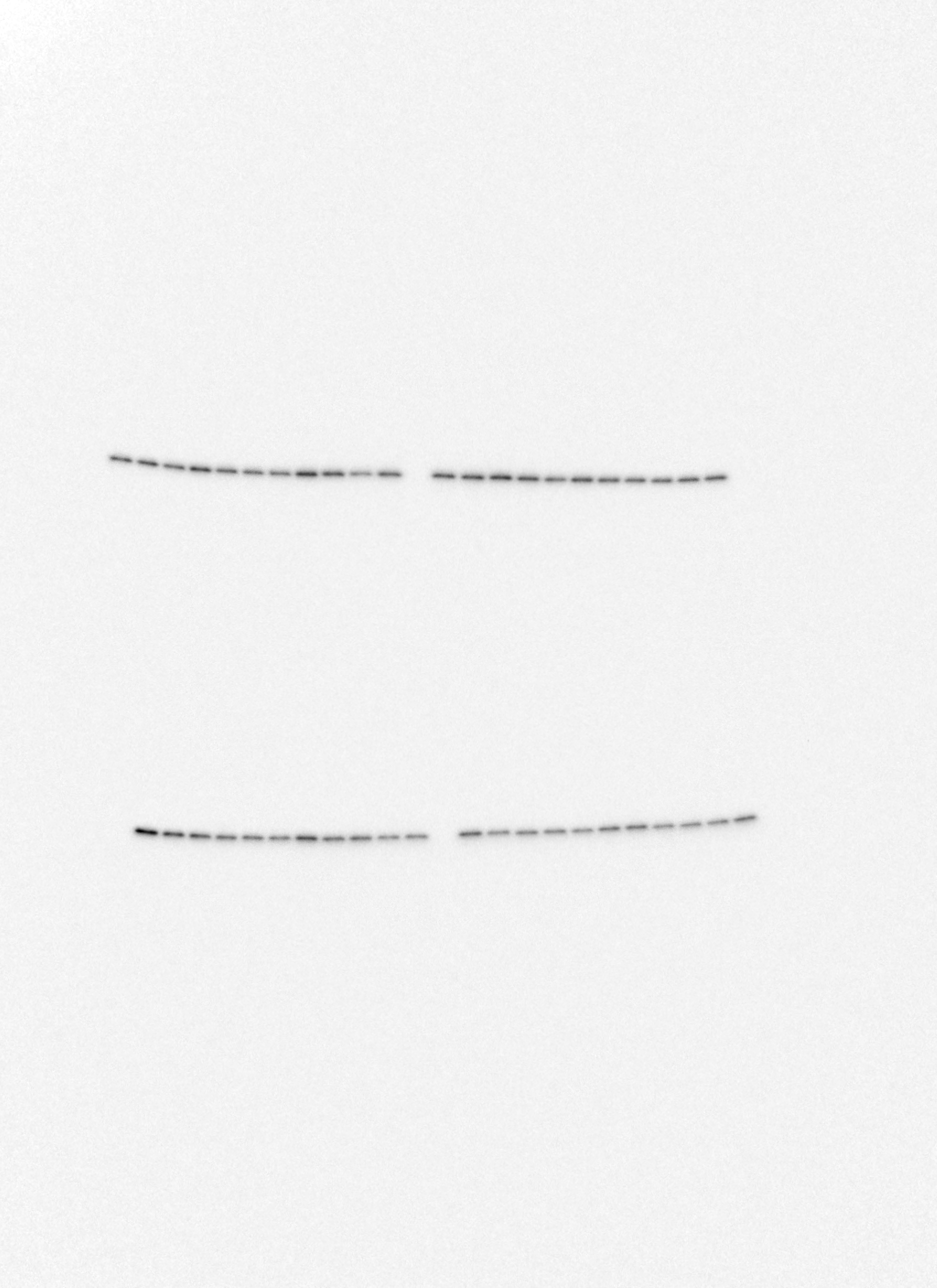

Supplement: Figure 2—figure supplement 1—source data 1. [file elife-81573-fig2-figsupp1-data1.zip › Figure 2-supplement 1-source data 1/Figure 2-supplement 1-source data 1_raw files/ws2 sun2gfp gapdh 2022.09.15_14.44.34_Ch/ws2 p+tran btr gapdh 2022.09.15_14.44.34_Ch.tif]

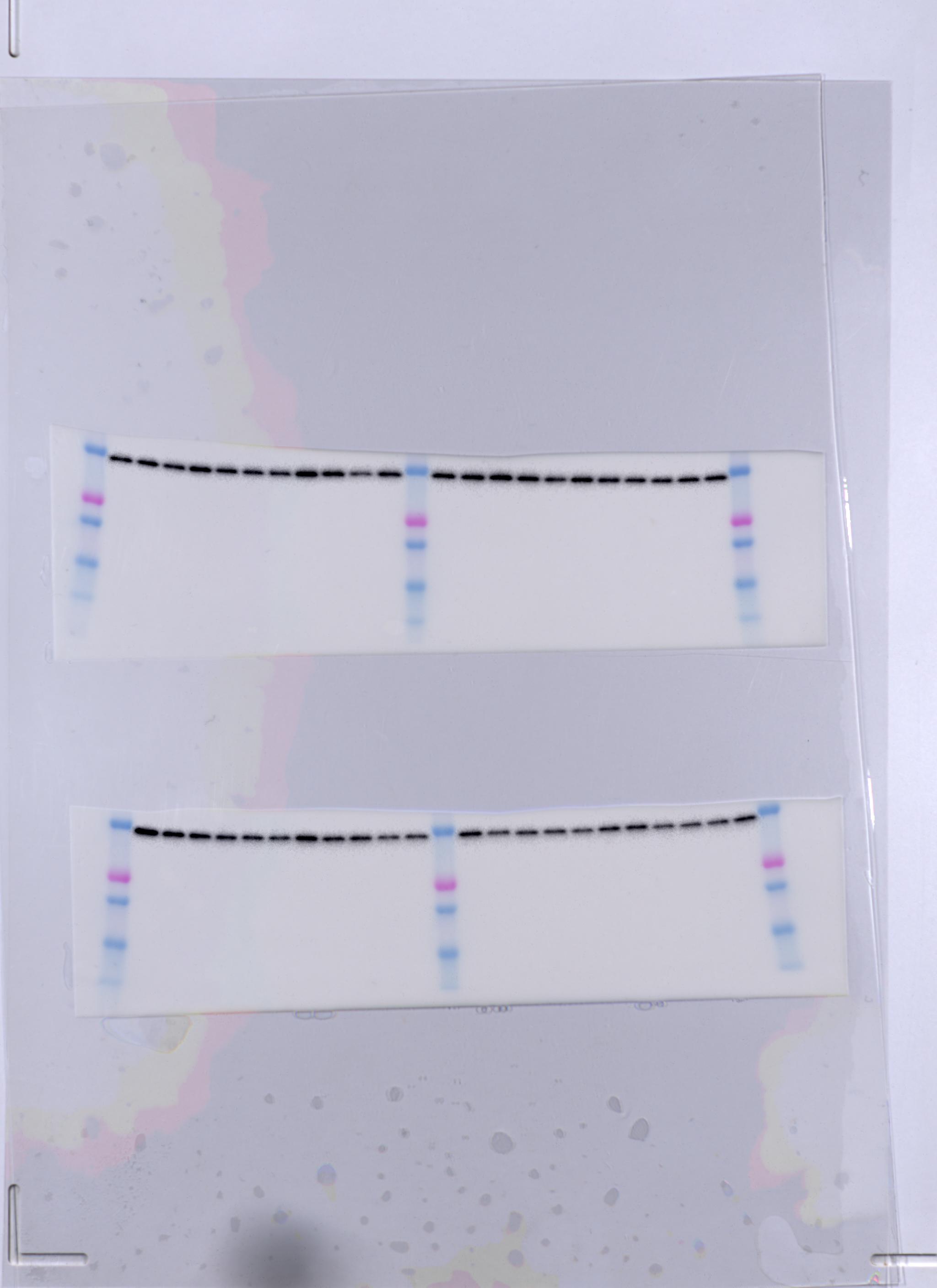

Supplement: Figure 2—figure supplement 1—source data 1. [file elife-81573-fig2-figsupp1-data1.zip › Figure 2-supplement 1-source data 1/Figure 2-supplement 1-source data 1_raw files/ws2 sun2gfp gapdh 2022.09.15_14.44.34_Ch/ws2 p+tran btr gapdh 2022.09.15_14.44.34_Ch+Marker.jpg]

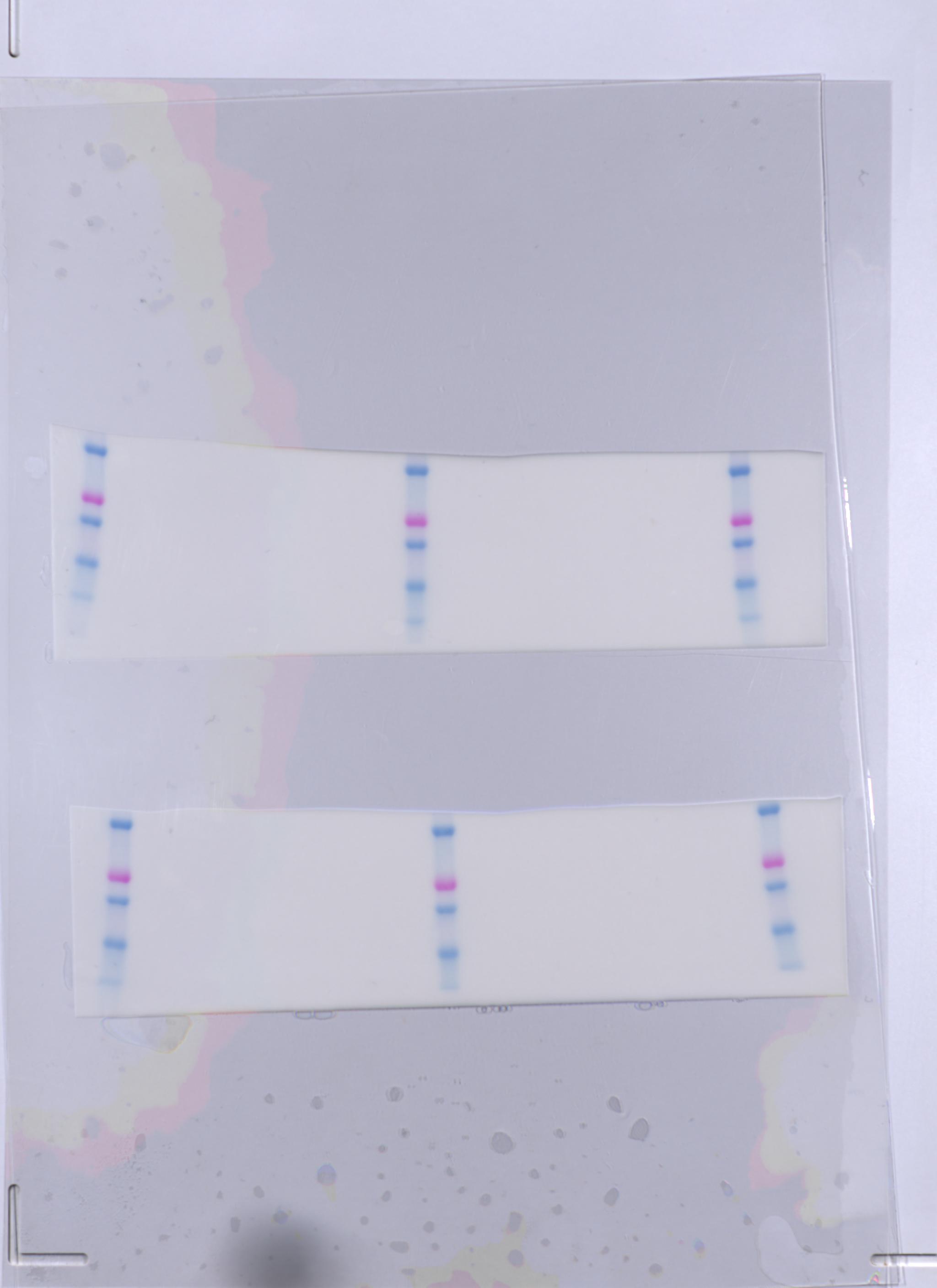

Supplement: Figure 2—figure supplement 1—source data 1. [file elife-81573-fig2-figsupp1-data1.zip › Figure 2-supplement 1-source data 1/Figure 2-supplement 1-source data 1_raw files/ws2 sun2gfp gapdh 2022.09.15_14.44.34_Ch/ws2 p+tran btr gapdh 2022.09.15_14.44.34_Ch-Marker.jpg]

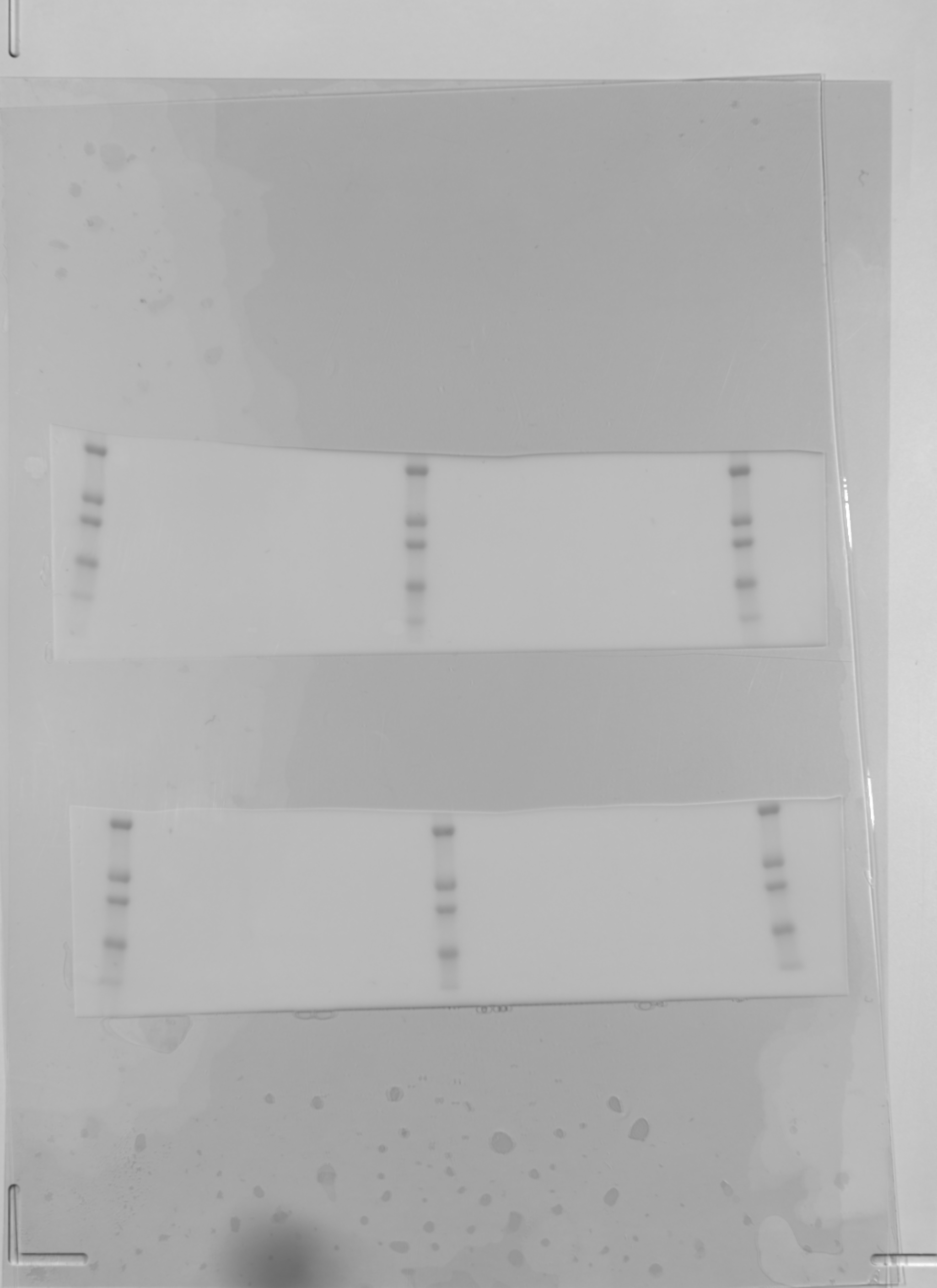

Supplement: Figure 2—figure supplement 1—source data 1. [file elife-81573-fig2-figsupp1-data1.zip › Figure 2-supplement 1-source data 1/Figure 2-supplement 1-source data 1_raw files/ws2 sun2gfp gapdh 2022.09.15_14.44.34_Ch/ws2 p+tran btr gapdh 2022.09.15_14.44.34_Ch-Marker.tif]

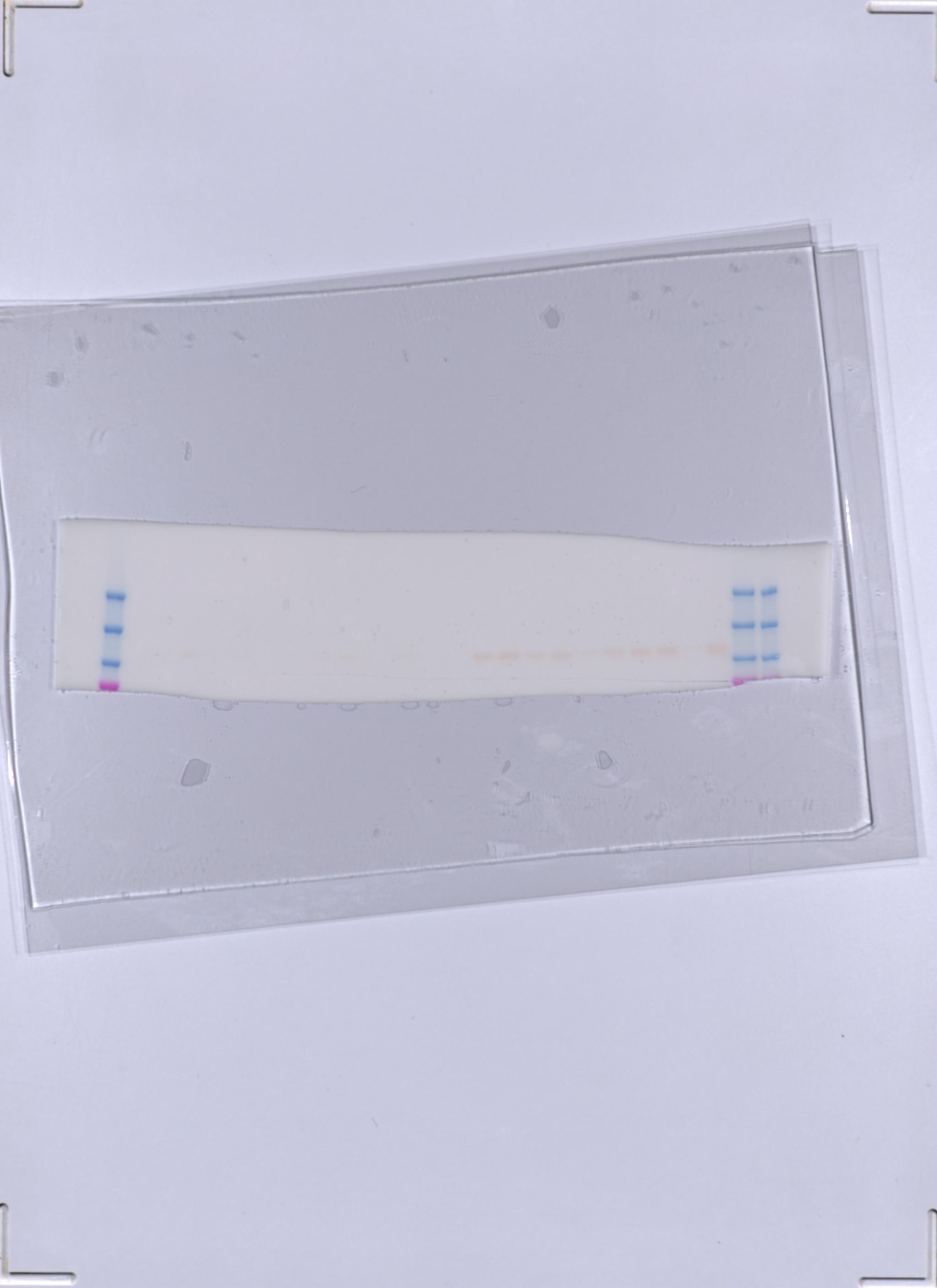

Supplement: Figure 3—source data 1. [file elife-81573-fig3-data1.zip › Figure 3-source data 1/Figure 3-source data 1_raw files/ws CoIP2 HA 2022.05.10_19.04.36_Ch-Marker.jpg]

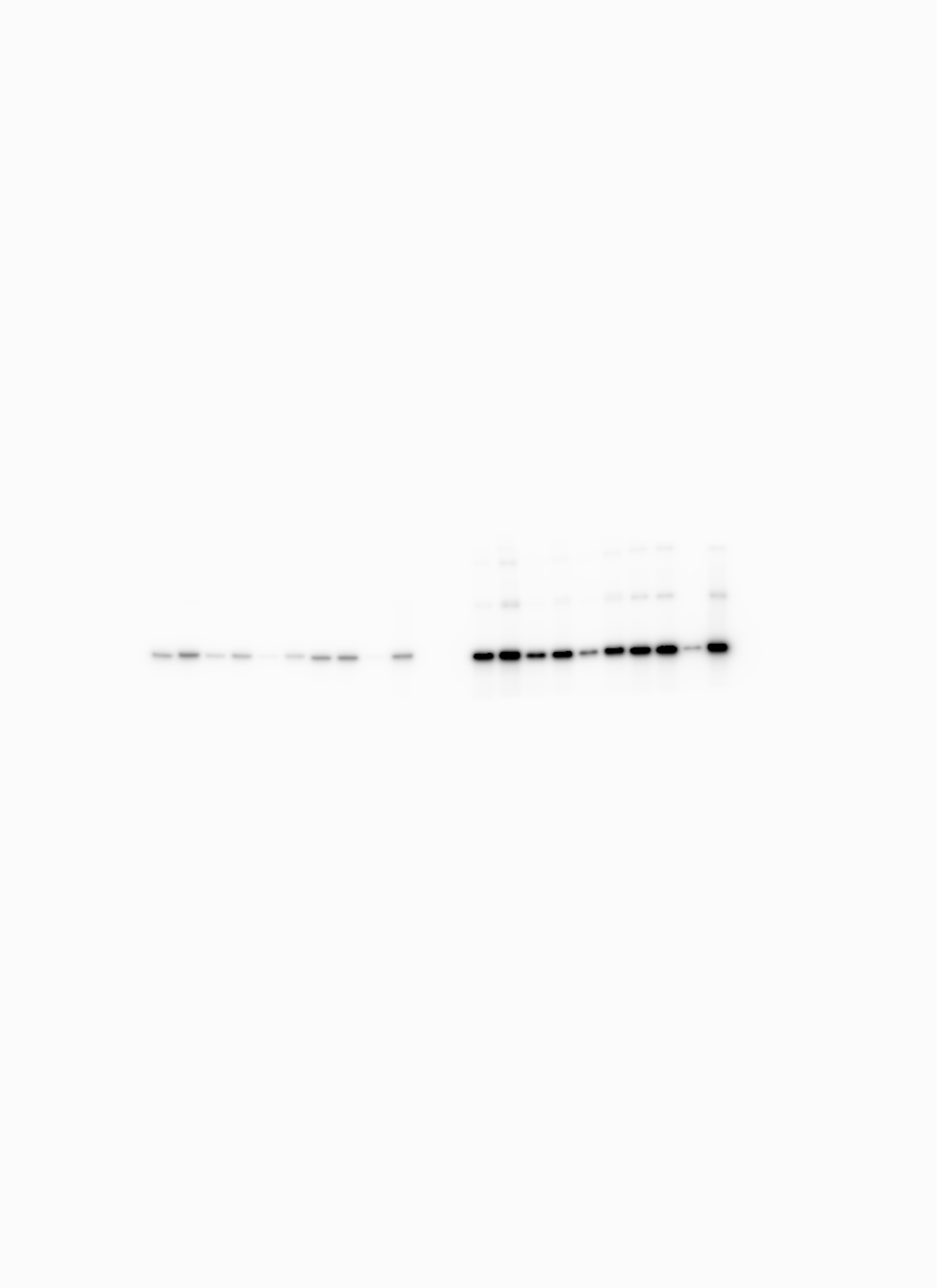

Supplement: Figure 3—source data 1. [file elife-81573-fig3-data1.zip › Figure 3-source data 1/Figure 3-source data 1_raw files/ws CoIP2 HA 2022.05.10_18.52.40-01_Ch.tif]

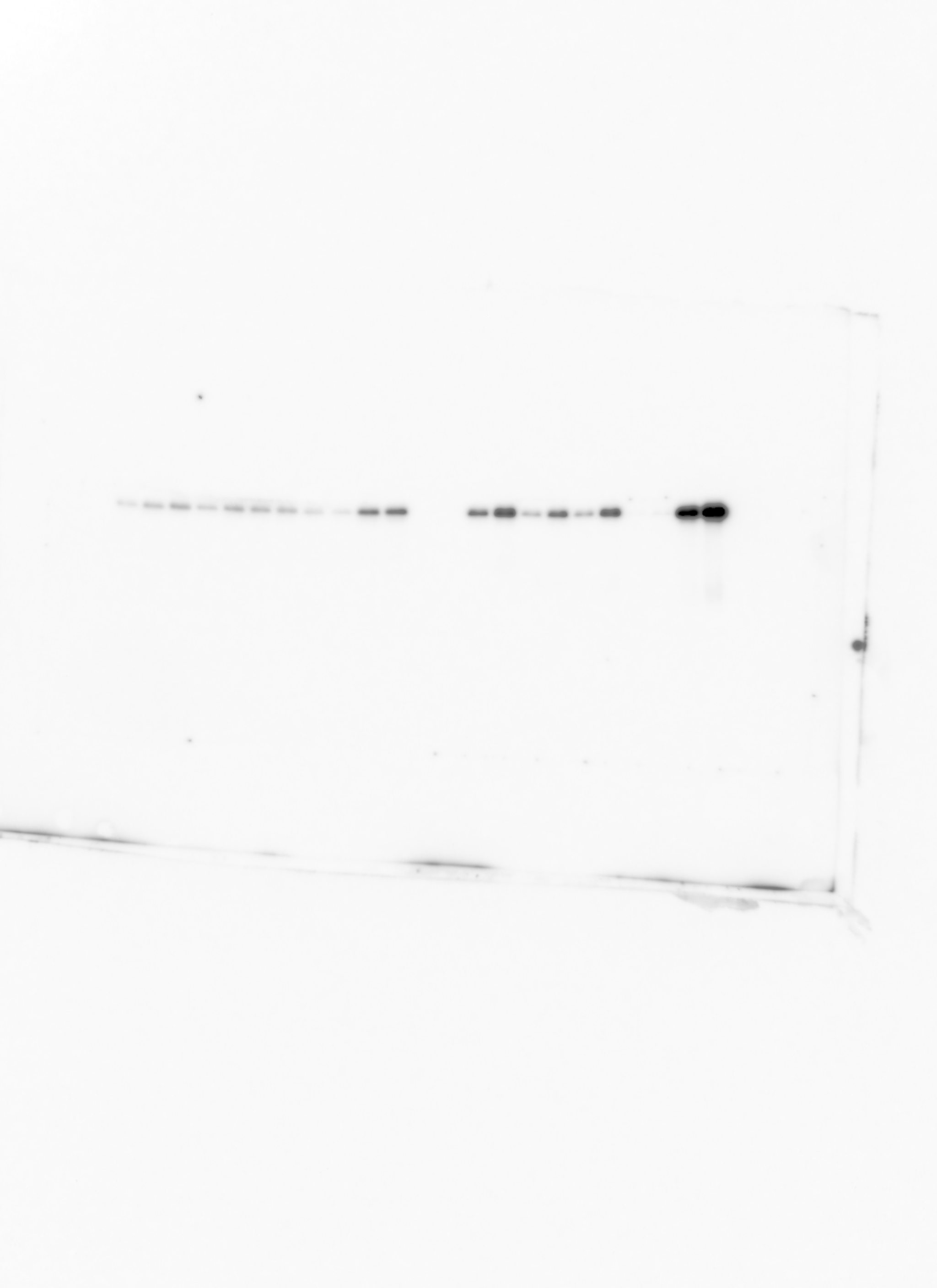

Supplement: Figure 3—source data 1. [file elife-81573-fig3-data1.zip › Figure 3-source data 1/Figure 3-source data 1_raw files/ws CoIP2 bTrCP 2022.05.10_20.50.26-12_Ch.tif]

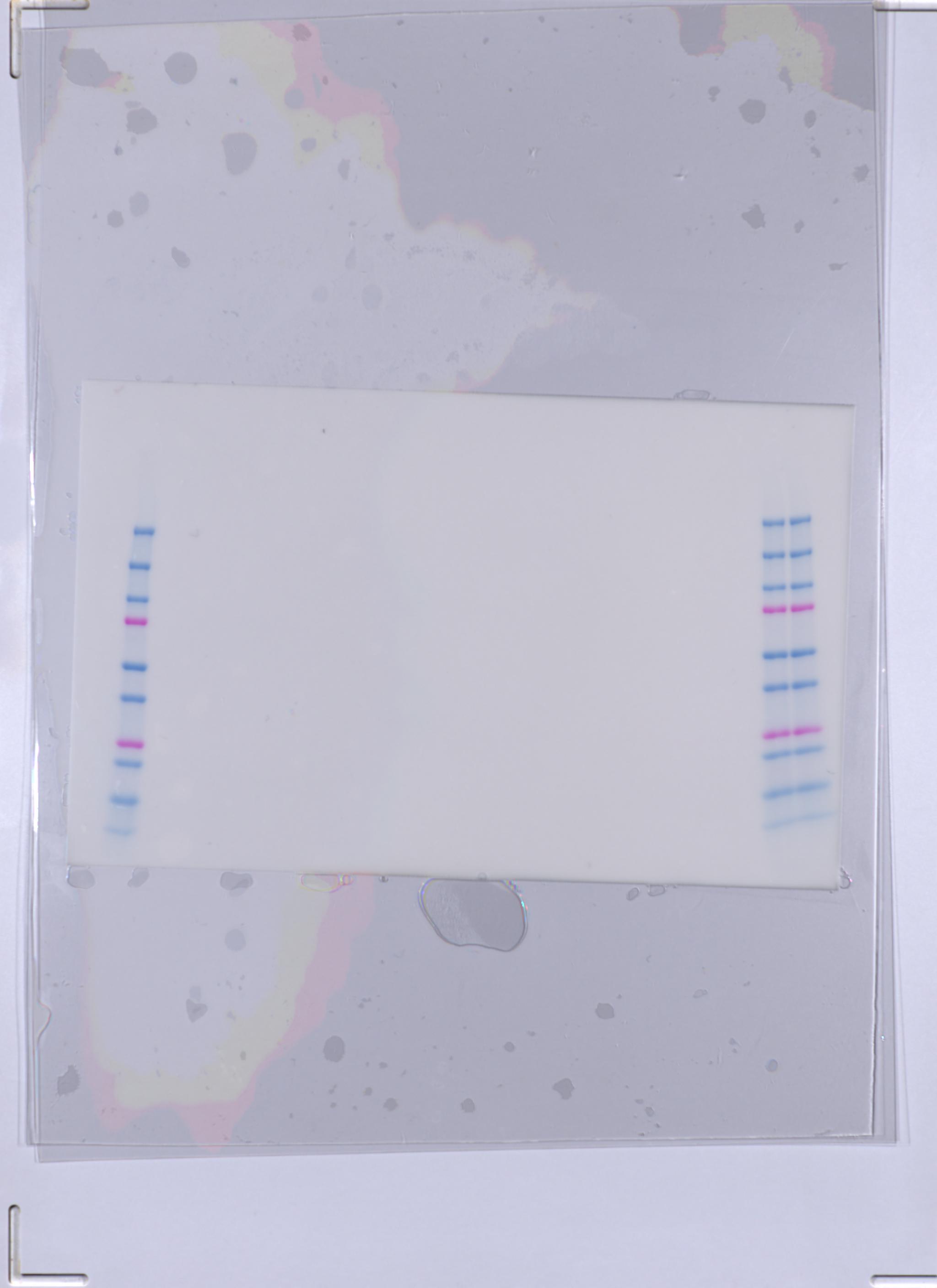

Supplement: Figure 3—source data 1. [file elife-81573-fig3-data1.zip › Figure 3-source data 1/Figure 3-source data 1_raw files/ws CoIP2 Cullin1 2022.05.10_21.43.21_Ch-Marker.jpg]

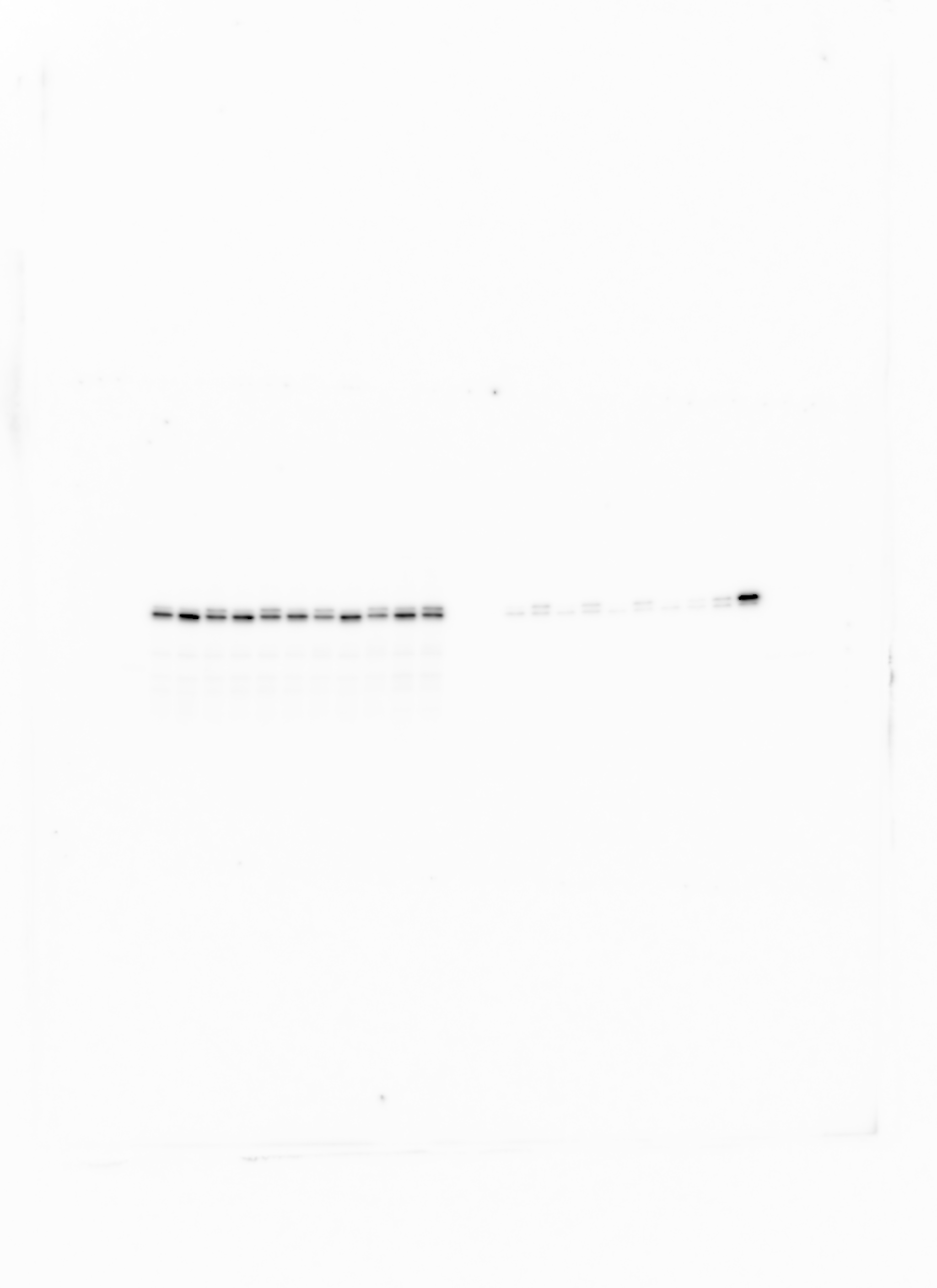

Supplement: Figure 3—source data 1. [file elife-81573-fig3-data1.zip › Figure 3-source data 1/Figure 3-source data 1_raw files/ws CoIP2 Cullin1 lon 2022.05.10_22.11.02-12_Ch.tif]

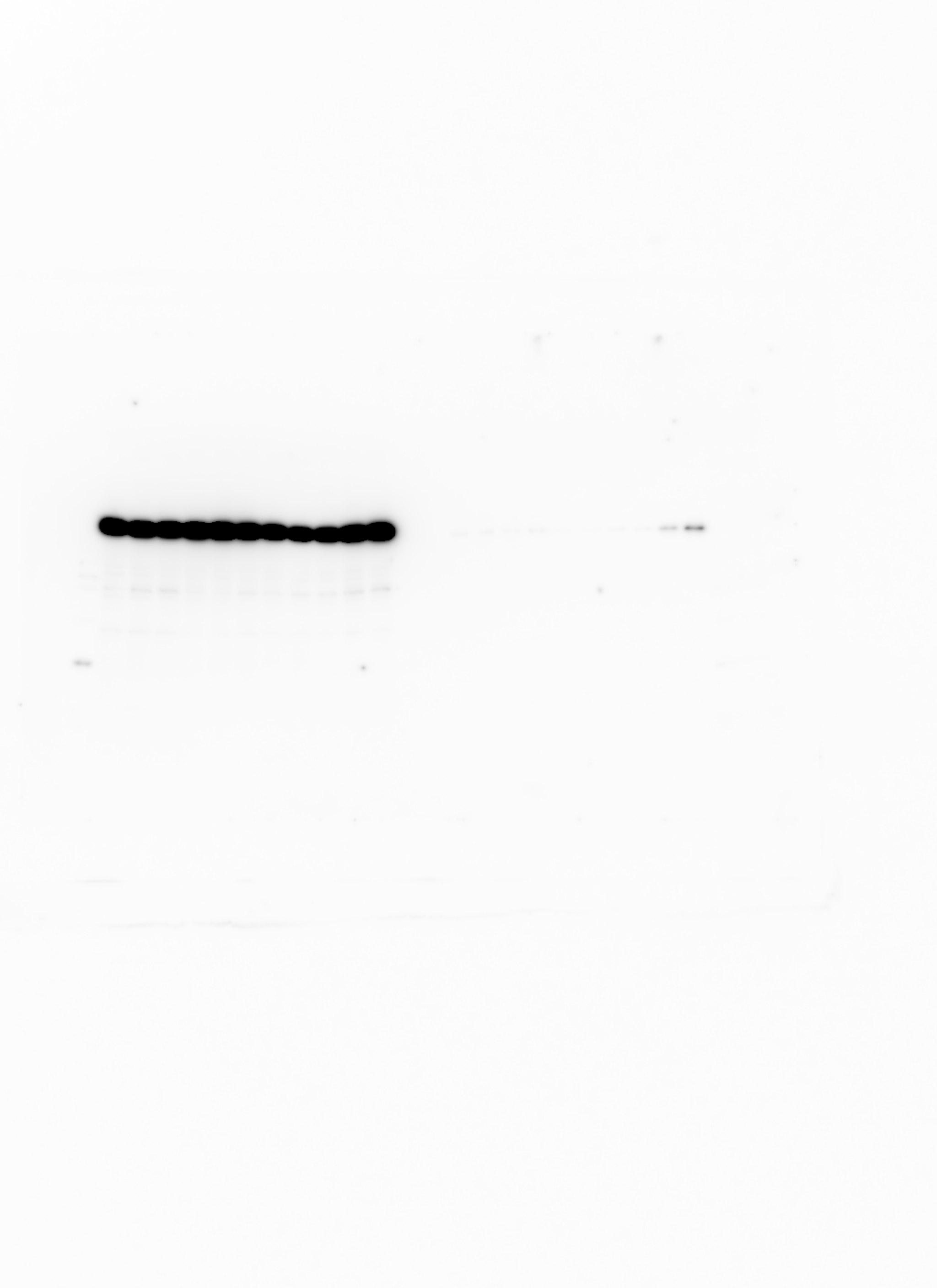

Supplement: Figure 3—source data 1. [file elife-81573-fig3-data1.zip › Figure 3-source data 1/Figure 3-source data 1_raw files/ws CoIP2 p97 2022.05.10_19.30.24-12_Ch.tif]

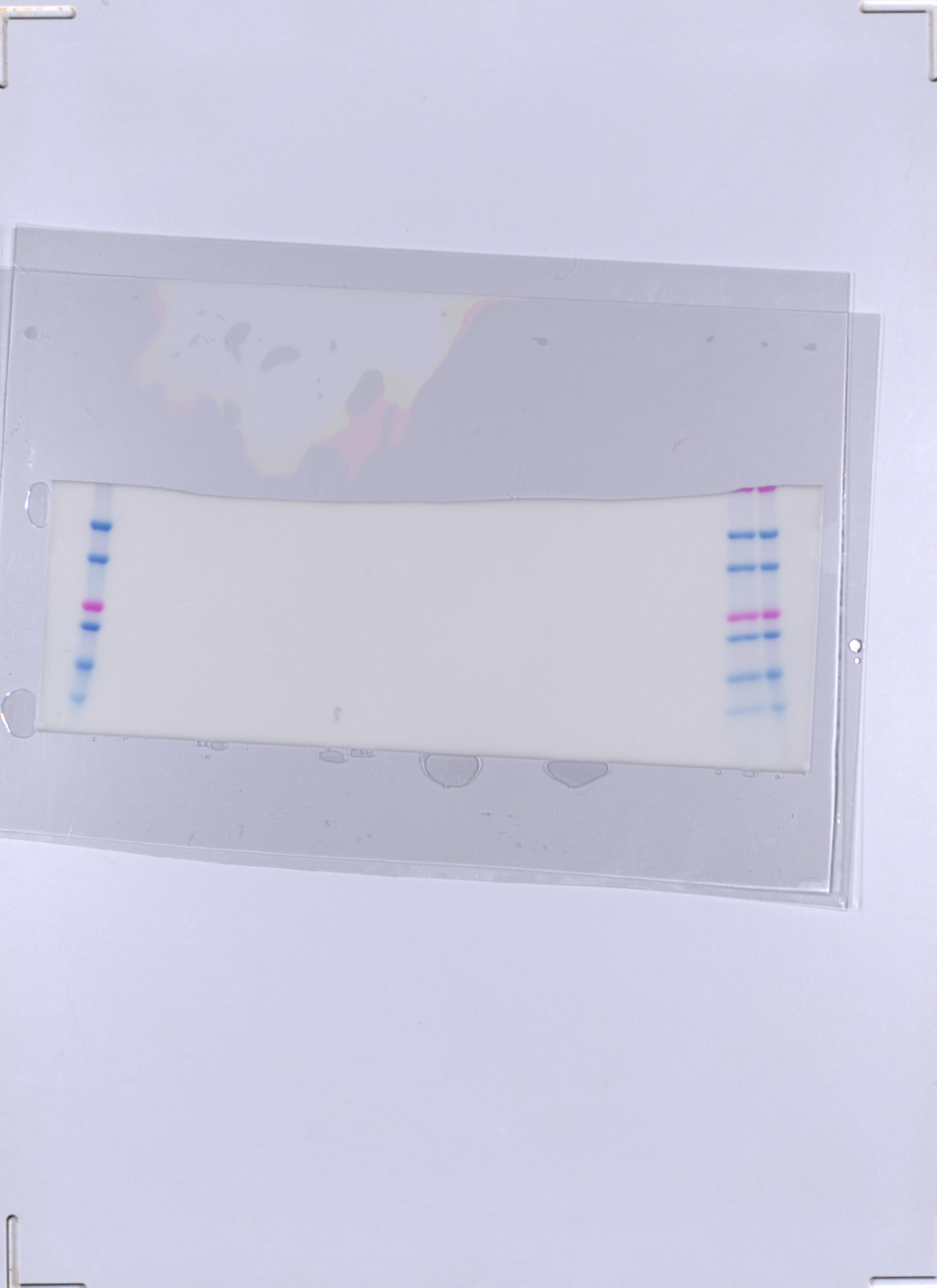

Supplement: Figure 3—source data 1. [file elife-81573-fig3-data1.zip › Figure 3-source data 1/Figure 3-source data 1_raw files/ws CoIP2 bTrCP 2022.05.10_20.22.05_Ch-Marker.jpg]

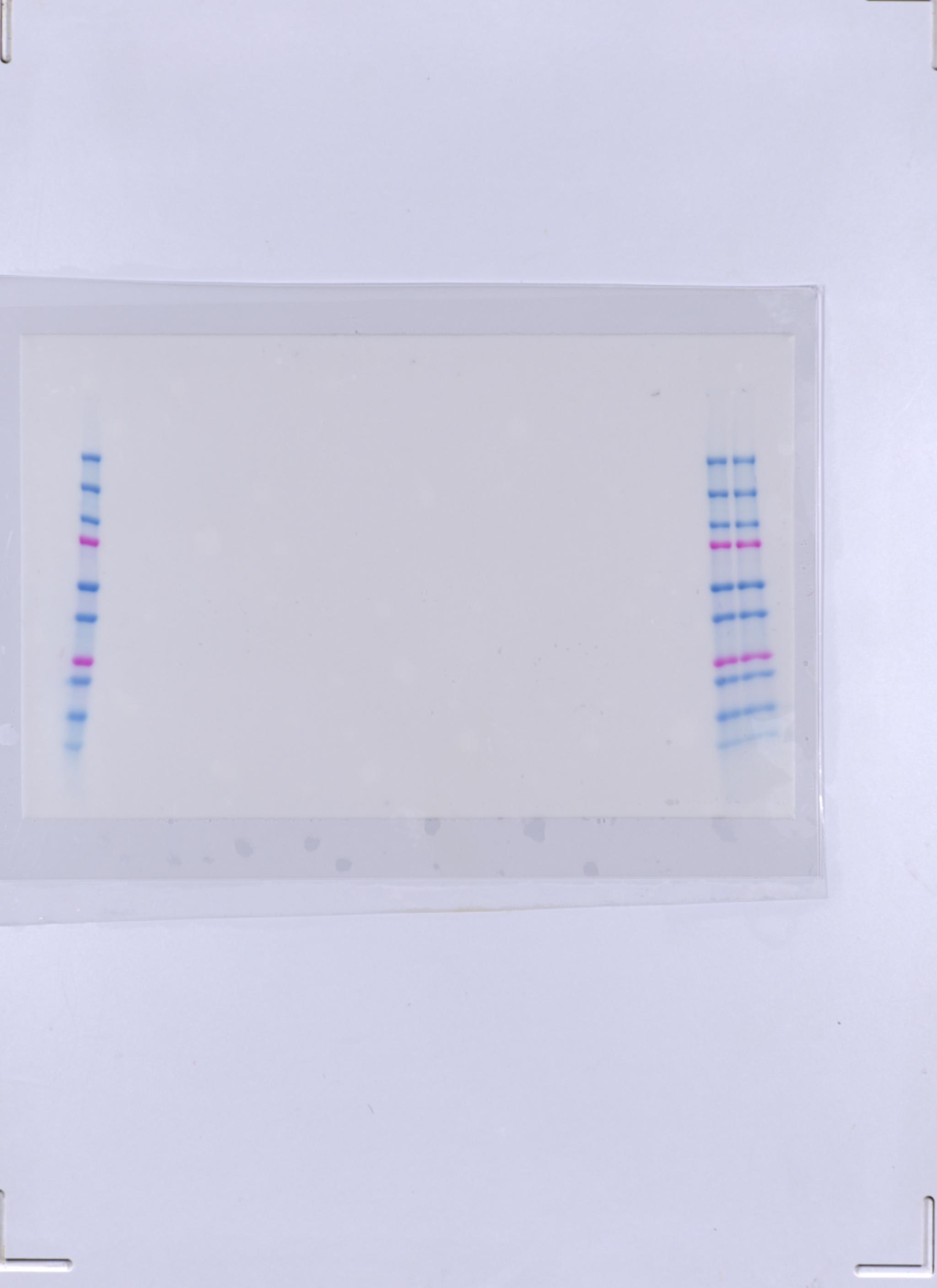

Supplement: Figure 3—source data 1. [file elife-81573-fig3-data1.zip › Figure 3-source data 1/Figure 3-source data 1_raw files/ws CoIP2 p97 2022.05.10_20.10.12_Ch-Marker.jpg]

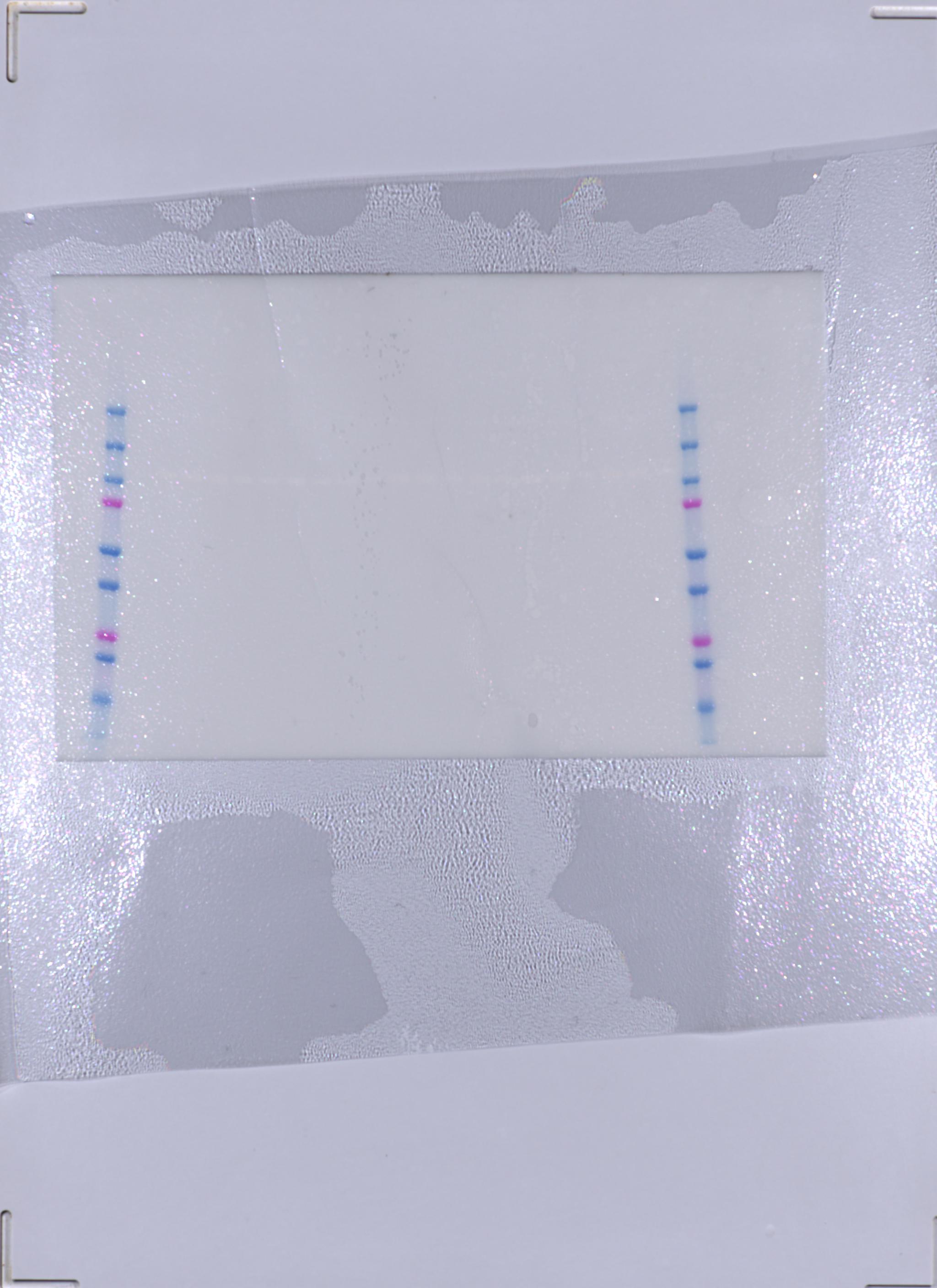

Supplement: Figure 3—source data 2. [file elife-81573-fig3-data2.zip › Figure 3-source data 2/Figure 3-source data 2_raw files/ws deIP HA 2022.05.11_16.33.30_Ch-Marker.jpg]

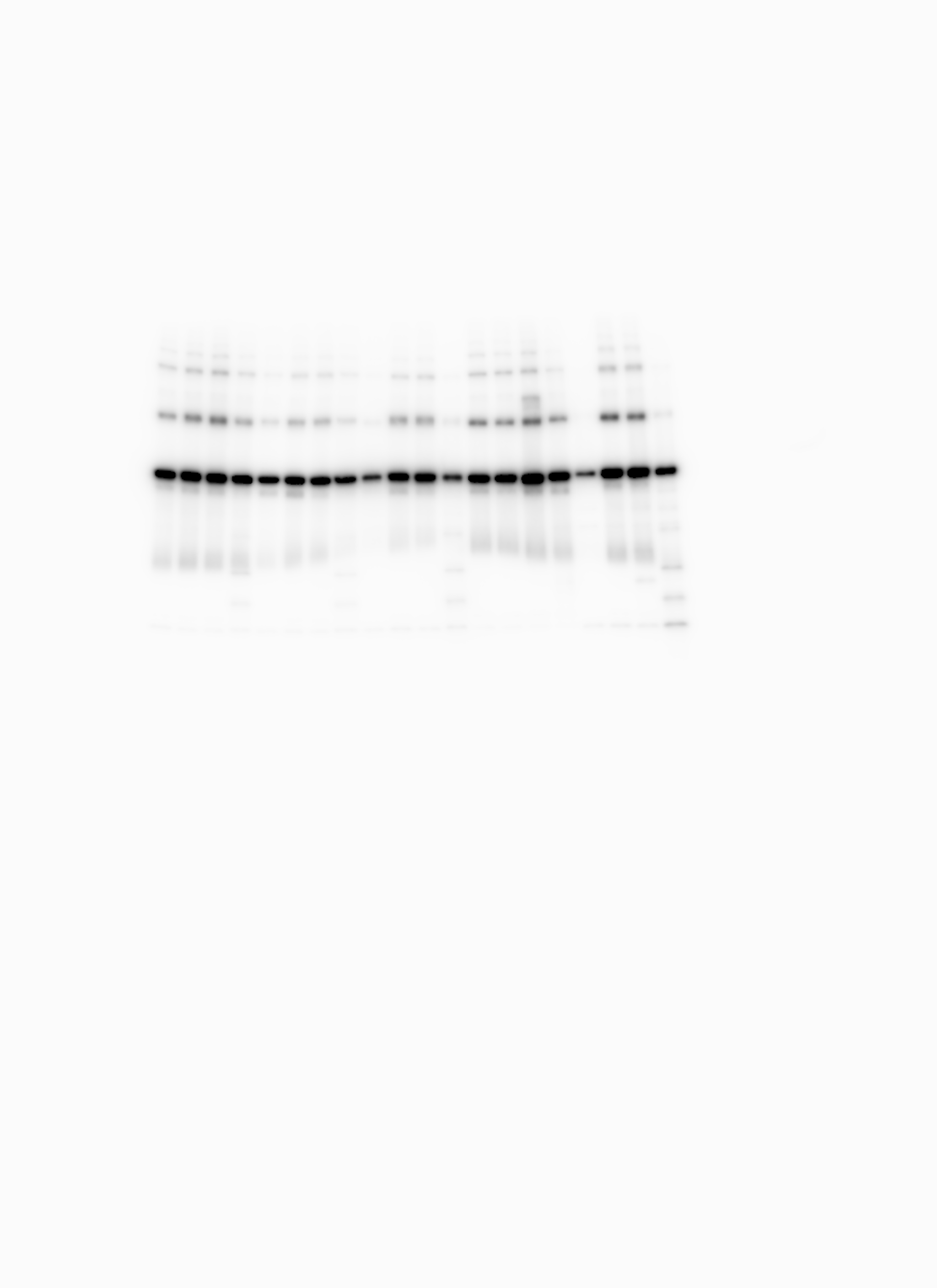

Supplement: Figure 3—source data 2. [file elife-81573-fig3-data2.zip › Figure 3-source data 2/Figure 3-source data 2_raw files/ws deIP HA 2022.05.11_16.28.32-01_Ch.tif]

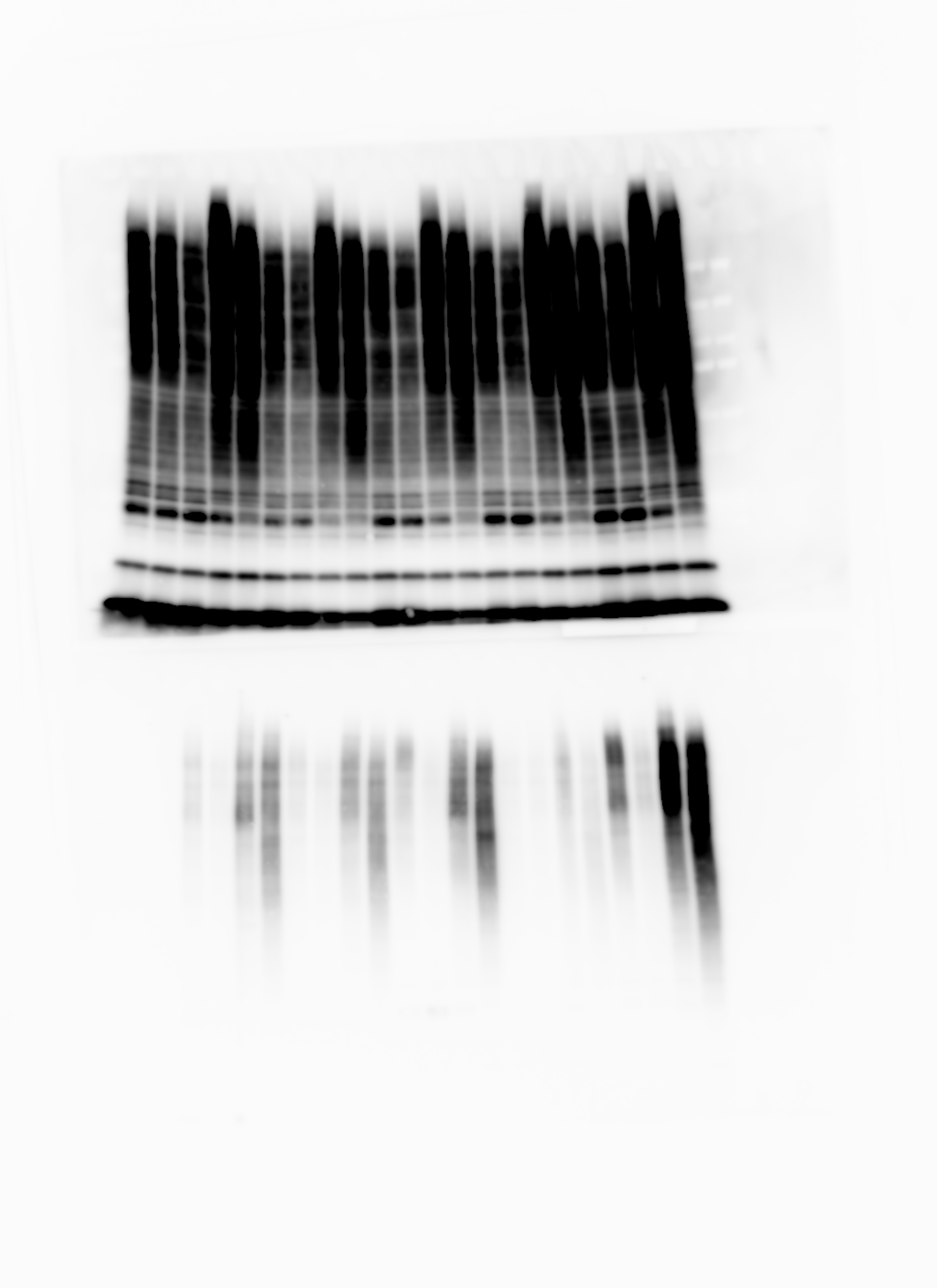

Supplement: Figure 3—source data 2. [file elife-81573-fig3-data2.zip › Figure 3-source data 2/Figure 3-source data 2_raw files/ws deIP ubiquitin 2022.05.11_16.54.22-06_Ch.tif]

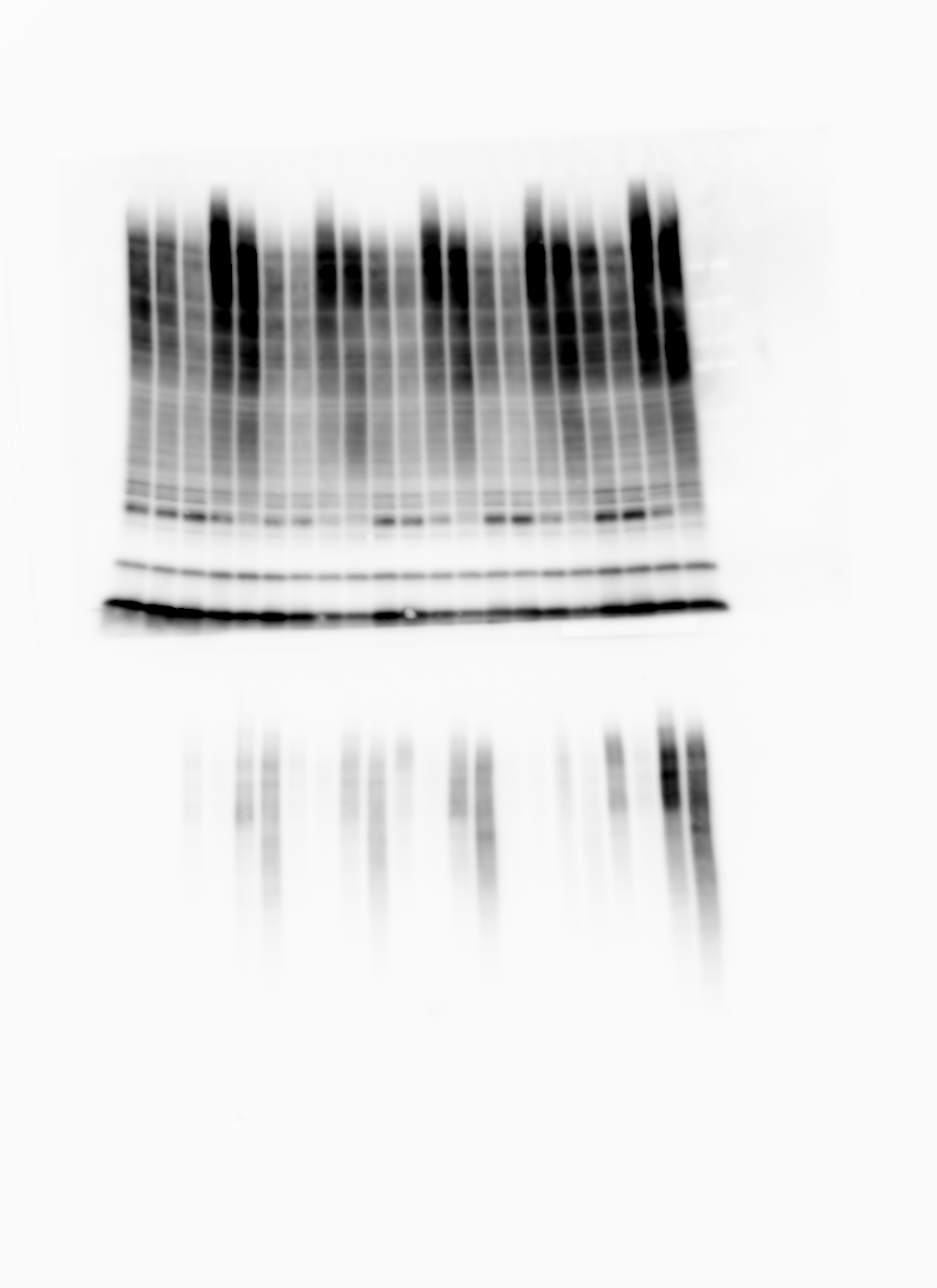

Supplement: Figure 3—source data 2. [file elife-81573-fig3-data2.zip › Figure 3-source data 2/Figure 3-source data 2_raw files/ws deIP ubiquitin 2022.05.11_16.54.22-03_Ch.tif]

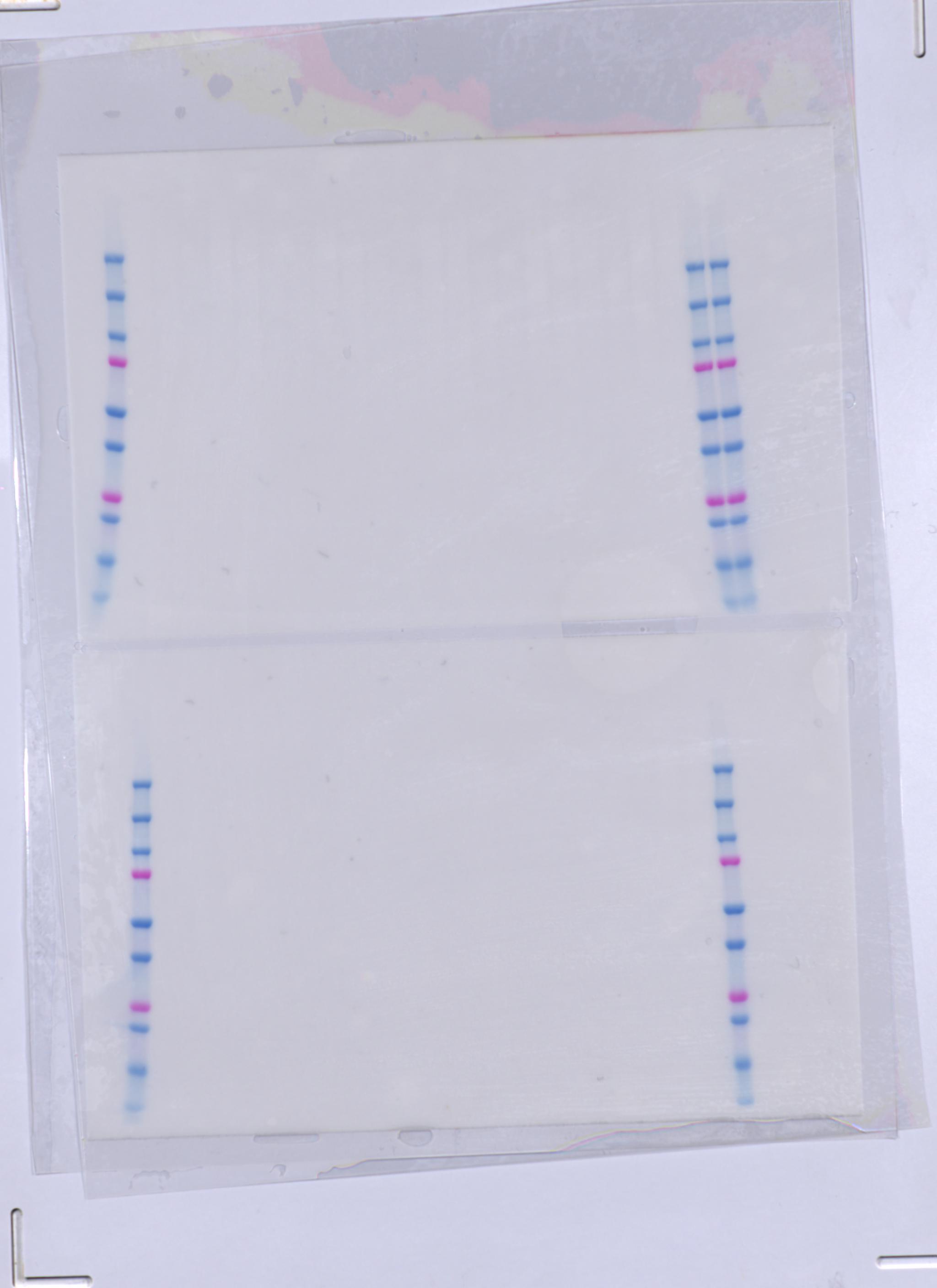

Supplement: Figure 3—source data 2. [file elife-81573-fig3-data2.zip › Figure 3-source data 2/Figure 3-source data 2_raw files/ws deIP ubiquitin 2022.05.11_16.52.42_Ch-Marker.jpg]

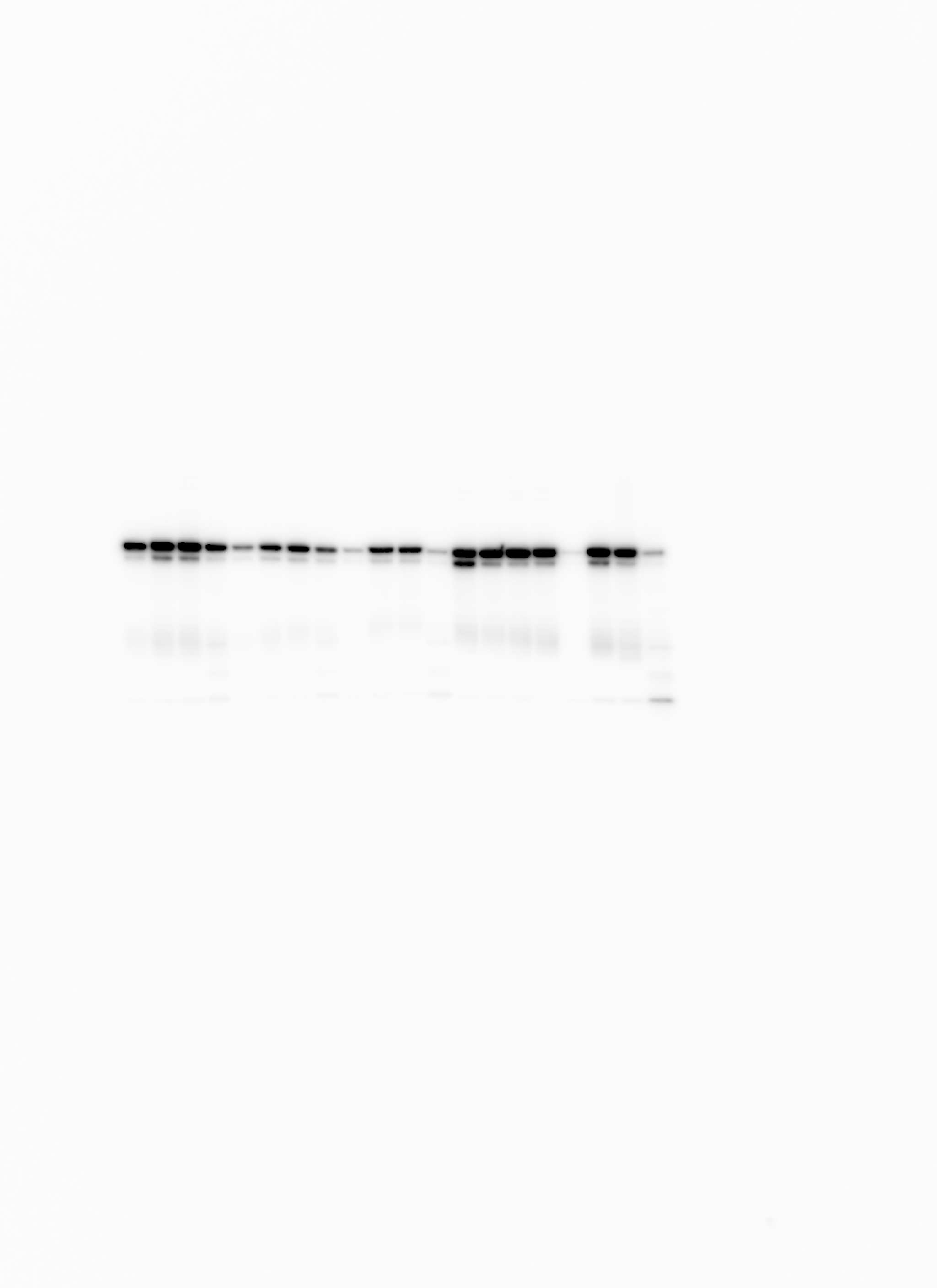

Supplement: Figure 3—source data 2. [file elife-81573-fig3-data2.zip › Figure 3-source data 2/Figure 3-source data 2_raw files/ws deIP HA 2022.05.11_16.40.55-05_Ch.tif]

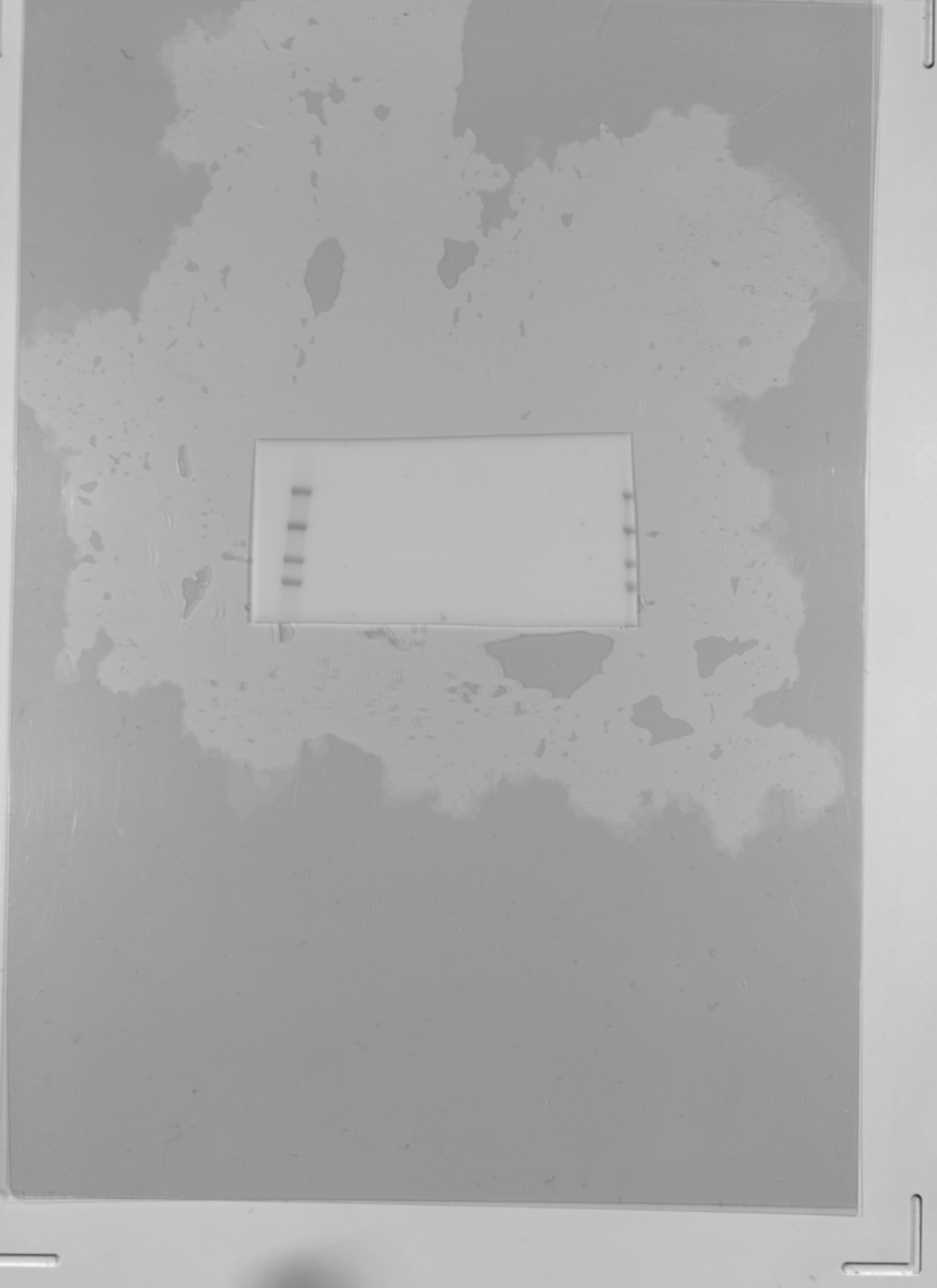

Supplement: Figure 4—source data 1. [file elife-81573-fig4-data1.zip › Figure 4-source data 1/Figure 4-source data 1_raw files/LK220708 Fig4C HA 2022.07.08_18.48.58_Ch-Marker.tif]

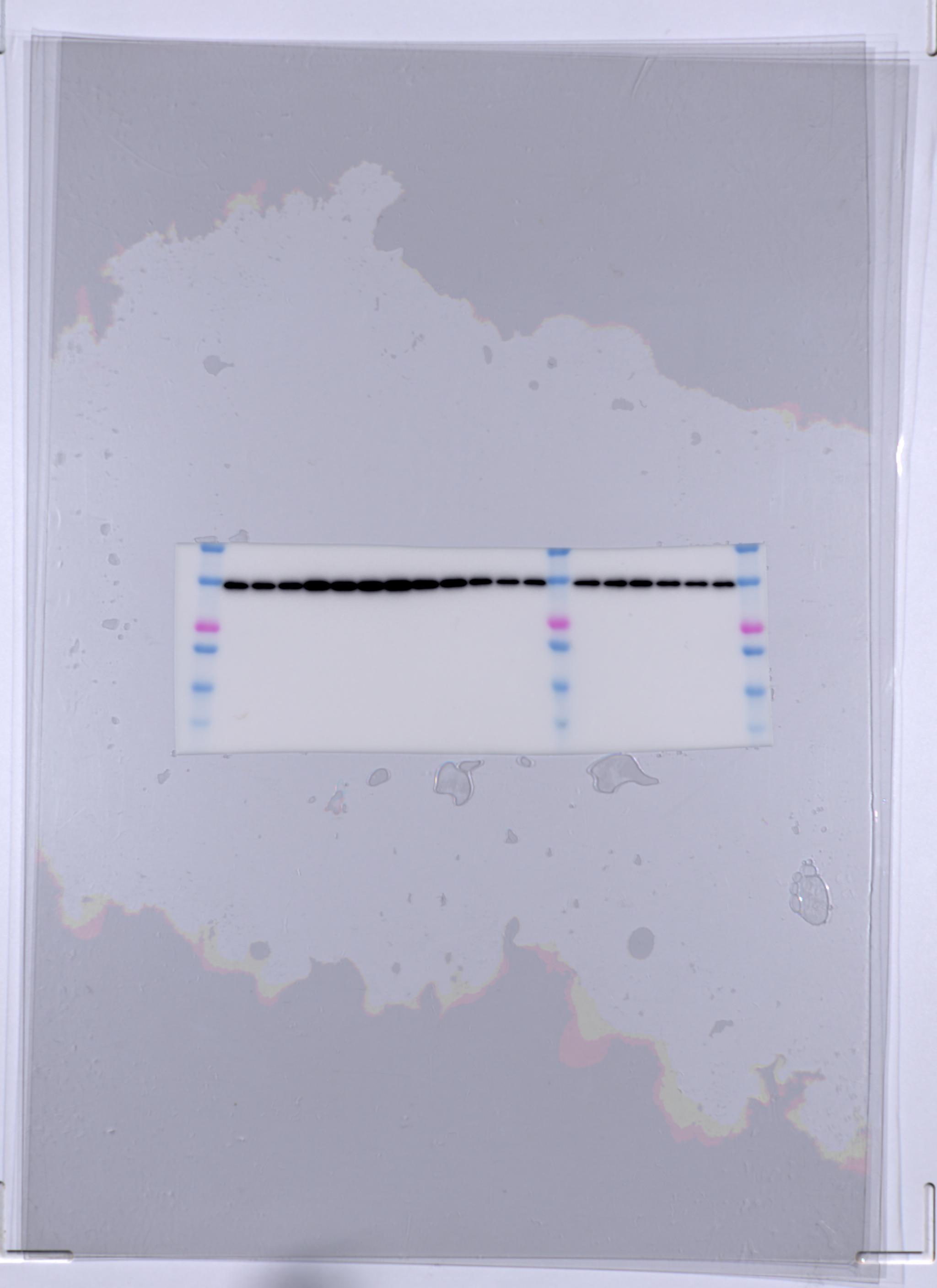

Supplement: Figure 4—source data 1. [file elife-81573-fig4-data1.zip › Figure 4-source data 1/Figure 4-source data 1_raw files/LK220708 Fig4C Gpdh 2022.07.08_20.42.33_Ch+Marker.jpg]

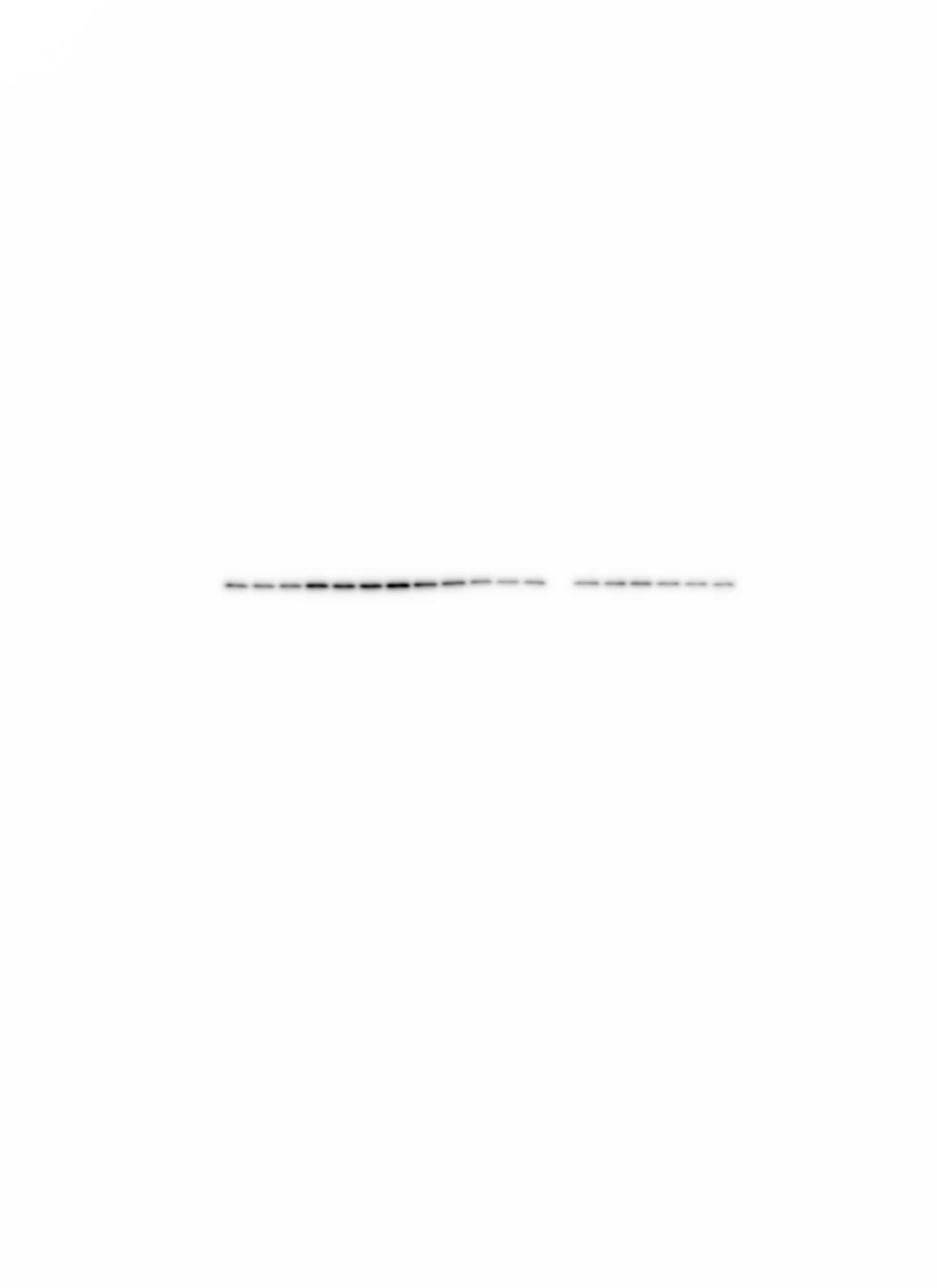

Supplement: Figure 4—source data 1. [file elife-81573-fig4-data1.zip › Figure 4-source data 1/Figure 4-source data 1_raw files/LK220708 Fig4C Gpdh 2022.07.08_20.45.33-03_Ch.tif]

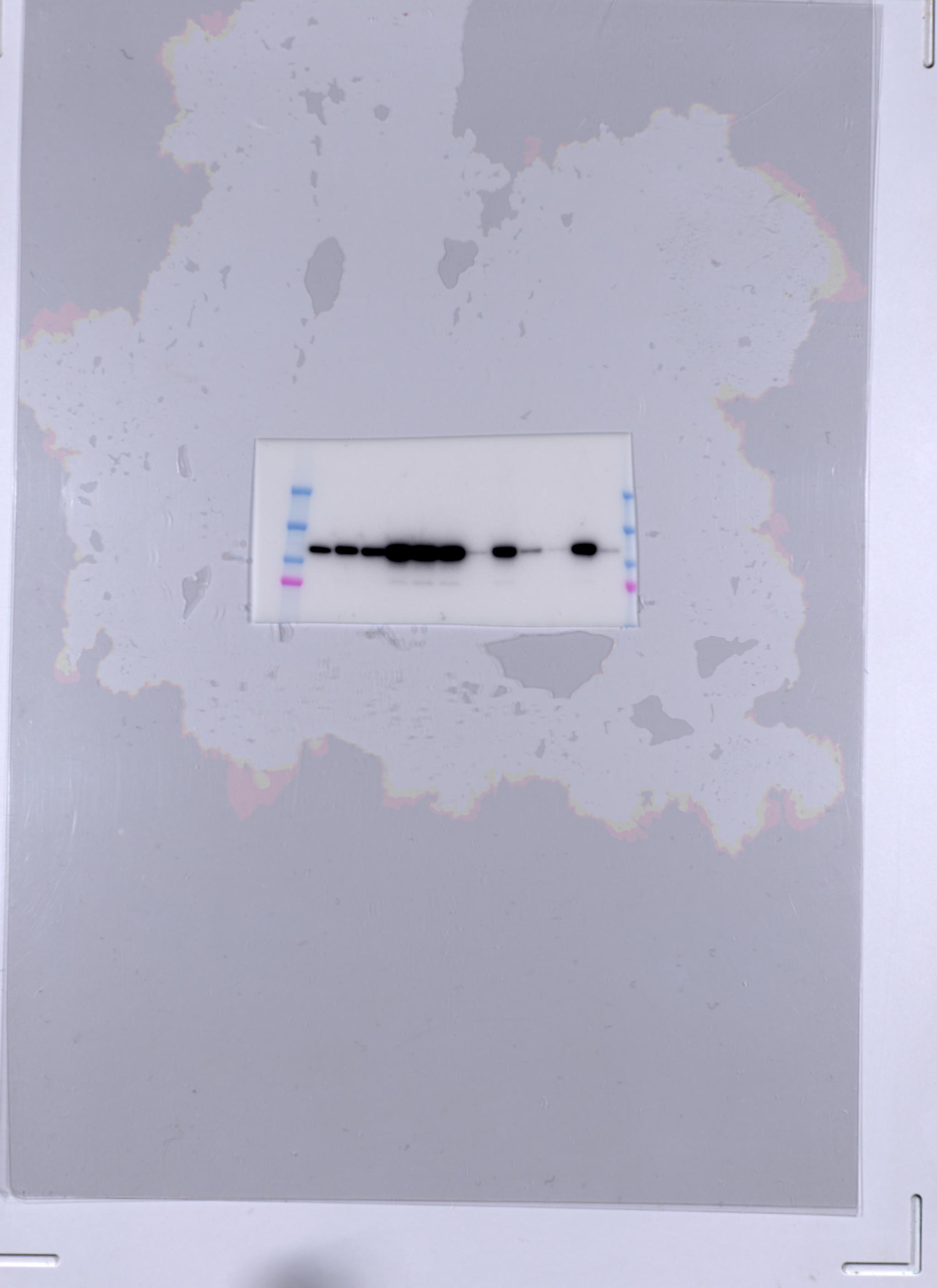

Supplement: Figure 4—source data 1. [file elife-81573-fig4-data1.zip › Figure 4-source data 1/Figure 4-source data 1_raw files/LK220708 Fig4C HA 2022.07.08_18.48.58_Ch+Marker.jpg]

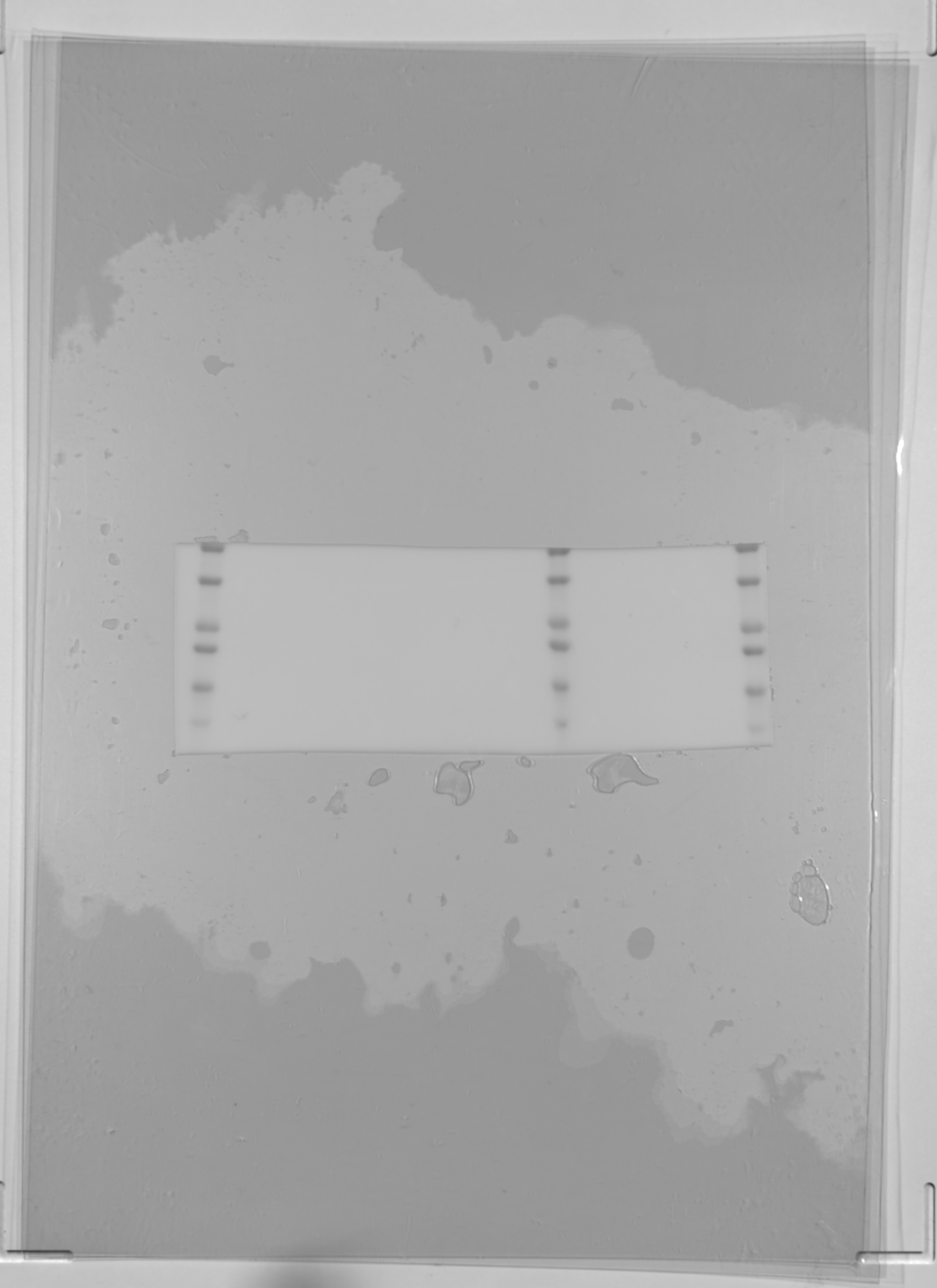

Supplement: Figure 4—source data 1. [file elife-81573-fig4-data1.zip › Figure 4-source data 1/Figure 4-source data 1_raw files/LK220708 Fig4C Gpdh 2022.07.08_20.42.33_Ch-Marker.tif]

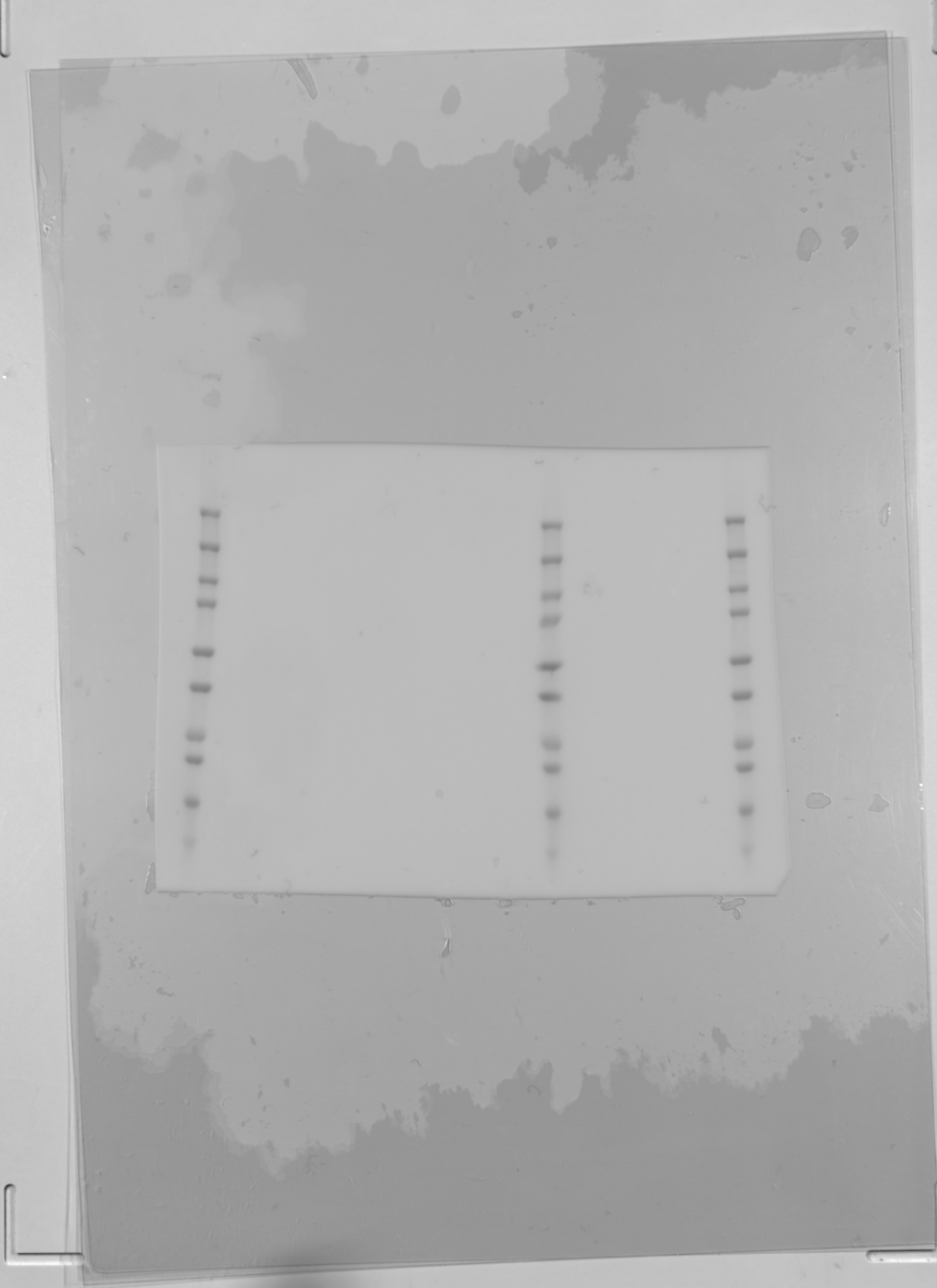

Supplement: Figure 4—source data 1. [file elife-81573-fig4-data1.zip › Figure 4-source data 1/Figure 4-source data 1_raw files/LK220708 Fig4C CK2Sb 2022.07.08_19.09.28_Ch-Marker.tif]

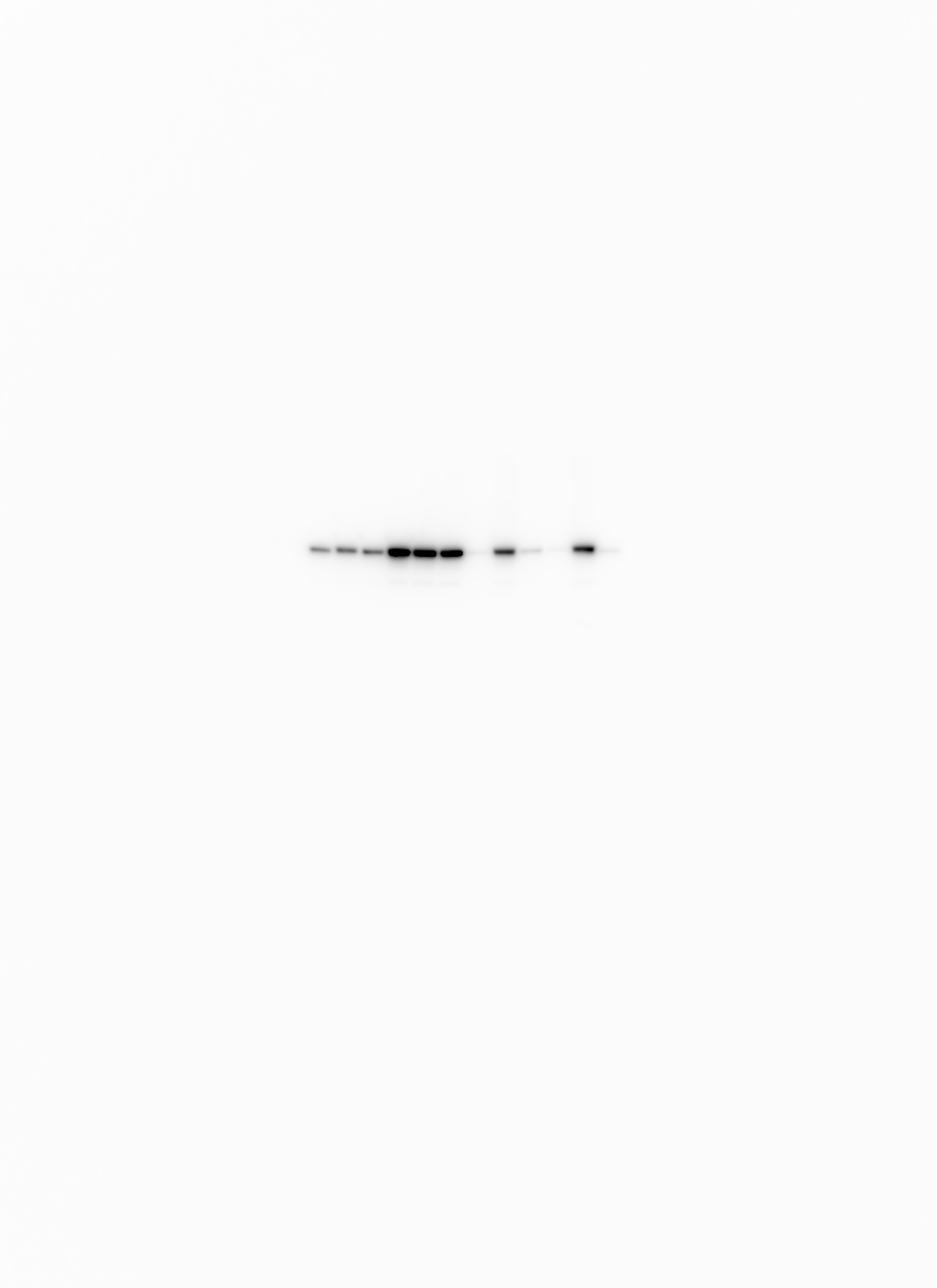

Supplement: Figure 4—source data 1. [file elife-81573-fig4-data1.zip › Figure 4-source data 1/Figure 4-source data 1_raw files/LK220708 Fig4C HA 2022.07.08_18.50.47-06_Ch.tif]

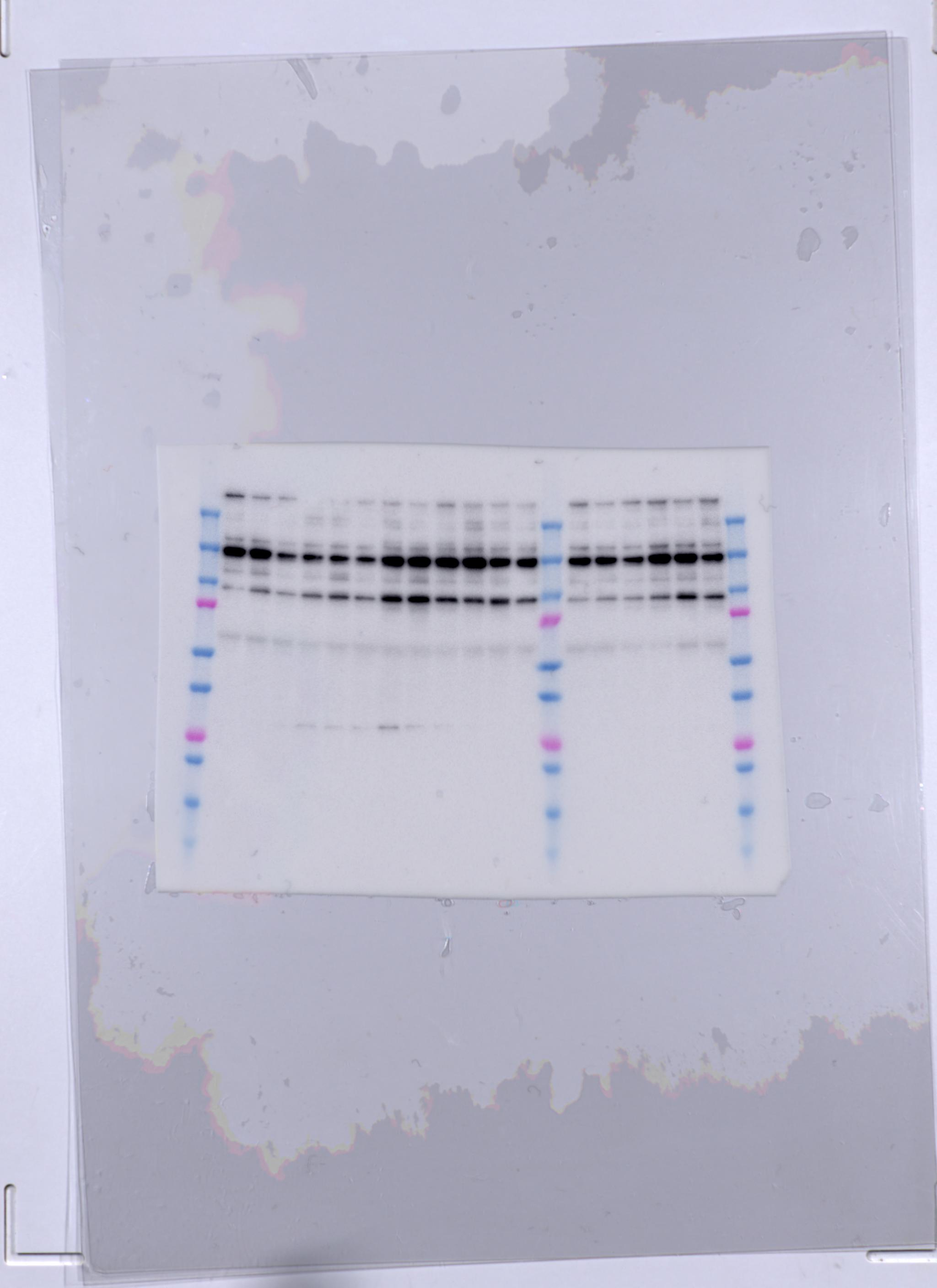

Supplement: Figure 4—source data 1. [file elife-81573-fig4-data1.zip › Figure 4-source data 1/Figure 4-source data 1_raw files/LK220708 Fig4C CK2Sb 2022.07.08_19.09.28_Ch+Marker.jpg]

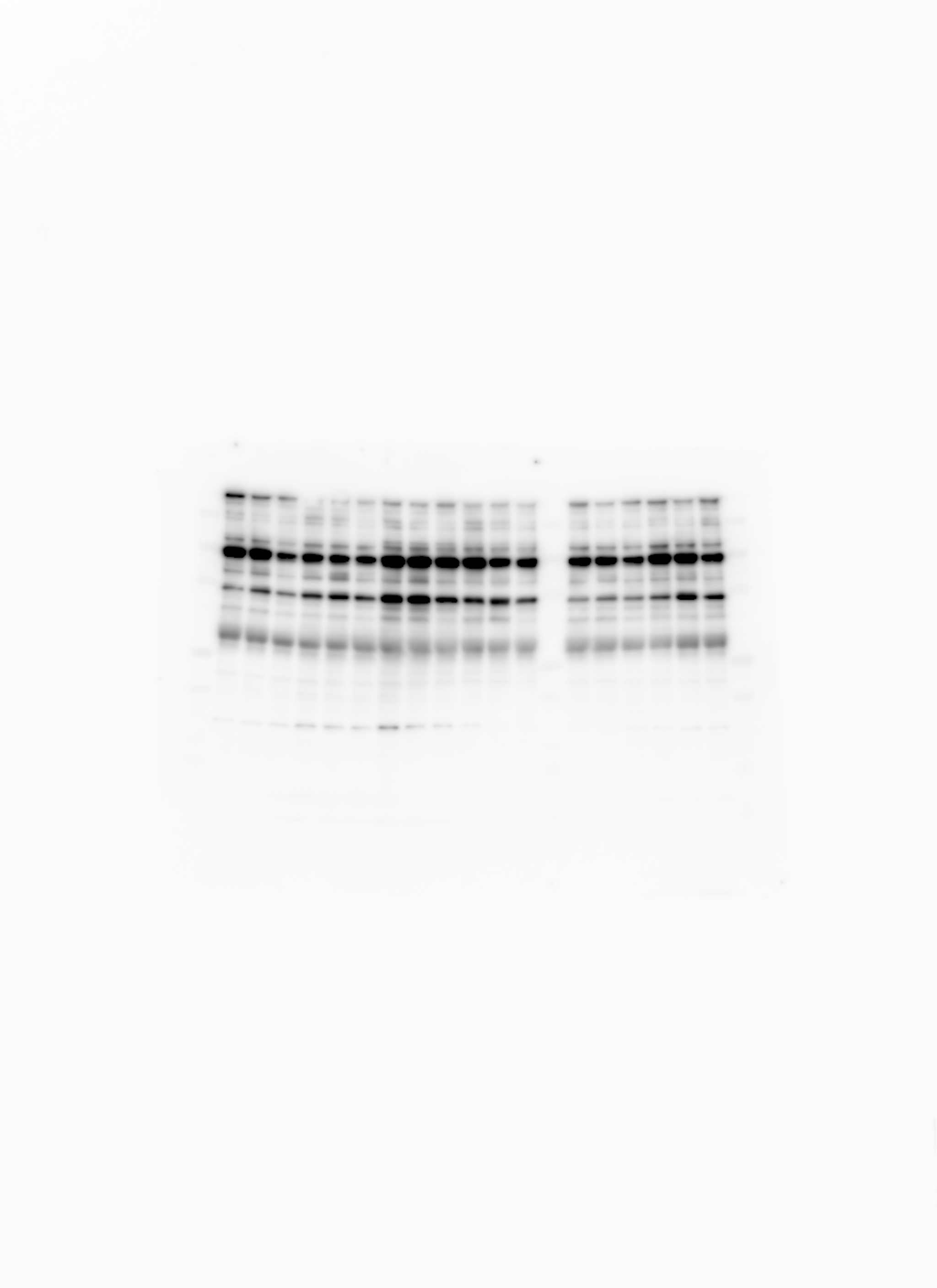

Supplement: Figure 4—source data 1. [file elife-81573-fig4-data1.zip › Figure 4-source data 1/Figure 4-source data 1_raw files/LK220708 Fig4C CK2Sb 2022.07.08_19.11.10-12_Ch.tif]

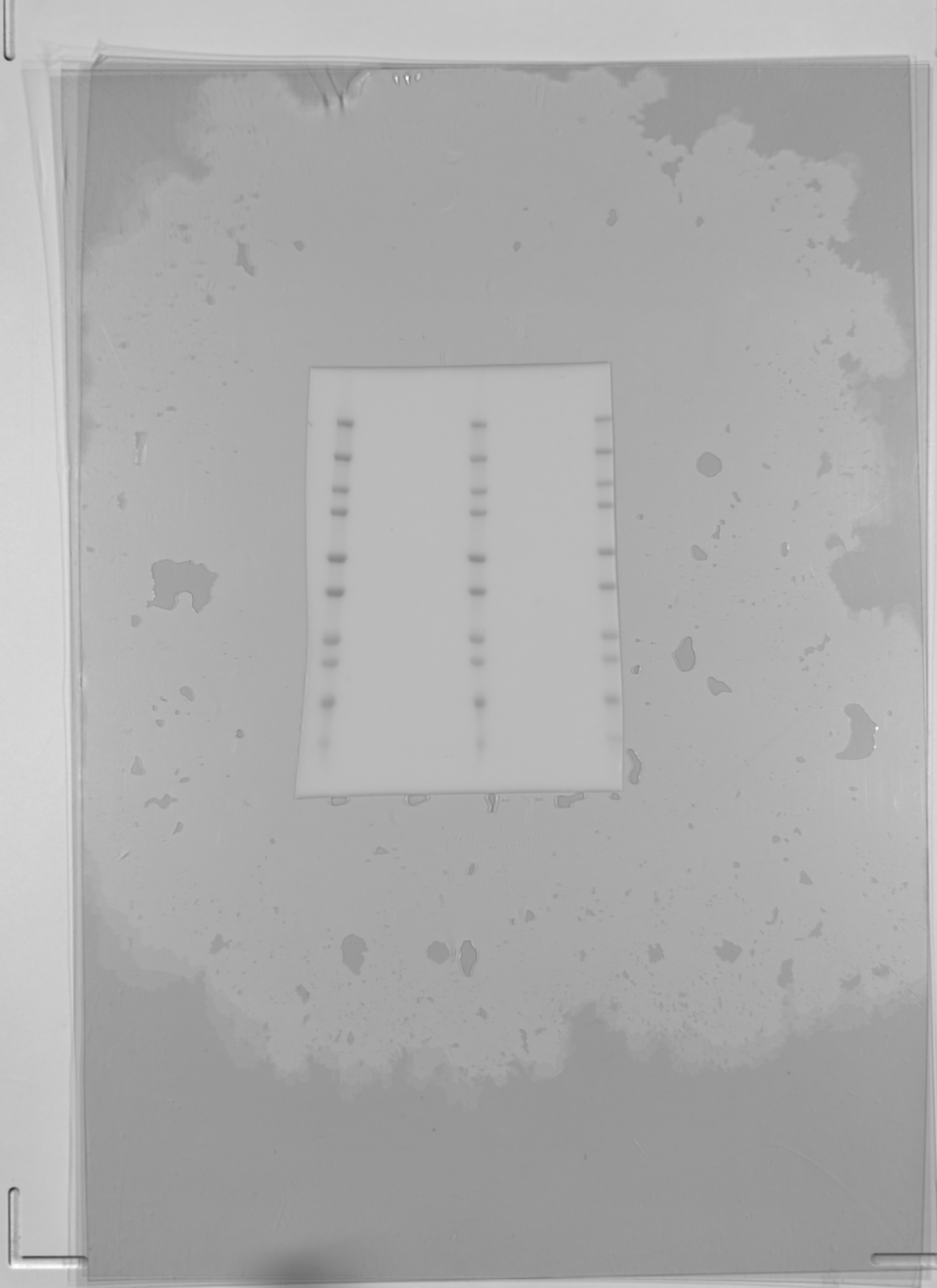

Supplement: Figure 4—source data 2. [file elife-81573-fig4-data2.zip › Figure 4-source data 2/Figure 4-source data 2_raw files/LK220709 CK2iHAIP BT 2022.07.10_15.05.00_Ch-Marker.tif]

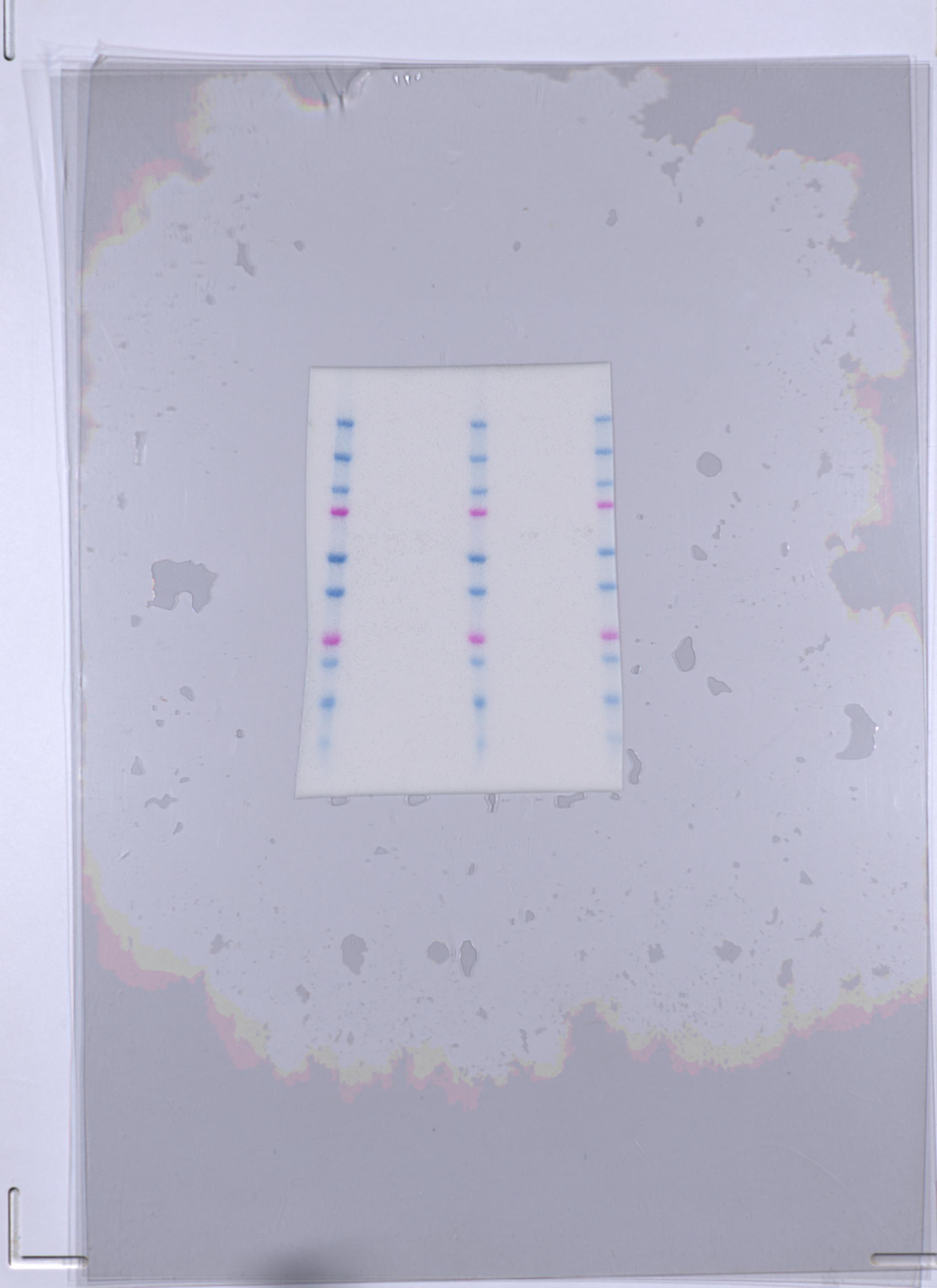

Supplement: Figure 4—source data 2. [file elife-81573-fig4-data2.zip › Figure 4-source data 2/Figure 4-source data 2_raw files/LK220709 CK2iHAIP BT 2022.07.10_15.05.00_Ch+Marker.jpg]

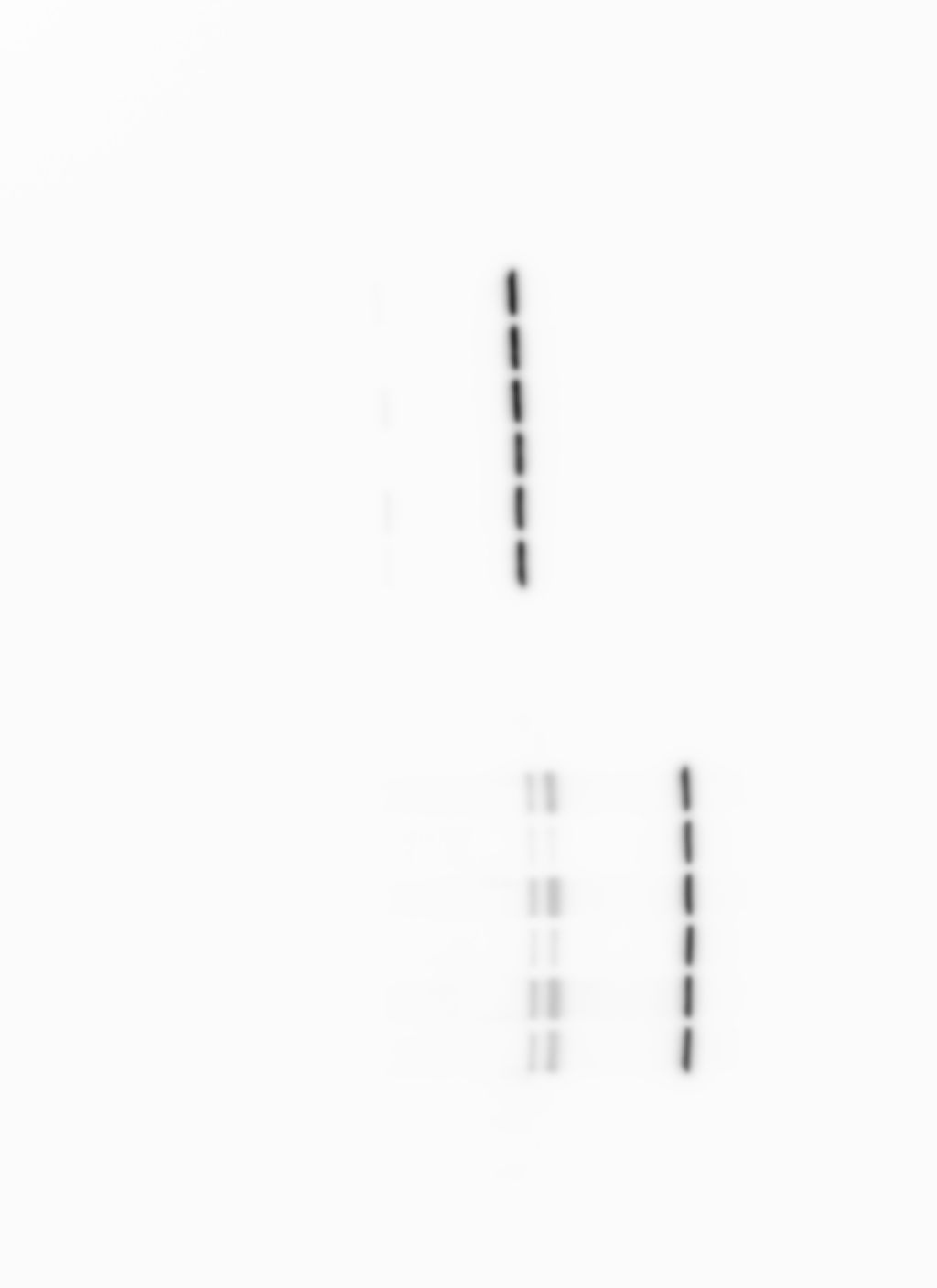

Supplement: Figure 4—figure supplement 1—source data 1. [file elife-81573-fig4-figsupp1-data1.zip › Figure 4-supplement 1-source data 1/Figure 4-supplement 1-source data 1_raw files/SUN2 sgCK2b Tub 2022.03.28_17.52.30_Ch v Tub/SUN2 sgCK2b Tub 2022.03.28_17.52.30_Ch.tif]

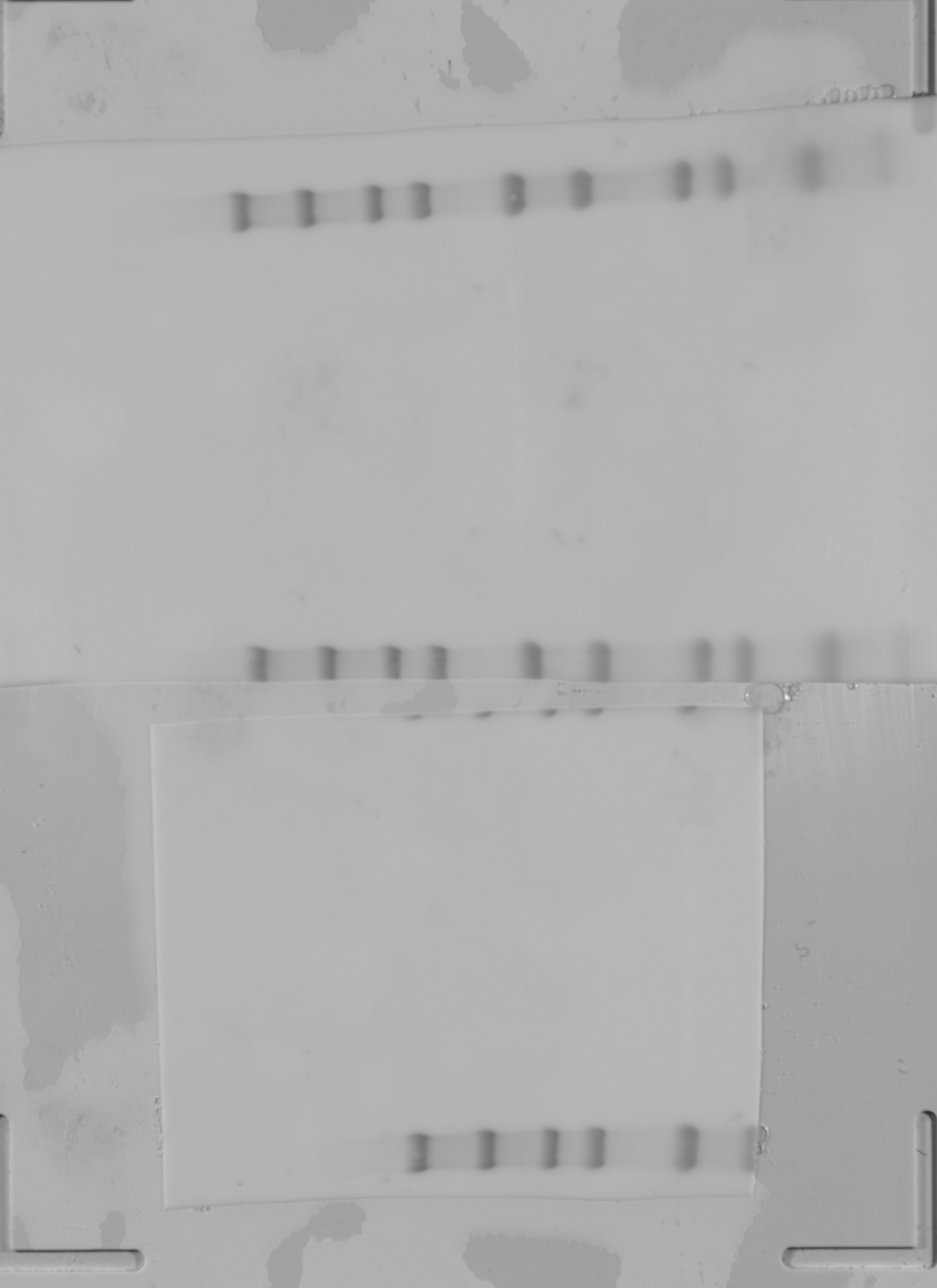

Supplement: Figure 4—figure supplement 1—source data 1. [file elife-81573-fig4-figsupp1-data1.zip › Figure 4-supplement 1-source data 1/Figure 4-supplement 1-source data 1_raw files/SUN2 sgCK2b Tub 2022.03.28_17.52.30_Ch v Tub/SUN2 sgCK2b Tub 2022.03.28_17.52.30_Ch-Marker.tif]

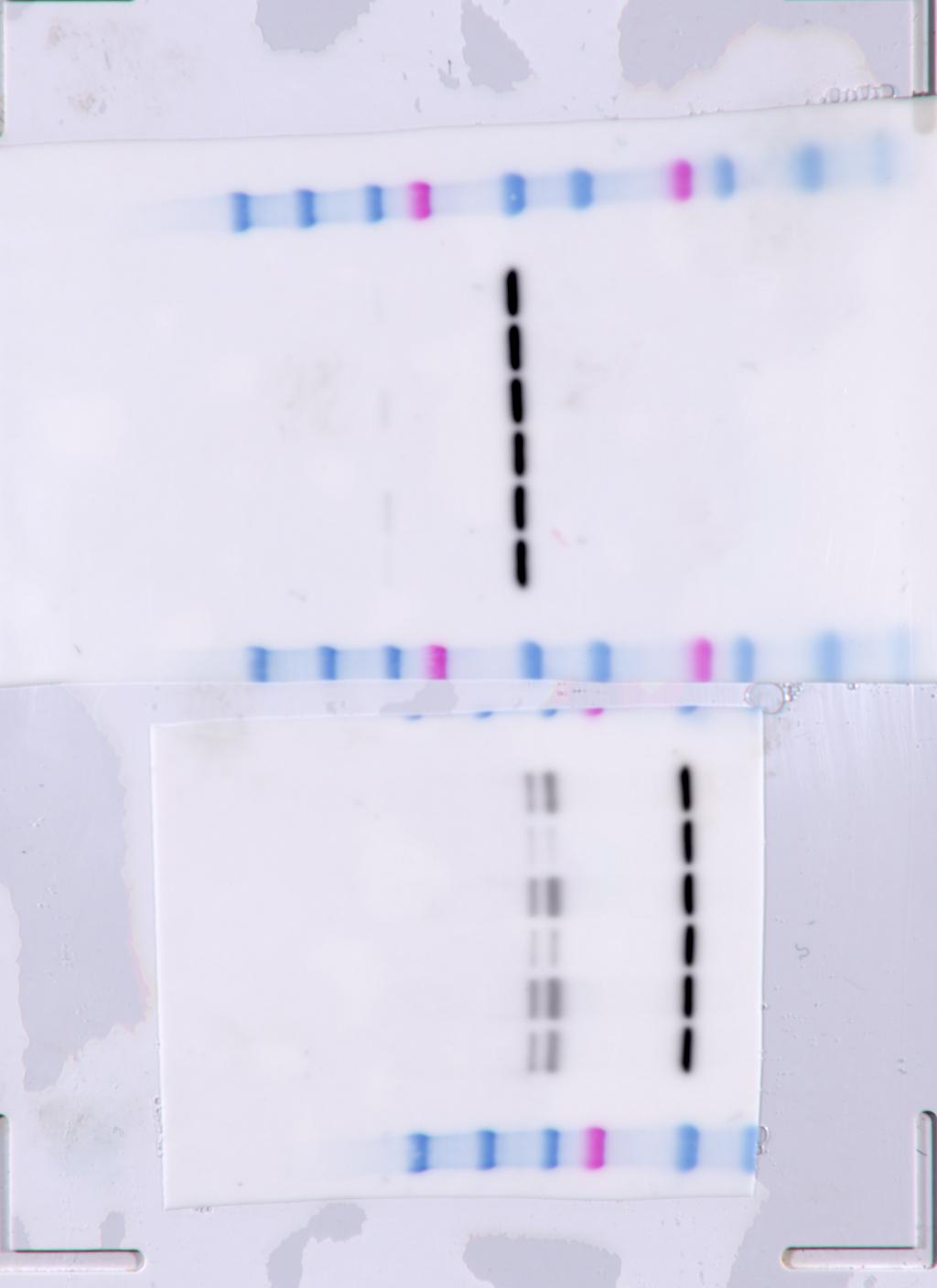

Supplement: Figure 4—figure supplement 1—source data 1. [file elife-81573-fig4-figsupp1-data1.zip › Figure 4-supplement 1-source data 1/Figure 4-supplement 1-source data 1_raw files/SUN2 sgCK2b Tub 2022.03.28_17.52.30_Ch v Tub/SUN2 sgCK2b Tub 2022.03.28_17.52.30_Ch+Marker.jpg]

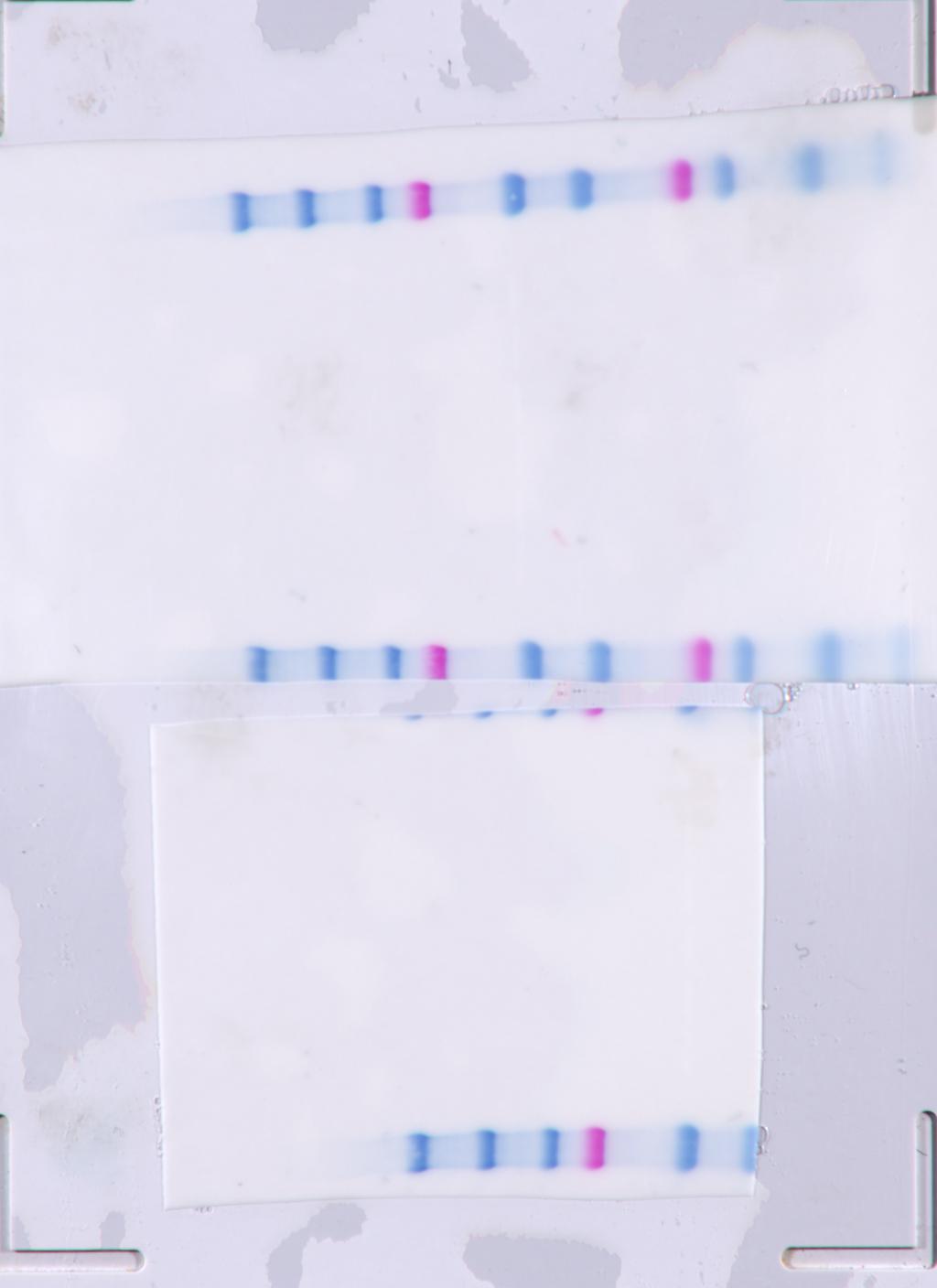

Supplement: Figure 4—figure supplement 1—source data 1. [file elife-81573-fig4-figsupp1-data1.zip › Figure 4-supplement 1-source data 1/Figure 4-supplement 1-source data 1_raw files/SUN2 sgCK2b Tub 2022.03.28_17.52.30_Ch v Tub/SUN2 sgCK2b Tub 2022.03.28_17.52.30_Ch-Marker.jpg]

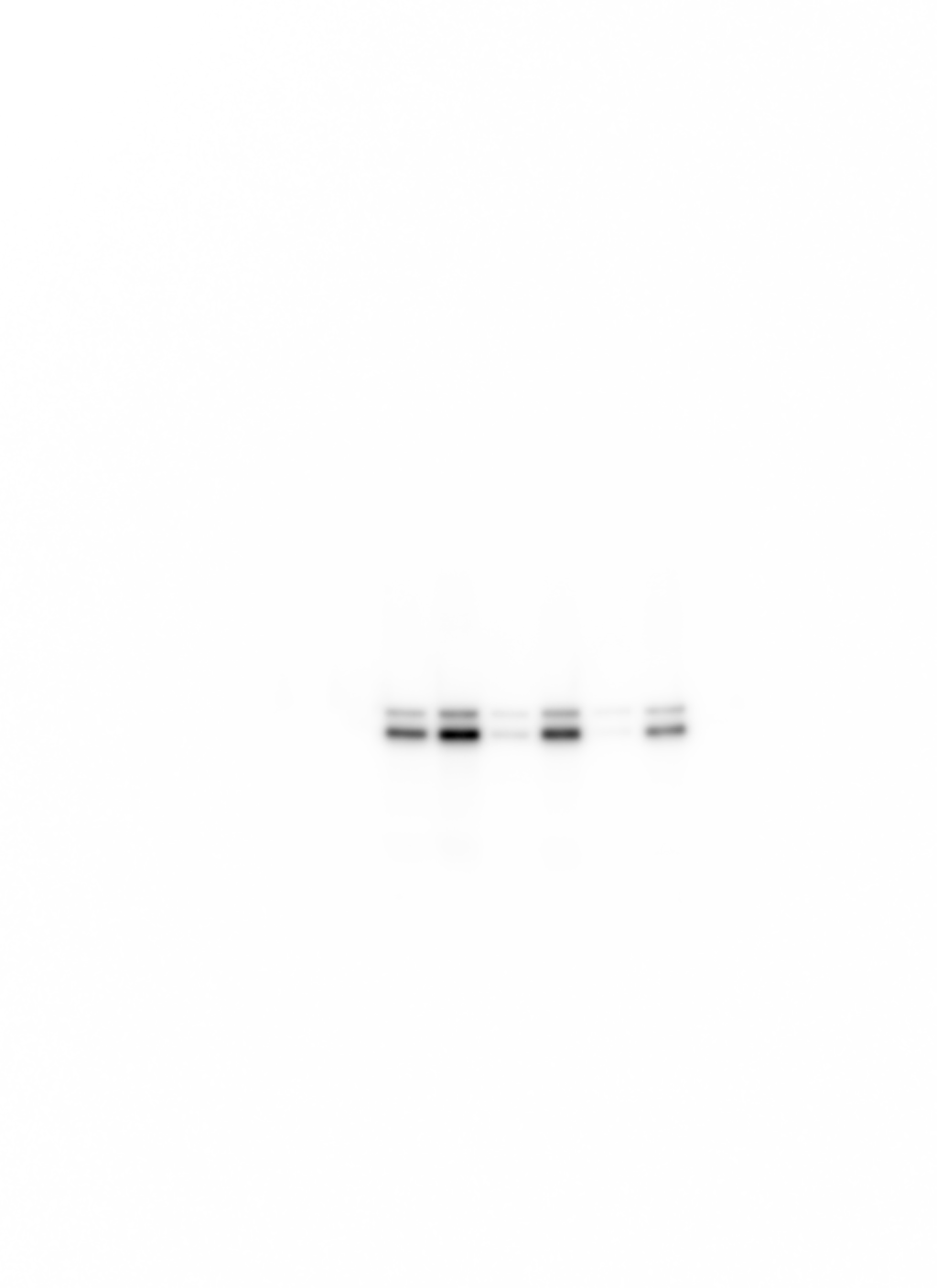

Supplement: Figure 4—figure supplement 1—source data 1. [file elife-81573-fig4-figsupp1-data1.zip › Figure 4-supplement 1-source data 1/Figure 4-supplement 1-source data 1_raw files/SUN2 sgCK2b HA 2022.03.25_21.48.14_Ch v HA/SUN2 sgCK2b HA 2022.03.25_21.48.14_Ch.tif]

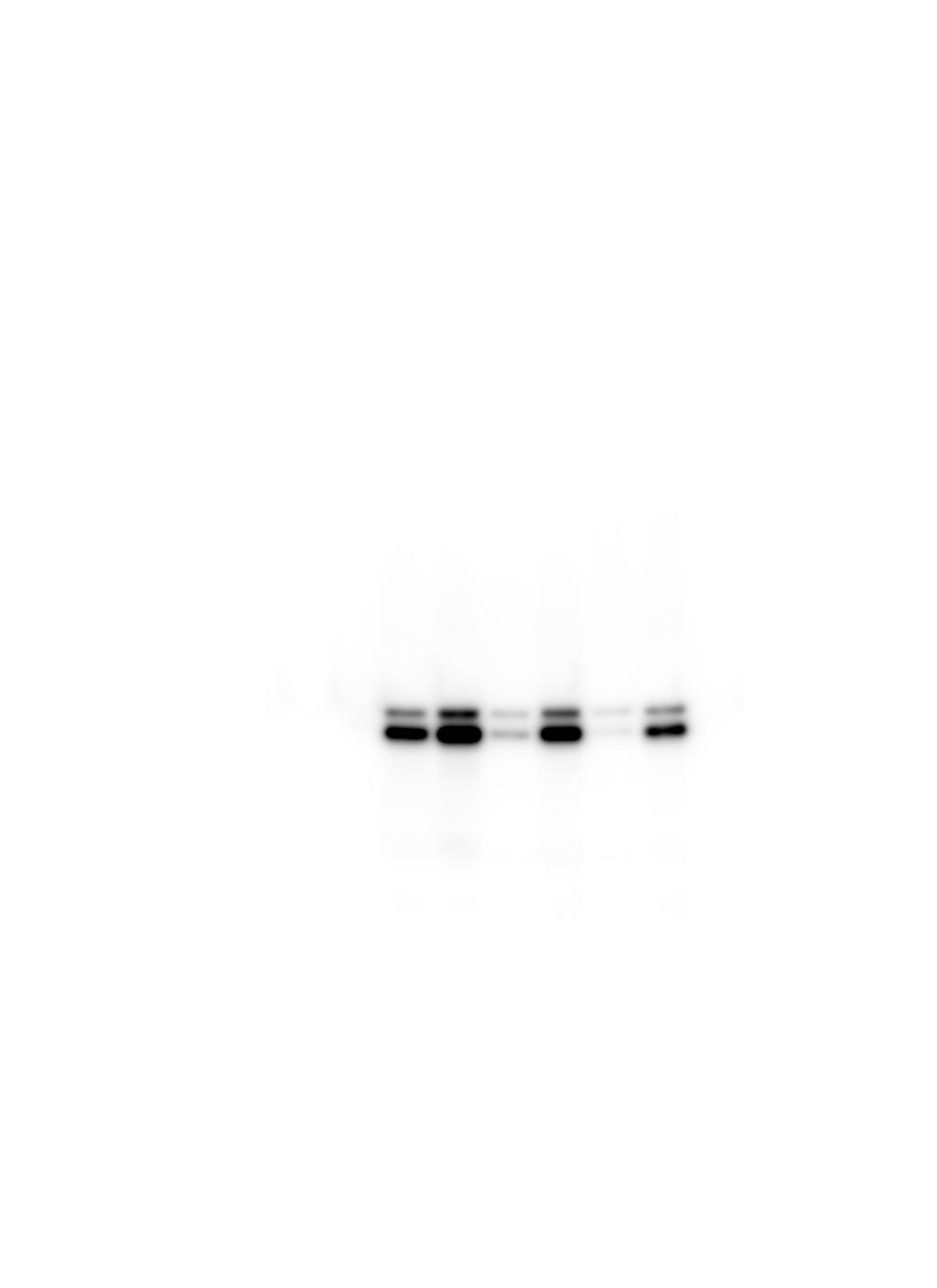

Supplement: Figure 4—figure supplement 1—source data 1. [file elife-81573-fig4-figsupp1-data1.zip › Figure 4-supplement 1-source data 1/Figure 4-supplement 1-source data 1_raw files/SUN2 sgCK2b HA 2022.03.25_21.48.14_Ch v HA/SUN2 sgCK2b HA 2022.03.25_21.48.14_Ch.jpg]

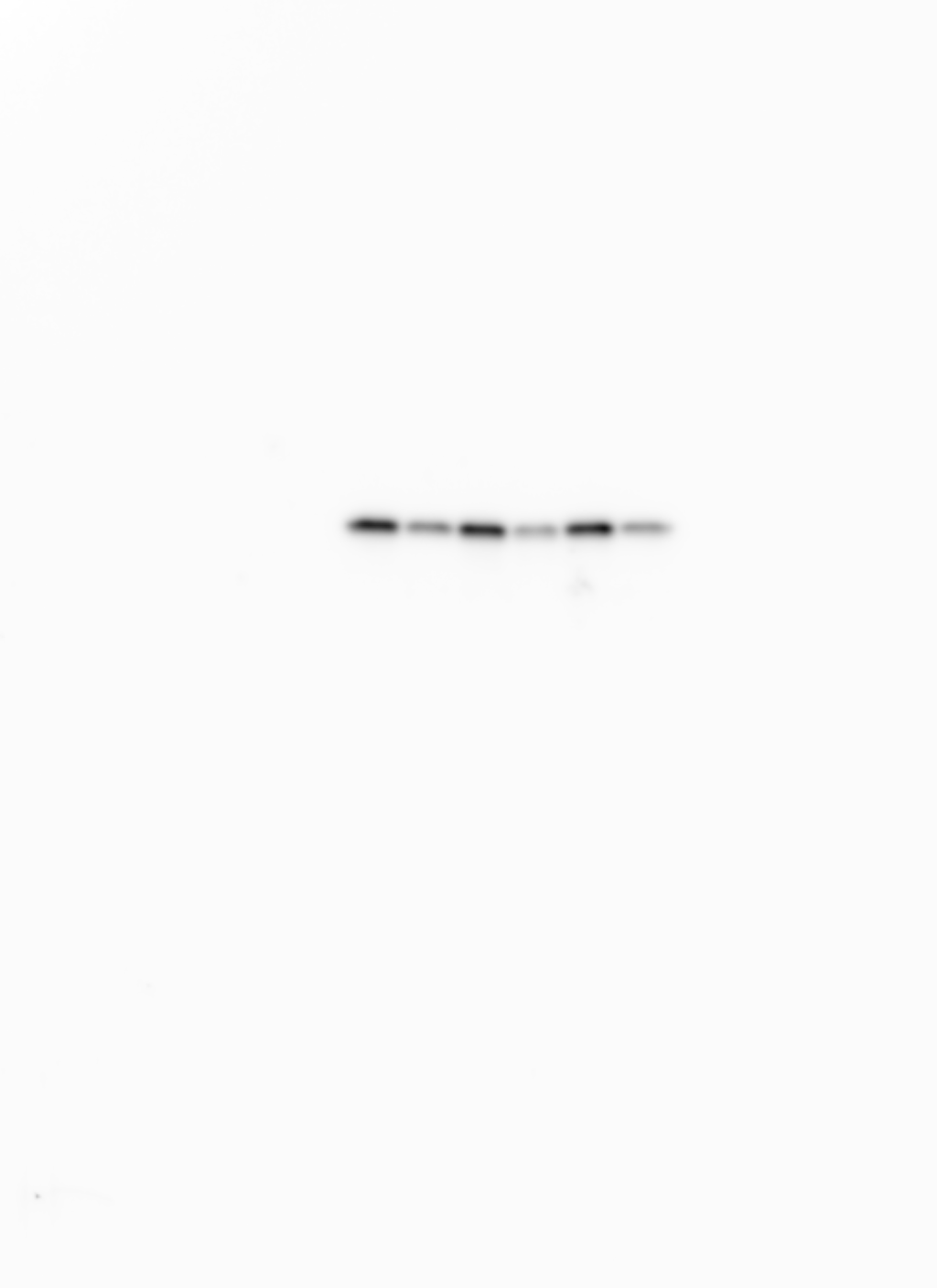

Supplement: Figure 4—figure supplement 1—source data 1. [file elife-81573-fig4-figsupp1-data1.zip › Figure 4-supplement 1-source data 1/Figure 4-supplement 1-source data 1_raw files/SUN2 sgCK2b CK2b 2022.03.25_21.36.18-05_Ch/SUN2 sgCK2b CK2b 2022.03.25_21.36.18-05_Ch.tif]

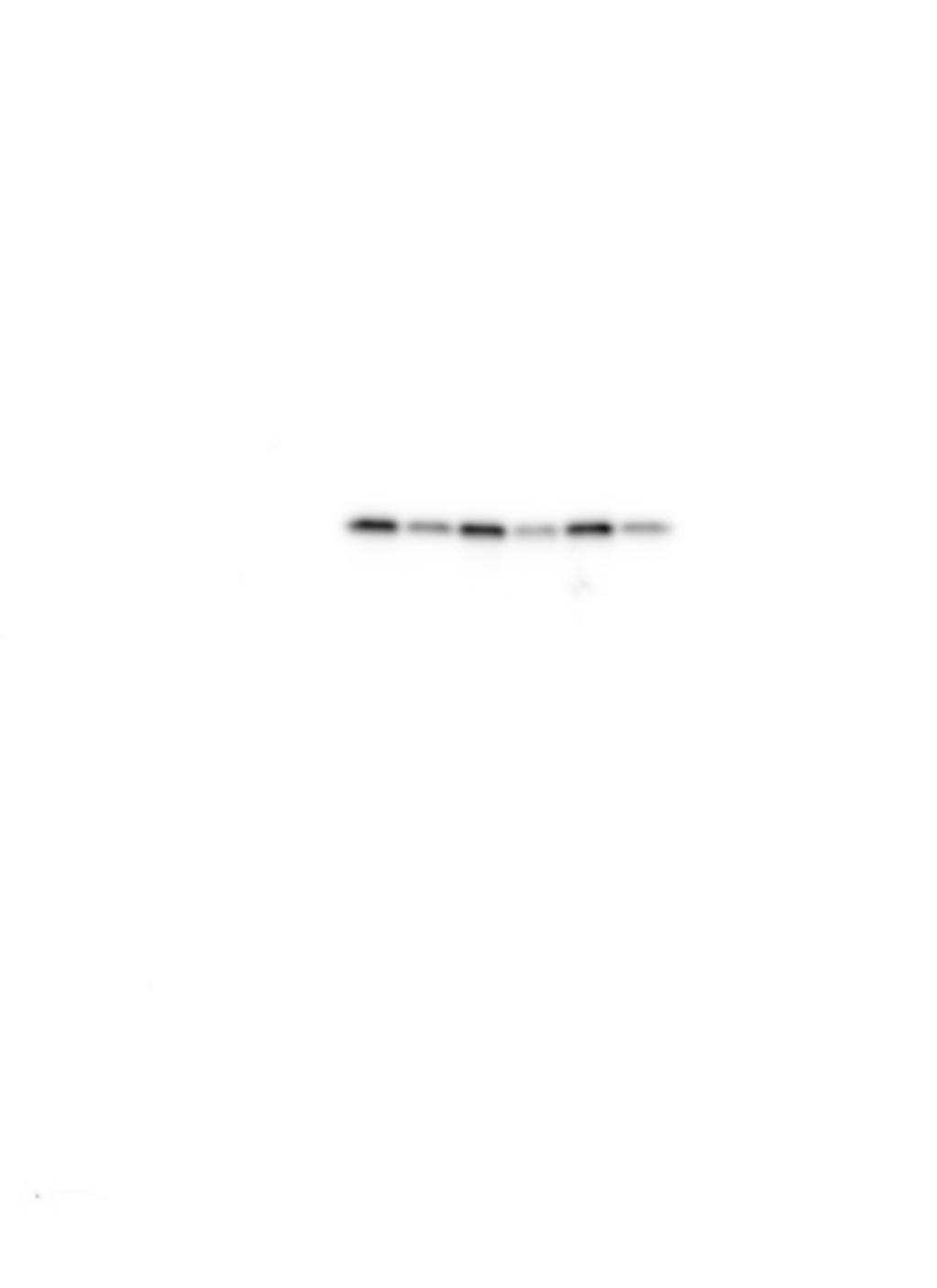

Supplement: Figure 4—figure supplement 1—source data 1. [file elife-81573-fig4-figsupp1-data1.zip › Figure 4-supplement 1-source data 1/Figure 4-supplement 1-source data 1_raw files/SUN2 sgCK2b CK2b 2022.03.25_21.36.18-05_Ch/SUN2 sgCK2b CK2b 2022.03.25_21.36.18-05_Ch.jpg]

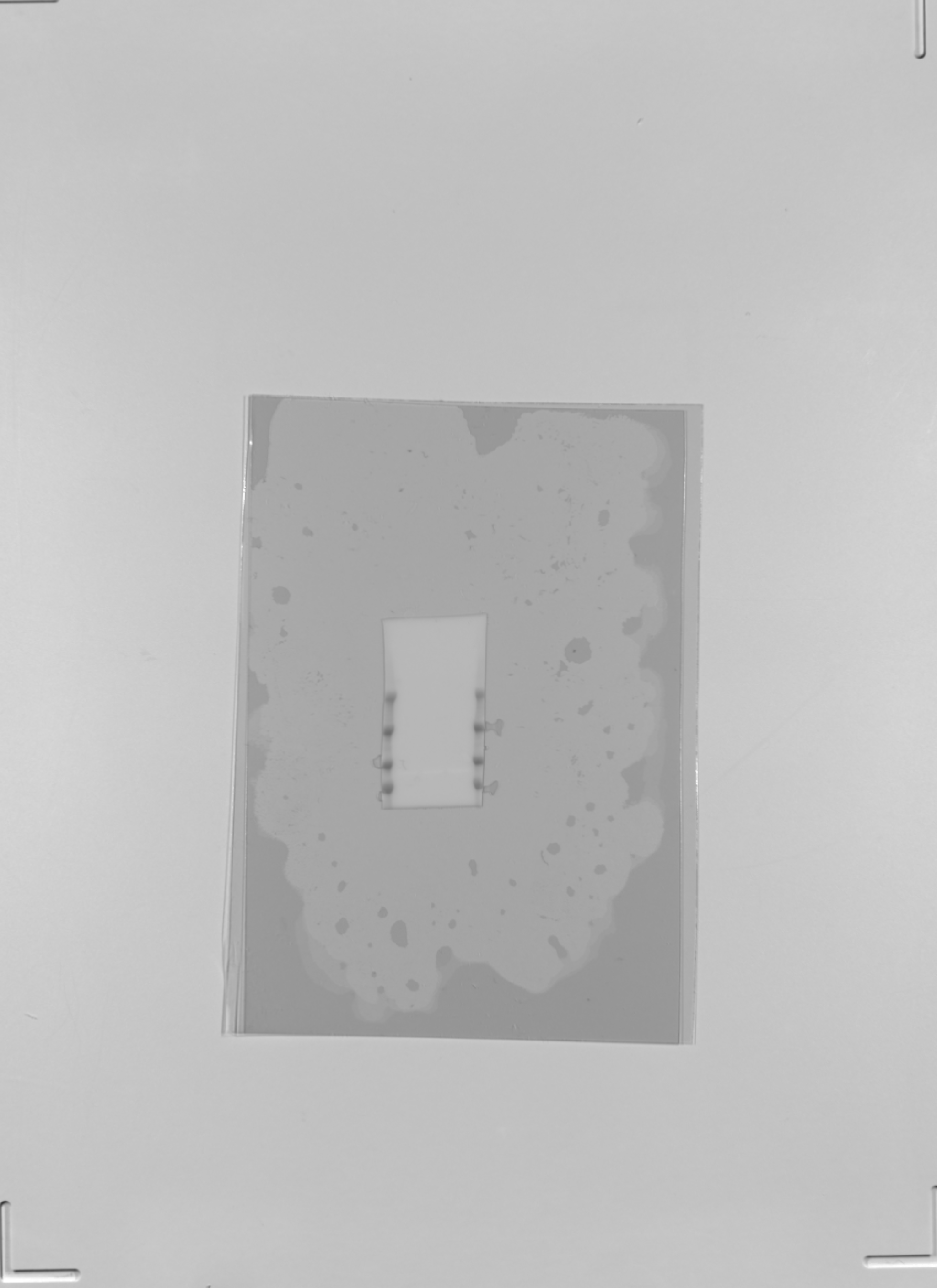

Supplement: Figure 5—source data 1. [file elife-81573-fig5-data1.zip › Figure 5-source data 1/Figure 5-source data 1_raw files/LK210313 ctdko cnx 2021.03.13_16.08.30_Ch-Marker.tif]

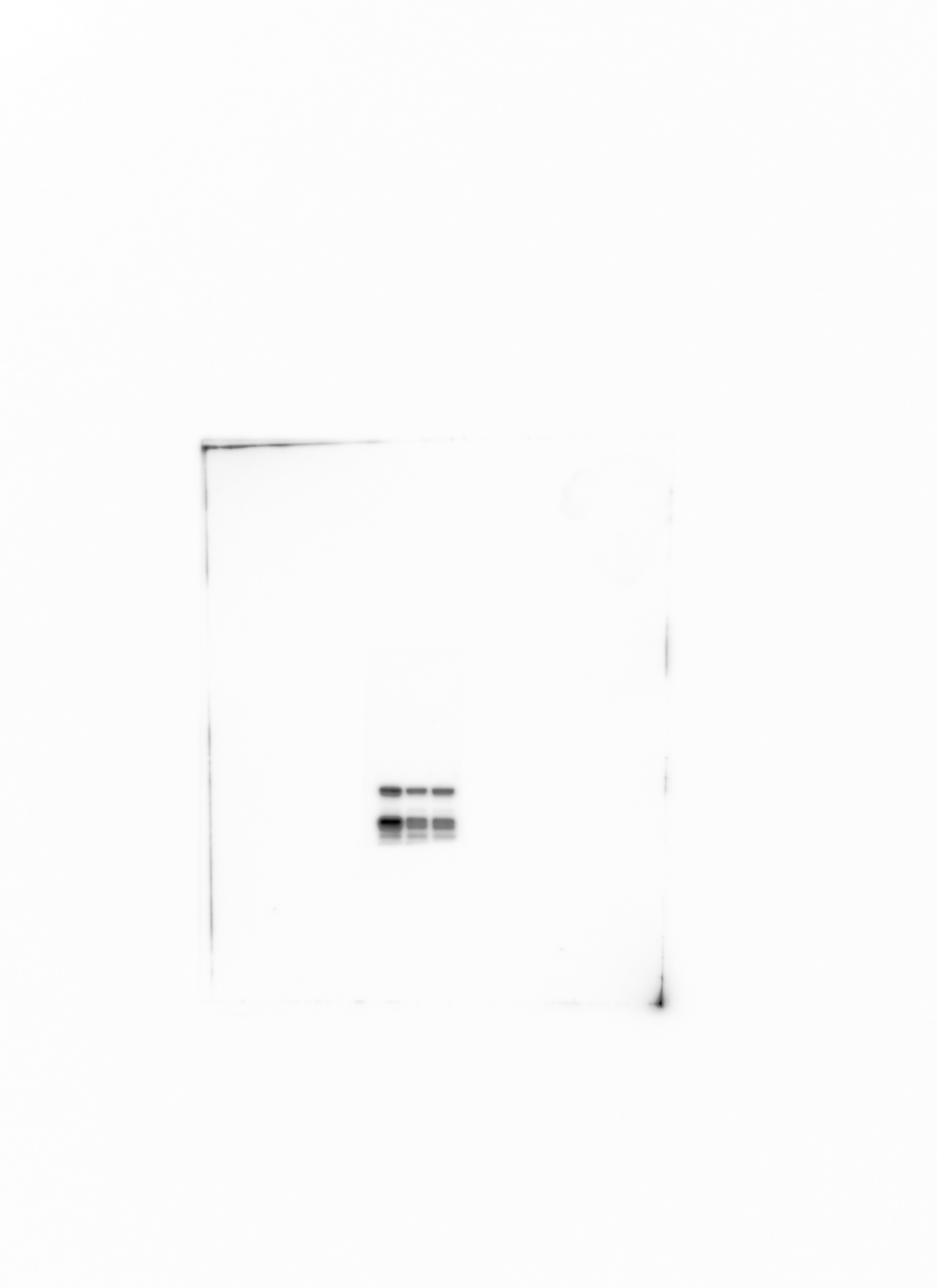

Supplement: Figure 5—source data 1. [file elife-81573-fig5-data1.zip › Figure 5-source data 1/Figure 5-source data 1_raw files/LK210313 ctdko sun2 2021.03.13_16.29.13-03_Ch.tif]

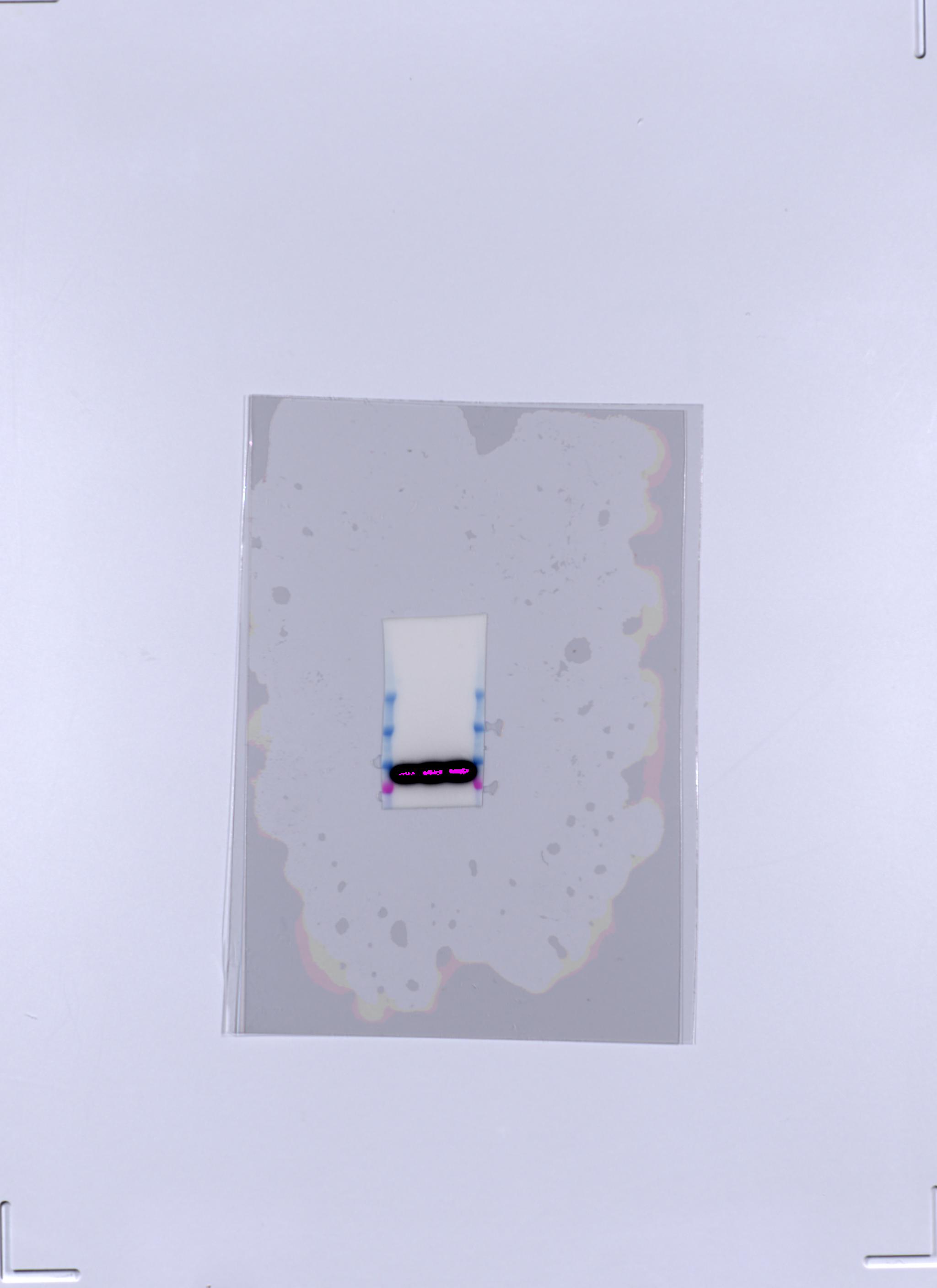

Supplement: Figure 5—source data 1. [file elife-81573-fig5-data1.zip › Figure 5-source data 1/Figure 5-source data 1_raw files/LK210313 ctdko cnx 2021.03.13_16.08.30_Ch+Marker.jpg]

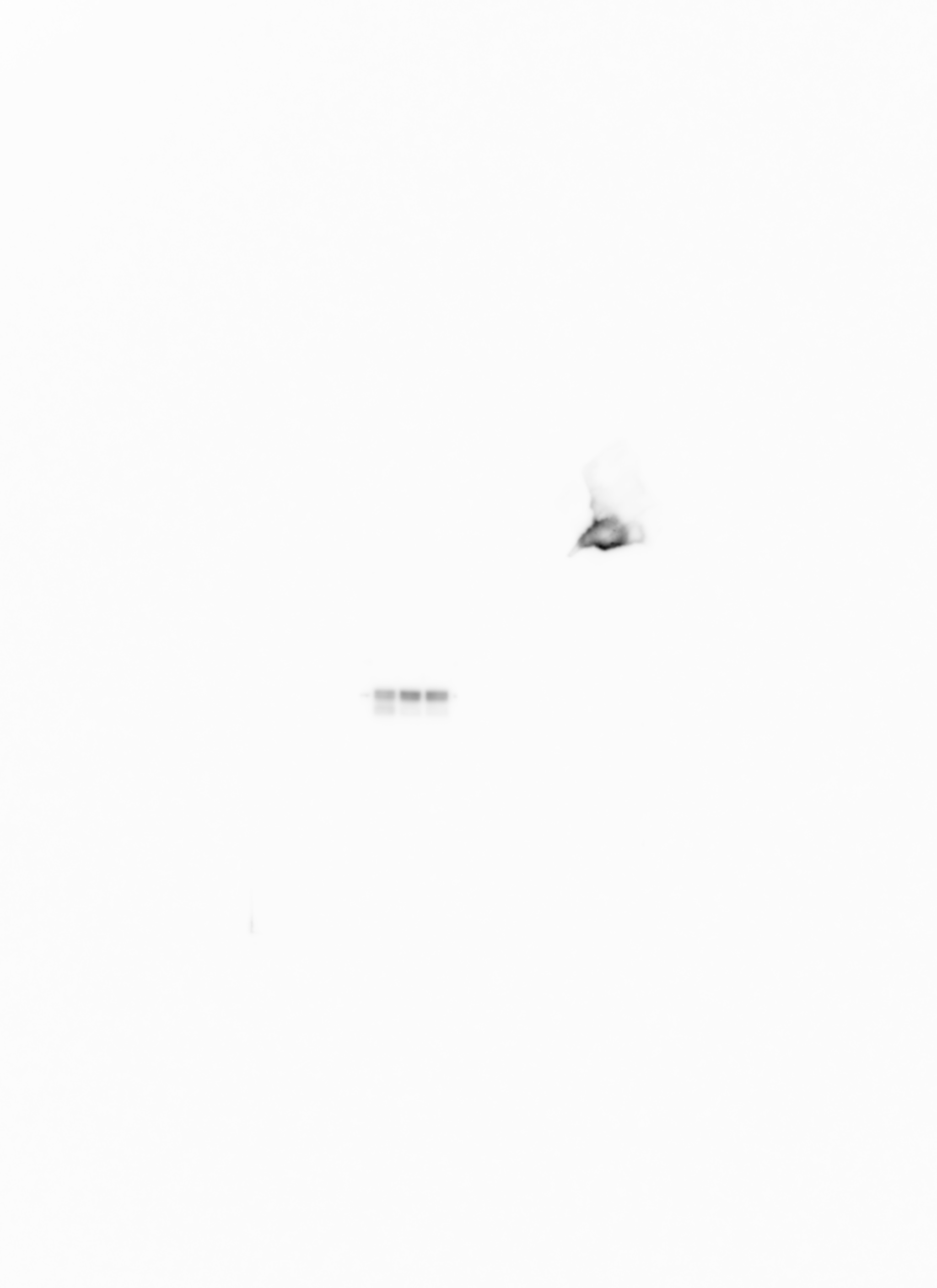

Supplement: Figure 5—source data 1. [file elife-81573-fig5-data1.zip › Figure 5-source data 1/Figure 5-source data 1_raw files/LK210313 ctdko sun1 2021.03.13_16.48.24-09_Ch.tif]

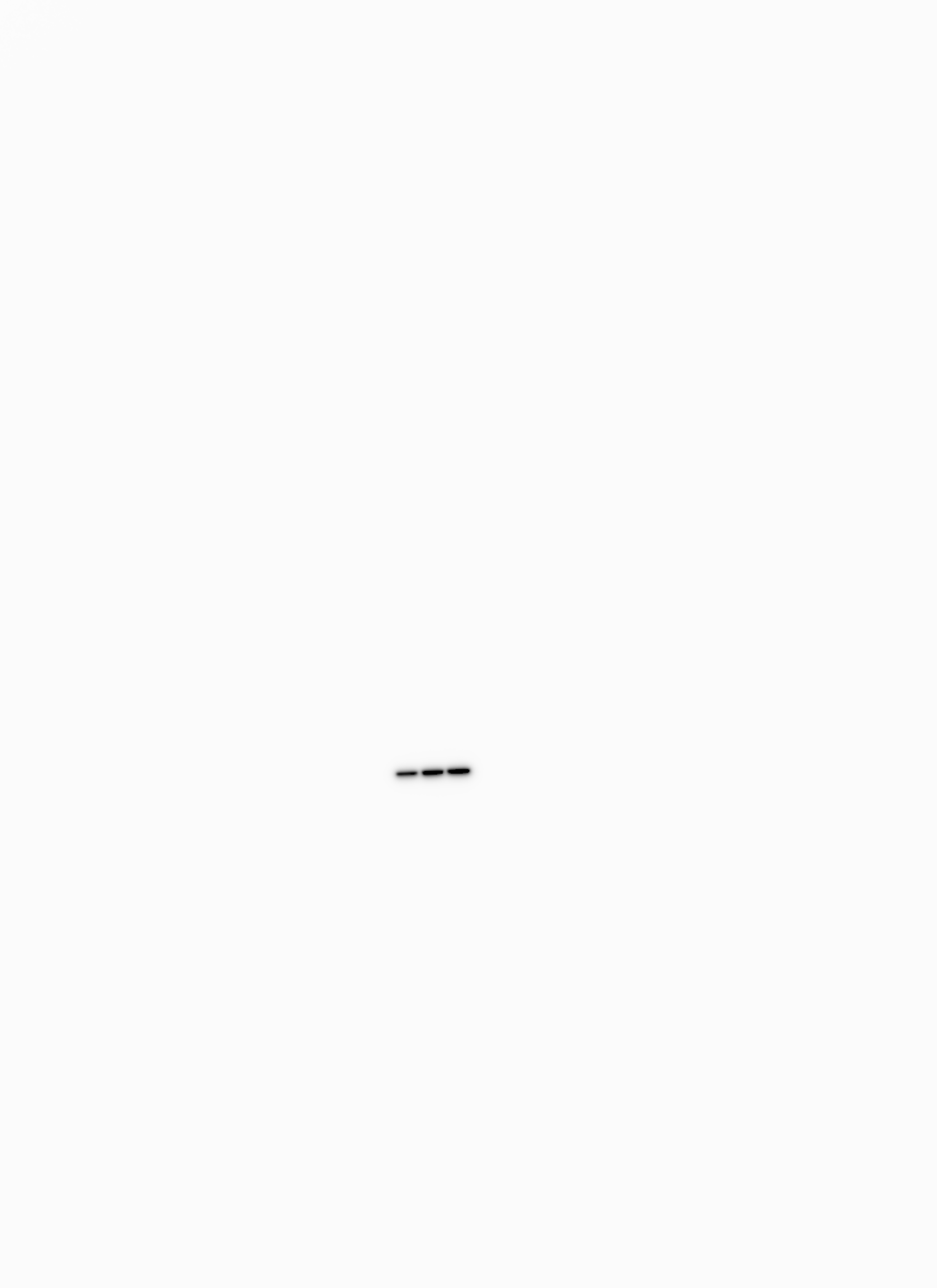

Supplement: Figure 5—source data 1. [file elife-81573-fig5-data1.zip › Figure 5-source data 1/Figure 5-source data 1_raw files/LK210313 ctdko cnx 2021.03.13_16.10.35-02_Ch.tif]

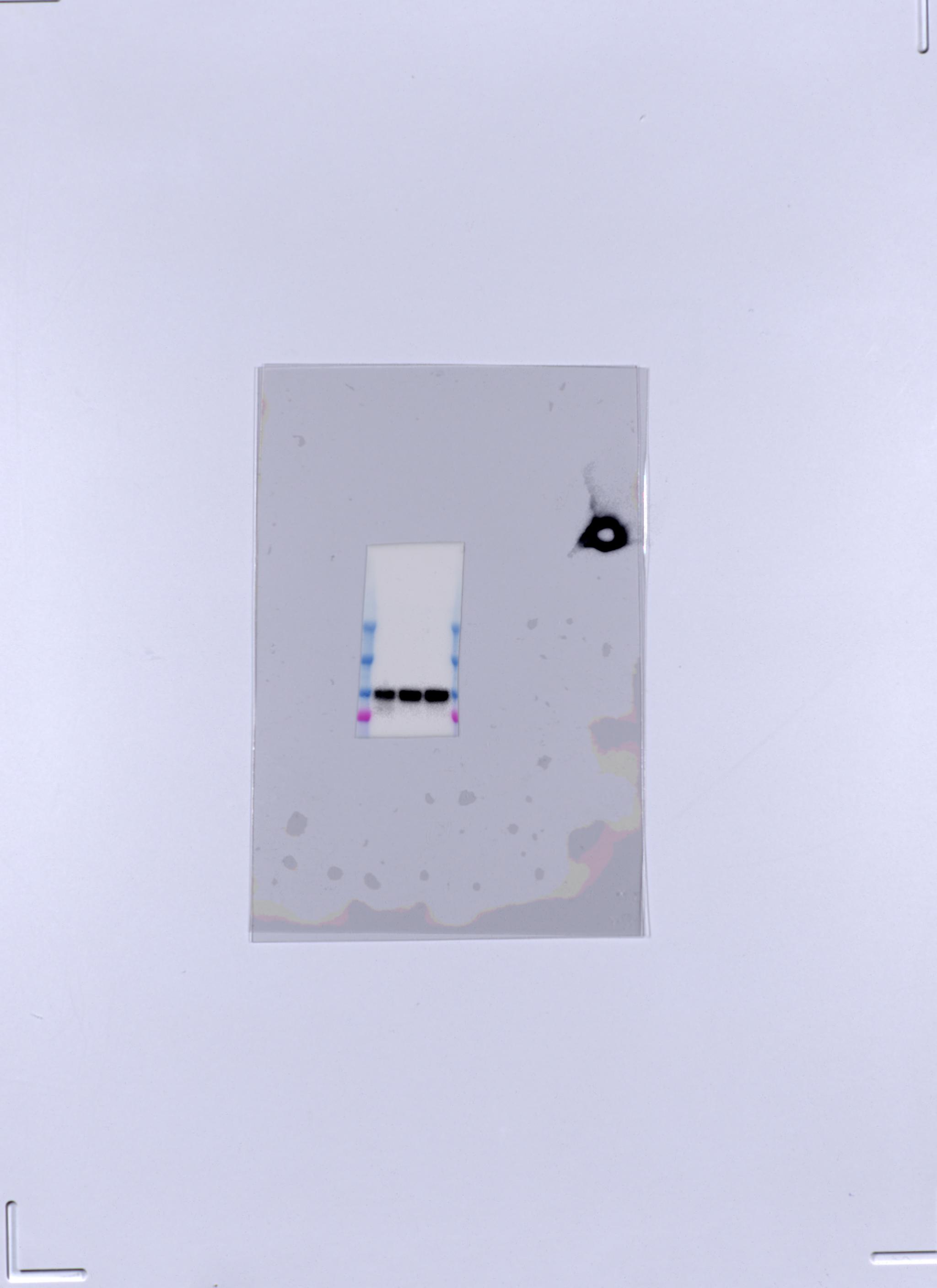

Supplement: Figure 5—source data 1. [file elife-81573-fig5-data1.zip › Figure 5-source data 1/Figure 5-source data 1_raw files/LK210313 ctdko sun1 2021.03.13_16.46.45_Ch+Marker.jpg]

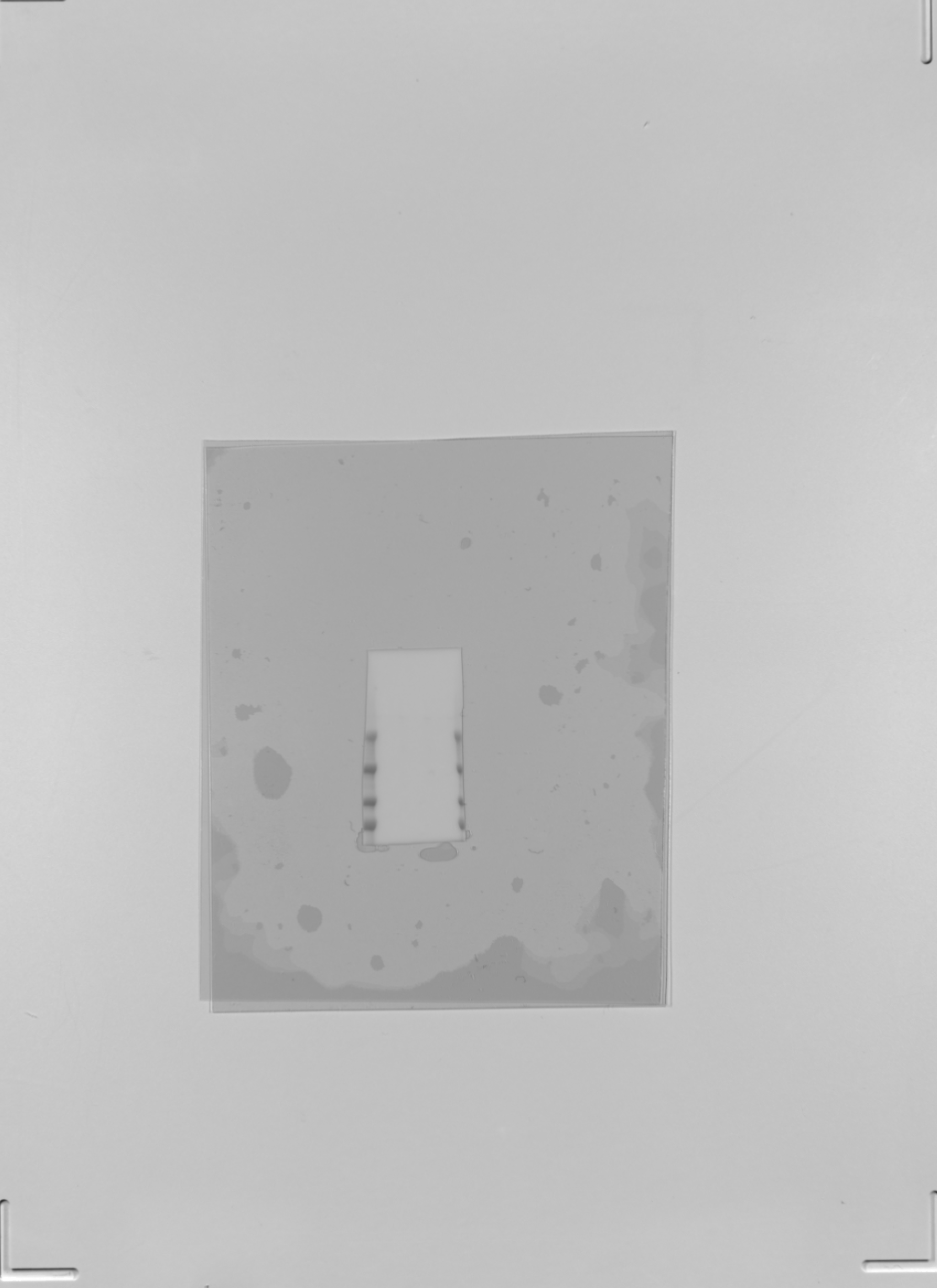

Supplement: Figure 5—source data 1. [file elife-81573-fig5-data1.zip › Figure 5-source data 1/Figure 5-source data 1_raw files/LK210313 ctdko sun2 2021.03.13_16.19.22_Ch-Marker.tif]

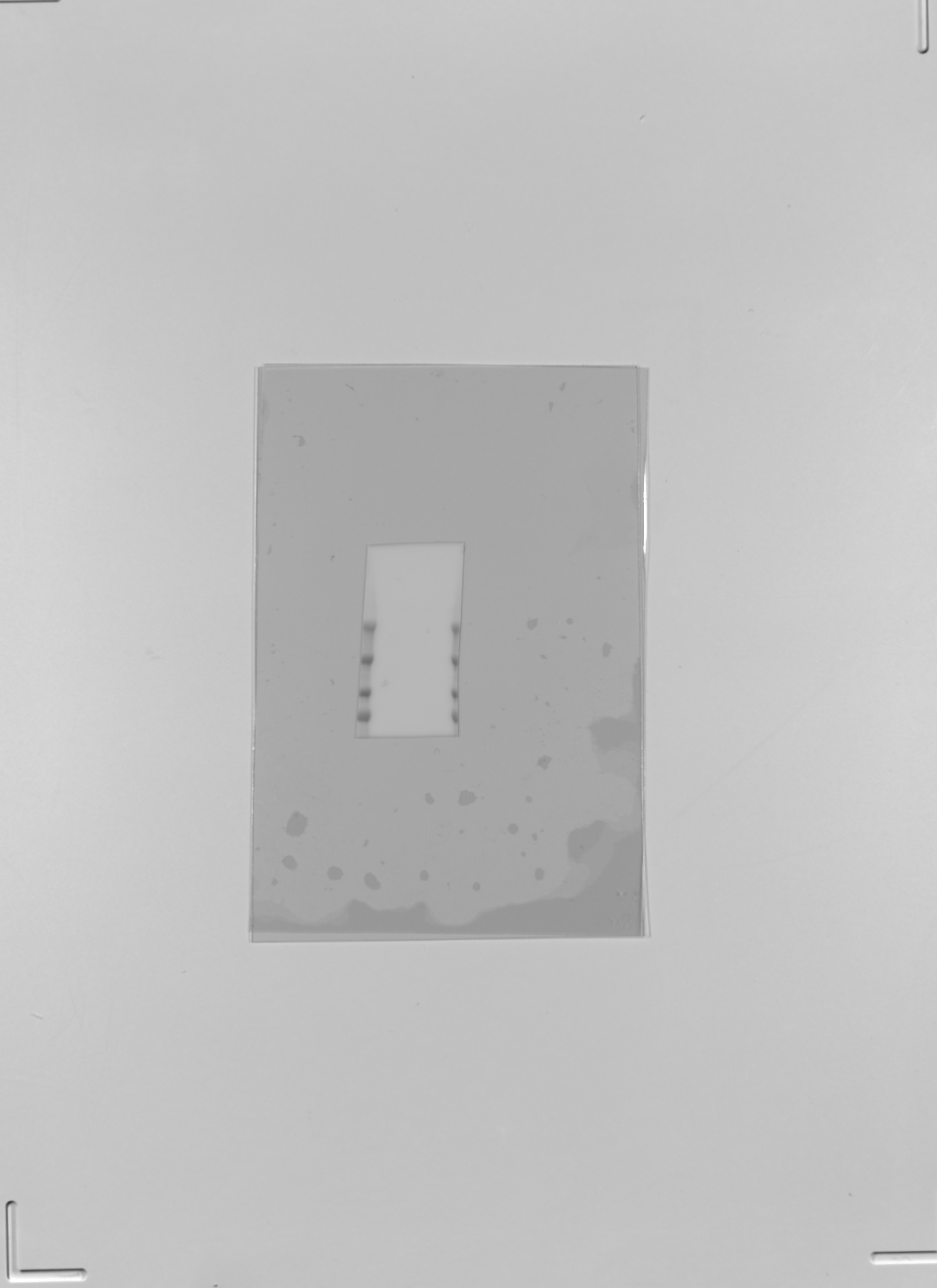

Supplement: Figure 5—source data 1. [file elife-81573-fig5-data1.zip › Figure 5-source data 1/Figure 5-source data 1_raw files/LK210313 ctdko sun1 2021.03.13_16.46.45_Ch-Marker.tif]

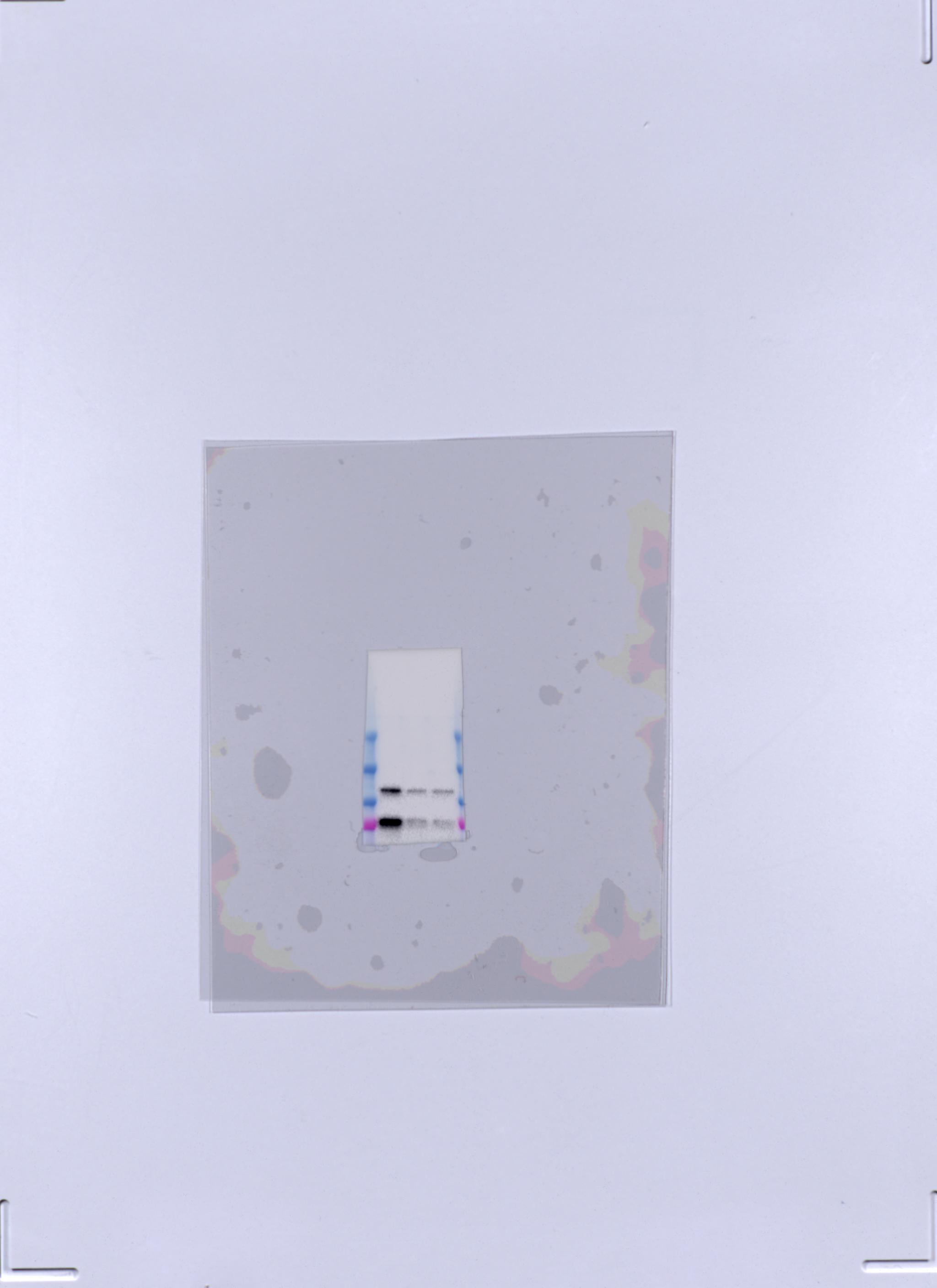

Supplement: Figure 5—source data 1. [file elife-81573-fig5-data1.zip › Figure 5-source data 1/Figure 5-source data 1_raw files/LK210313 ctdko sun2 2021.03.13_16.19.22_Ch+Marker.jpg]

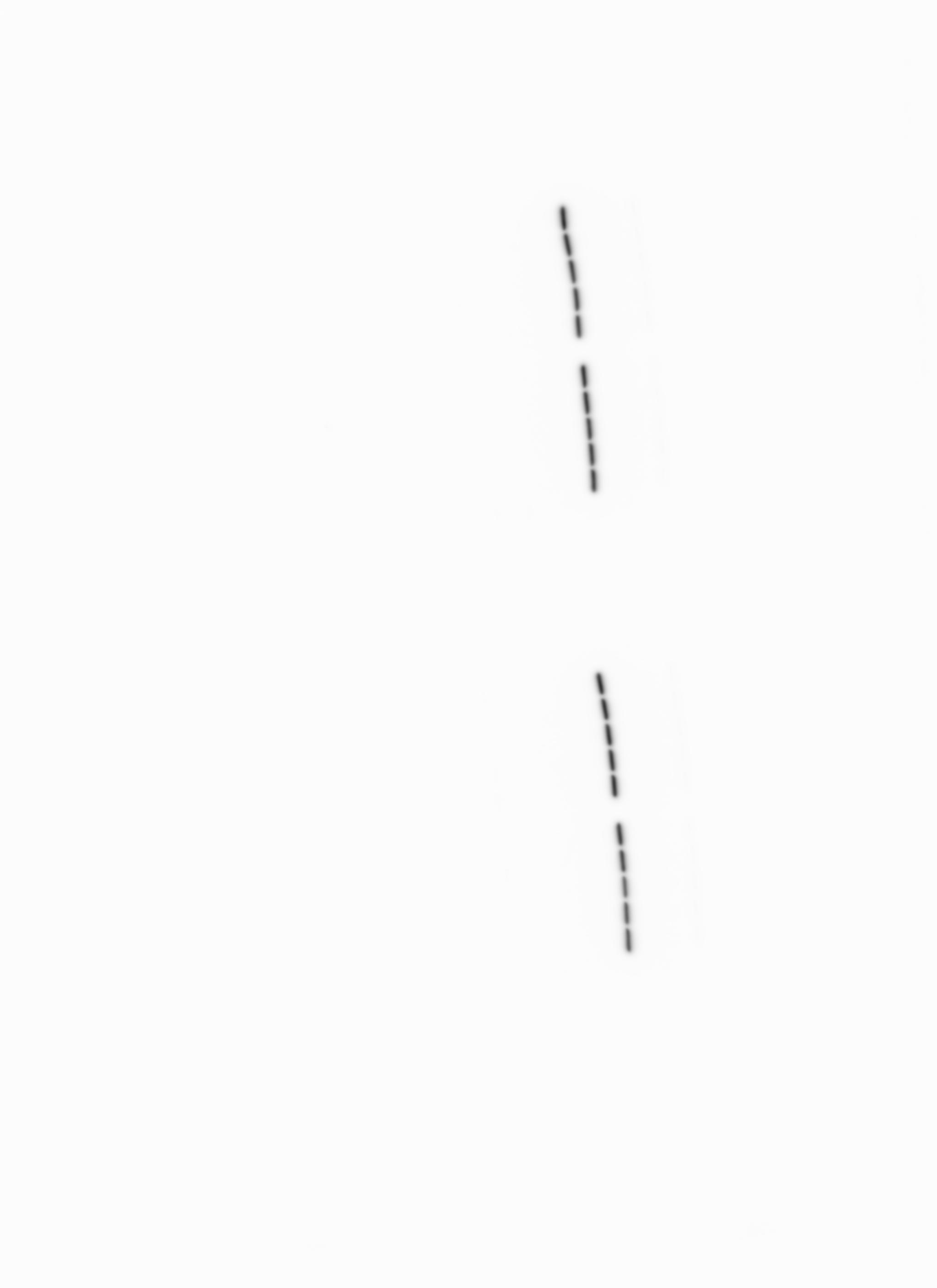

Supplement: Figure 5—source data 2. [file elife-81573-fig5-data2.zip › Figure 5-source data 2/Figure 5-source data 2_raw files/SUN2 end CHX Tub 2022.04.15_14.30.07-03_Ch v Tub/SUN2 end CHX Tub 2022.04.15_14.30.07-03_Ch.jpg]

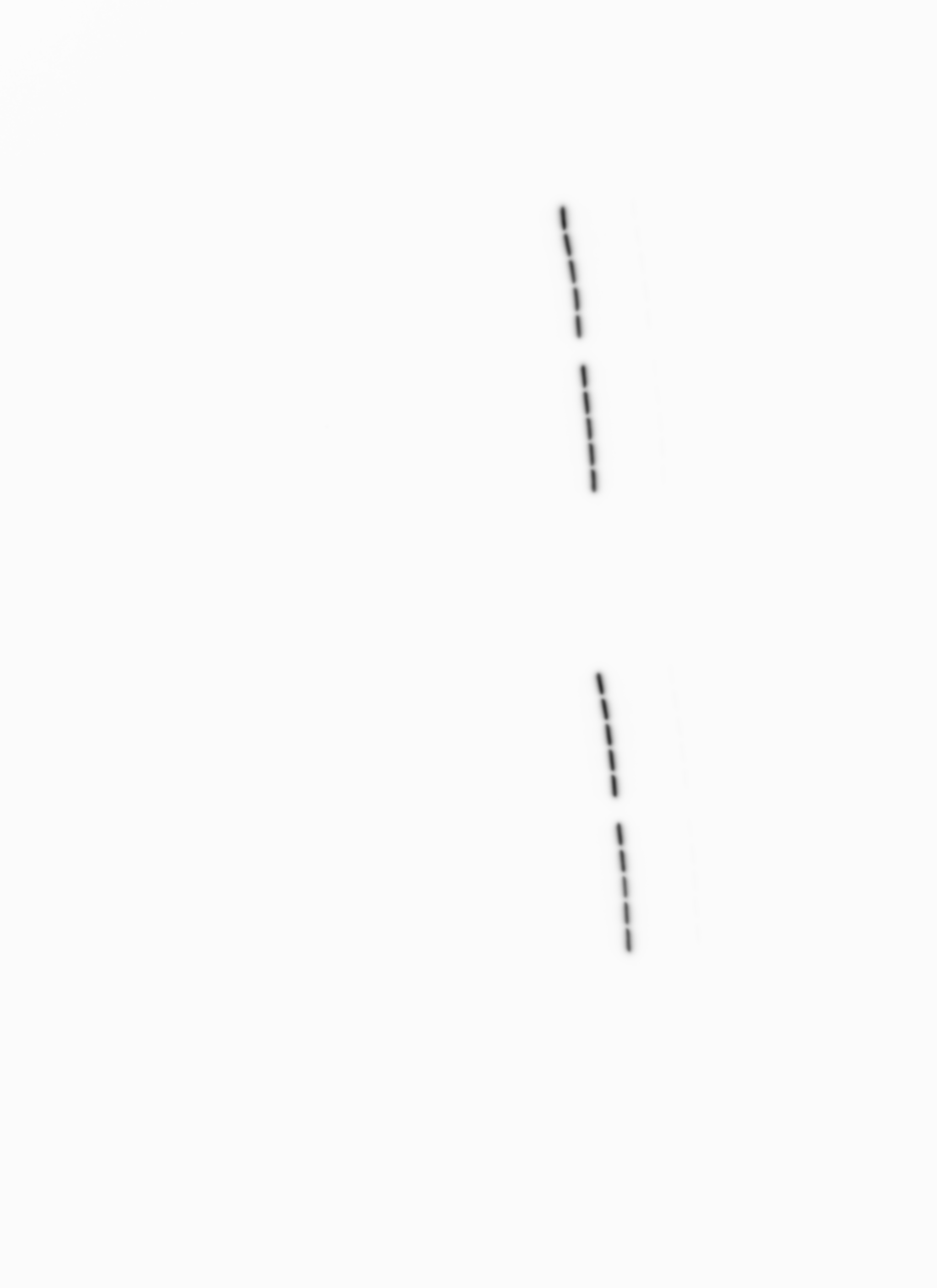

Supplement: Figure 5—source data 2. [file elife-81573-fig5-data2.zip › Figure 5-source data 2/Figure 5-source data 2_raw files/SUN2 end CHX Tub 2022.04.15_14.30.07-03_Ch v Tub/SUN2 end CHX Tub 2022.04.15_14.30.07-03_Ch.tif]

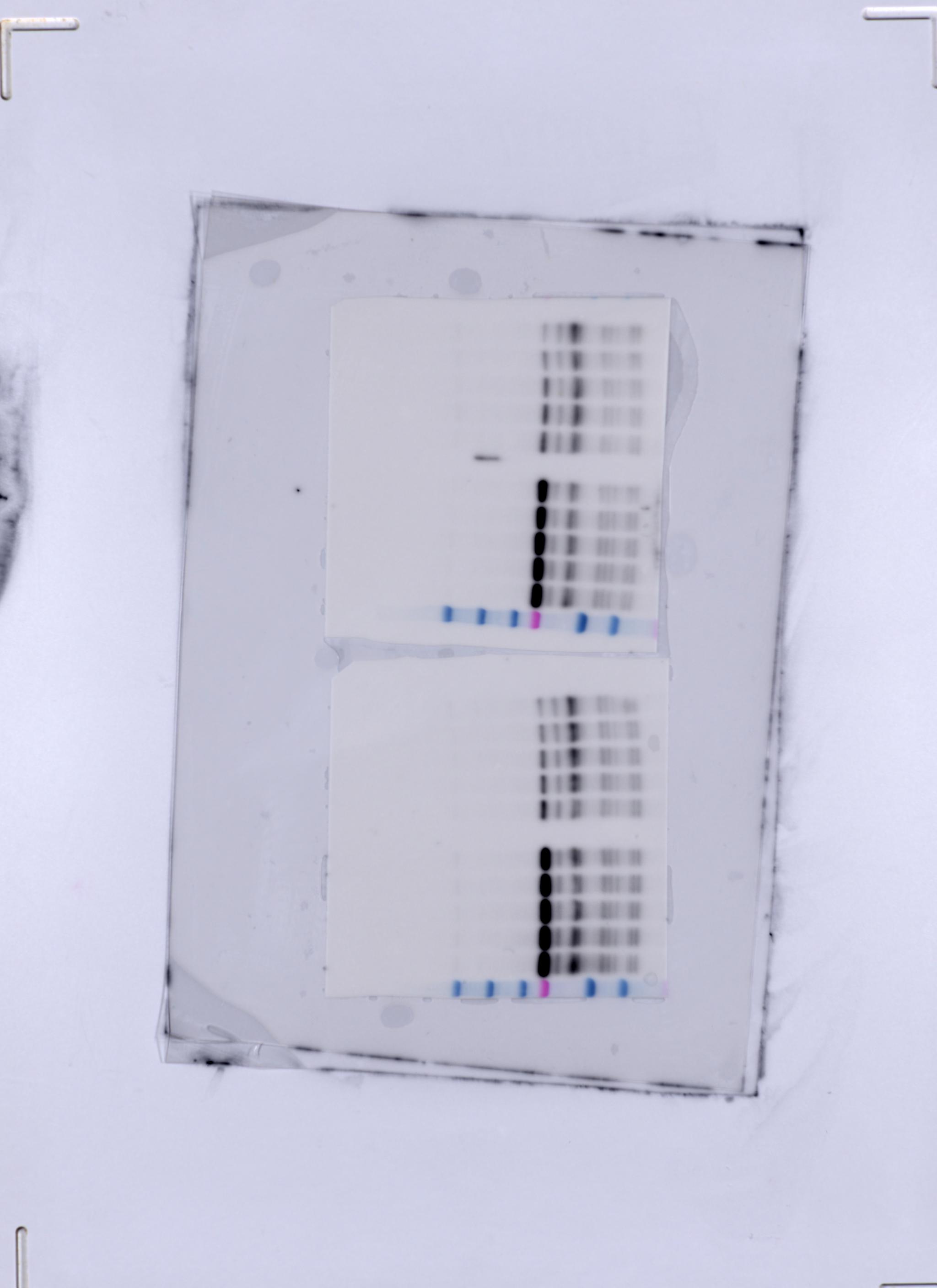

Supplement: Figure 5—source data 2. [file elife-81573-fig5-data2.zip › Figure 5-source data 2/Figure 5-source data 2_raw files/SUN2 end CHX SUN2 2022.04.14_16.16.18_Ch v SUN2/SUN2 end CHX SUN2 2022.04.14_16.16.18_Ch+Marker.jpg]

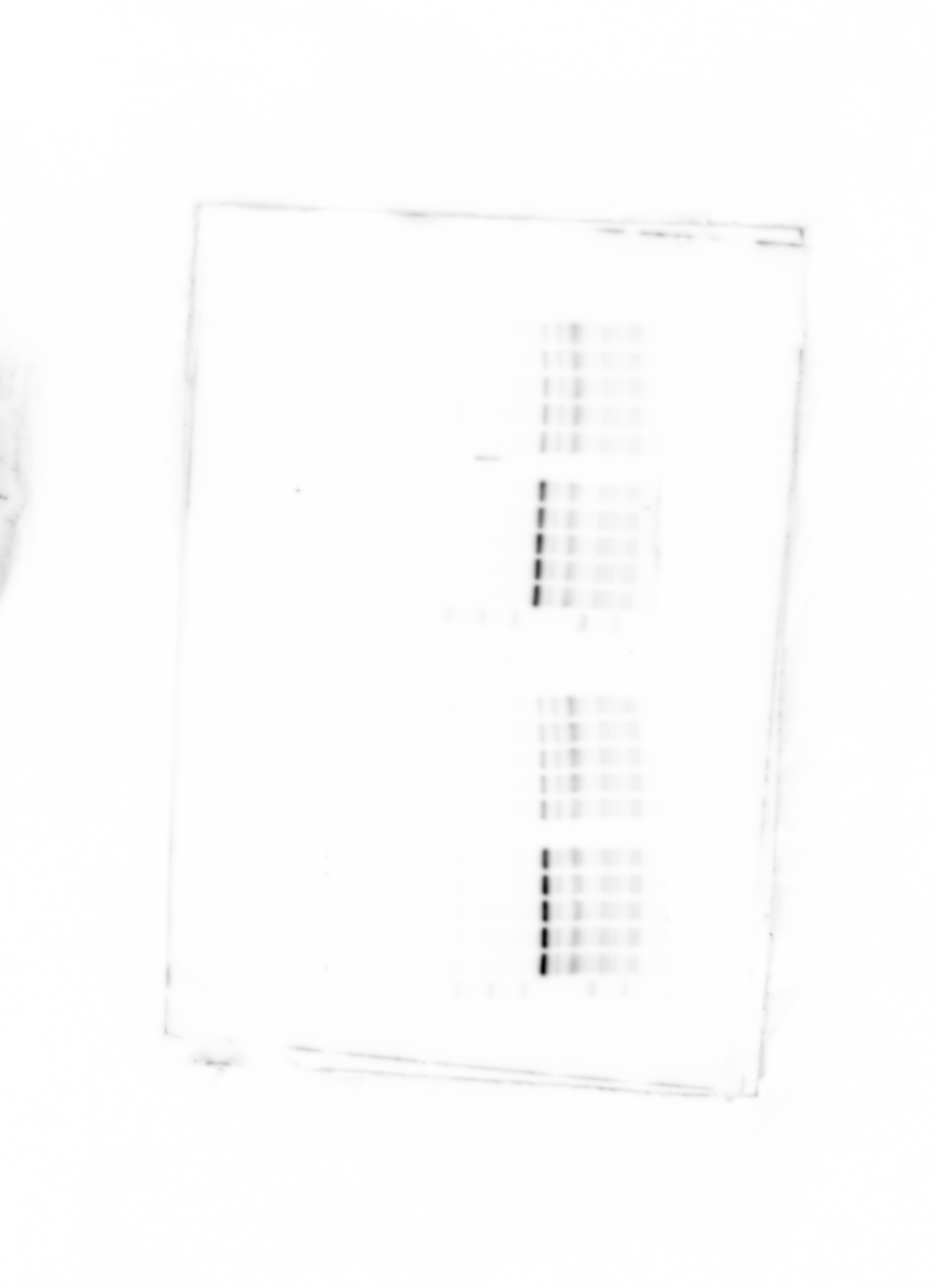

Supplement: Figure 5—source data 2. [file elife-81573-fig5-data2.zip › Figure 5-source data 2/Figure 5-source data 2_raw files/SUN2 end CHX SUN2 2022.04.14_16.16.18_Ch v SUN2/SUN2 end CHX SUN2 2022.04.14_16.16.18_Ch.tif]

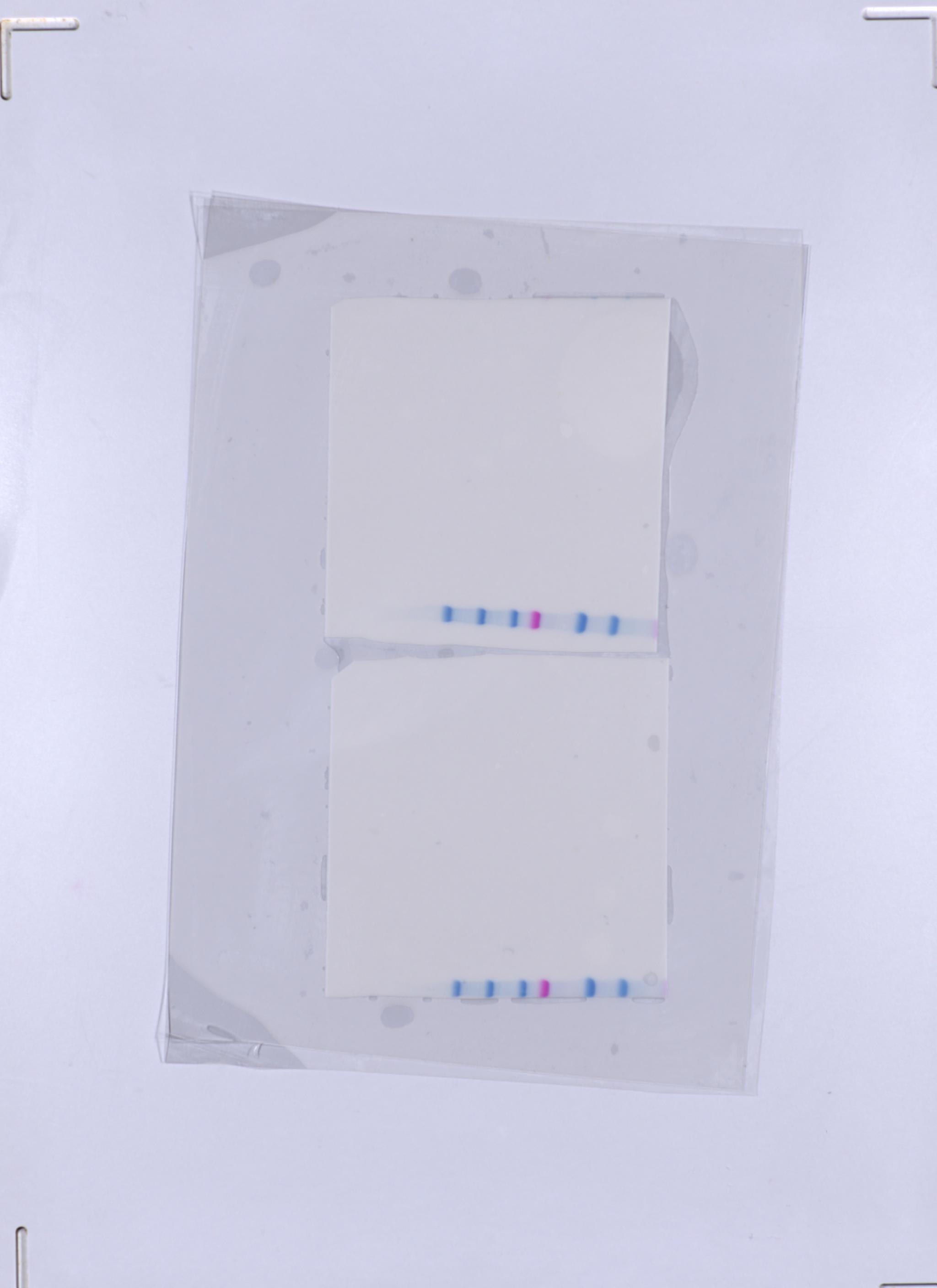

Supplement: Figure 5—source data 2. [file elife-81573-fig5-data2.zip › Figure 5-source data 2/Figure 5-source data 2_raw files/SUN2 end CHX SUN2 2022.04.14_16.16.18_Ch v SUN2/SUN2 end CHX SUN2 2022.04.14_16.16.18_Ch-Marker.jpg]

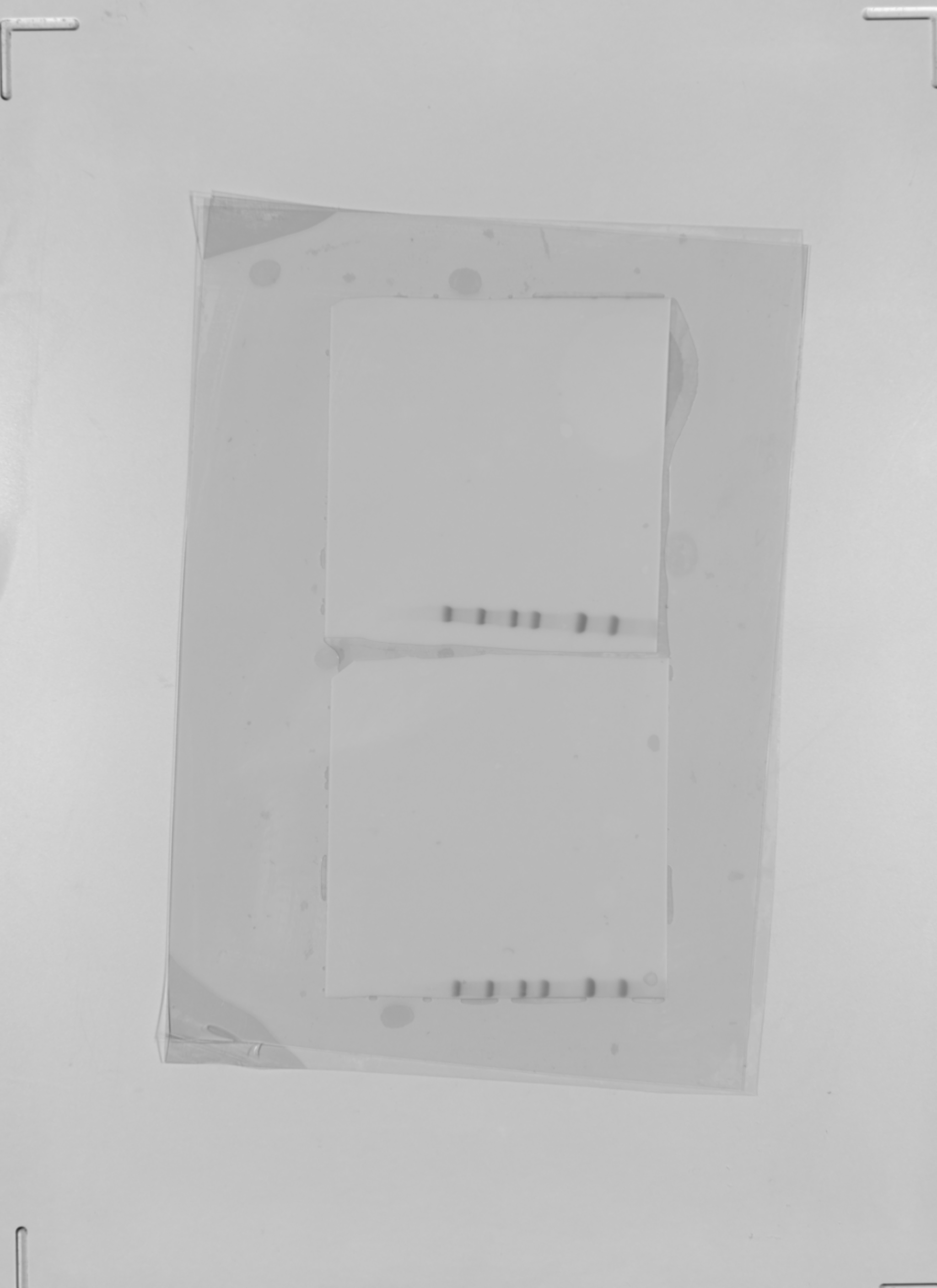

Supplement: Figure 5—source data 2. [file elife-81573-fig5-data2.zip › Figure 5-source data 2/Figure 5-source data 2_raw files/SUN2 end CHX SUN2 2022.04.14_16.16.18_Ch v SUN2/SUN2 end CHX SUN2 2022.04.14_16.16.18_Ch-Marker.tif]

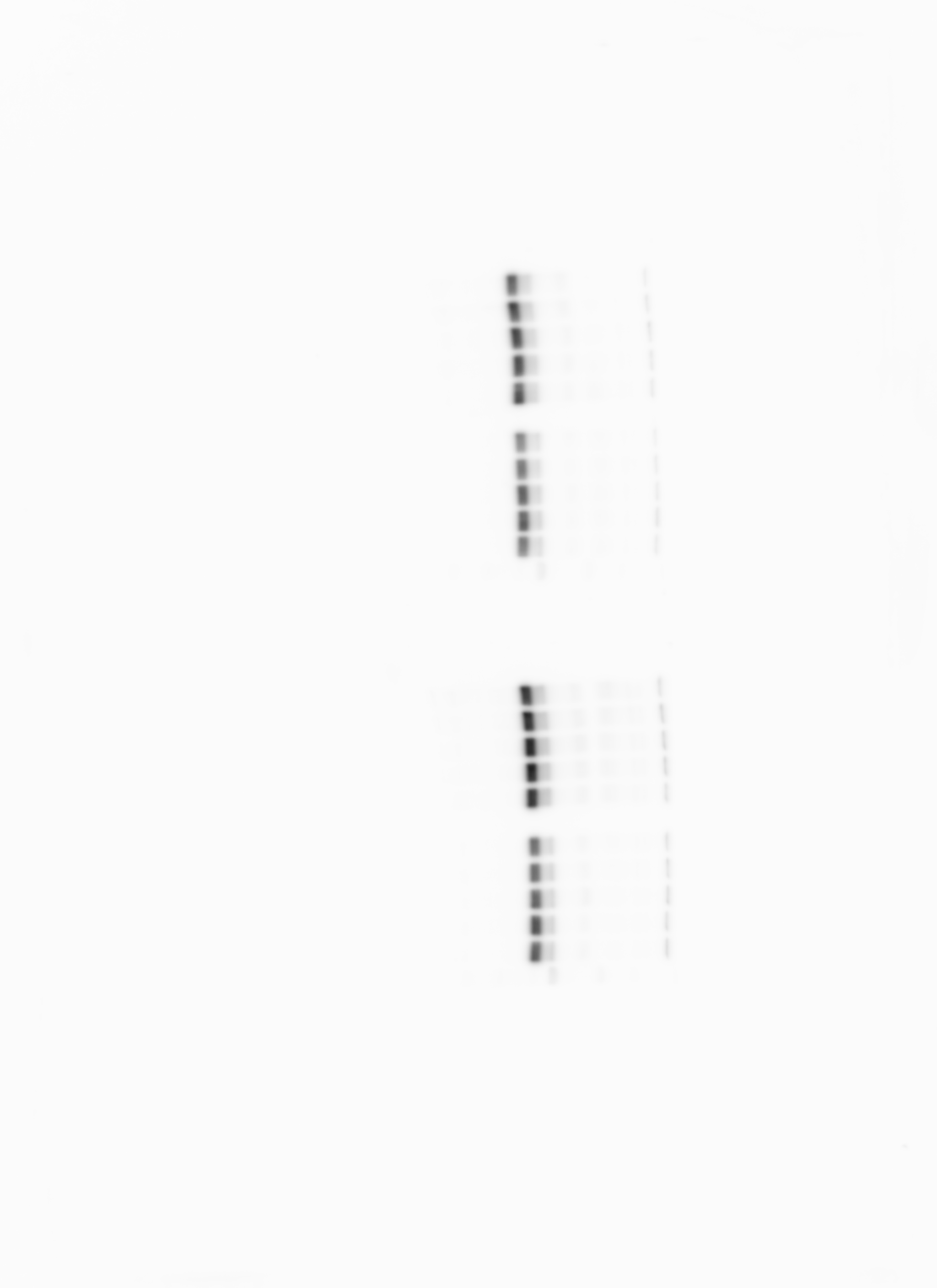

Supplement: Figure 5—source data 2. [file elife-81573-fig5-data2.zip › Figure 5-source data 2/Figure 5-source data 2_raw files/SUN2 end CHX SUN1 2022.04.18_16.44.34-04_Ch v SUN1/SUN2 end CHX SUN1 2022.04.18_16.44.34-04_Ch.tif]

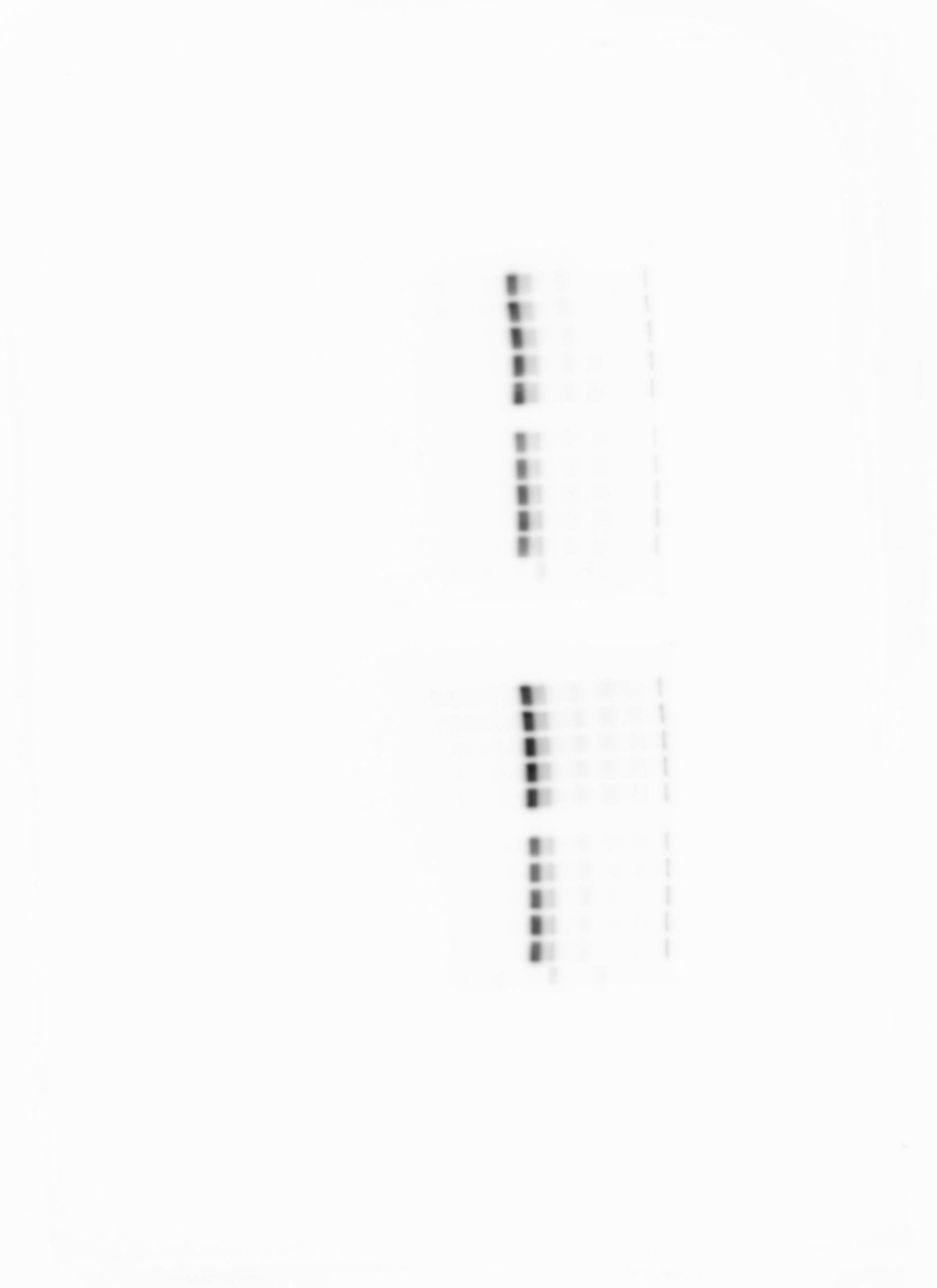

Supplement: Figure 5—source data 2. [file elife-81573-fig5-data2.zip › Figure 5-source data 2/Figure 5-source data 2_raw files/SUN2 end CHX SUN1 2022.04.18_16.44.34-04_Ch v SUN1/SUN2 end CHX SUN1 2022.04.18_16.44.34-04_Ch.jpg]

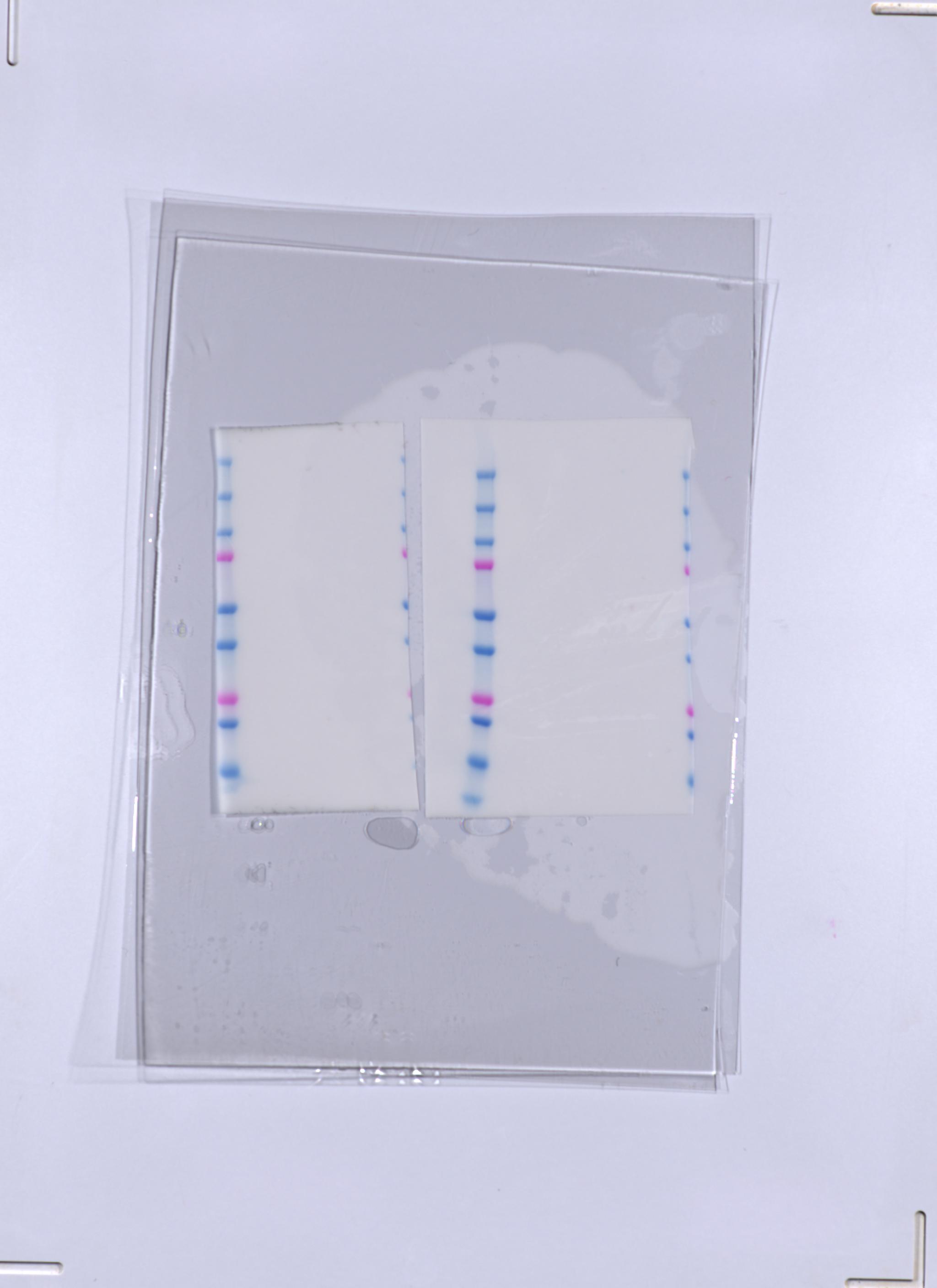

Supplement: Figure 5—source data 3. [file elife-81573-fig5-data3.zip › Figure 5-source data 3/Figure 5-source data 3_raw files/ws,lk ctd-flag FLAG 2022.03.02_11.49.16_Ch-Marker.jpg]

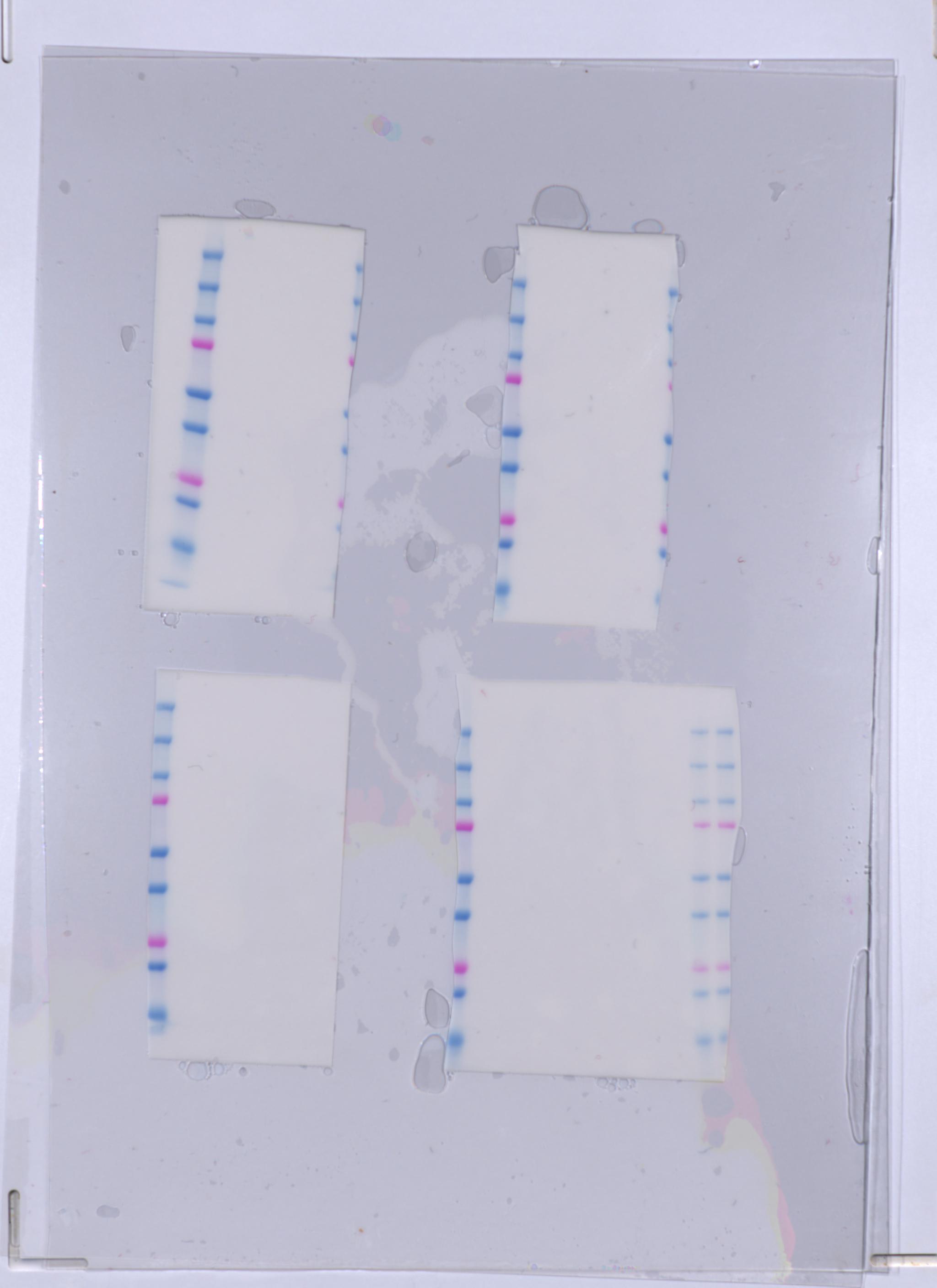

Supplement: Figure 5—source data 3. [file elife-81573-fig5-data3.zip › Figure 5-source data 3/Figure 5-source data 3_raw files/ws,lk ctd-flag SUN2 2022.03.02_11.53.28_Ch-Marker.jpg]

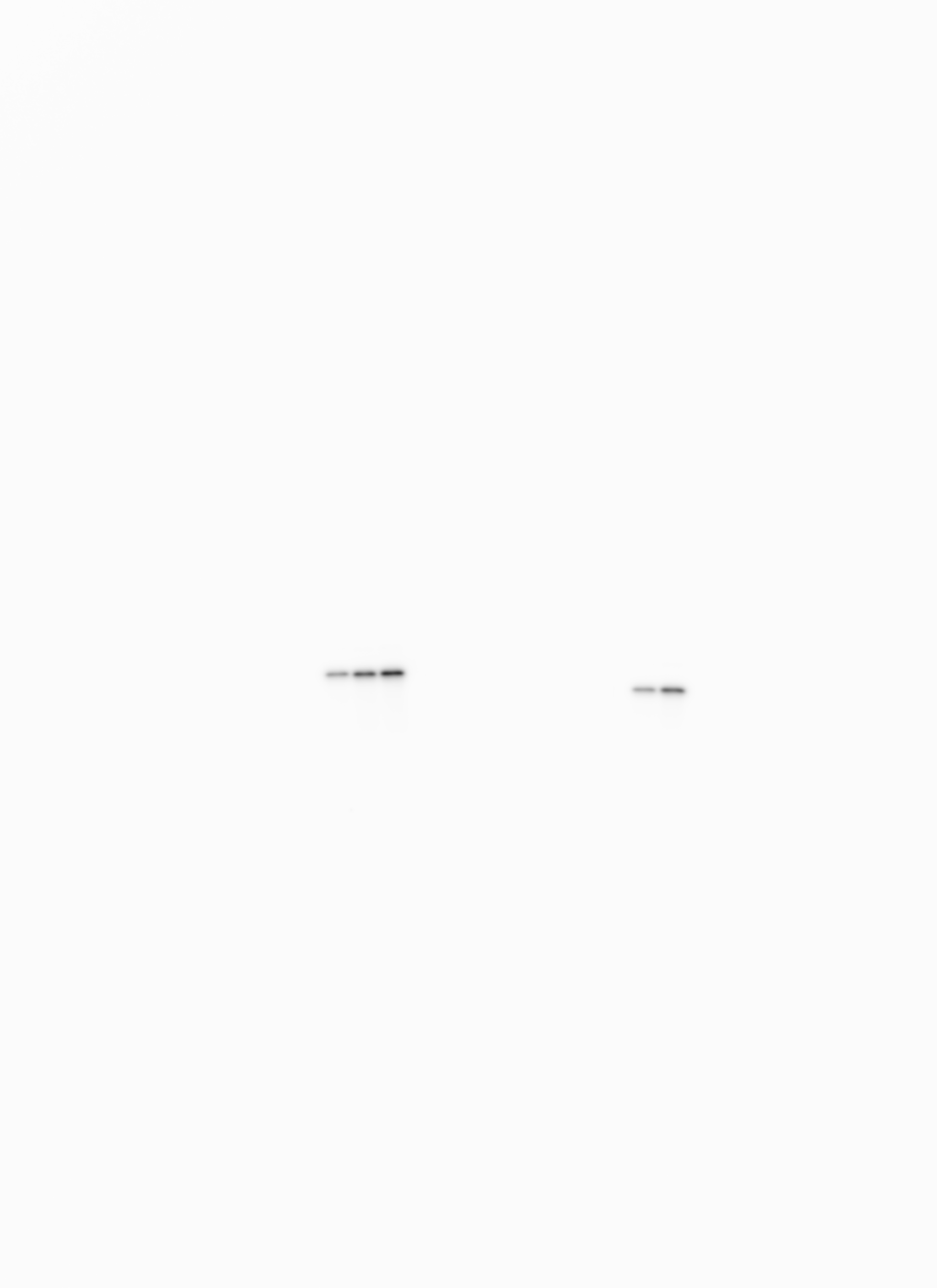

Supplement: Figure 5—source data 3. [file elife-81573-fig5-data3.zip › Figure 5-source data 3/Figure 5-source data 3_raw files/ws,lk ctd-flag FLAG 2022.03.02_11.44.58-03_Ch.tif]

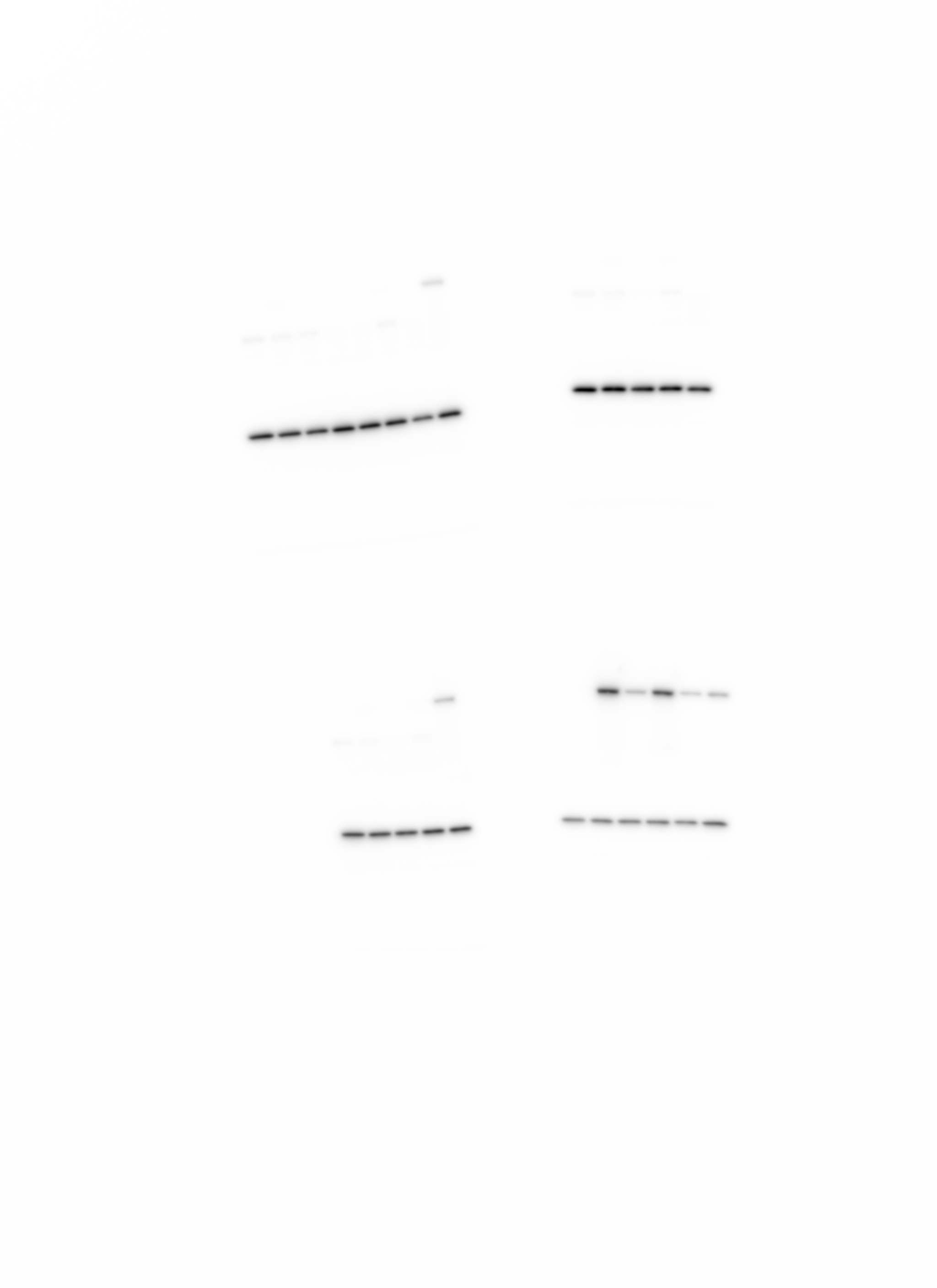

Supplement: Figure 5—source data 3. [file elife-81573-fig5-data3.zip › Figure 5-source data 3/Figure 5-source data 3_raw files/ws,lk ctd-FLAG Gapdh 2022.03.04_12.00.44-03_Ch.tif]

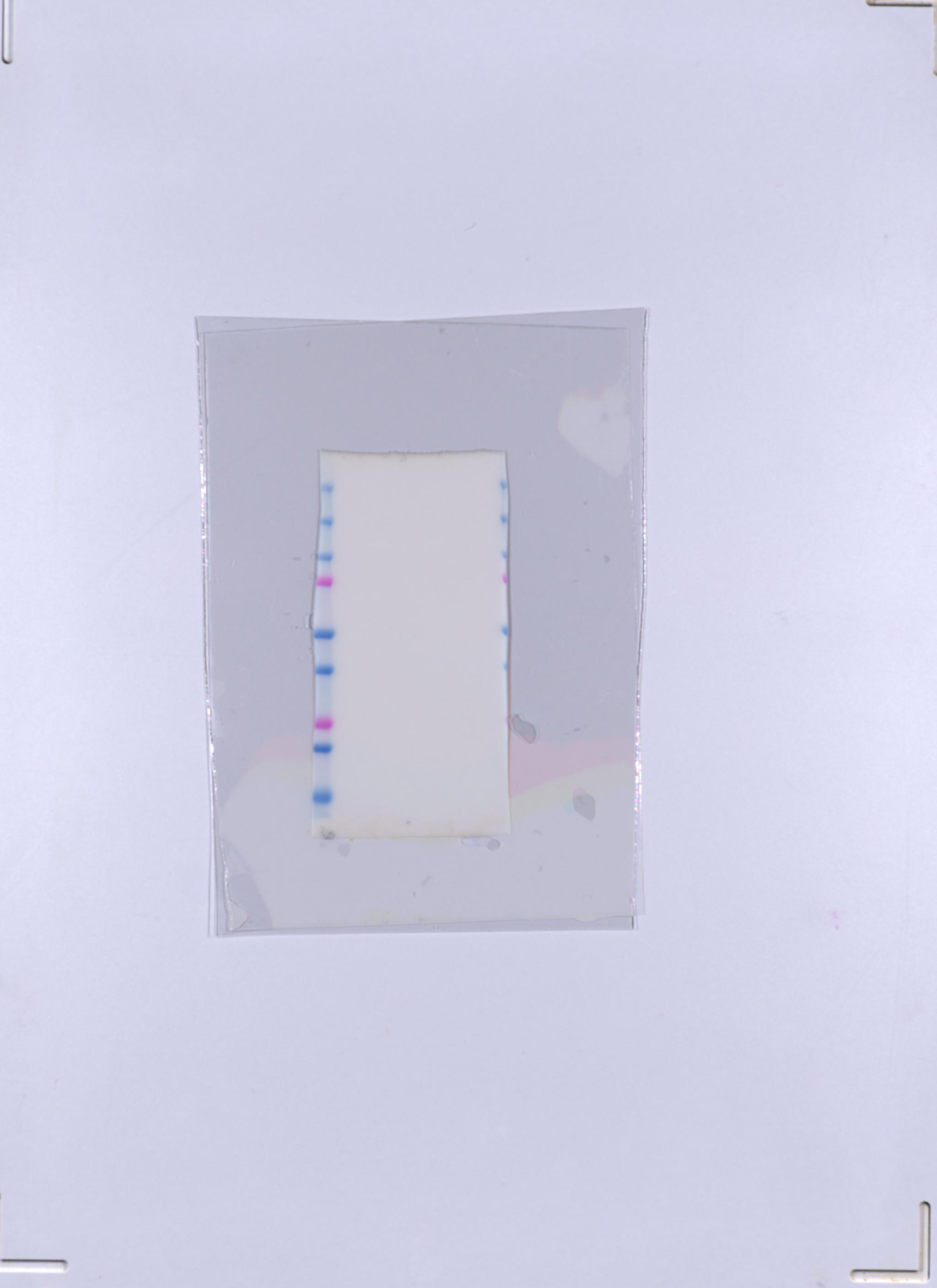

Supplement: Figure 5—source data 3. [file elife-81573-fig5-data3.zip › Figure 5-source data 3/Figure 5-source data 3_raw files/ws,lk ctd-flag HA 2022.03.02_11.35.32_Ch-Marker.jpg]

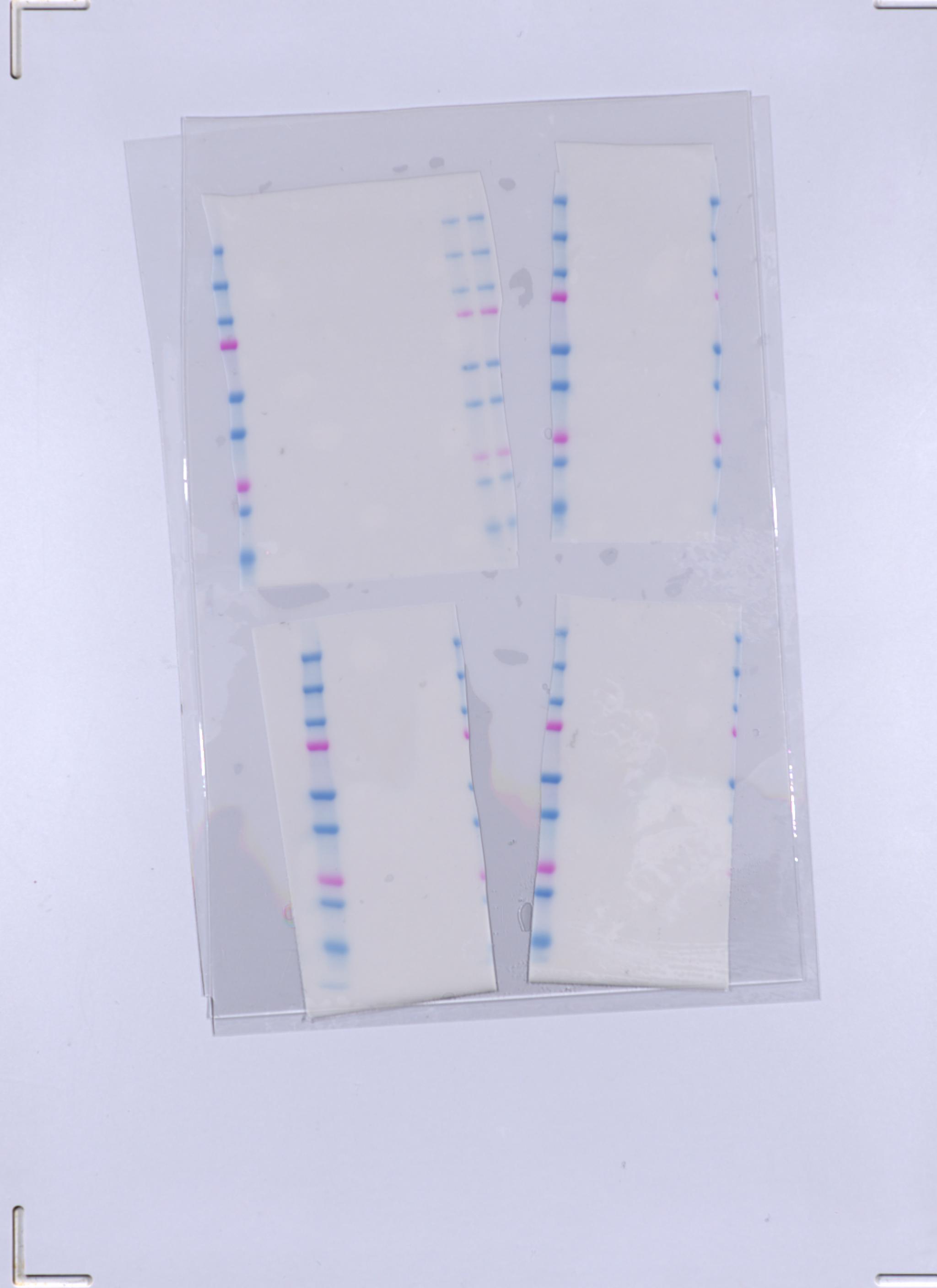

Supplement: Figure 5—source data 3. [file elife-81573-fig5-data3.zip › Figure 5-source data 3/Figure 5-source data 3_raw files/ws,lk ctd-FLAG Gapdh 2022.03.04_12.05.28_Ch-Marker.jpg]

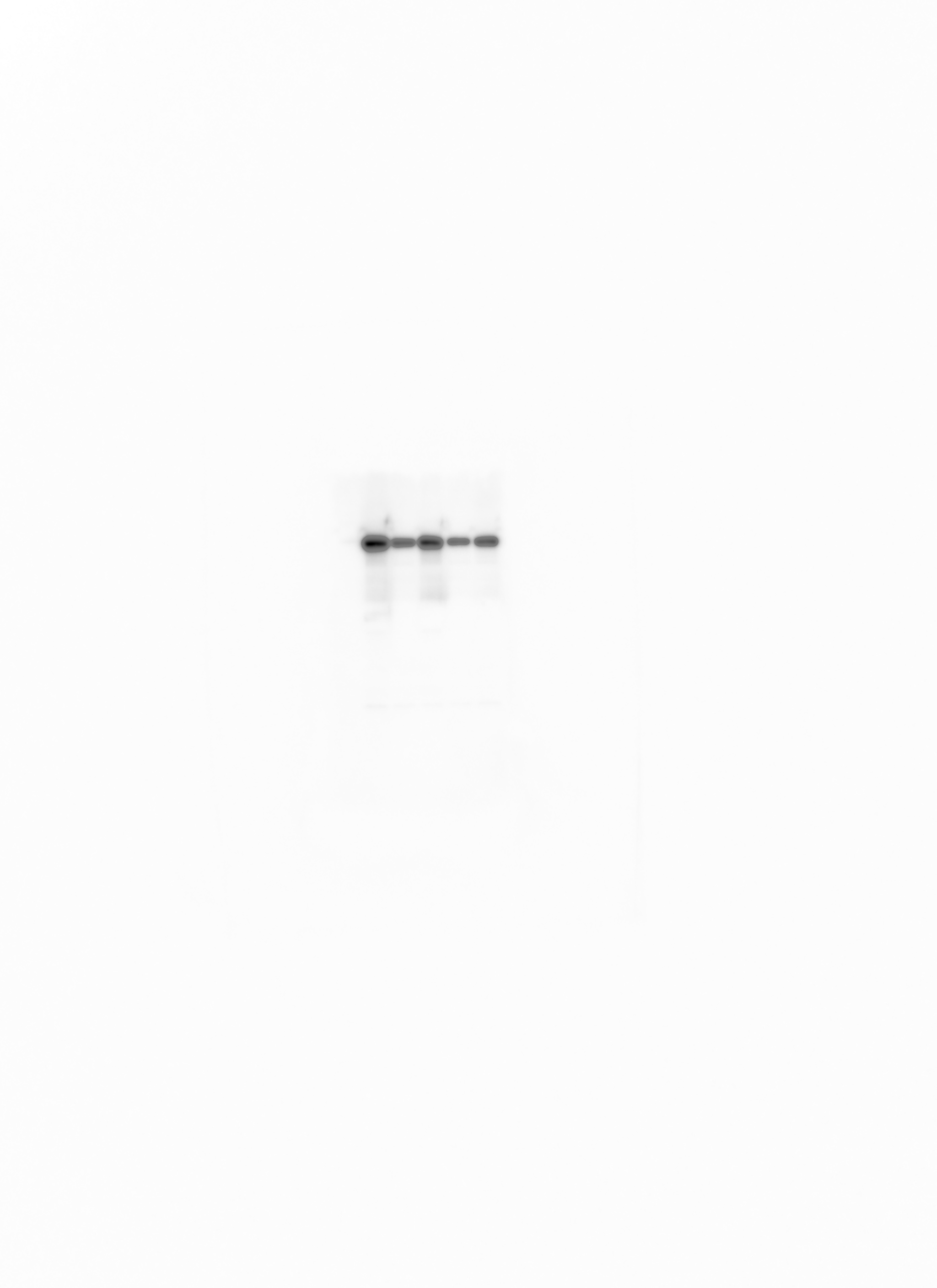

Supplement: Figure 5—source data 3. [file elife-81573-fig5-data3.zip › Figure 5-source data 3/Figure 5-source data 3_raw files/ws,lk ctd-flag HA 2022.03.02_11.38.22-03_Ch.tif]

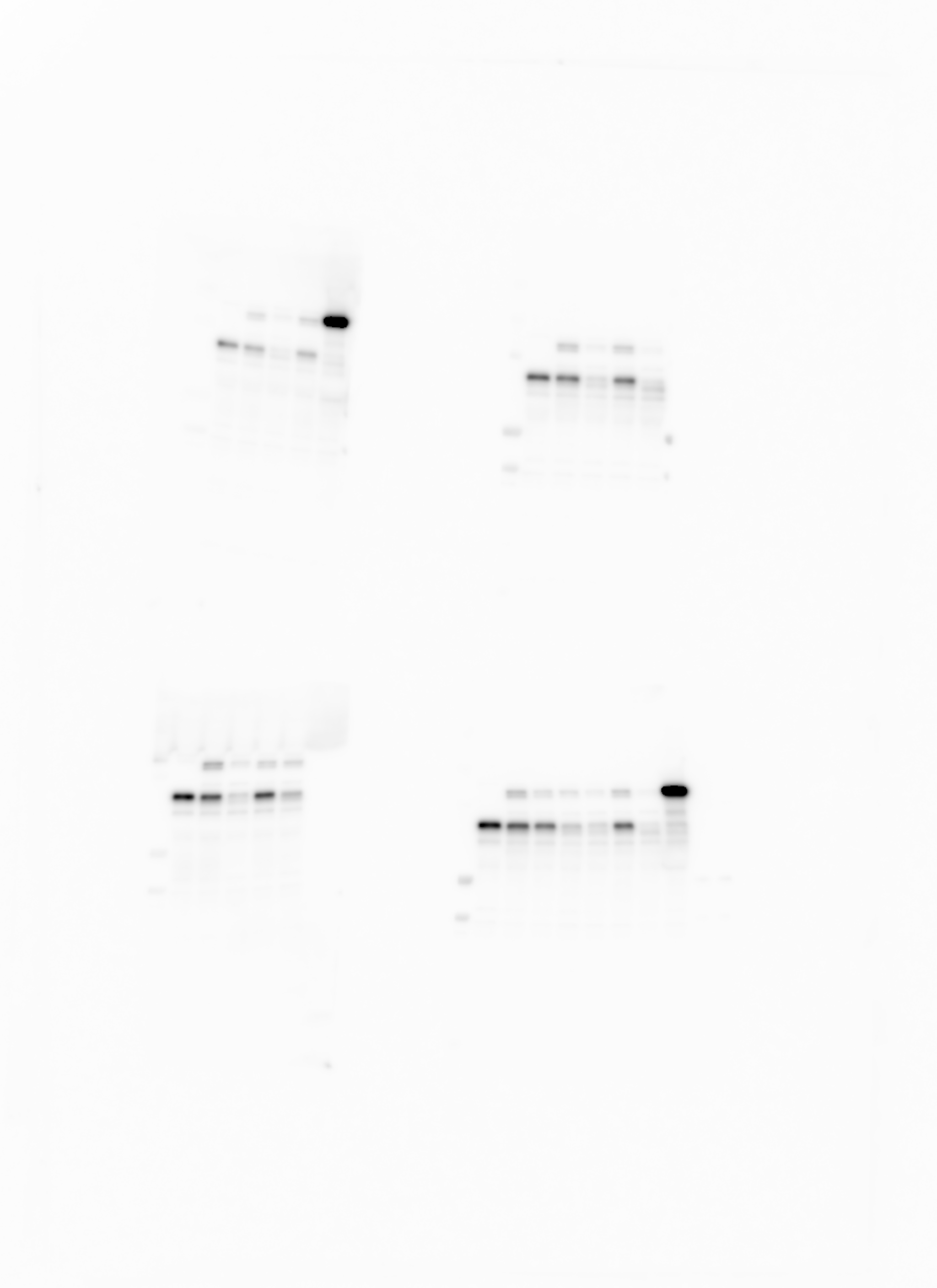

Supplement: Figure 5—source data 3. [file elife-81573-fig5-data3.zip › Figure 5-source data 3/Figure 5-source data 3_raw files/ws,lk ctd-flag SUN2 2022.03.02_11.55.47-10_Ch.tif]

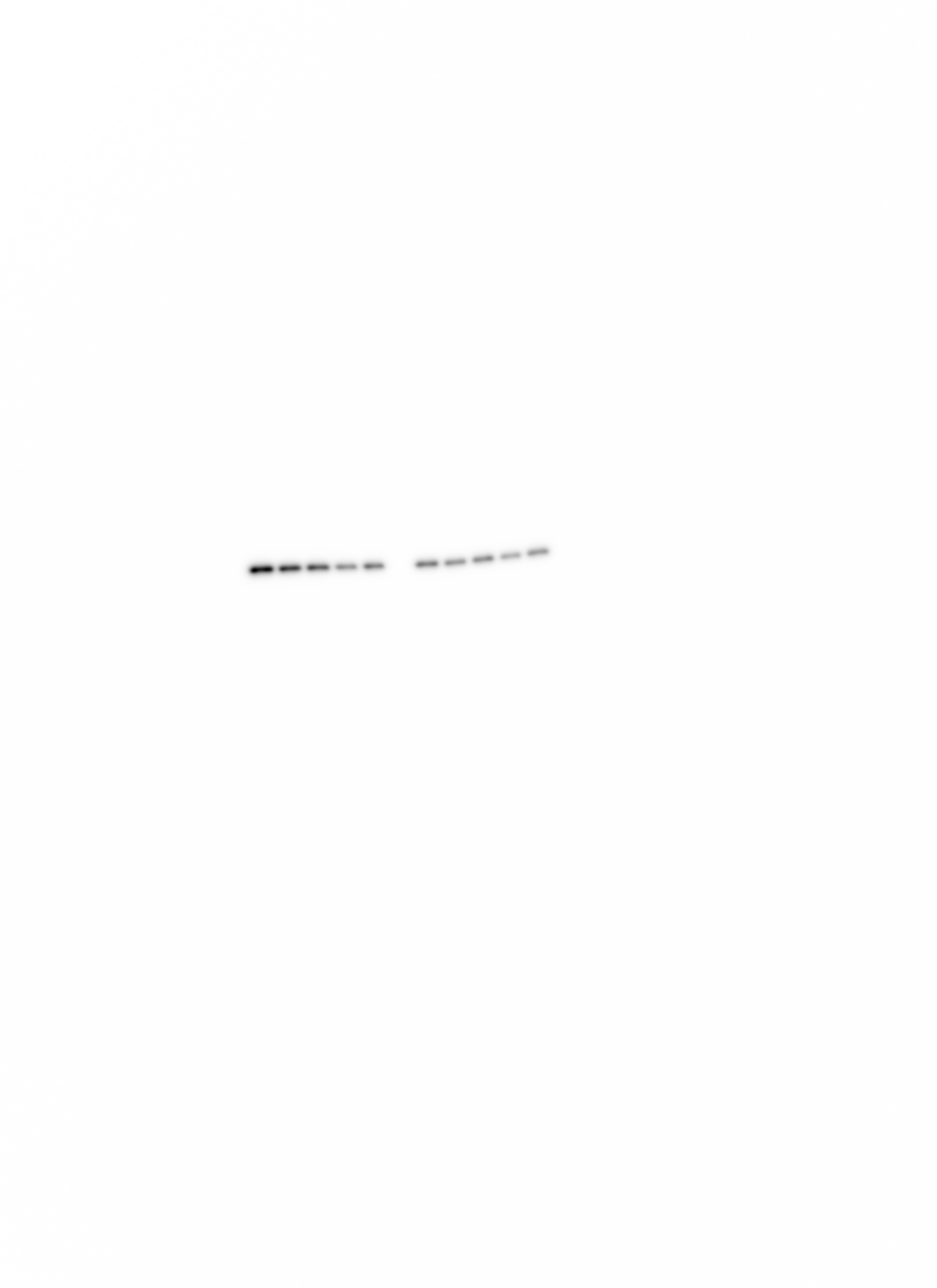

Supplement: Figure 5—source data 4. [file elife-81573-fig5-data4.zip › Figure 5-source data 4/Figure 5-source data 4_raw files/LK220707 Fig5G Gpdh 2022.07.07_22.31.41_Ch/LK220707 Fig5G Gpdh 2022.07.07_22.31.41_Ch.tif]

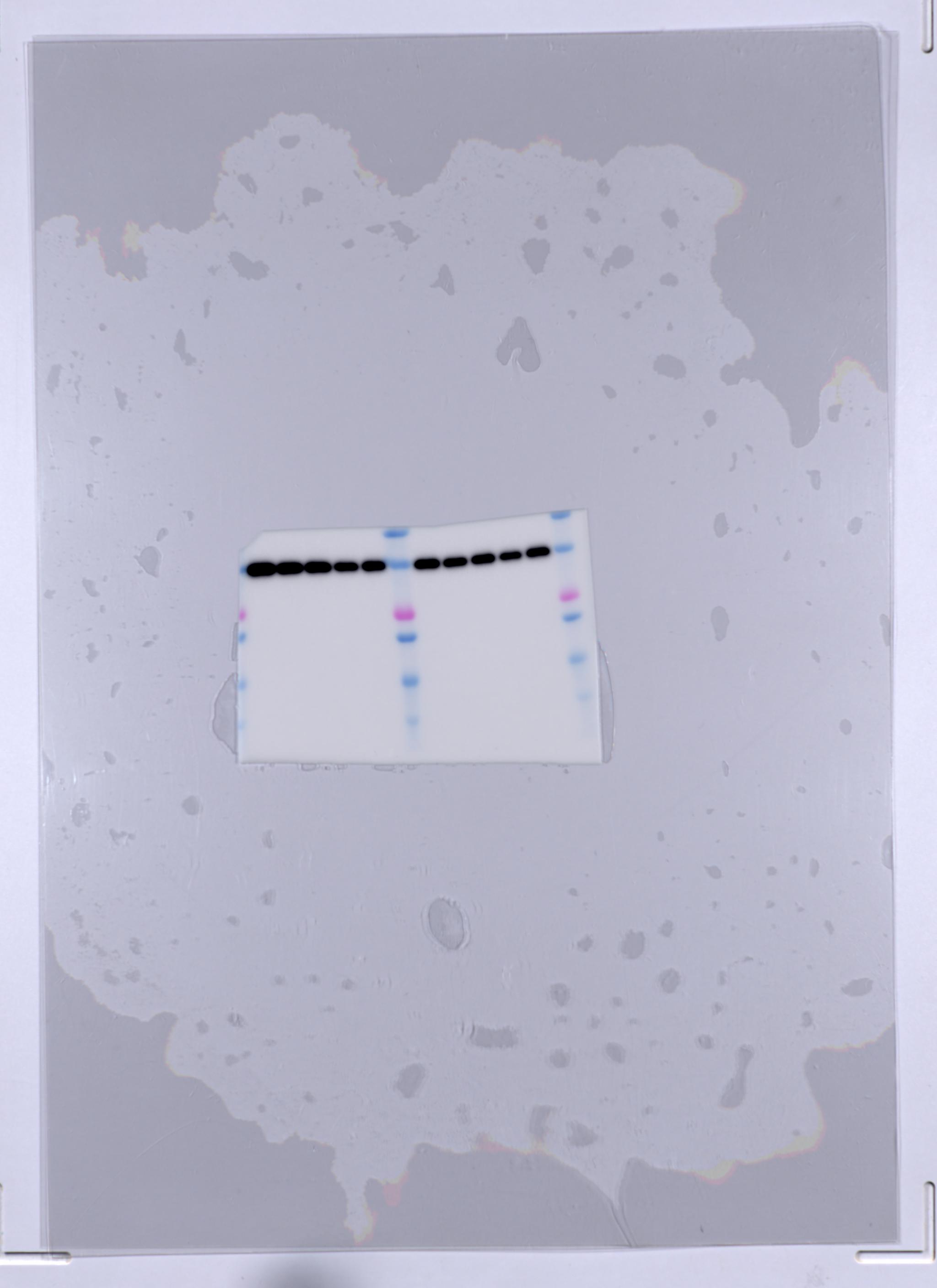

Supplement: Figure 5—source data 4. [file elife-81573-fig5-data4.zip › Figure 5-source data 4/Figure 5-source data 4_raw files/LK220707 Fig5G Gpdh 2022.07.07_22.31.41_Ch/LK220707 Fig5G Gpdh 2022.07.07_22.31.41_Ch+Marker.jpg]

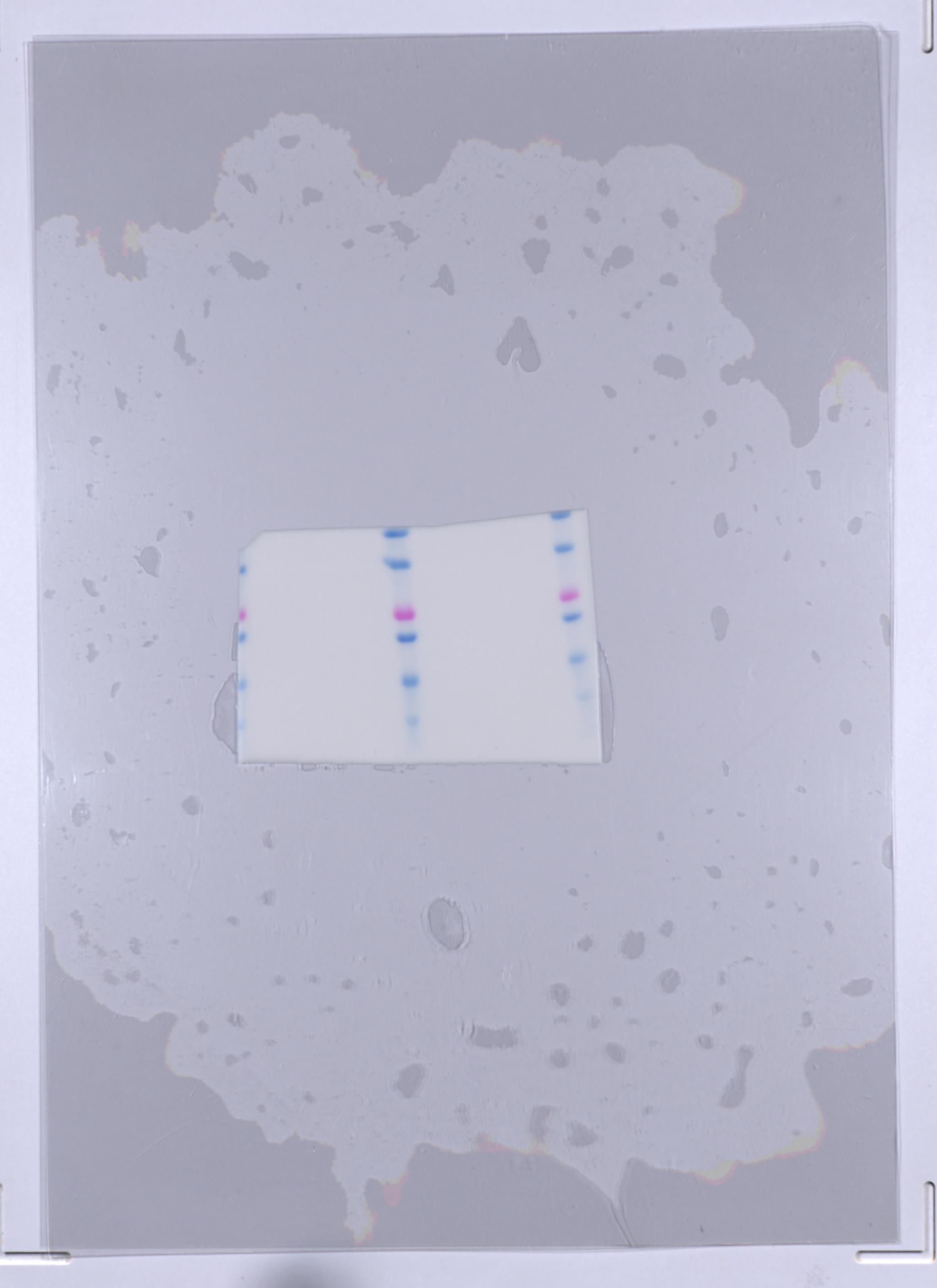

Supplement: Figure 5—source data 4. [file elife-81573-fig5-data4.zip › Figure 5-source data 4/Figure 5-source data 4_raw files/LK220707 Fig5G Gpdh 2022.07.07_22.31.41_Ch/LK220707 Fig5G Gpdh 2022.07.07_22.31.41_Ch-Marker.jpg]

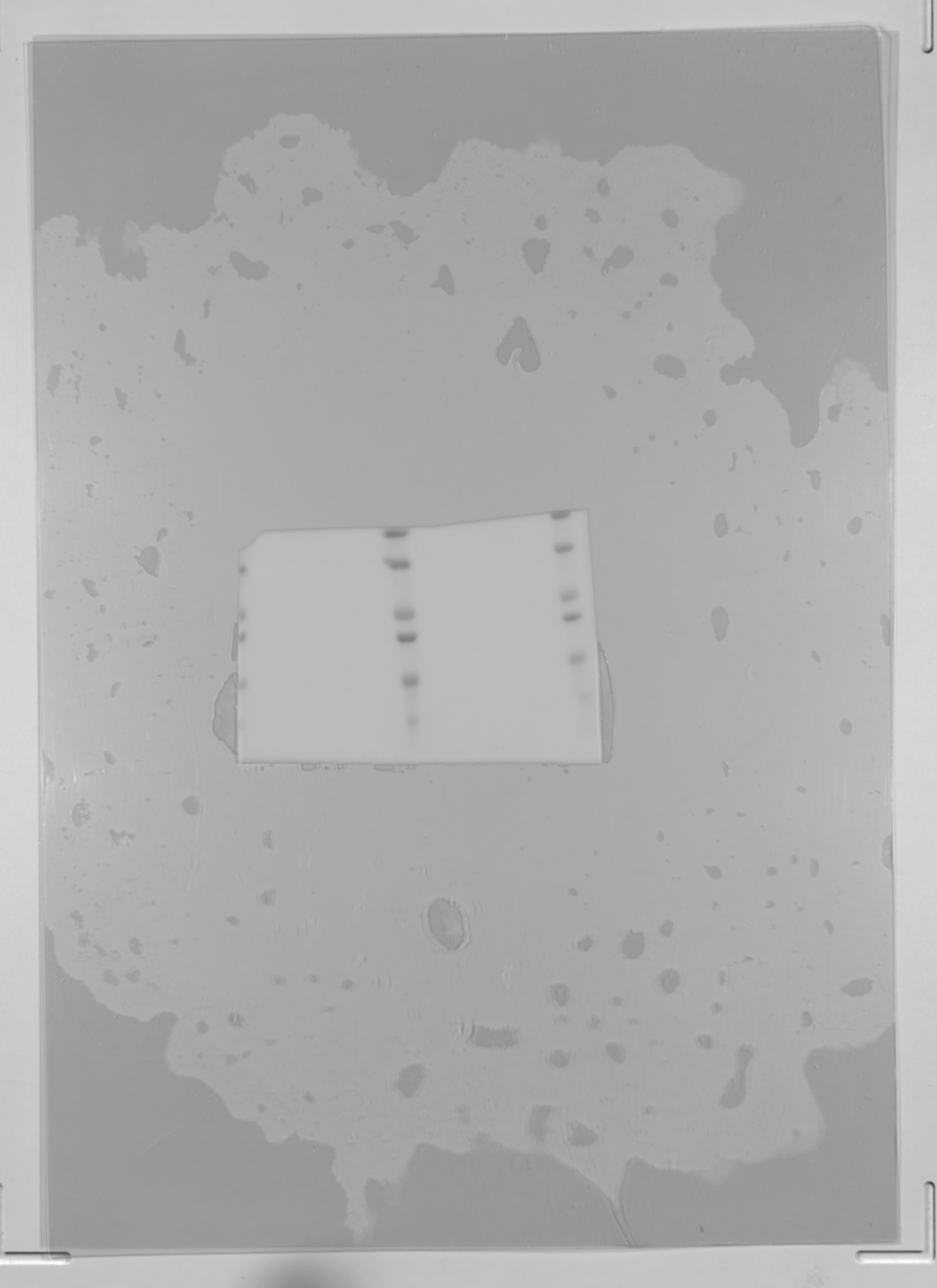

Supplement: Figure 5—source data 4. [file elife-81573-fig5-data4.zip › Figure 5-source data 4/Figure 5-source data 4_raw files/LK220707 Fig5G Gpdh 2022.07.07_22.31.41_Ch/LK220707 Fig5G Gpdh 2022.07.07_22.31.41_Ch-Marker.tif]
