# Supplementary figures and images for: Regulated degradation of the inner nuclear membrane protein SUN2 maintains nuclear envelope architecture and function (part 2 of 2)
Source: eLife. 2022 Nov 1;11:e81573. doi: 10.7554/eLife.81573 (PMC9662817; doi:10.7554/eLife.81573)

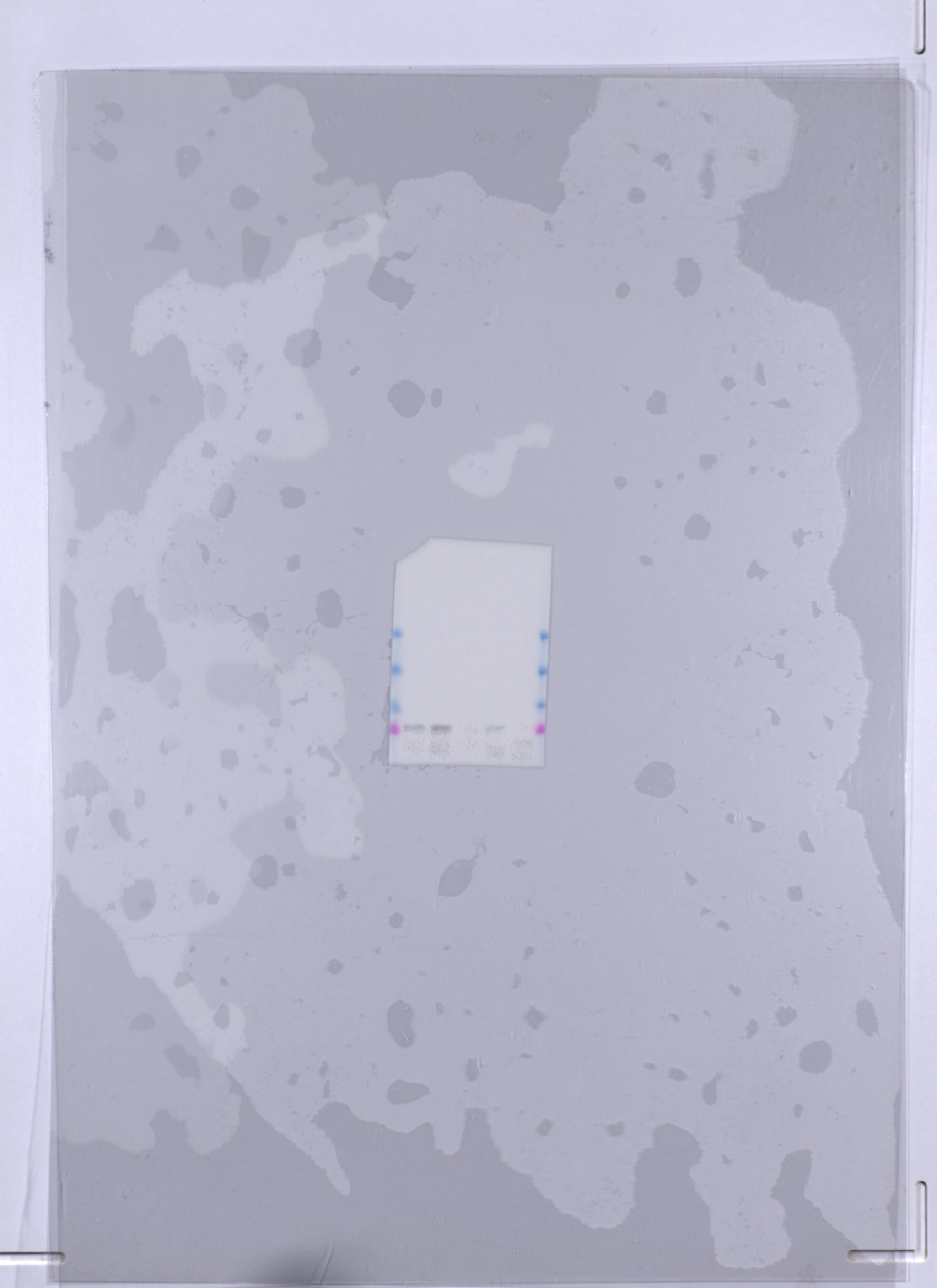

Supplement: Figure 5—source data 4. [file elife-81573-fig5-data4.zip › Figure 5-source data 4/Figure 5-source data 4_raw files/LK220707 Fig5G-2 Sun 2022.07.07_23.56.01_Ch/LK220707 Fig5G-2 Sun 2022.07.07_23.56.01_Ch+Marker.jpg]

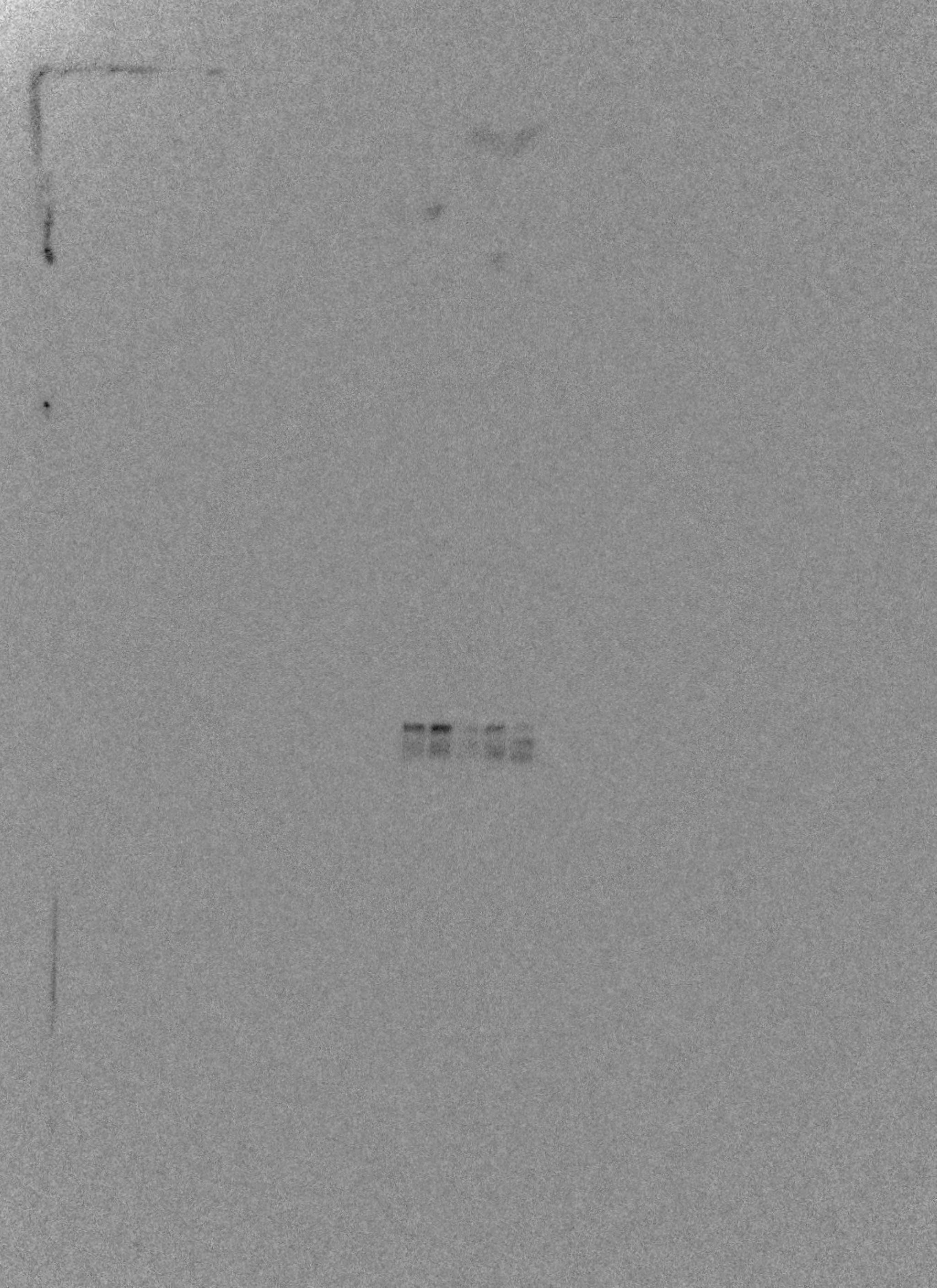

Supplement: Figure 5—source data 4. [file elife-81573-fig5-data4.zip › Figure 5-source data 4/Figure 5-source data 4_raw files/LK220707 Fig5G-2 Sun 2022.07.07_23.56.01_Ch/LK220707 Fig5G-2 Sun 2022.07.07_23.56.01_Ch.tif]

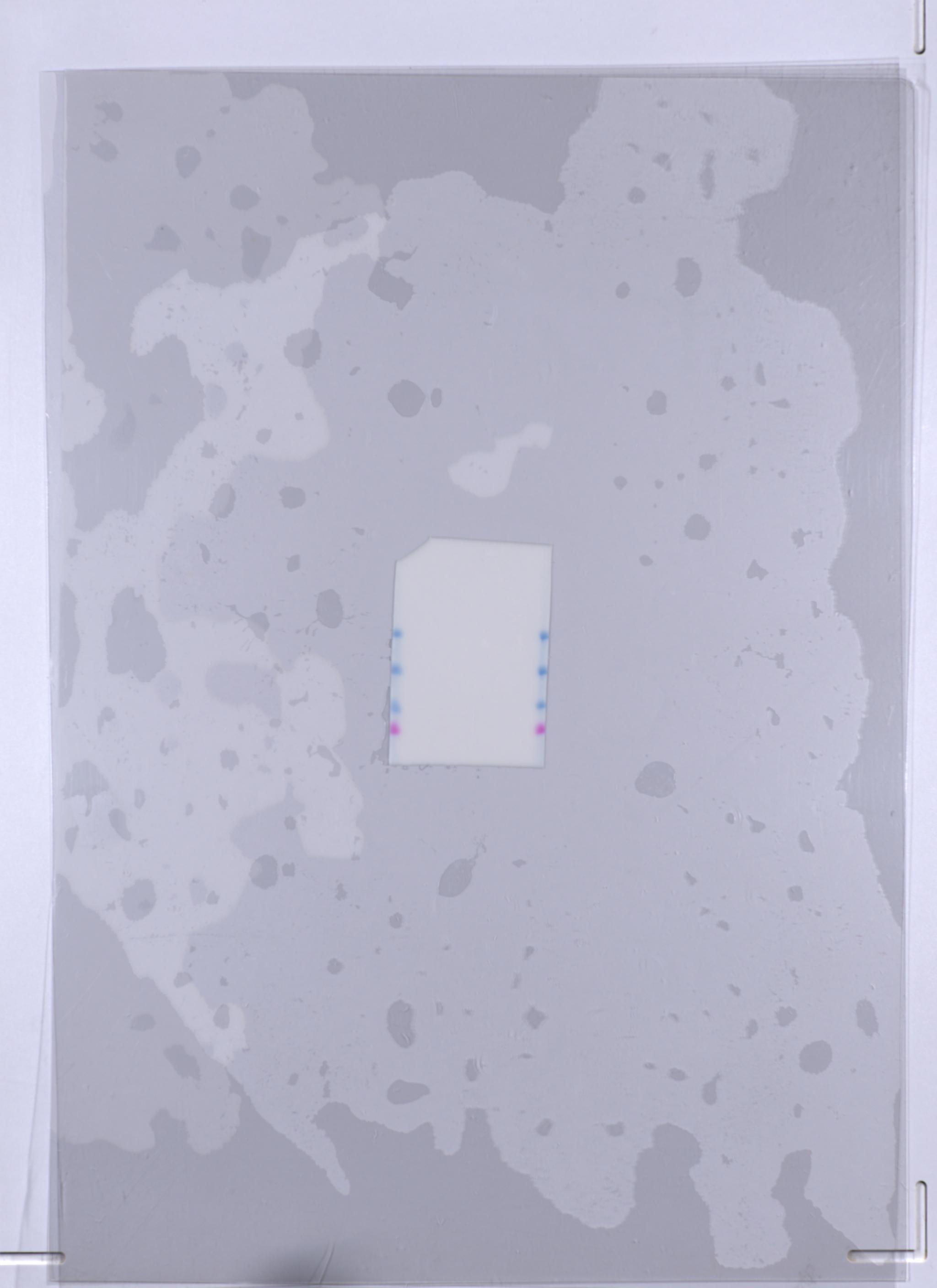

Supplement: Figure 5—source data 4. [file elife-81573-fig5-data4.zip › Figure 5-source data 4/Figure 5-source data 4_raw files/LK220707 Fig5G-2 Sun 2022.07.07_23.56.01_Ch/LK220707 Fig5G-2 Sun 2022.07.07_23.56.01_Ch-Marker.jpg]

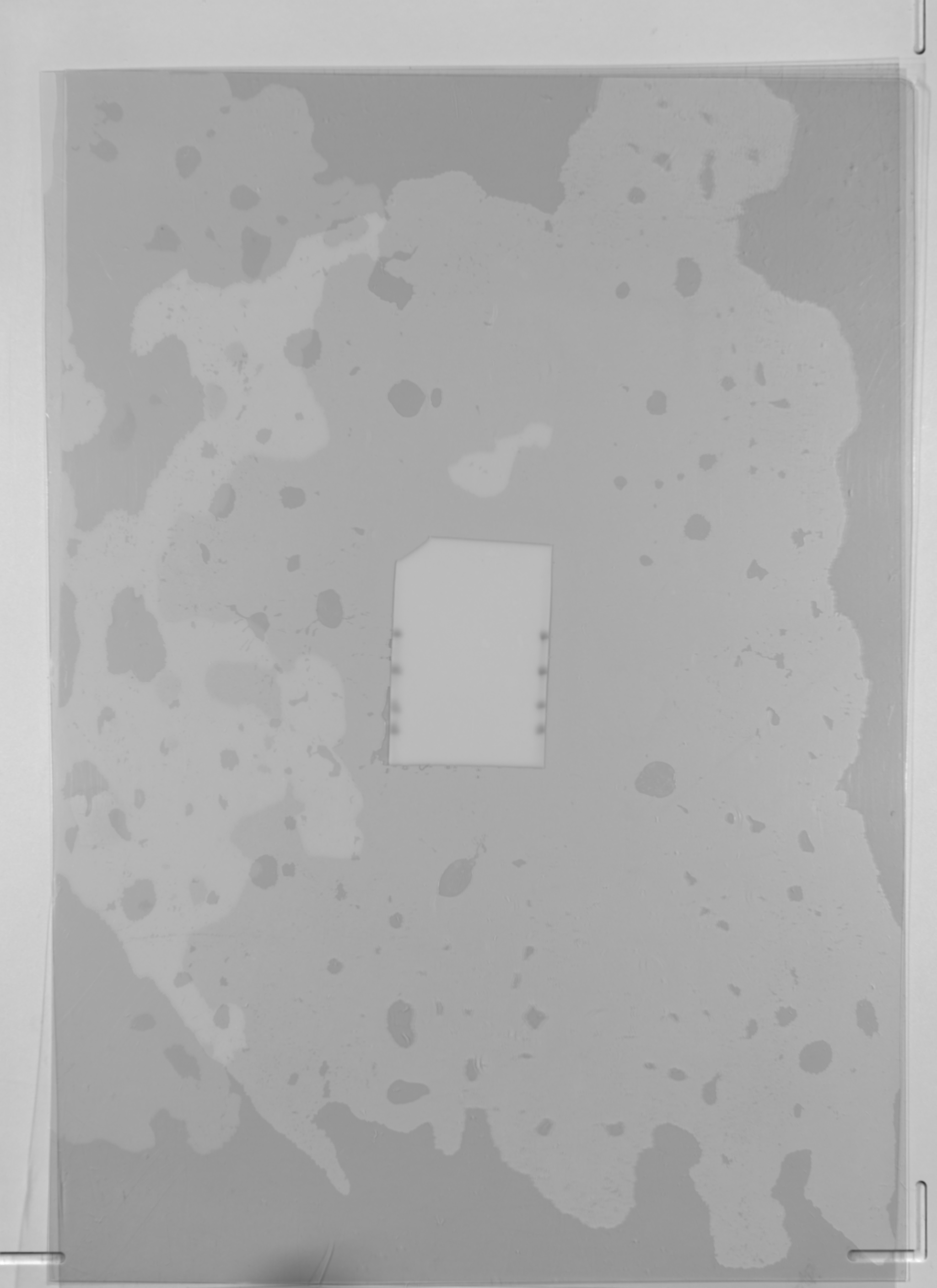

Supplement: Figure 5—source data 4. [file elife-81573-fig5-data4.zip › Figure 5-source data 4/Figure 5-source data 4_raw files/LK220707 Fig5G-2 Sun 2022.07.07_23.56.01_Ch/LK220707 Fig5G-2 Sun 2022.07.07_23.56.01_Ch-Marker.tif]

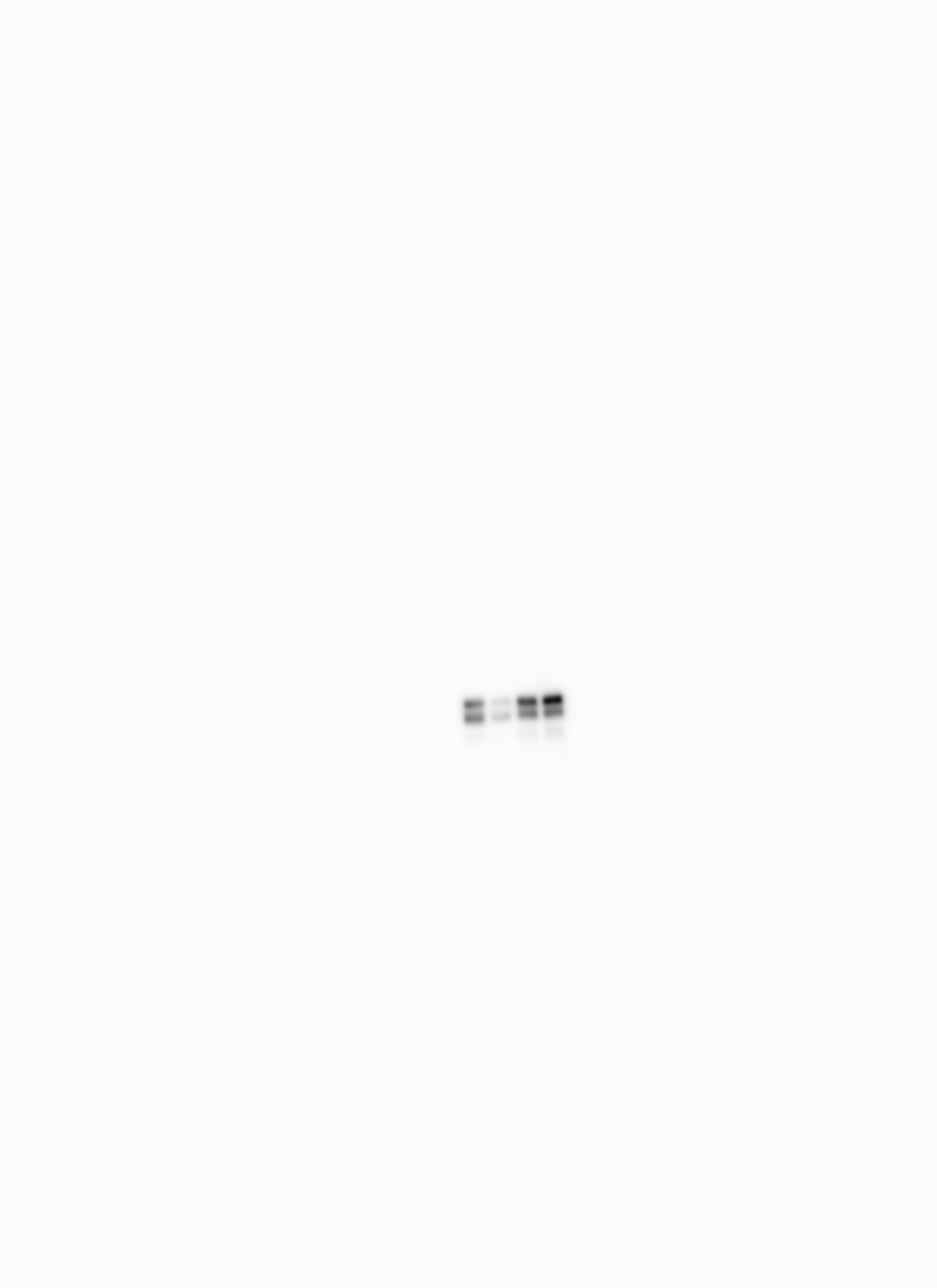

Supplement: Figure 5—source data 4. [file elife-81573-fig5-data4.zip › Figure 5-source data 4/Figure 5-source data 4_raw files/LK220707 Fig5G HA 2022.07.07_22.36.55-03_Ch/LK220707 Fig5G HA 2022.07.07_22.36.55-03_Ch.jpg]

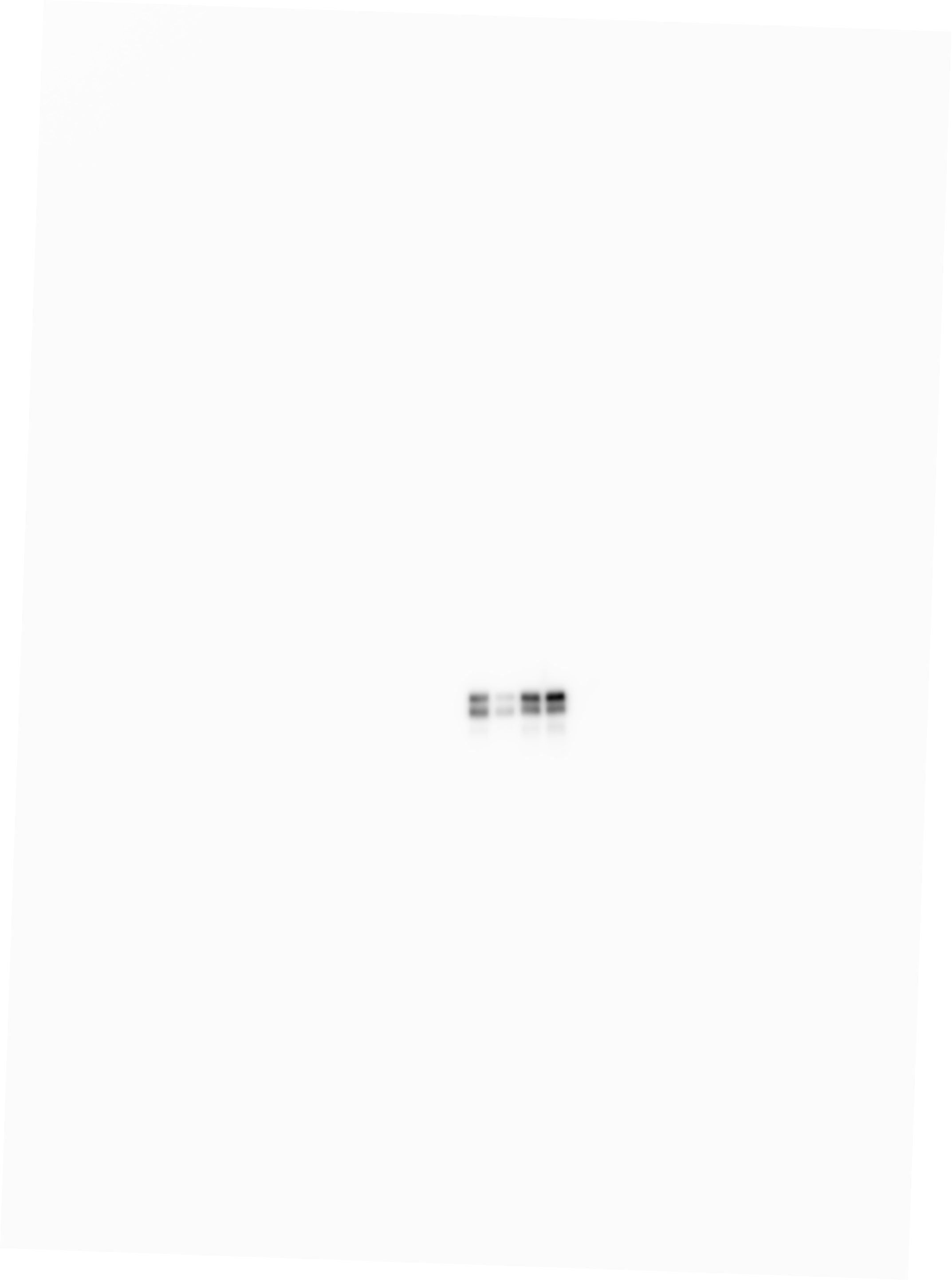

Supplement: Figure 5—source data 4. [file elife-81573-fig5-data4.zip › Figure 5-source data 4/Figure 5-source data 4_raw files/LK220707 Fig5G HA 2022.07.07_22.36.55-03_Ch/LK220707 Fig5G HA 2022.07.07_22.36.55-03_Ch_rotated.tif]

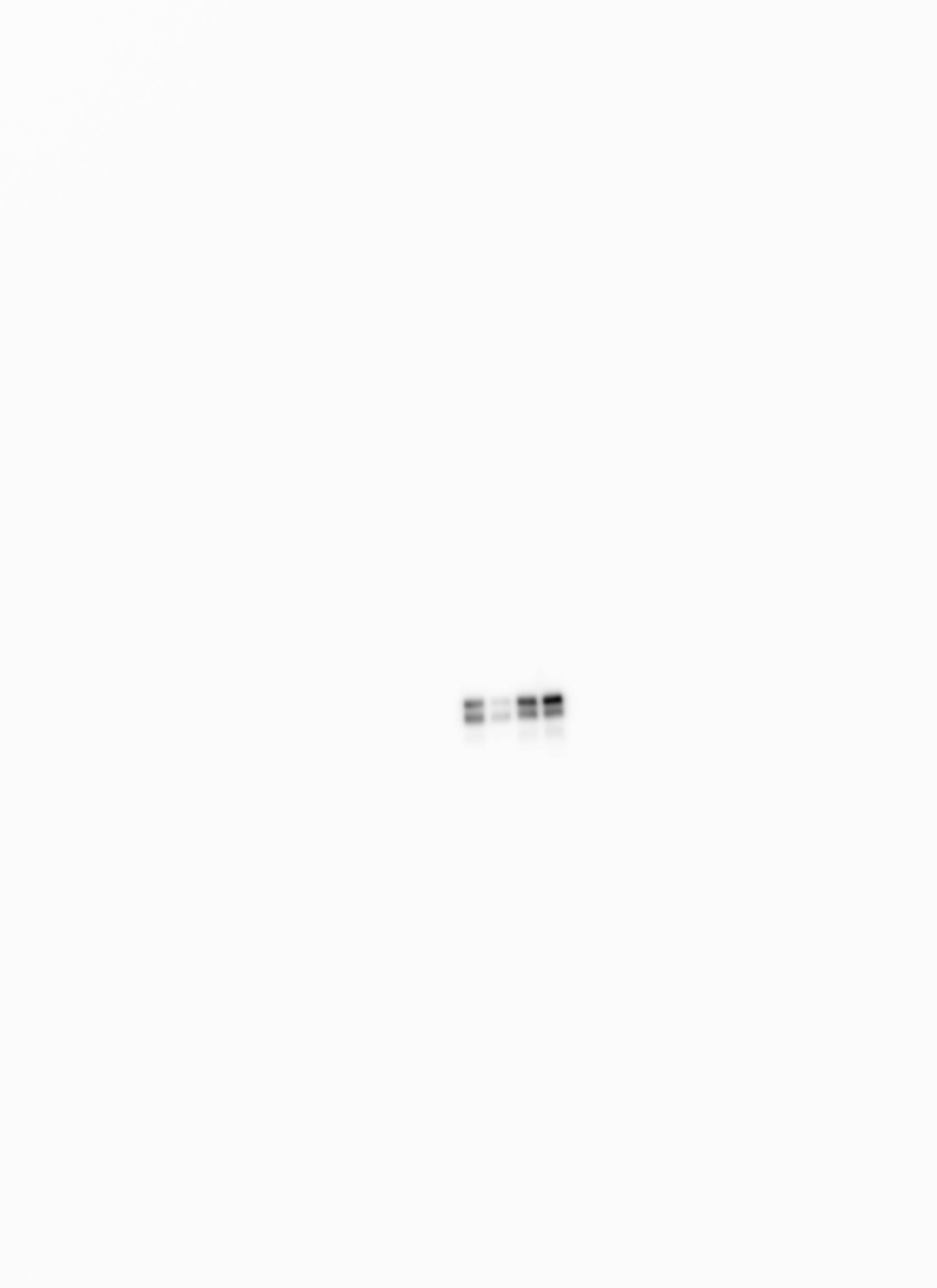

Supplement: Figure 5—source data 4. [file elife-81573-fig5-data4.zip › Figure 5-source data 4/Figure 5-source data 4_raw files/LK220707 Fig5G HA 2022.07.07_22.36.55-03_Ch/LK220707 Fig5G HA 2022.07.07_22.36.55-03_Ch.tif]

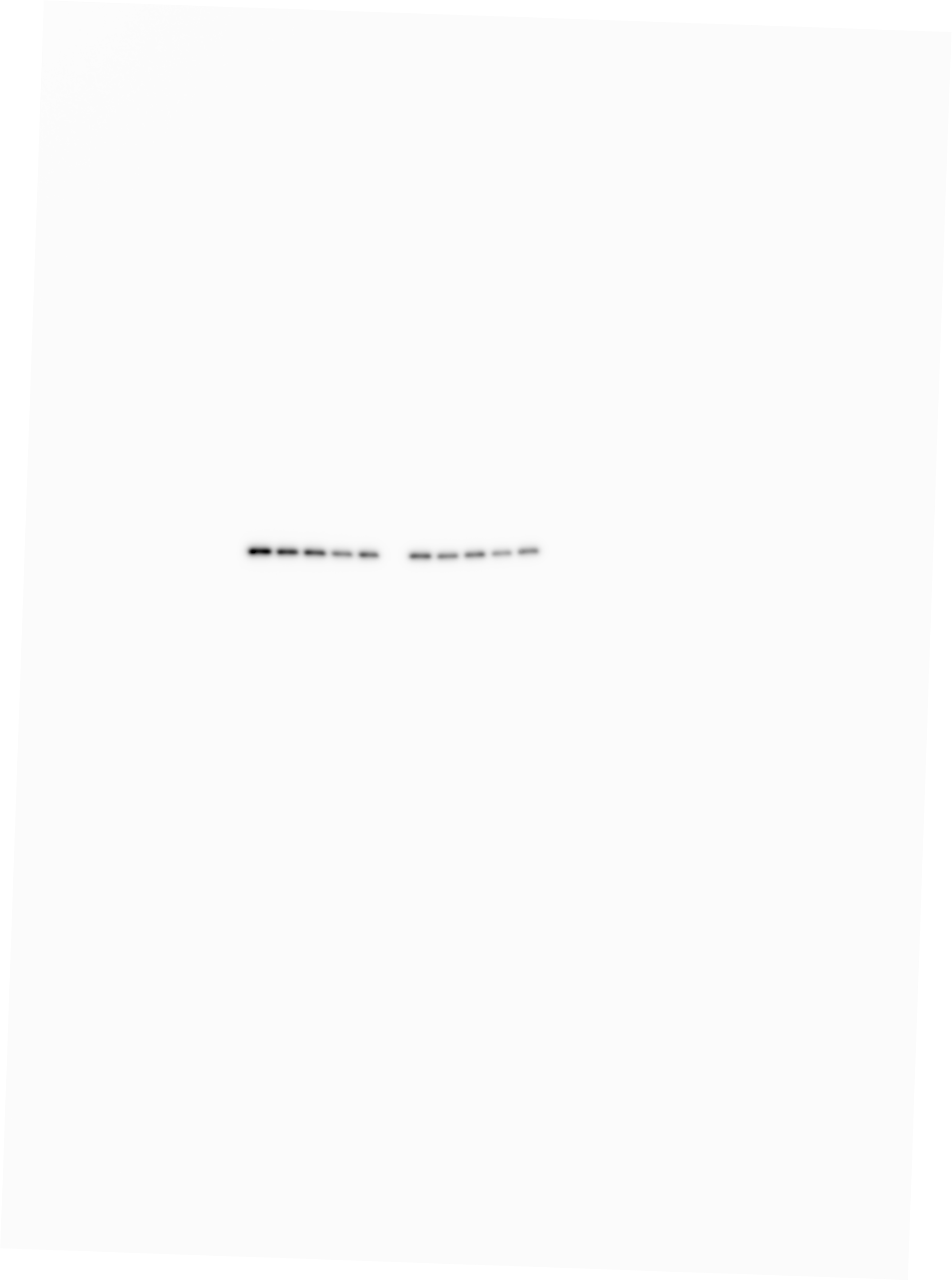

Supplement: Figure 5—source data 4. [file elife-81573-fig5-data4.zip › Figure 5-source data 4/Figure 5-source data 4_raw files/LK220707 Fig5G Gpdh 2022.07.07_22.27.16-03_Ch/LK220707 Fig5G Gpdh 2022.07.07_22.27.16-03_Ch_rotated.tif]

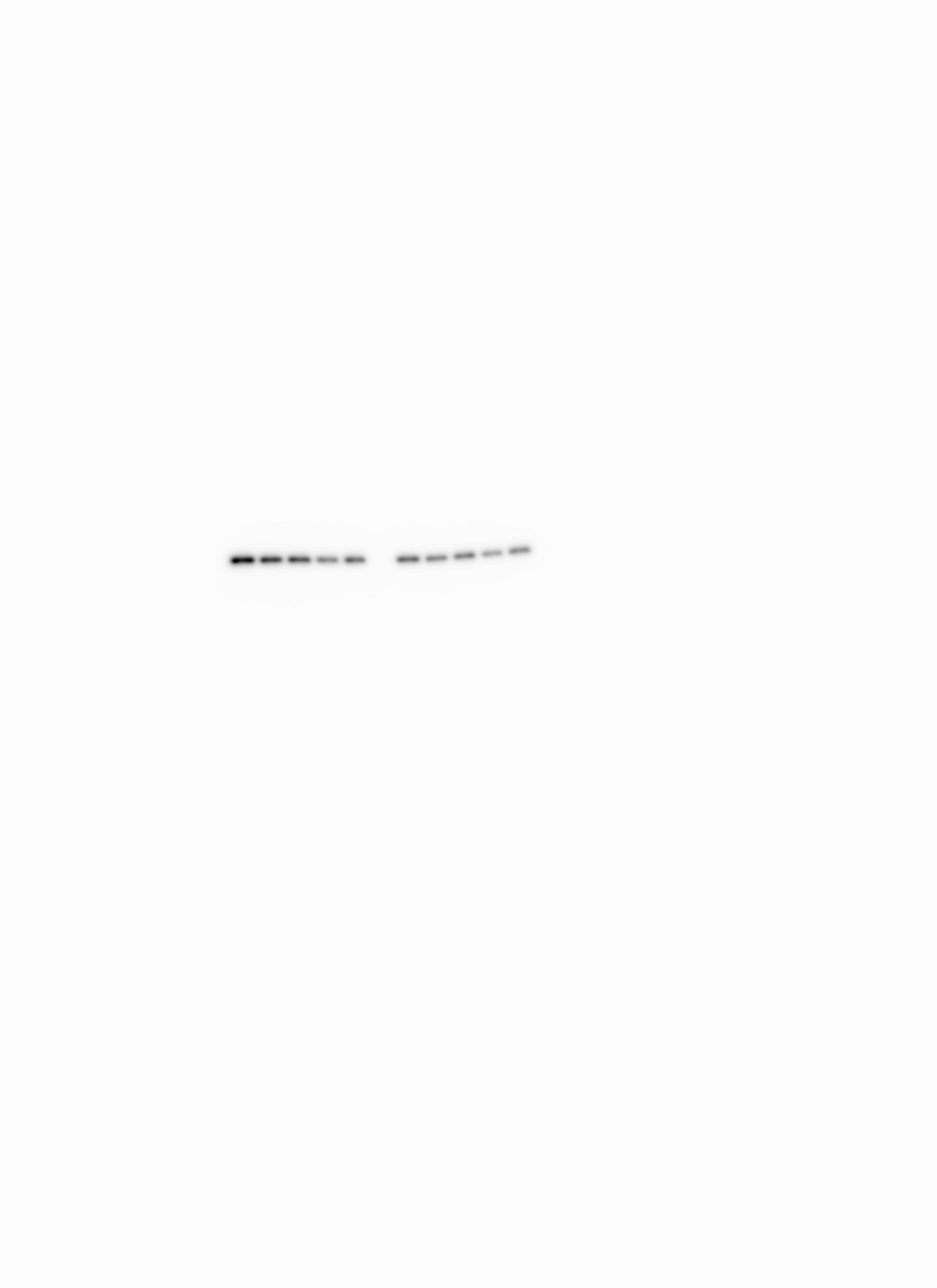

Supplement: Figure 5—source data 4. [file elife-81573-fig5-data4.zip › Figure 5-source data 4/Figure 5-source data 4_raw files/LK220707 Fig5G Gpdh 2022.07.07_22.27.16-03_Ch/LK220707 Fig5G Gpdh 2022.07.07_22.27.16-03_Ch.jpg]

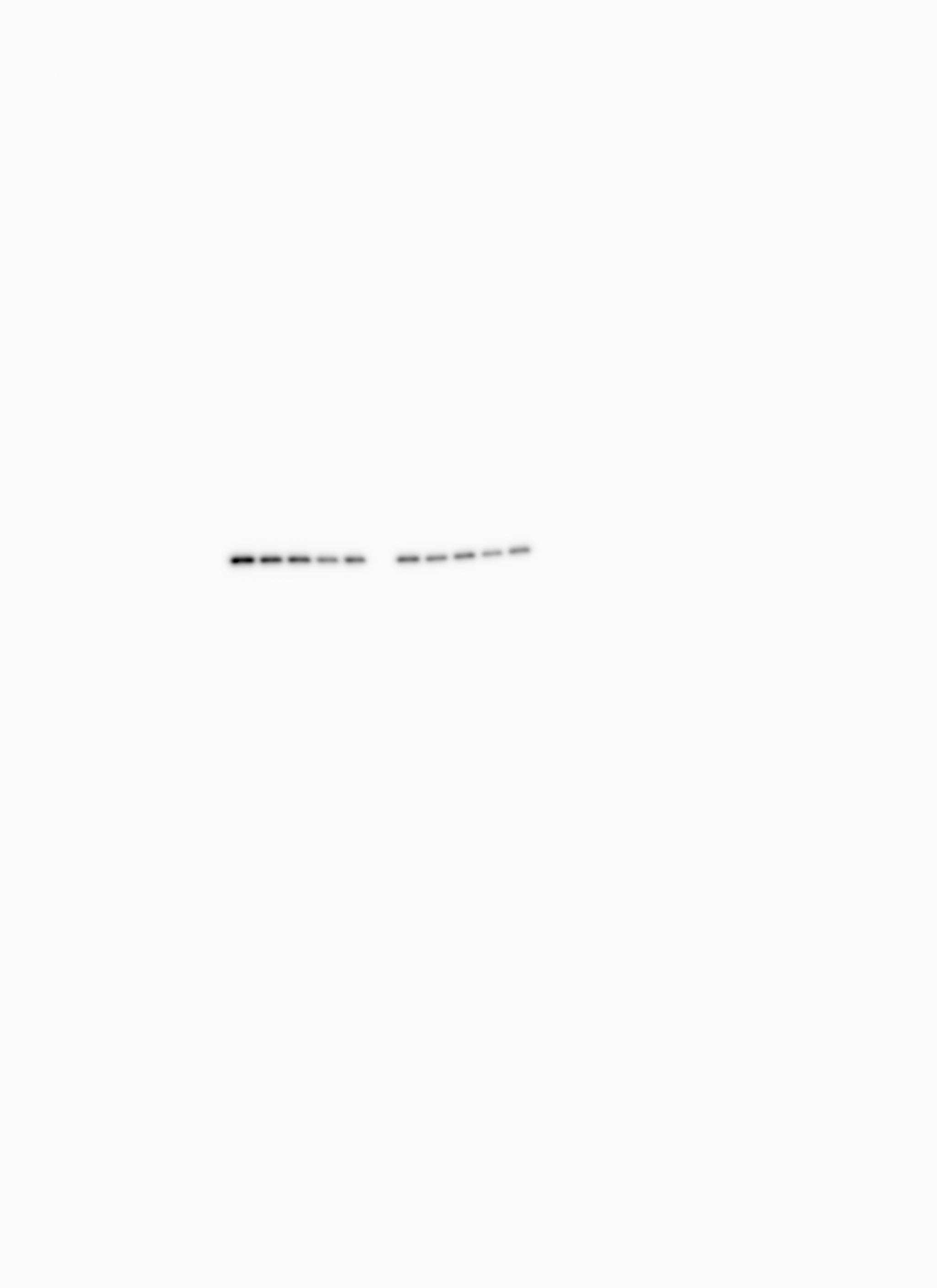

Supplement: Figure 5—source data 4. [file elife-81573-fig5-data4.zip › Figure 5-source data 4/Figure 5-source data 4_raw files/LK220707 Fig5G Gpdh 2022.07.07_22.27.16-03_Ch/LK220707 Fig5G Gpdh 2022.07.07_22.27.16-03_Ch.tif]

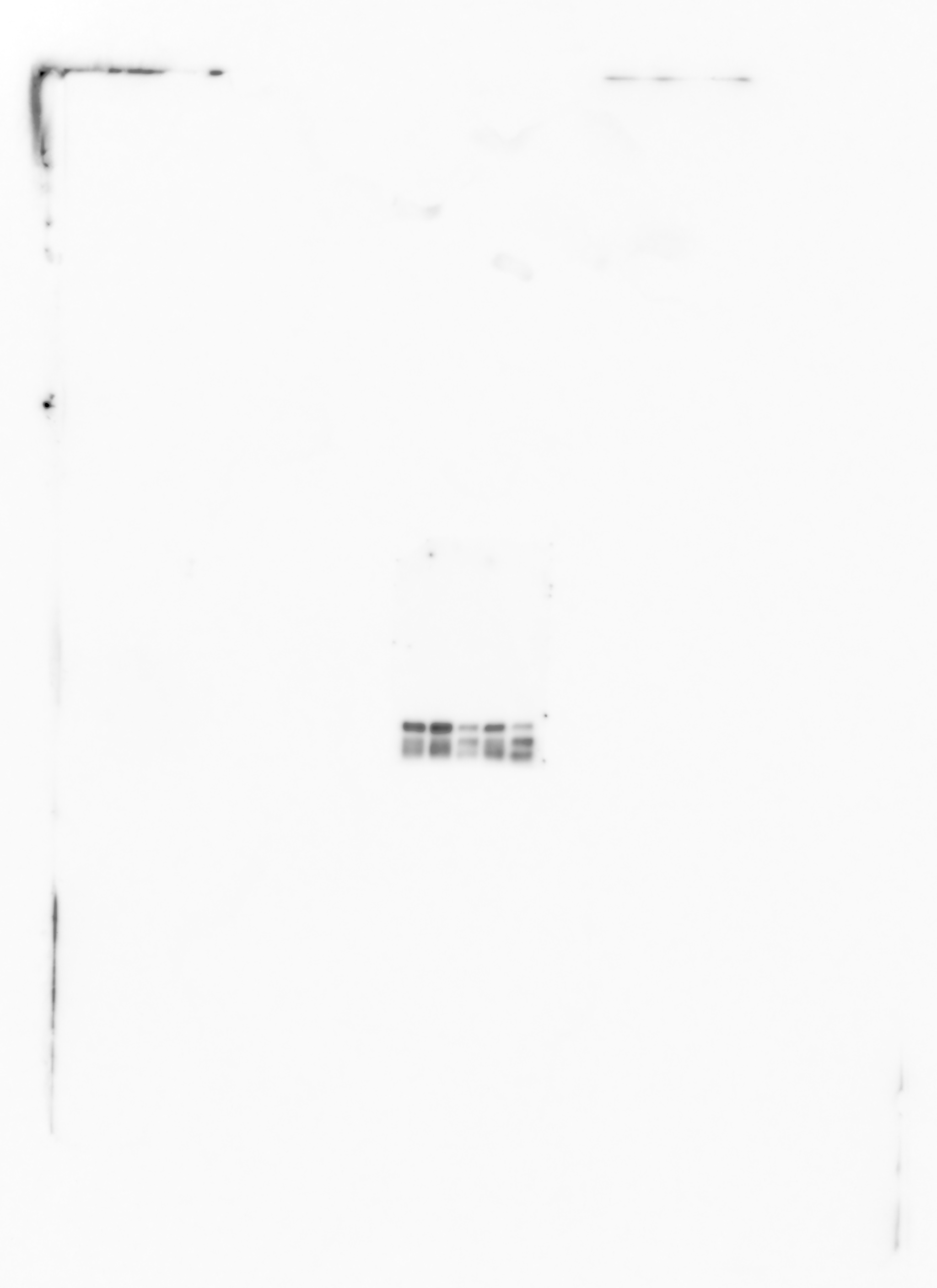

Supplement: Figure 5—source data 4. [file elife-81573-fig5-data4.zip › Figure 5-source data 4/Figure 5-source data 4_raw files/LK220707 Fig5G-2 Sun 2022.07.07_23.57.24-12_Ch/LK220707 Fig5G-2 Sun 2022.07.07_23.57.24-12_Ch.tif]

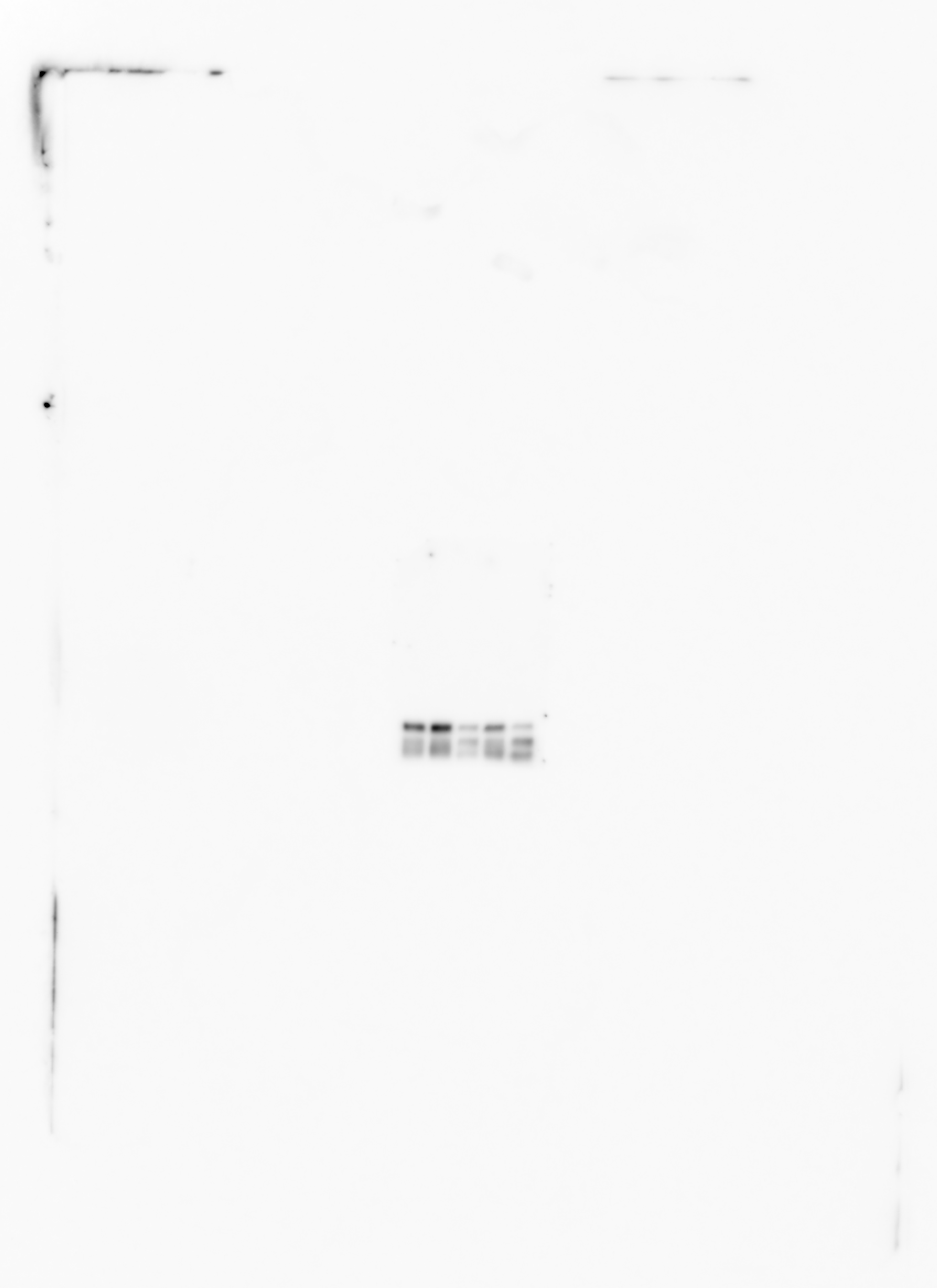

Supplement: Figure 5—source data 4. [file elife-81573-fig5-data4.zip › Figure 5-source data 4/Figure 5-source data 4_raw files/LK220707 Fig5G-2 Sun 2022.07.07_23.57.24-12_Ch/LK220707 Fig5G-2 Sun 2022.07.07_23.57.24-12_Ch_intensified.tif]

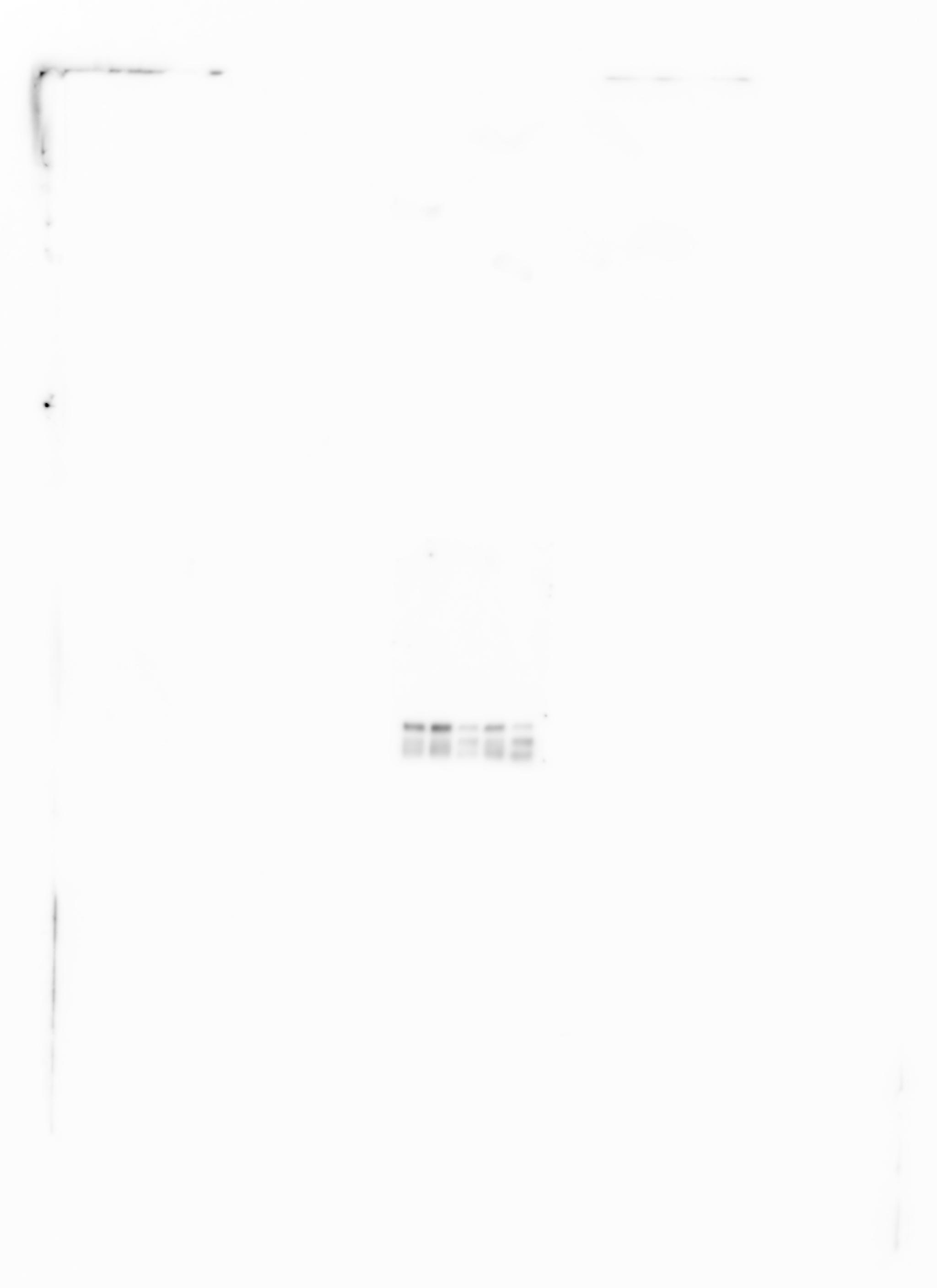

Supplement: Figure 5—source data 4. [file elife-81573-fig5-data4.zip › Figure 5-source data 4/Figure 5-source data 4_raw files/LK220707 Fig5G-2 Sun 2022.07.07_23.57.24-12_Ch/LK220707 Fig5G-2 Sun 2022.07.07_23.57.24-12_Ch.jpg]

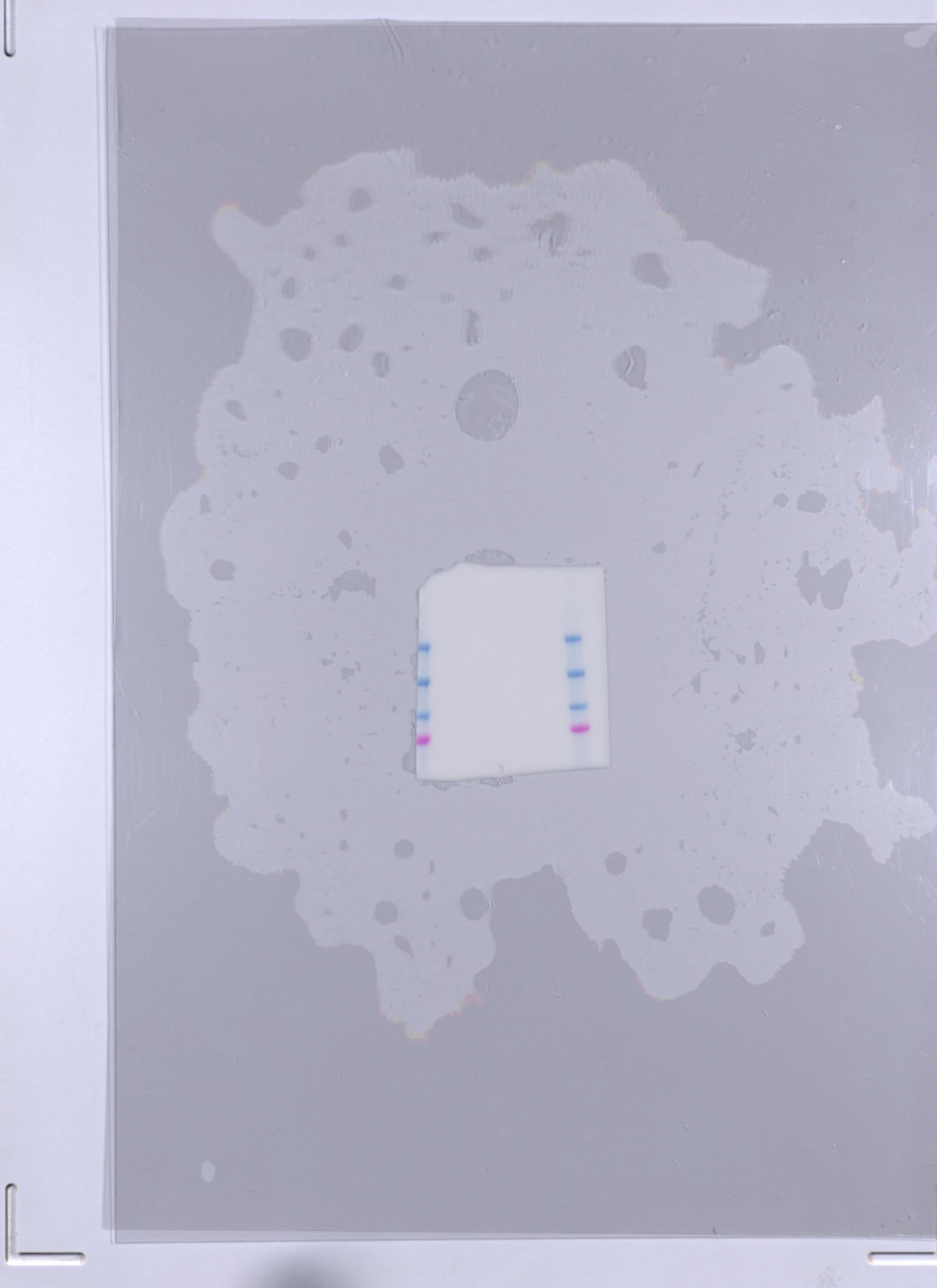

Supplement: Figure 5—source data 4. [file elife-81573-fig5-data4.zip › Figure 5-source data 4/Figure 5-source data 4_raw files/LK220707 Fig5G HA 2022.07.07_22.39.36_Ch/LK220707 Fig5G HA 2022.07.07_22.39.36_Ch-Marker.jpg]

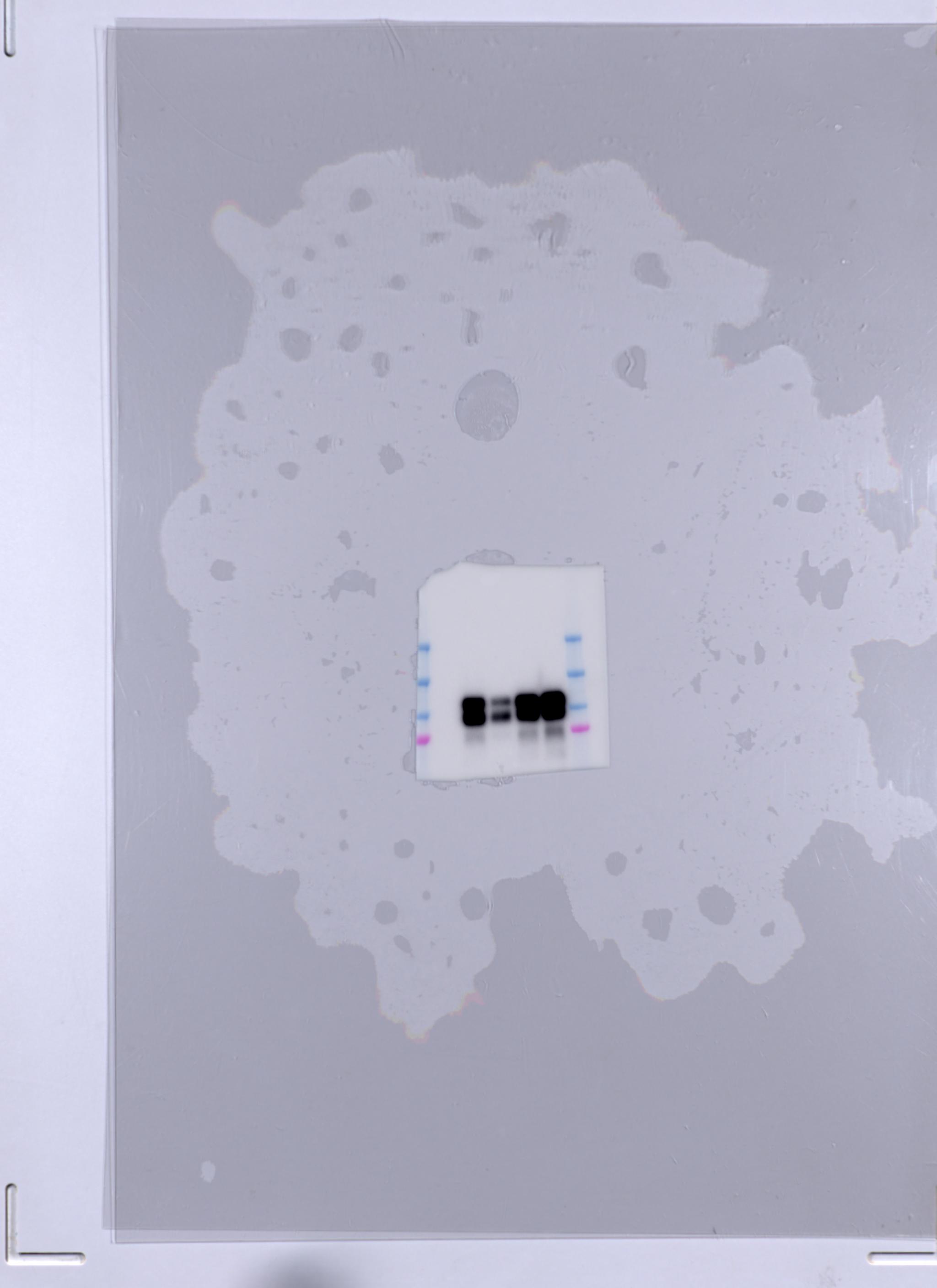

Supplement: Figure 5—source data 4. [file elife-81573-fig5-data4.zip › Figure 5-source data 4/Figure 5-source data 4_raw files/LK220707 Fig5G HA 2022.07.07_22.39.36_Ch/LK220707 Fig5G HA 2022.07.07_22.39.36_Ch+Marker.jpg]

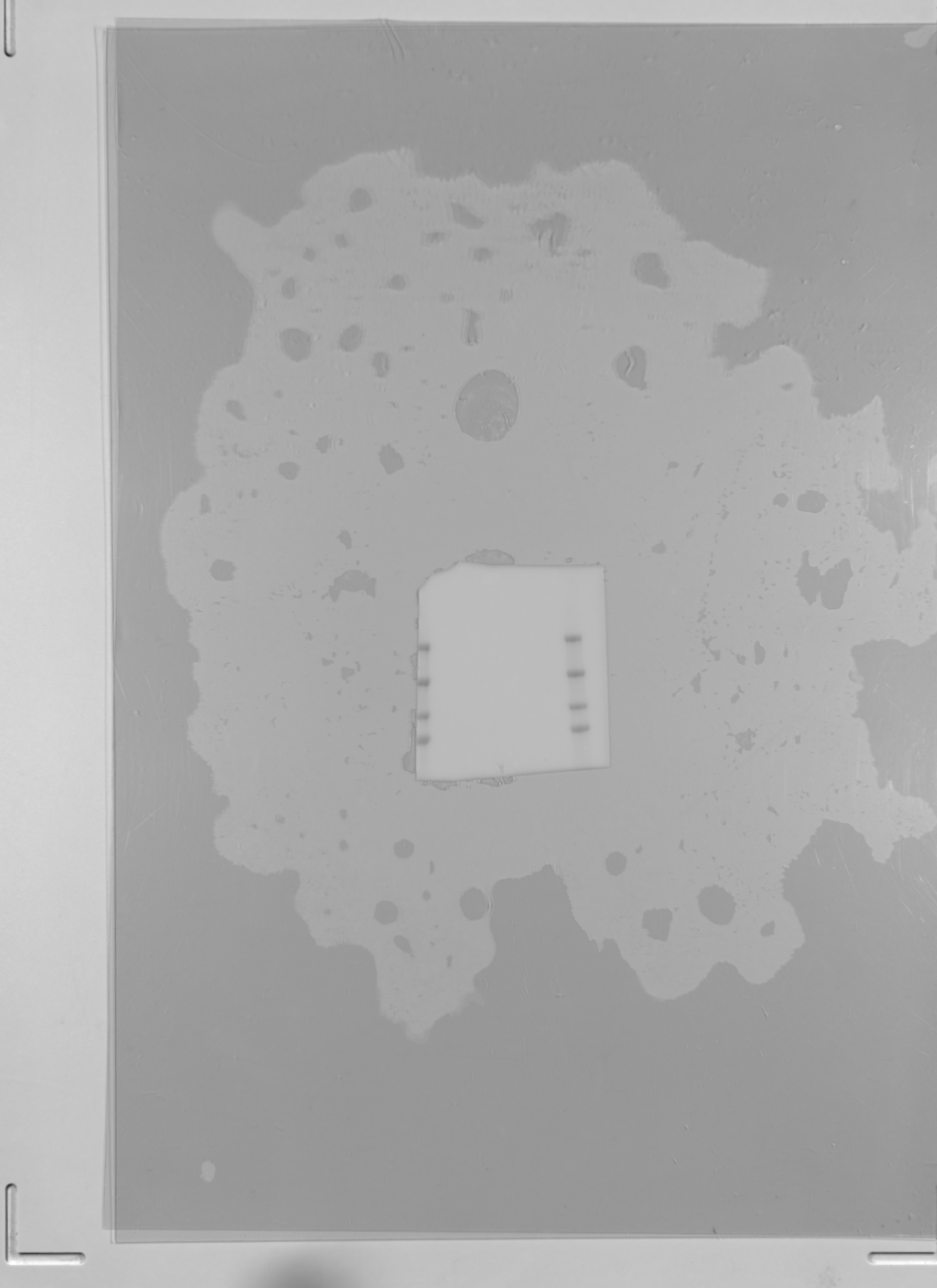

Supplement: Figure 5—source data 4. [file elife-81573-fig5-data4.zip › Figure 5-source data 4/Figure 5-source data 4_raw files/LK220707 Fig5G HA 2022.07.07_22.39.36_Ch/LK220707 Fig5G HA 2022.07.07_22.39.36_Ch-Marker.tif]

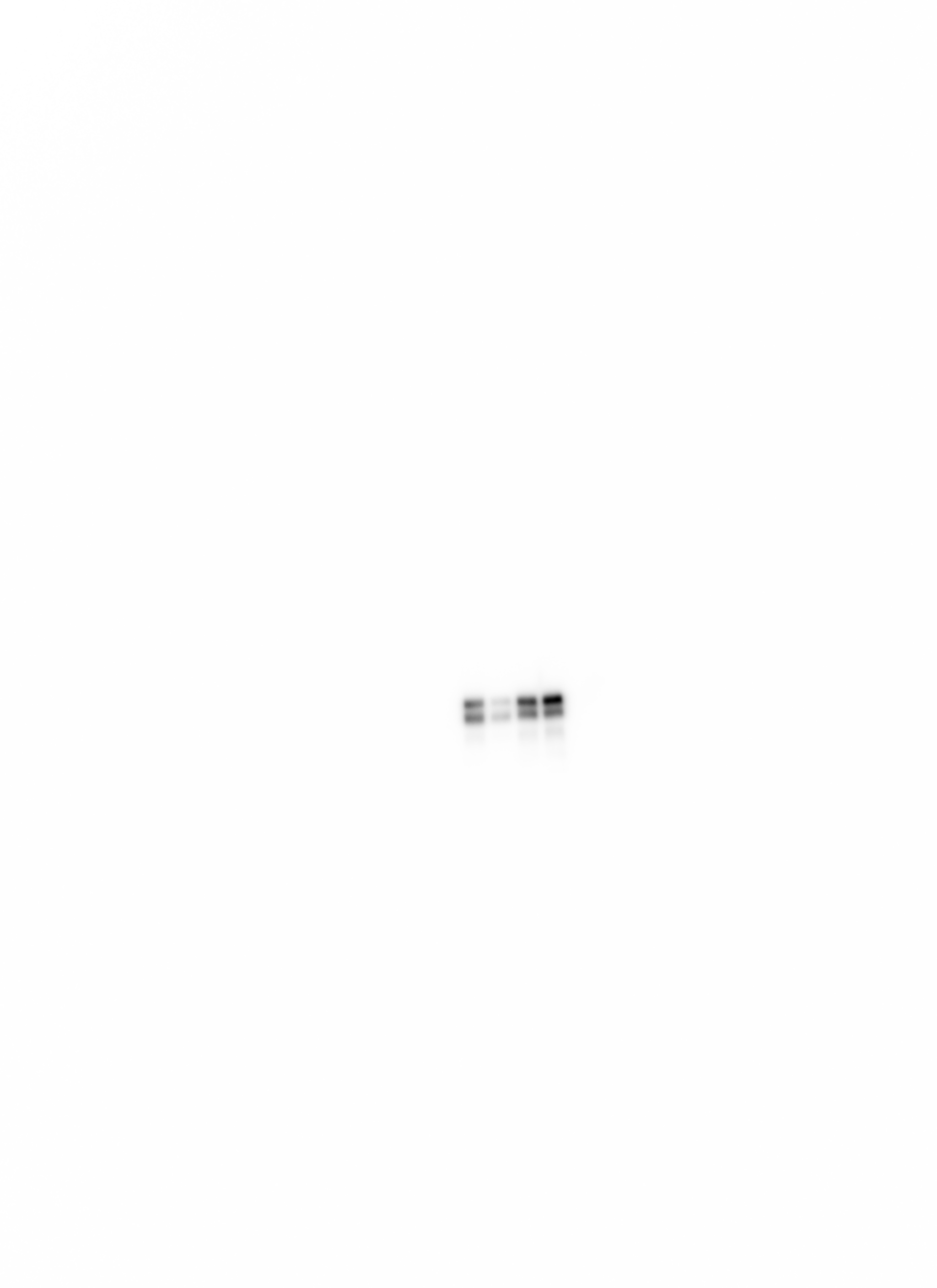

Supplement: Figure 5—source data 4. [file elife-81573-fig5-data4.zip › Figure 5-source data 4/Figure 5-source data 4_raw files/LK220707 Fig5G HA 2022.07.07_22.39.36_Ch/LK220707 Fig5G HA 2022.07.07_22.39.36_Ch.tif]

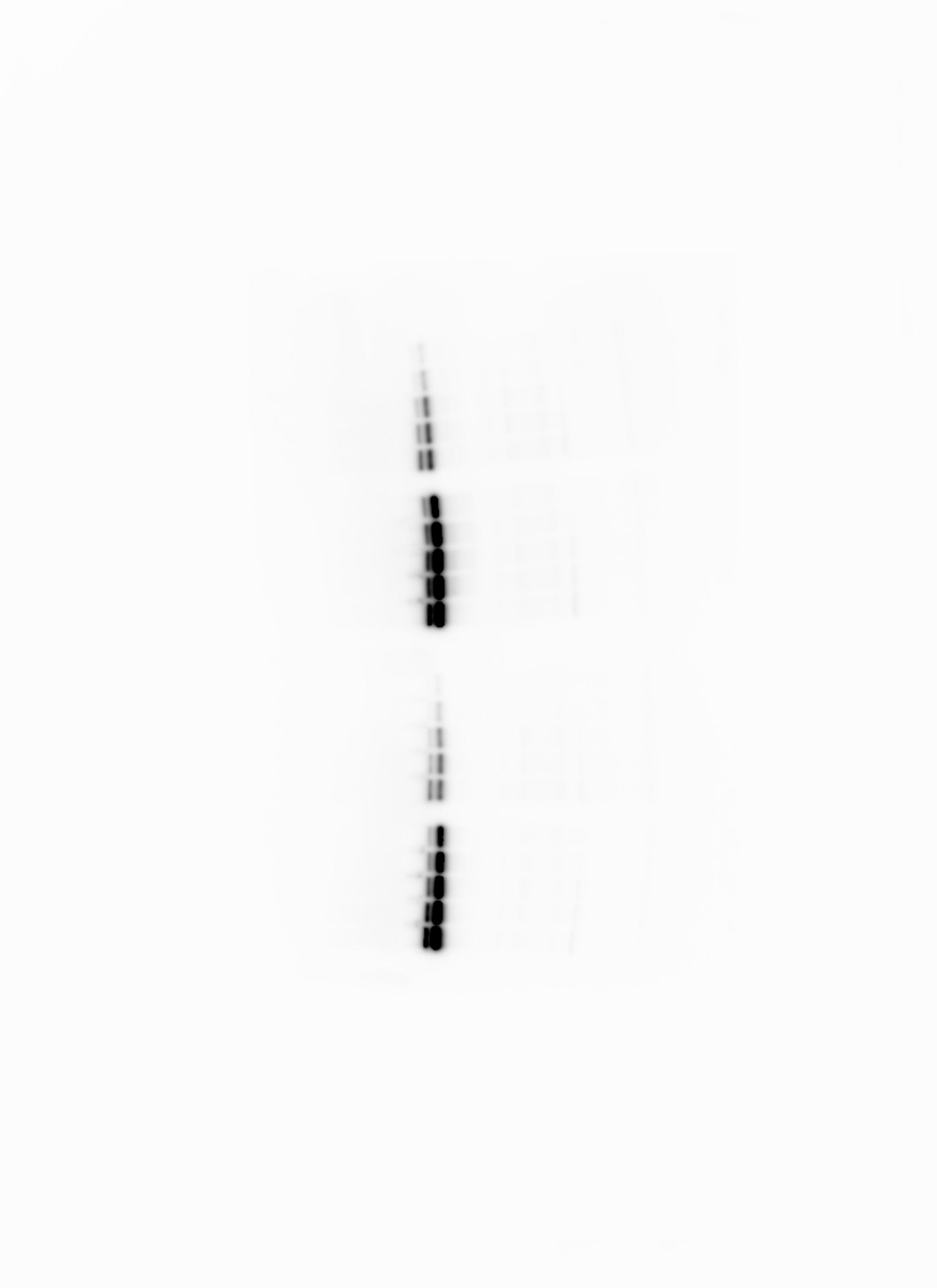

Supplement: Figure 5—figure supplement 1—source data 1. [file elife-81573-fig5-figsupp1-data1.zip › Figure 5-supplement 1-source data 1/Figure 5-supplement 1-source data 1_raw files/SUN2 CTKO CHX HA 2022.03.09_18.48.34-07_Ch.jpg]

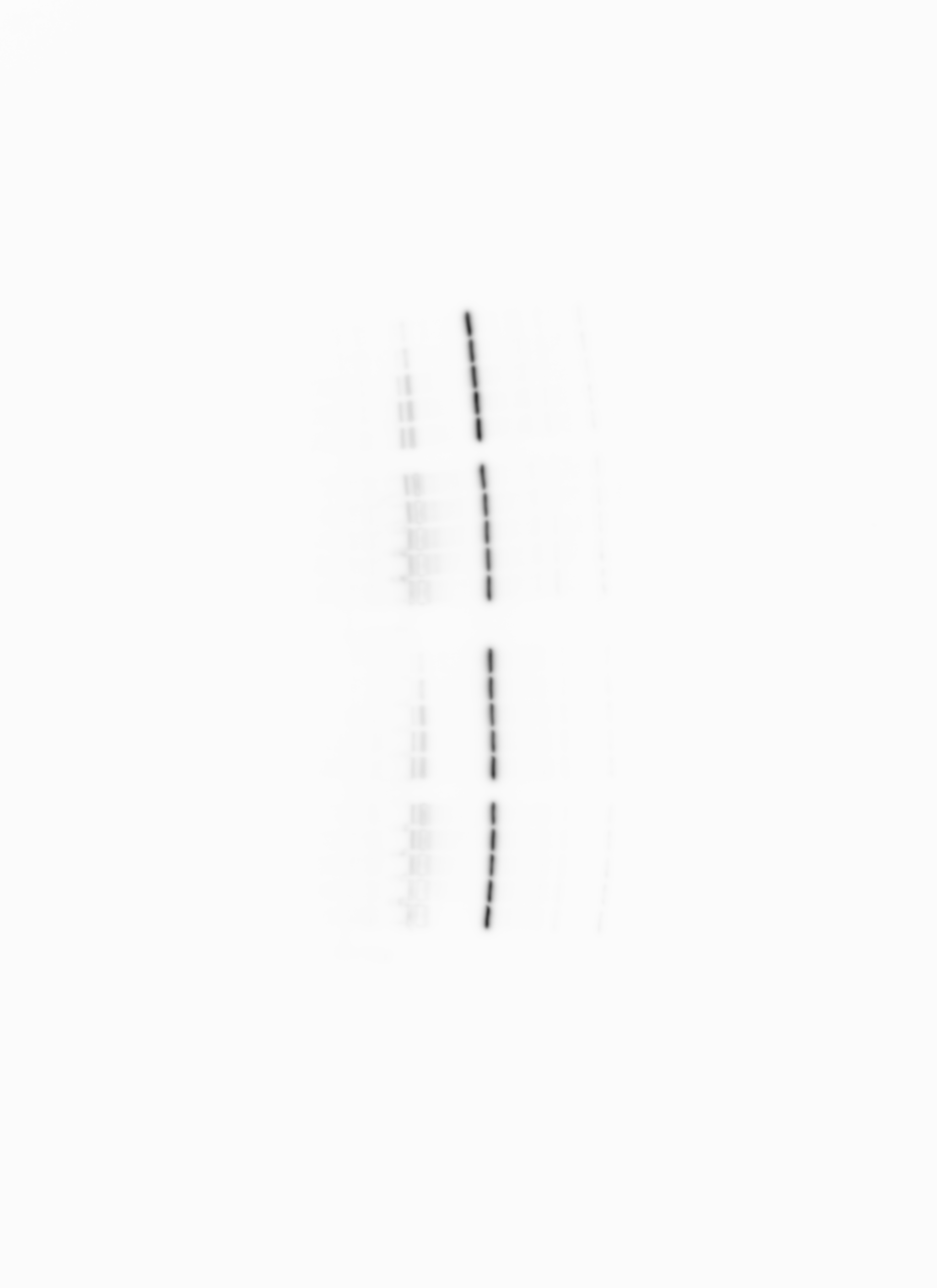

Supplement: Figure 5—figure supplement 1—source data 1. [file elife-81573-fig5-figsupp1-data1.zip › Figure 5-supplement 1-source data 1/Figure 5-supplement 1-source data 1_raw files/SUN2 CTKO CHX Tub 2022.03.10_17.42.17-02_Ch.tif]

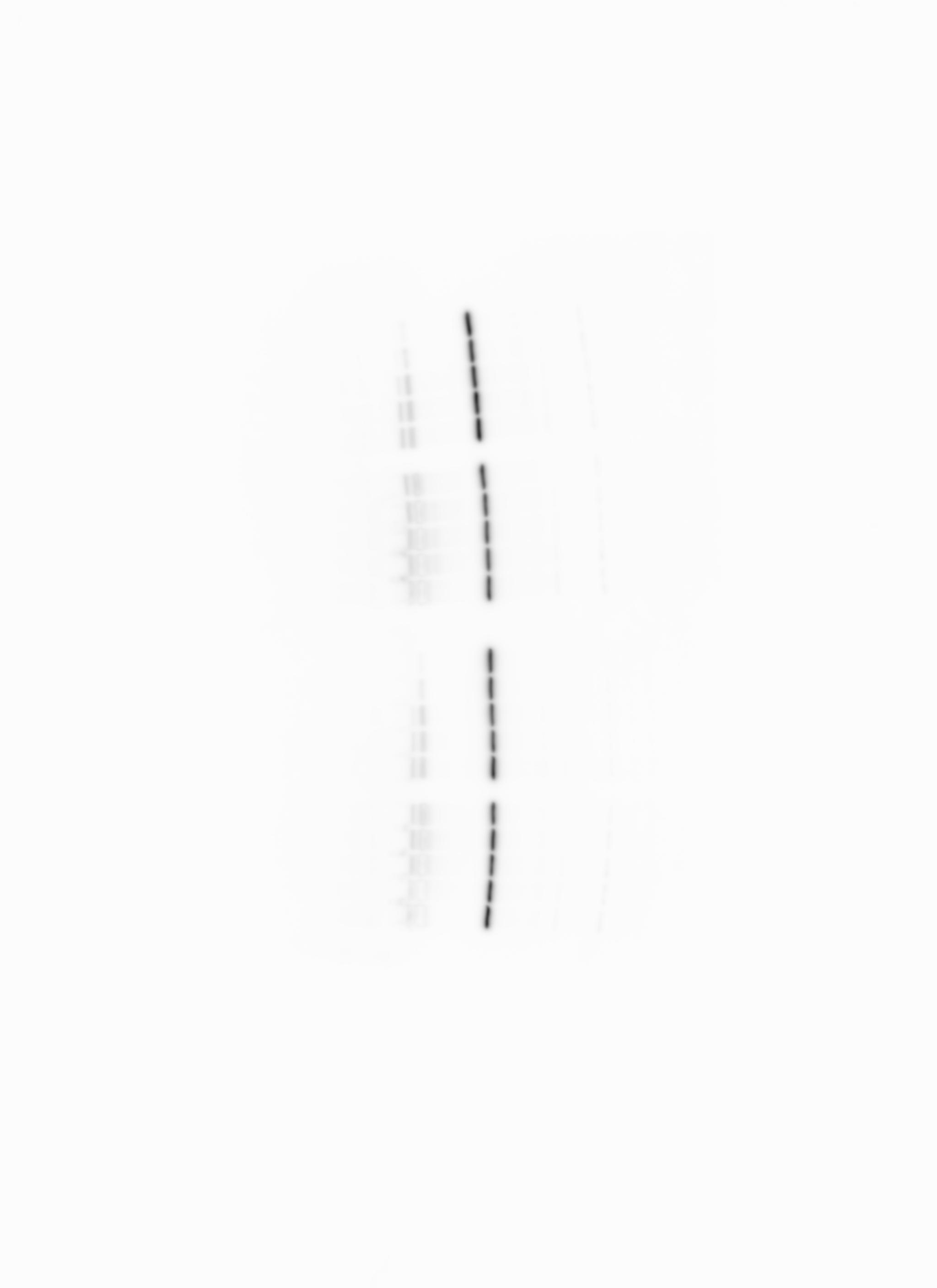

Supplement: Figure 5—figure supplement 1—source data 1. [file elife-81573-fig5-figsupp1-data1.zip › Figure 5-supplement 1-source data 1/Figure 5-supplement 1-source data 1_raw files/SUN2 CTKO CHX Tub 2022.03.10_17.42.17-02_Ch.jpg]

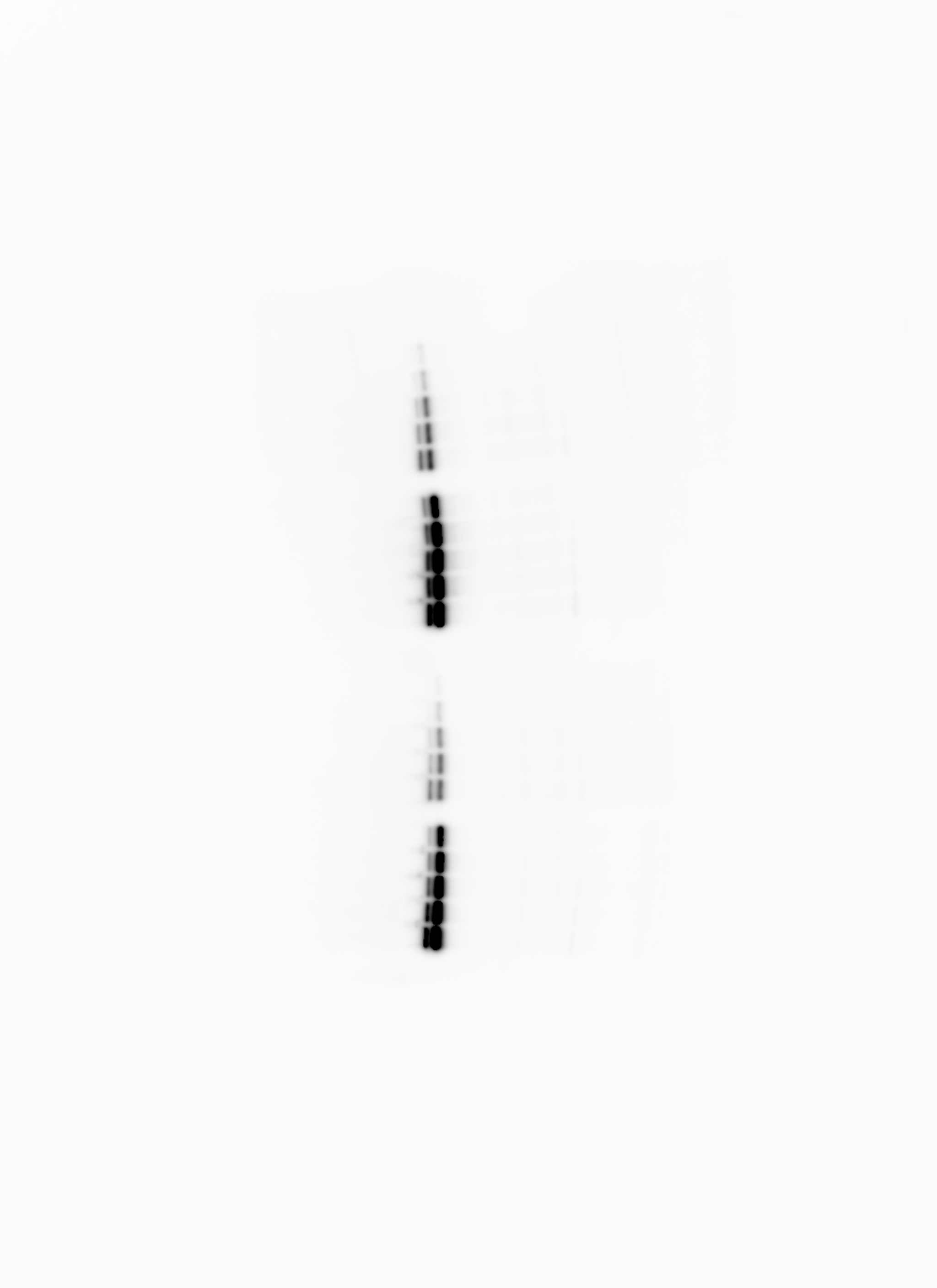

Supplement: Figure 5—figure supplement 1—source data 1. [file elife-81573-fig5-figsupp1-data1.zip › Figure 5-supplement 1-source data 1/Figure 5-supplement 1-source data 1_raw files/SUN2 CTKO CHX HA 2022.03.09_18.48.34-07_Ch.tif]

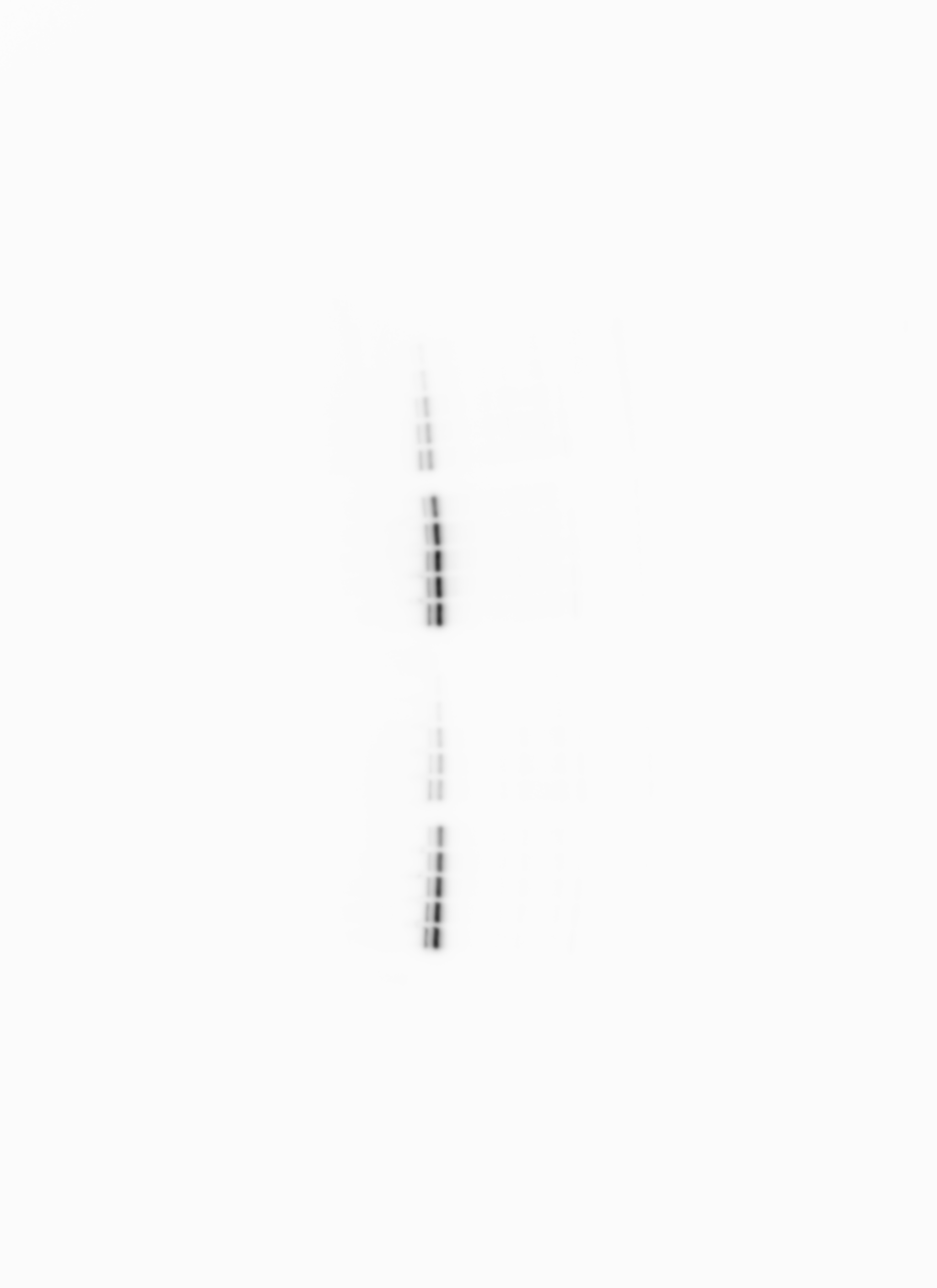

Supplement: Figure 5—figure supplement 1—source data 1. [file elife-81573-fig5-figsupp1-data1.zip › Figure 5-supplement 1-source data 1/Figure 5-supplement 1-source data 1_raw files/SUN2 CTKO CHX HA 2022.03.09_18.48.34-02_Ch.tif]

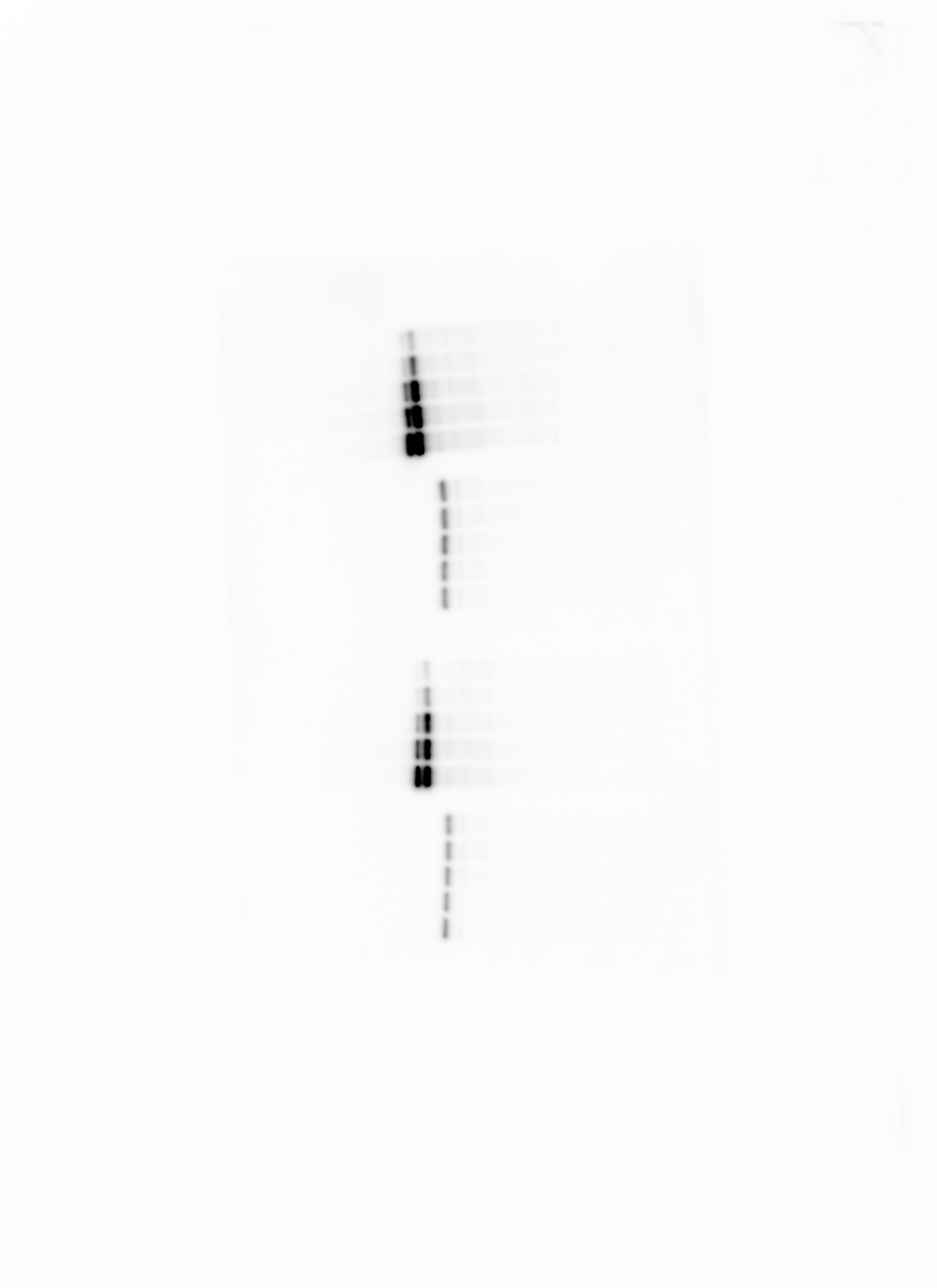

Supplement: Figure 5—figure supplement 1—source data 1. [file elife-81573-fig5-figsupp1-data1.zip › Figure 5-supplement 1-source data 1/Figure 5-supplement 1-source data 1_raw files/SUN2 CTKO CHX SUN2 2022.03.09_19.06.03-12_Ch.jpg]

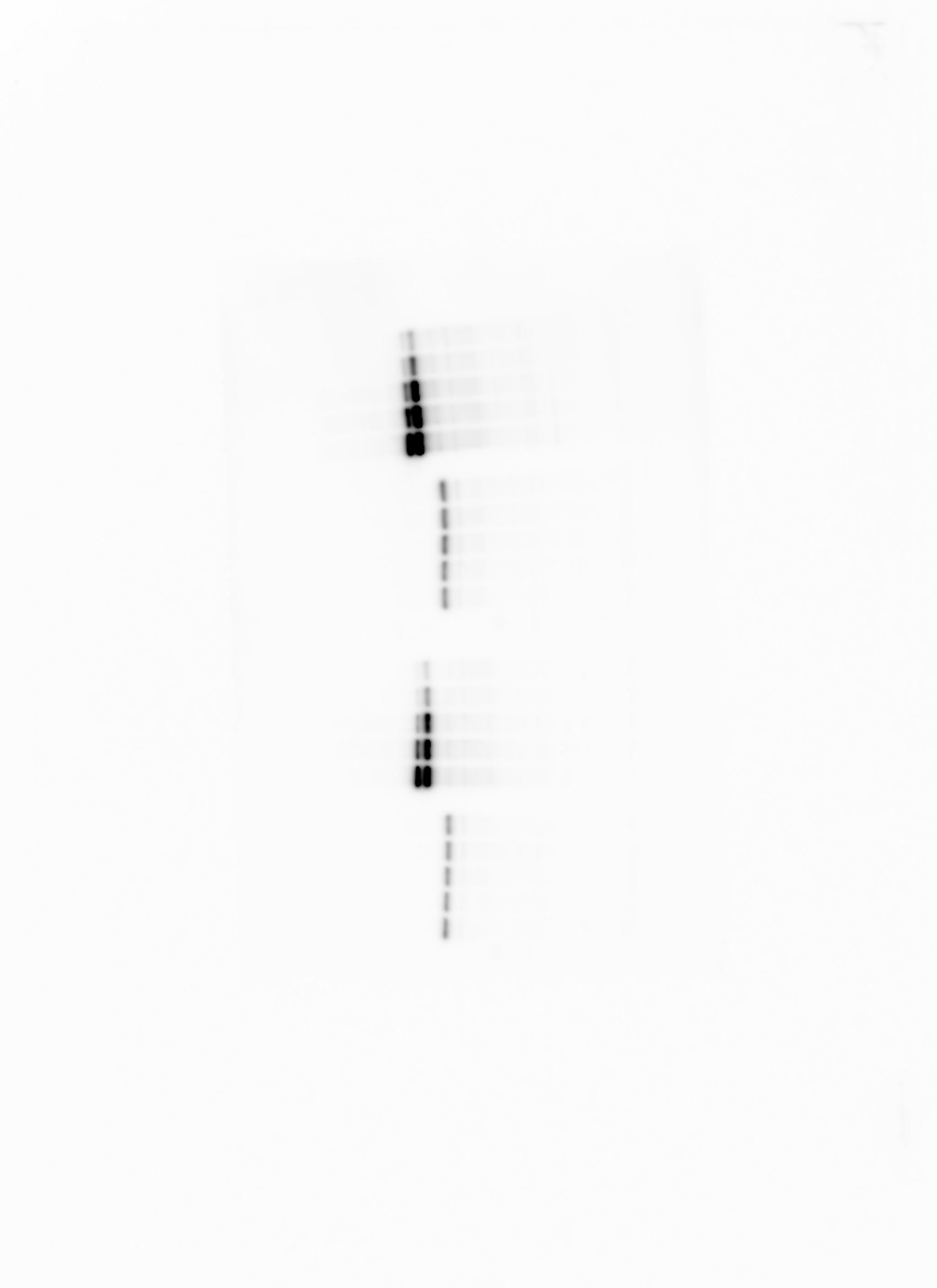

Supplement: Figure 5—figure supplement 1—source data 1. [file elife-81573-fig5-figsupp1-data1.zip › Figure 5-supplement 1-source data 1/Figure 5-supplement 1-source data 1_raw files/SUN2 CTKO CHX SUN2 2022.03.09_19.06.03-12_Ch.tif]

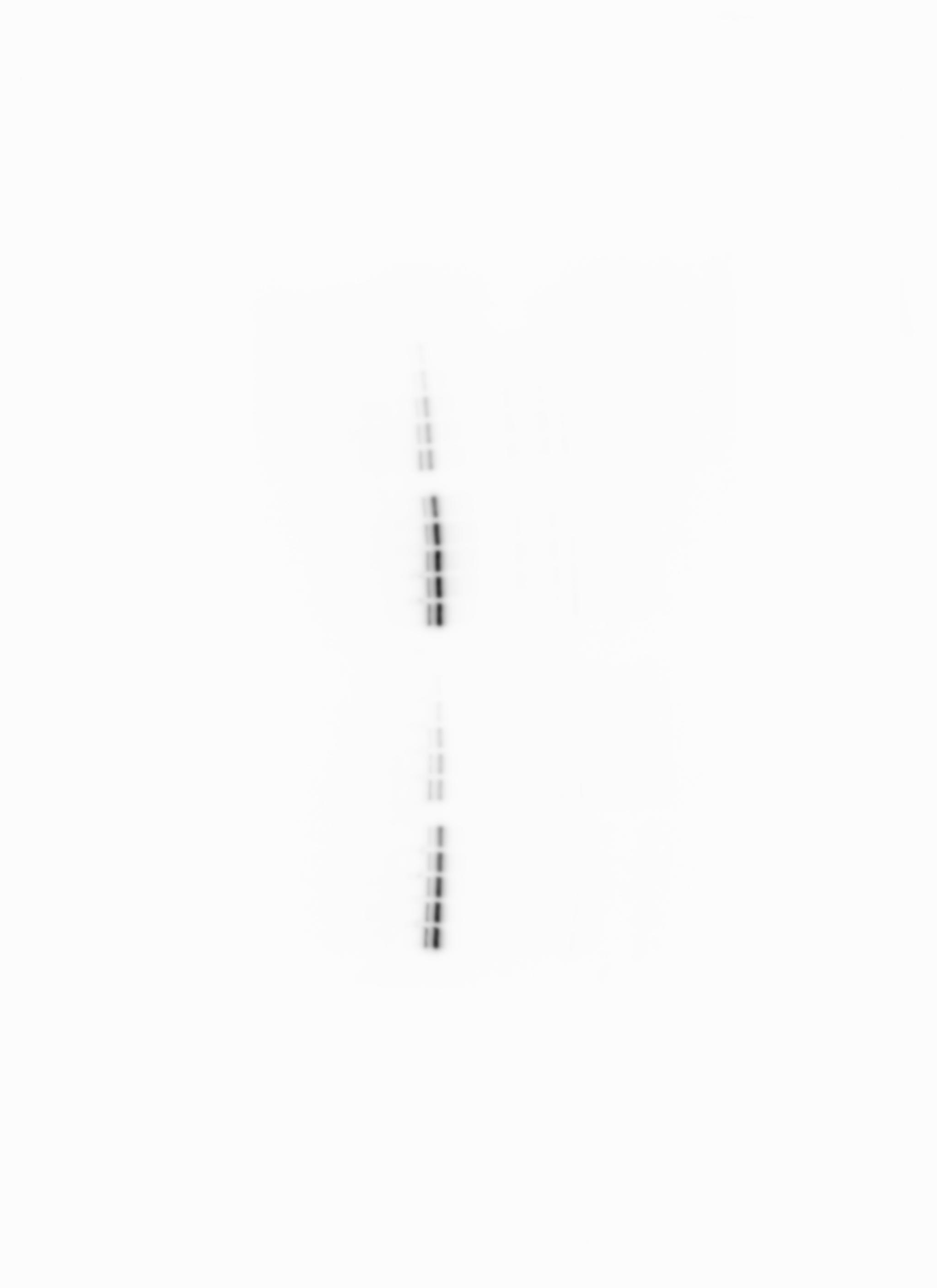

Supplement: Figure 5—figure supplement 1—source data 1. [file elife-81573-fig5-figsupp1-data1.zip › Figure 5-supplement 1-source data 1/Figure 5-supplement 1-source data 1_raw files/SUN2 CTKO CHX HA 2022.03.09_18.48.34-02_Ch.jpg]

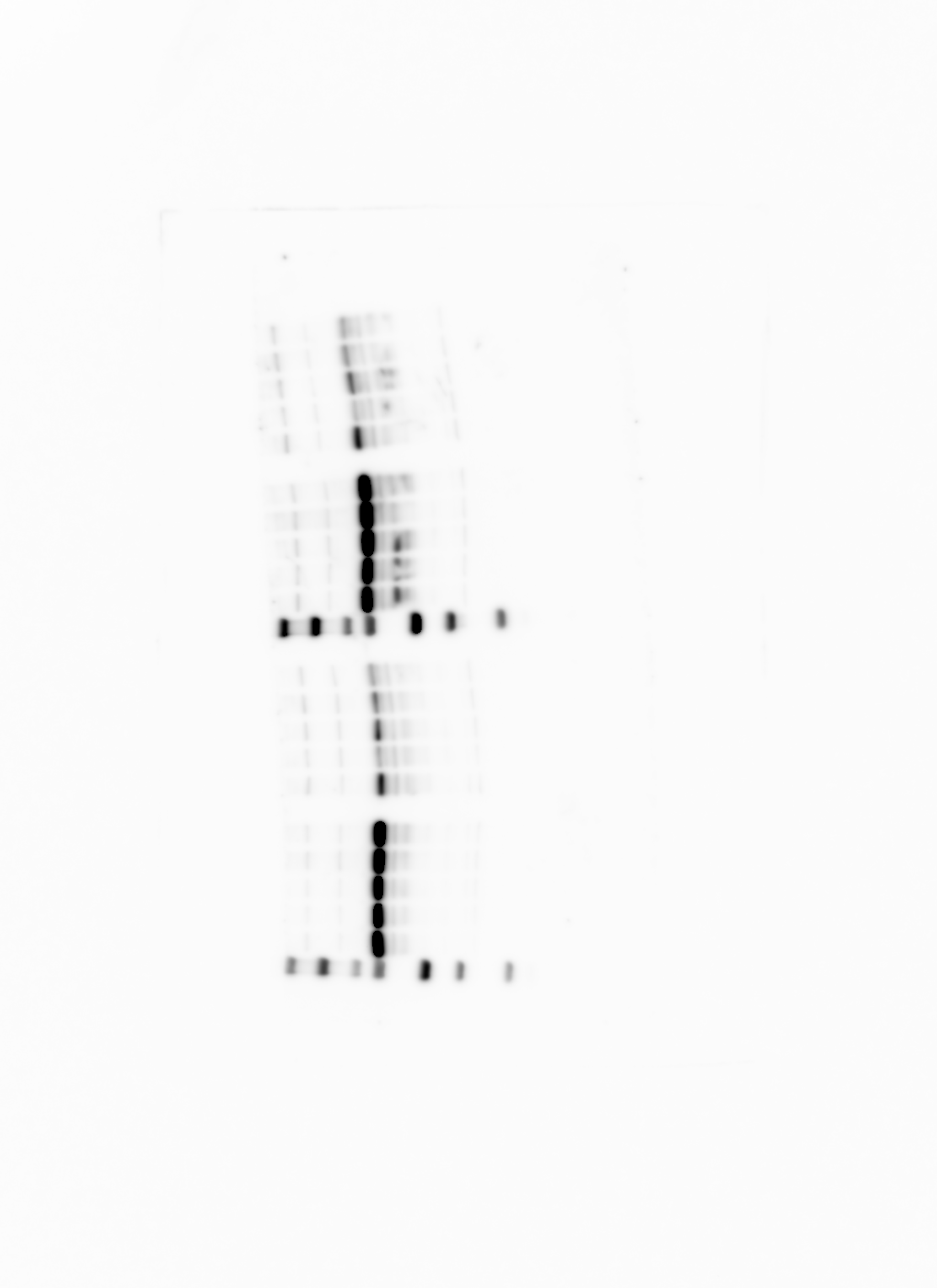

Supplement: Figure 5—figure supplement 1—source data 2. [file elife-81573-fig5-figsupp1-data2.zip › Figure 5-supplement 1-source data 2/Figure 5-supplement 1-source data 2_raw files/CTKO CHX240 inh 2022.08.24_17.48.45-09_Ch v SUN2/CTKO CHX240 inh 2022.08.24_17.48.45-09_Ch.tif]

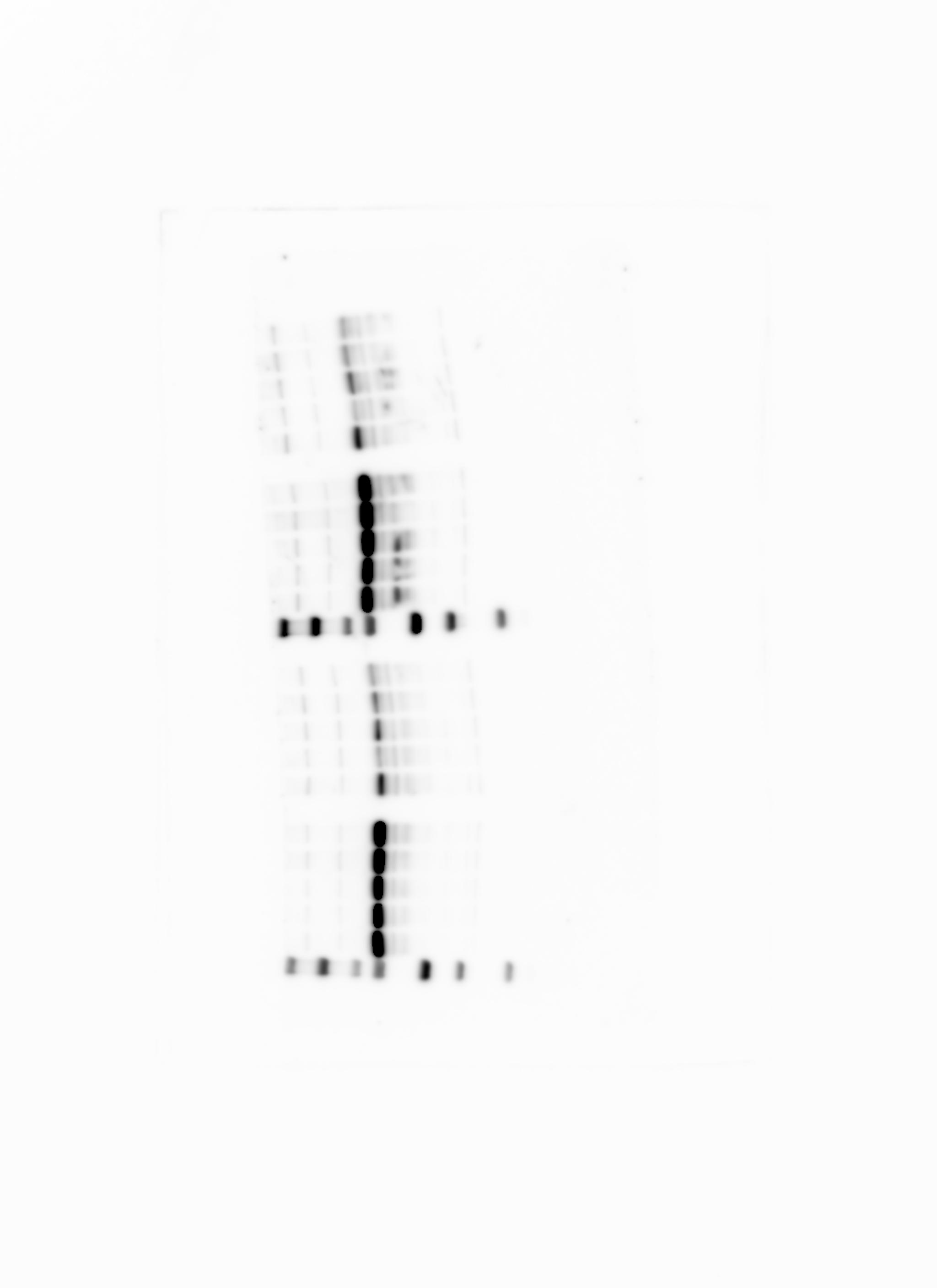

Supplement: Figure 5—figure supplement 1—source data 2. [file elife-81573-fig5-figsupp1-data2.zip › Figure 5-supplement 1-source data 2/Figure 5-supplement 1-source data 2_raw files/CTKO CHX240 inh 2022.08.24_17.48.45-09_Ch v SUN2/CTKO CHX240 inh 2022.08.24_17.48.45-09_Ch.jpg]

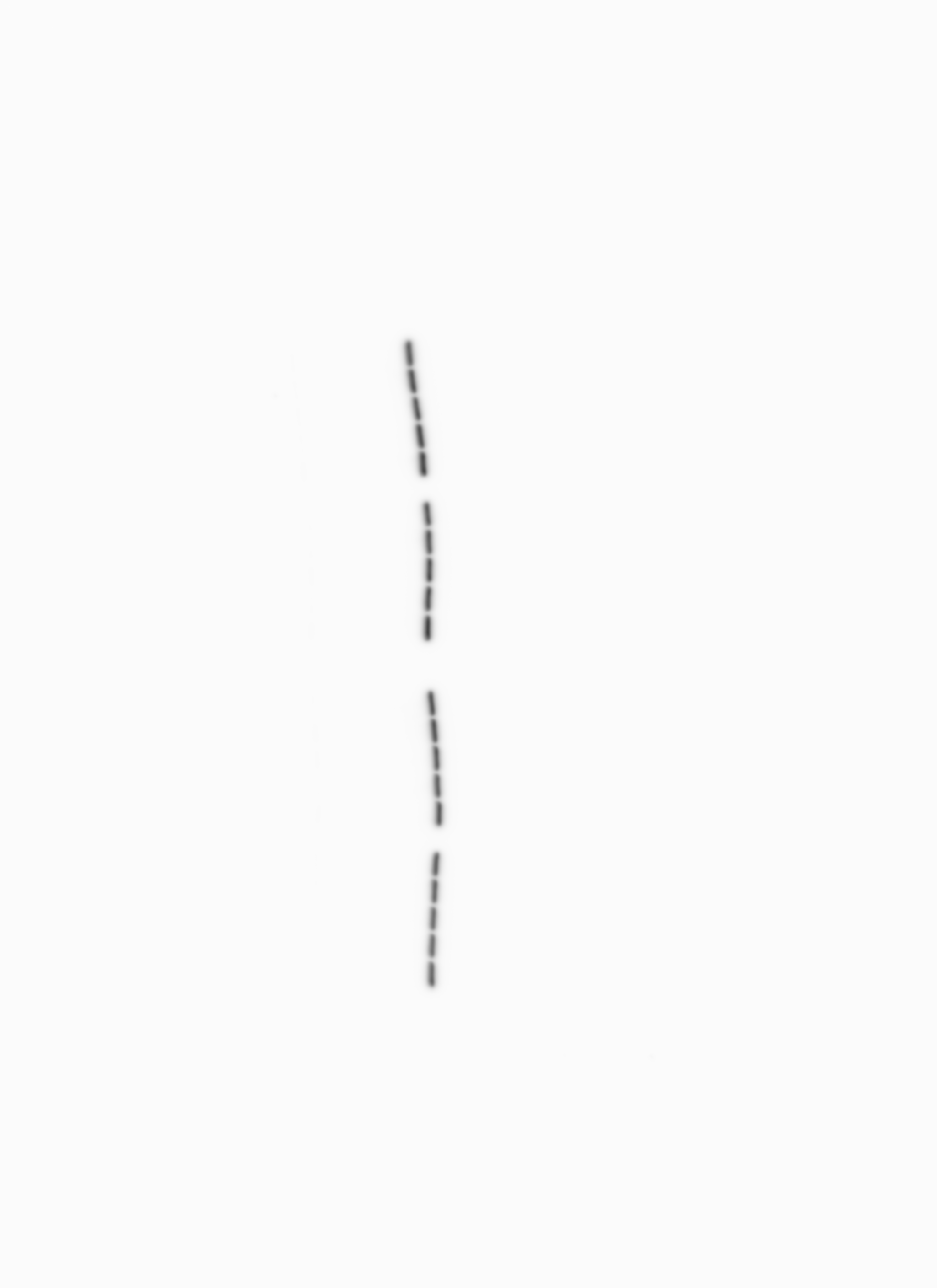

Supplement: Figure 5—figure supplement 1—source data 2. [file elife-81573-fig5-figsupp1-data2.zip › Figure 5-supplement 1-source data 2/Figure 5-supplement 1-source data 2_raw files/CTDNEP1KO CHX240 Tub 2022.08.25_17.18.37_Ch v Tub/CTDNEP1KO CHX240 Tub 2022.08.25_17.18.37_Ch.tif]

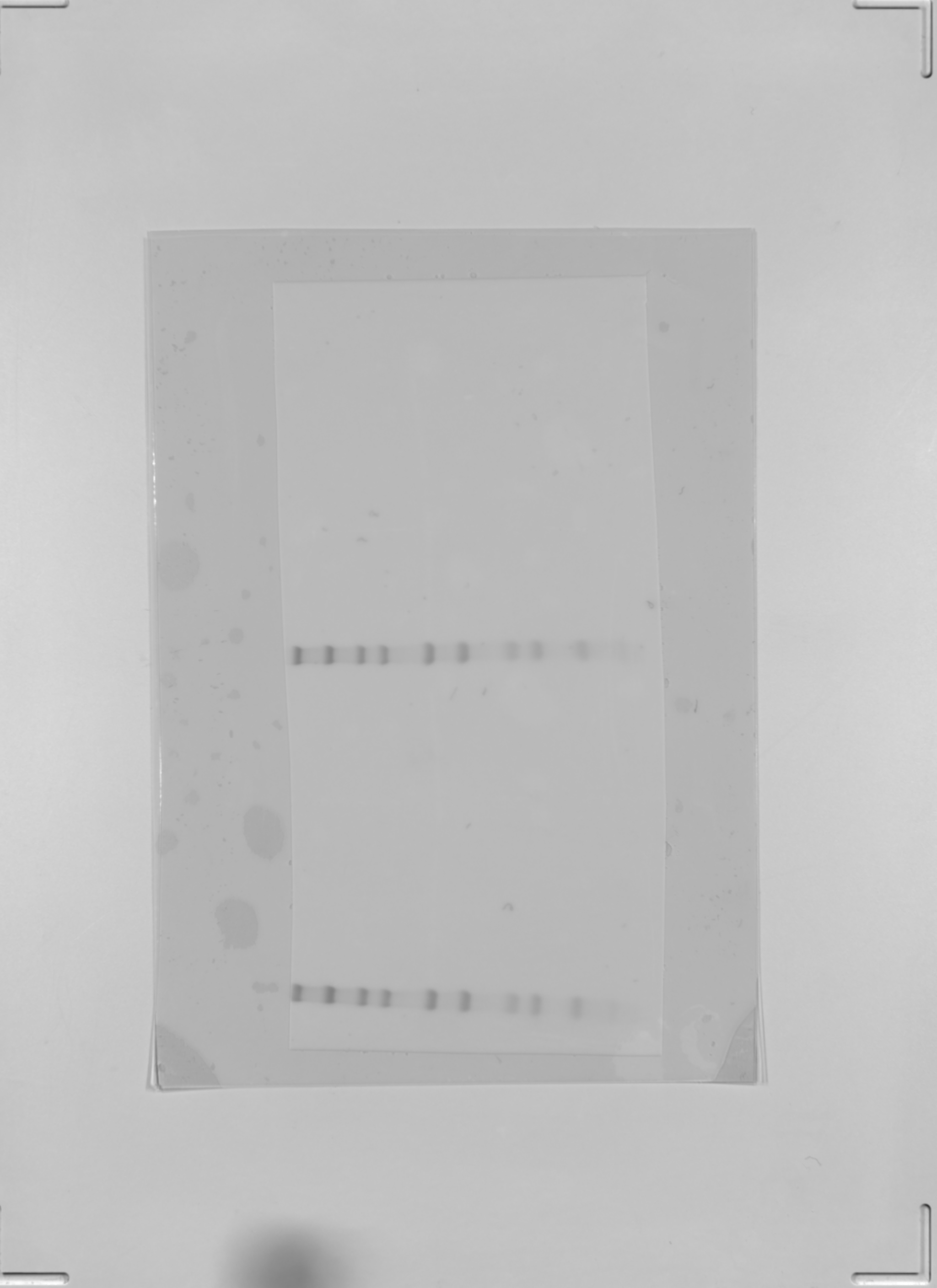

Supplement: Figure 5—figure supplement 1—source data 2. [file elife-81573-fig5-figsupp1-data2.zip › Figure 5-supplement 1-source data 2/Figure 5-supplement 1-source data 2_raw files/CTDNEP1KO CHX240 Tub 2022.08.25_17.18.37_Ch v Tub/CTDNEP1KO CHX240 Tub 2022.08.25_17.18.37_Ch-Marker.tif]

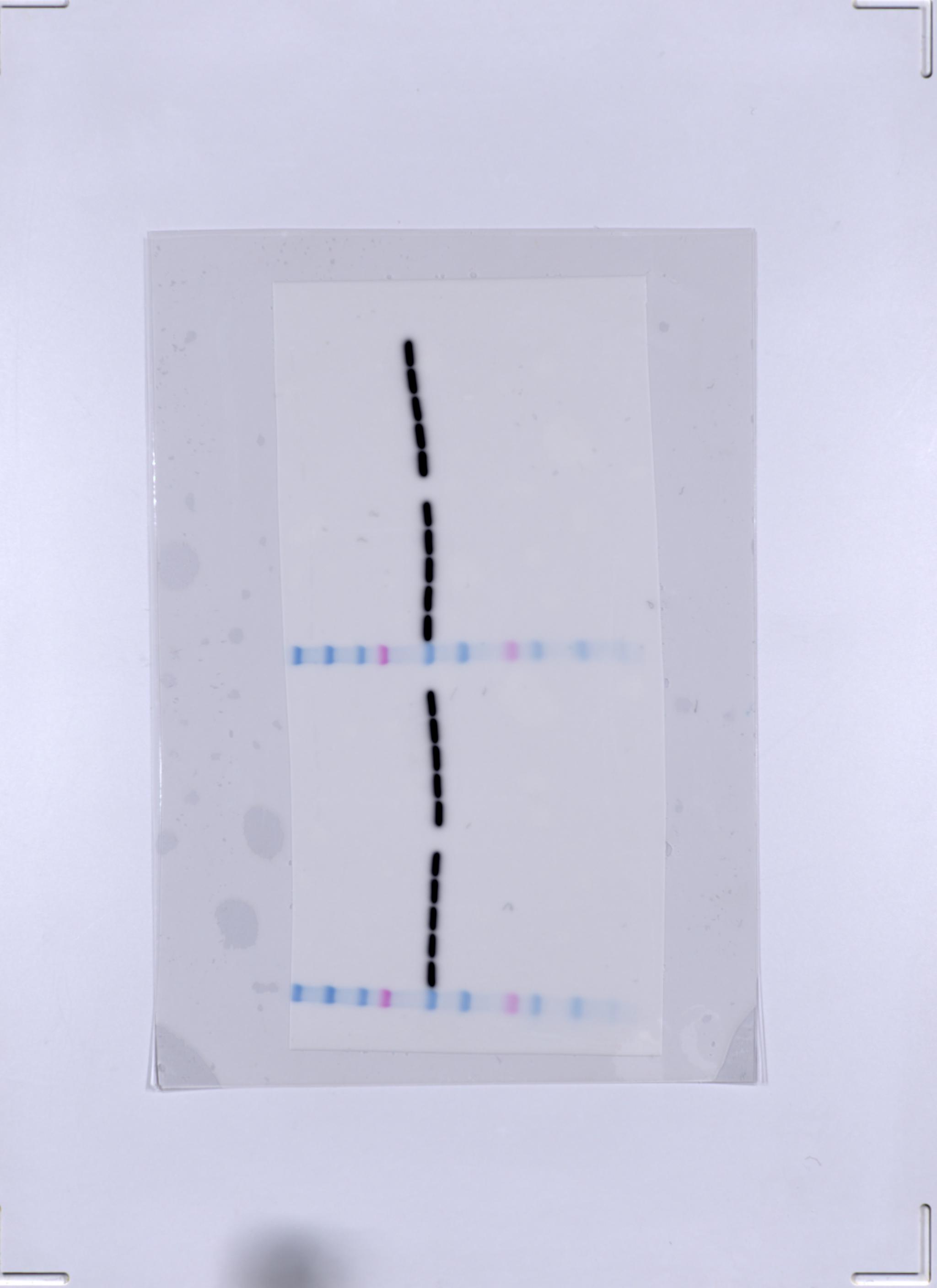

Supplement: Figure 5—figure supplement 1—source data 2. [file elife-81573-fig5-figsupp1-data2.zip › Figure 5-supplement 1-source data 2/Figure 5-supplement 1-source data 2_raw files/CTDNEP1KO CHX240 Tub 2022.08.25_17.18.37_Ch v Tub/CTDNEP1KO CHX240 Tub 2022.08.25_17.18.37_Ch+Marker.jpg]

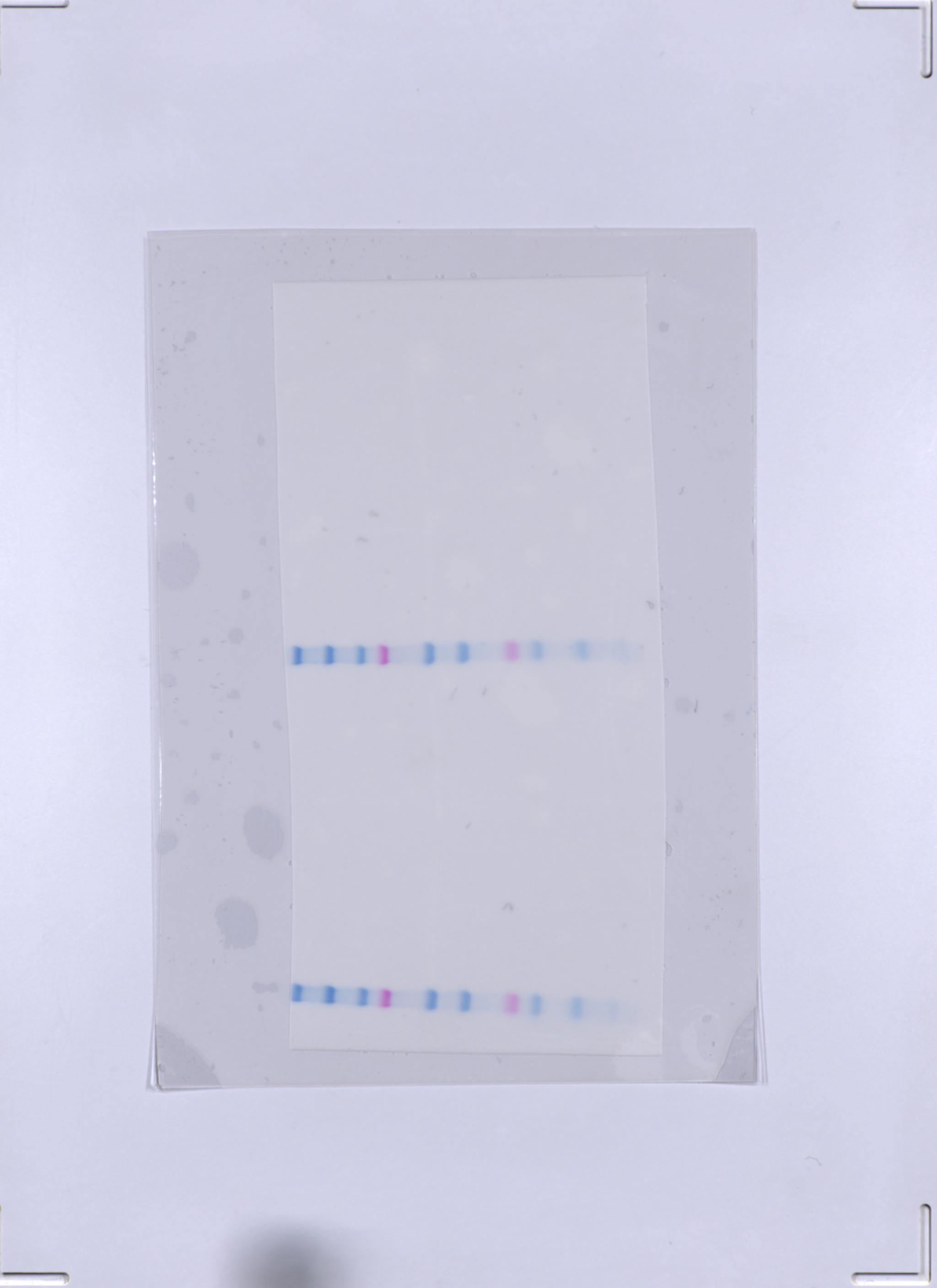

Supplement: Figure 5—figure supplement 1—source data 2. [file elife-81573-fig5-figsupp1-data2.zip › Figure 5-supplement 1-source data 2/Figure 5-supplement 1-source data 2_raw files/CTDNEP1KO CHX240 Tub 2022.08.25_17.18.37_Ch v Tub/CTDNEP1KO CHX240 Tub 2022.08.25_17.18.37_Ch-Marker.jpg]

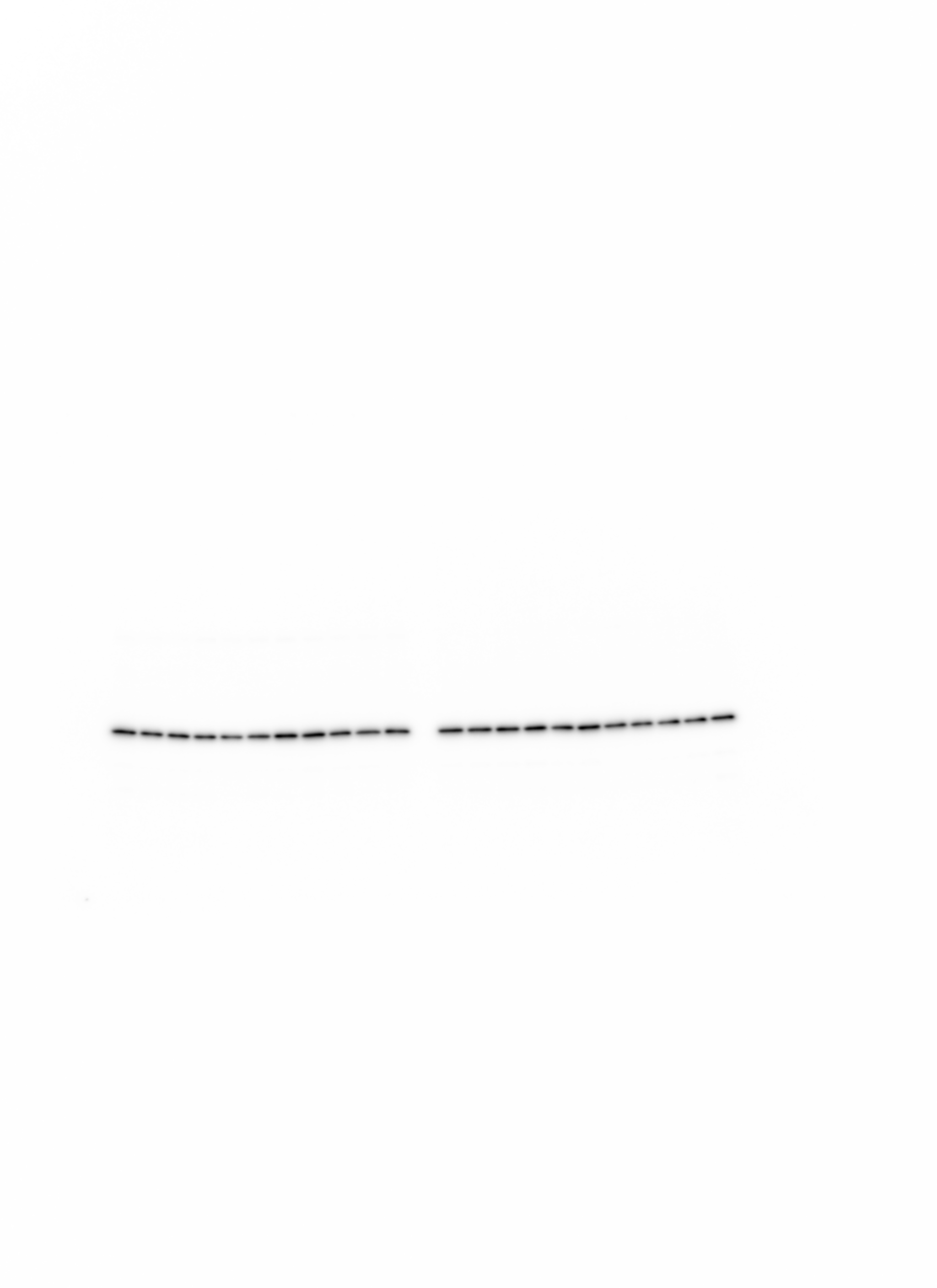

Supplement: Figure 5—figure supplement 1—source data 3. [file elife-81573-fig5-figsupp1-data3.zip › Figure 5-supplement 1-source data 3/Figure 5-supplement 1-source data 3_raw files/ws2 parental gapdh 2022.09.15_16.44.47_Ch/ws2 parental sun2+ga 2022.09.15_16.44.47_Ch.tif]

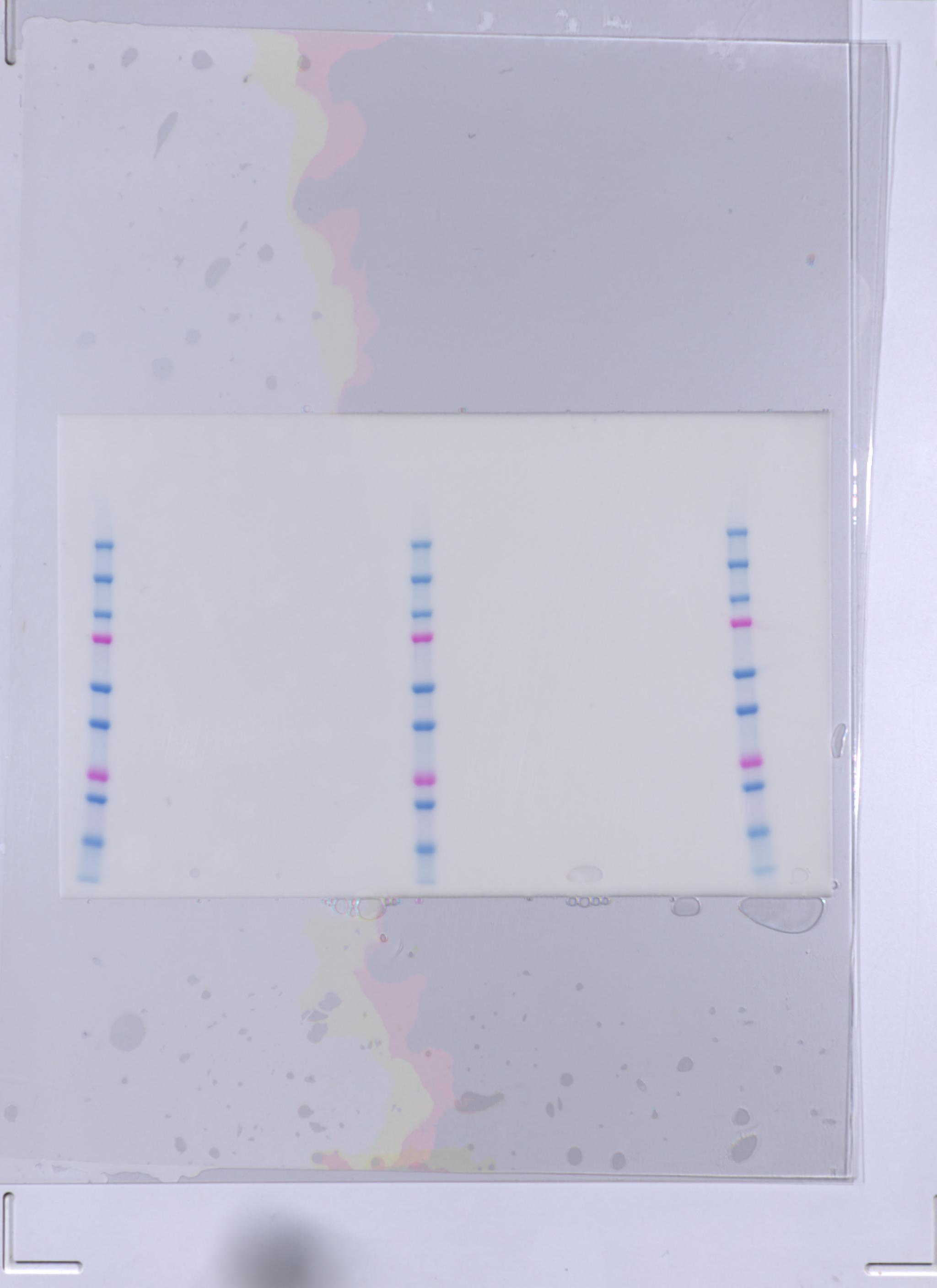

Supplement: Figure 5—figure supplement 1—source data 3. [file elife-81573-fig5-figsupp1-data3.zip › Figure 5-supplement 1-source data 3/Figure 5-supplement 1-source data 3_raw files/ws2 parental gapdh 2022.09.15_16.44.47_Ch/ws2 parental sun2+ga 2022.09.15_16.44.47_Ch-Marker.jpg]

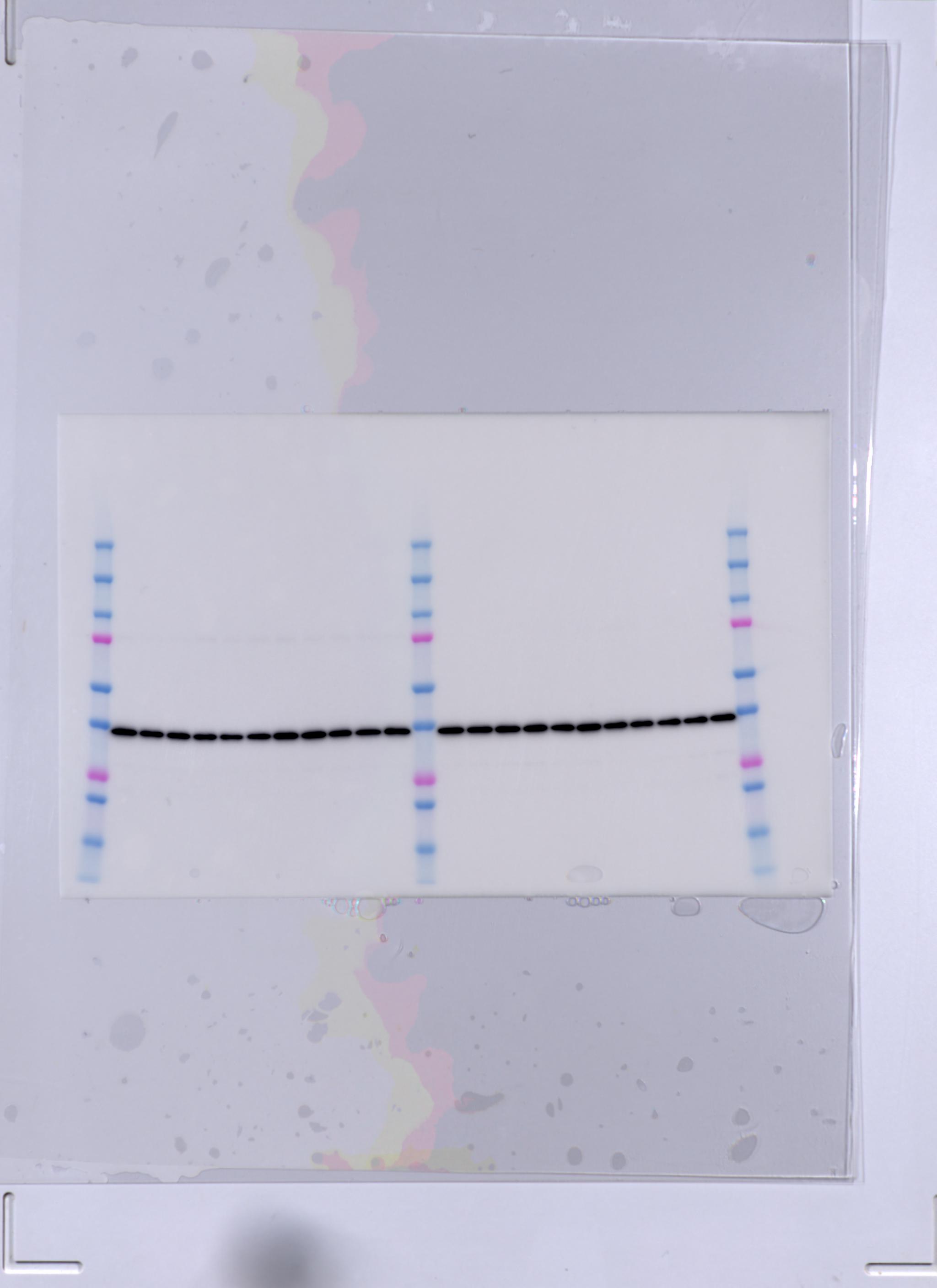

Supplement: Figure 5—figure supplement 1—source data 3. [file elife-81573-fig5-figsupp1-data3.zip › Figure 5-supplement 1-source data 3/Figure 5-supplement 1-source data 3_raw files/ws2 parental gapdh 2022.09.15_16.44.47_Ch/ws2 parental sun2+ga 2022.09.15_16.44.47_Ch+Marker.jpg]

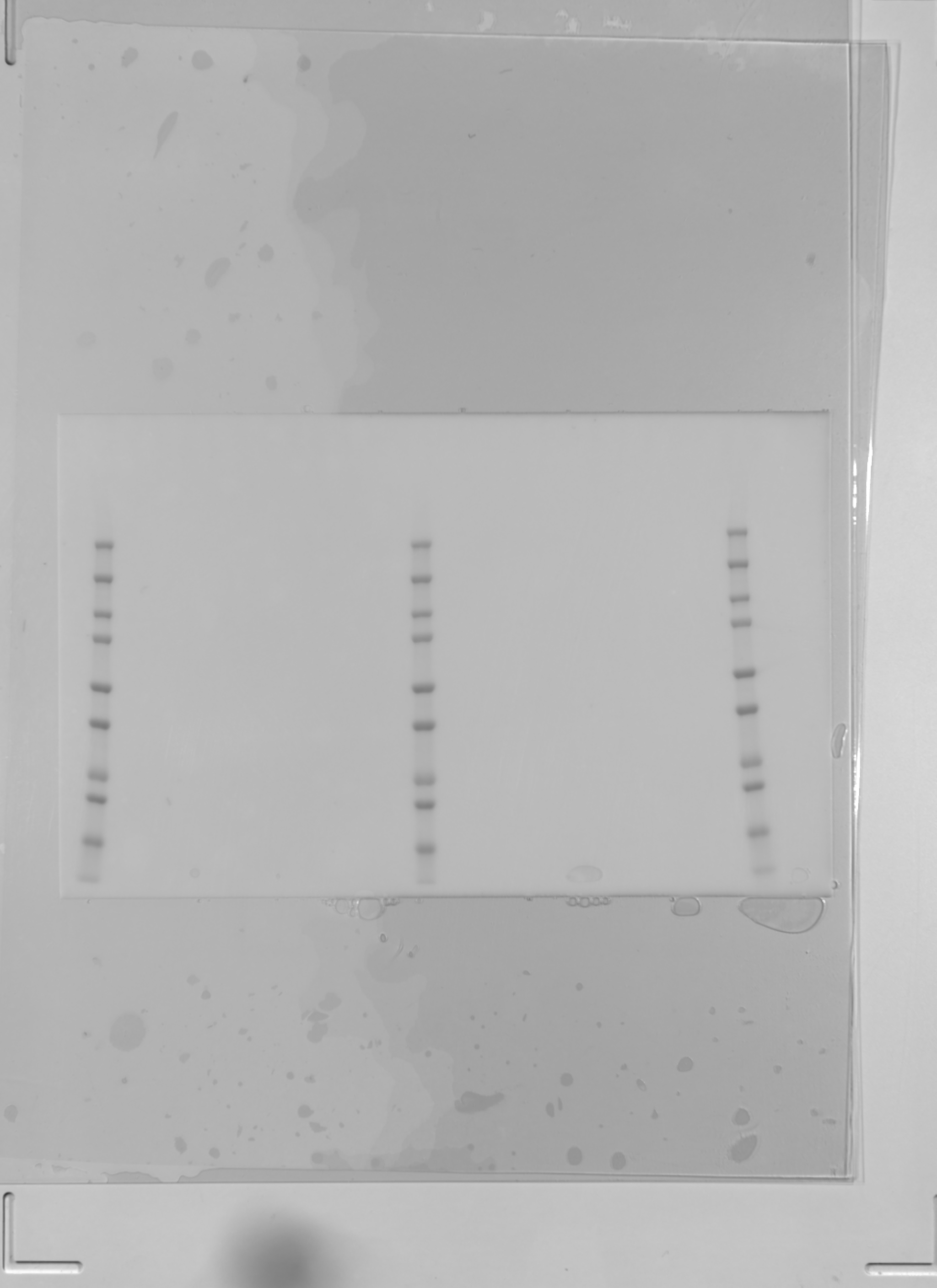

Supplement: Figure 5—figure supplement 1—source data 3. [file elife-81573-fig5-figsupp1-data3.zip › Figure 5-supplement 1-source data 3/Figure 5-supplement 1-source data 3_raw files/ws2 parental gapdh 2022.09.15_16.44.47_Ch/ws2 parental sun2+ga 2022.09.15_16.44.47_Ch-Marker.tif]

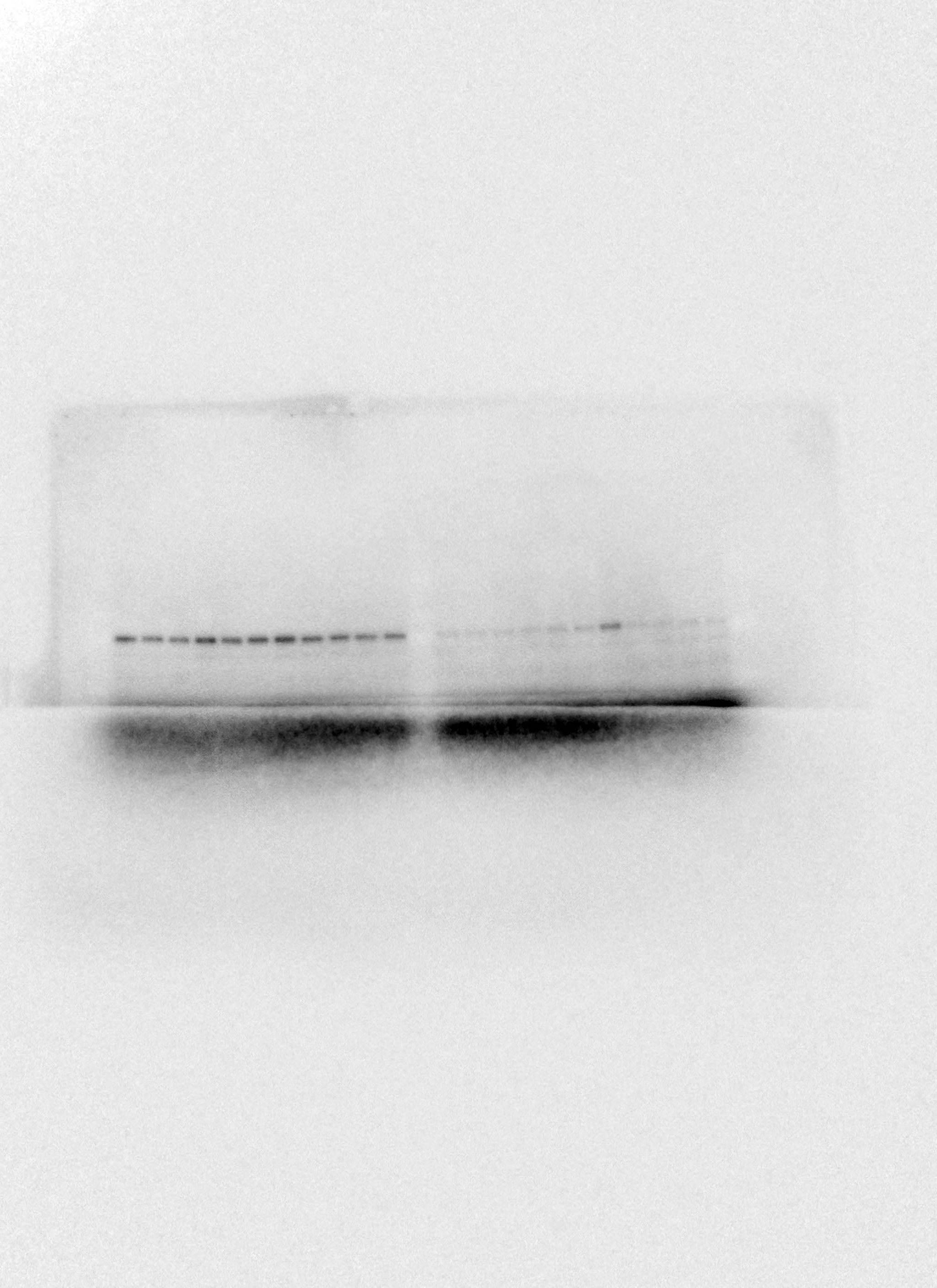

Supplement: Figure 5—figure supplement 1—source data 3. [file elife-81573-fig5-figsupp1-data3.zip › Figure 5-supplement 1-source data 3/Figure 5-supplement 1-source data 3_raw files/ws2 parental sun2 2022.09.15_16.51.01_Ch/ws2 parental sun2 2022.09.15_16.51.01_Ch.tif]

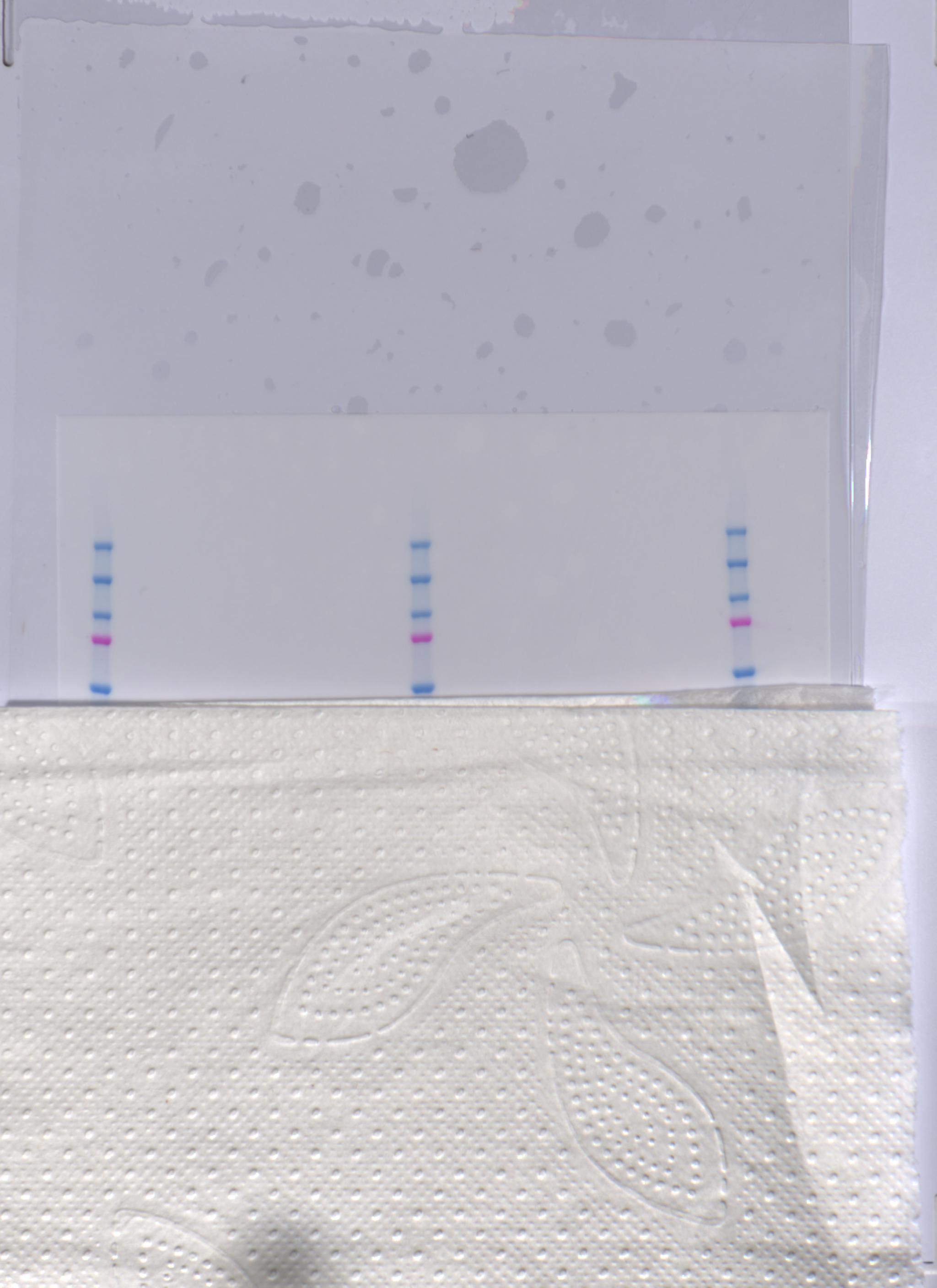

Supplement: Figure 5—figure supplement 1—source data 3. [file elife-81573-fig5-figsupp1-data3.zip › Figure 5-supplement 1-source data 3/Figure 5-supplement 1-source data 3_raw files/ws2 parental sun2 2022.09.15_16.51.01_Ch/ws2 parental sun2 2022.09.15_16.51.01_Ch-Marker.jpg]

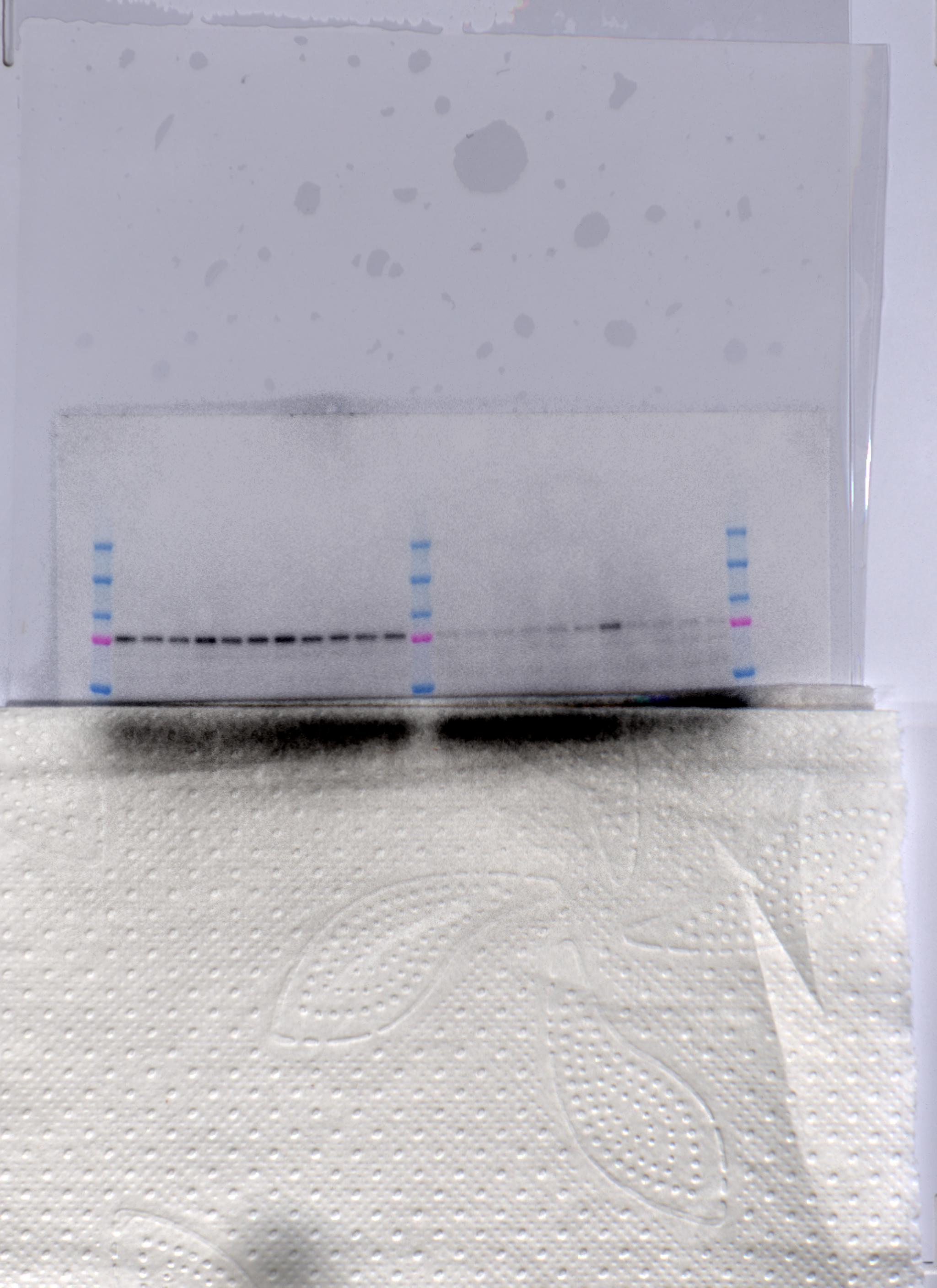

Supplement: Figure 5—figure supplement 1—source data 3. [file elife-81573-fig5-figsupp1-data3.zip › Figure 5-supplement 1-source data 3/Figure 5-supplement 1-source data 3_raw files/ws2 parental sun2 2022.09.15_16.51.01_Ch/ws2 parental sun2 2022.09.15_16.51.01_Ch+Marker.jpg]

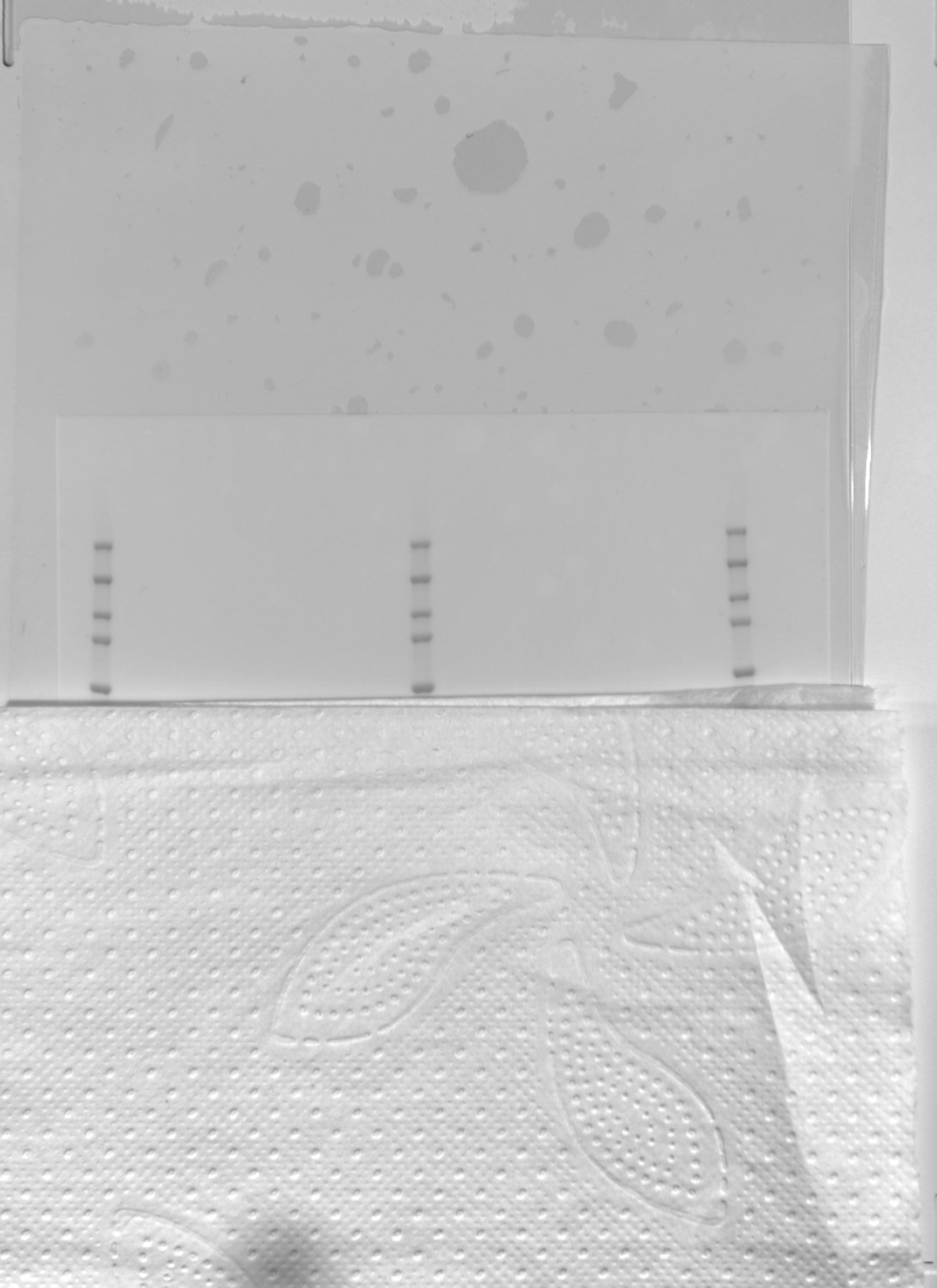

Supplement: Figure 5—figure supplement 1—source data 3. [file elife-81573-fig5-figsupp1-data3.zip › Figure 5-supplement 1-source data 3/Figure 5-supplement 1-source data 3_raw files/ws2 parental sun2 2022.09.15_16.51.01_Ch/ws2 parental sun2 2022.09.15_16.51.01_Ch-Marker.tif]

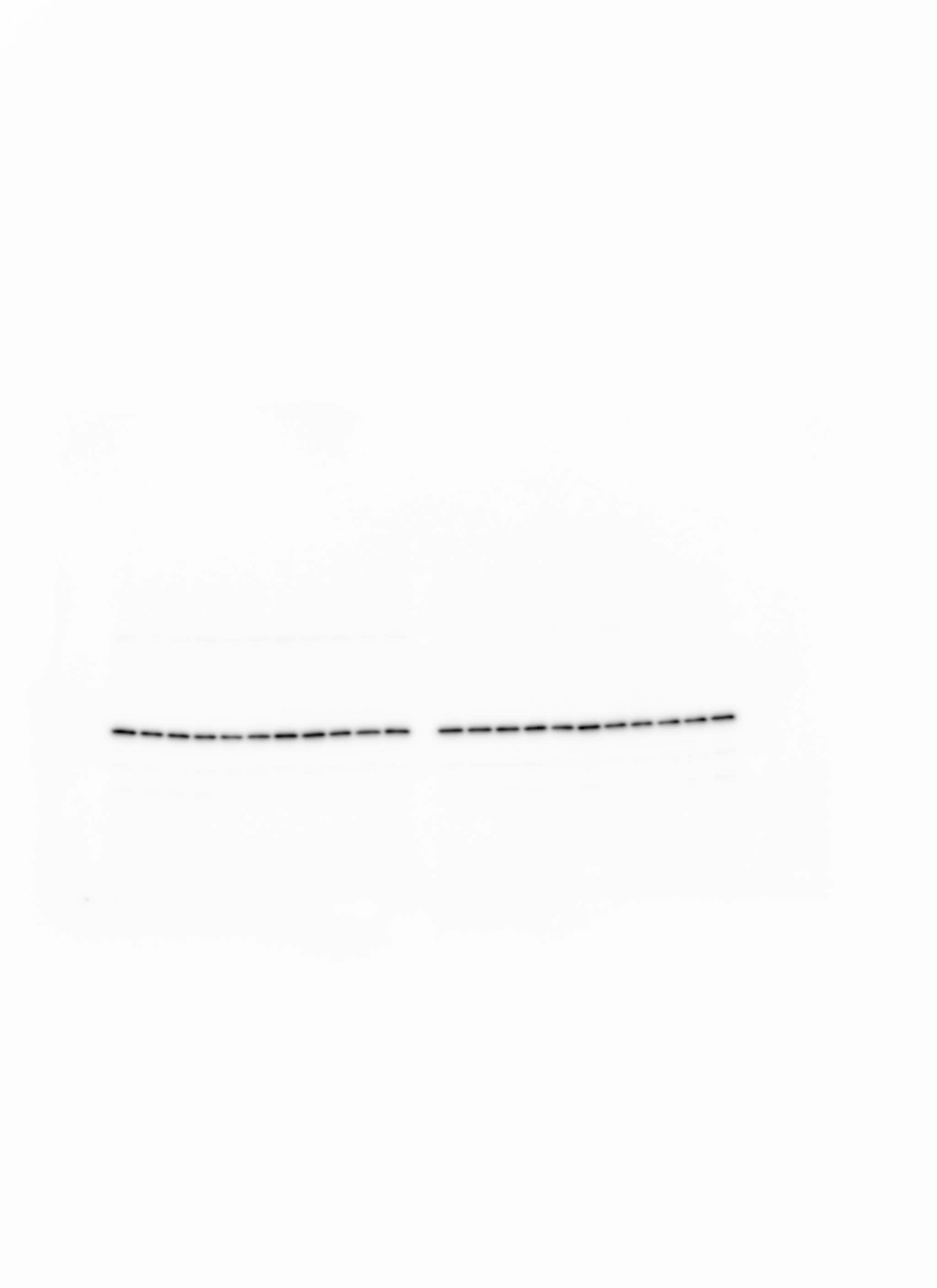

Supplement: Figure 5—figure supplement 1—source data 3. [file elife-81573-fig5-figsupp1-data3.zip › Figure 5-supplement 1-source data 3/Figure 5-supplement 1-source data 3_raw files/ws2 parental gapdh 2022.09.15_16.46.45-09_Ch/ws2 parental sun2+ga 2022.09.15_16.46.45-09_Ch.jpg]

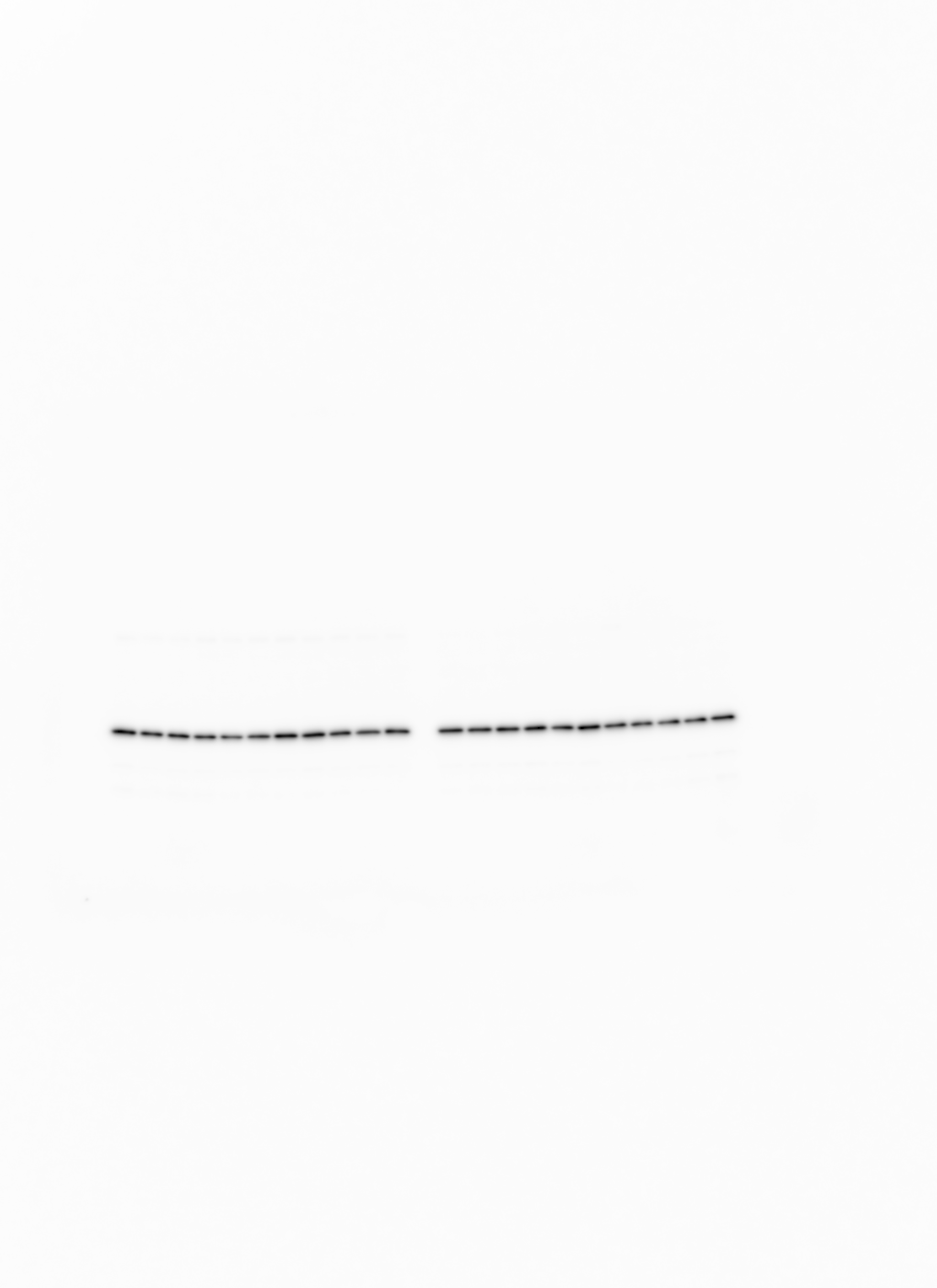

Supplement: Figure 5—figure supplement 1—source data 3. [file elife-81573-fig5-figsupp1-data3.zip › Figure 5-supplement 1-source data 3/Figure 5-supplement 1-source data 3_raw files/ws2 parental gapdh 2022.09.15_16.46.45-09_Ch/ws2 parental sun2+ga 2022.09.15_16.46.45-09_Ch.tif]

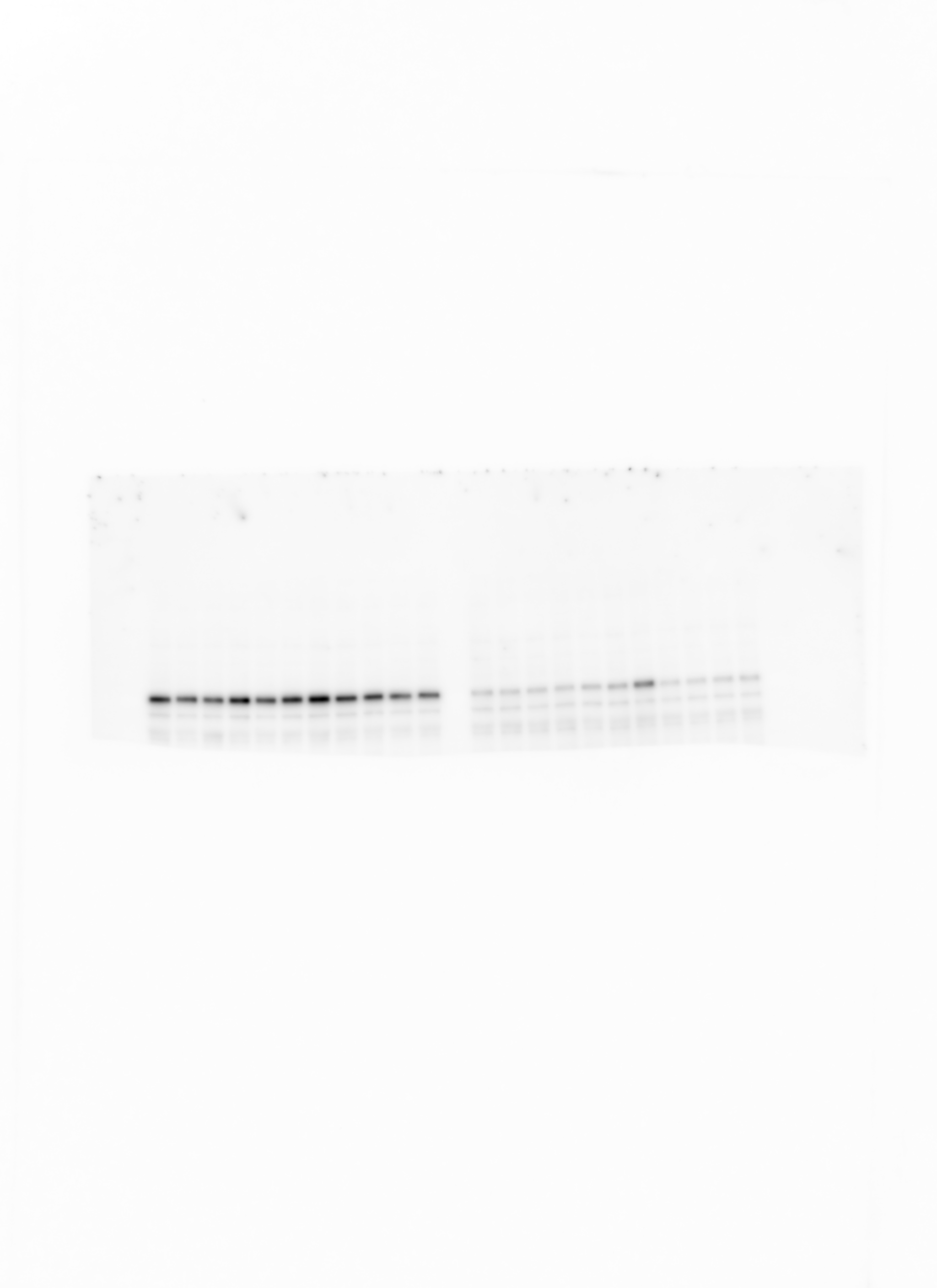

Supplement: Figure 5—figure supplement 1—source data 3. [file elife-81573-fig5-figsupp1-data3.zip › Figure 5-supplement 1-source data 3/Figure 5-supplement 1-source data 3_raw files/ws2 parental sun2 2022.09.15_17.20.58-12_Ch/ws2 parental sun2 2022.09.15_17.20.58-12_Ch.tif]

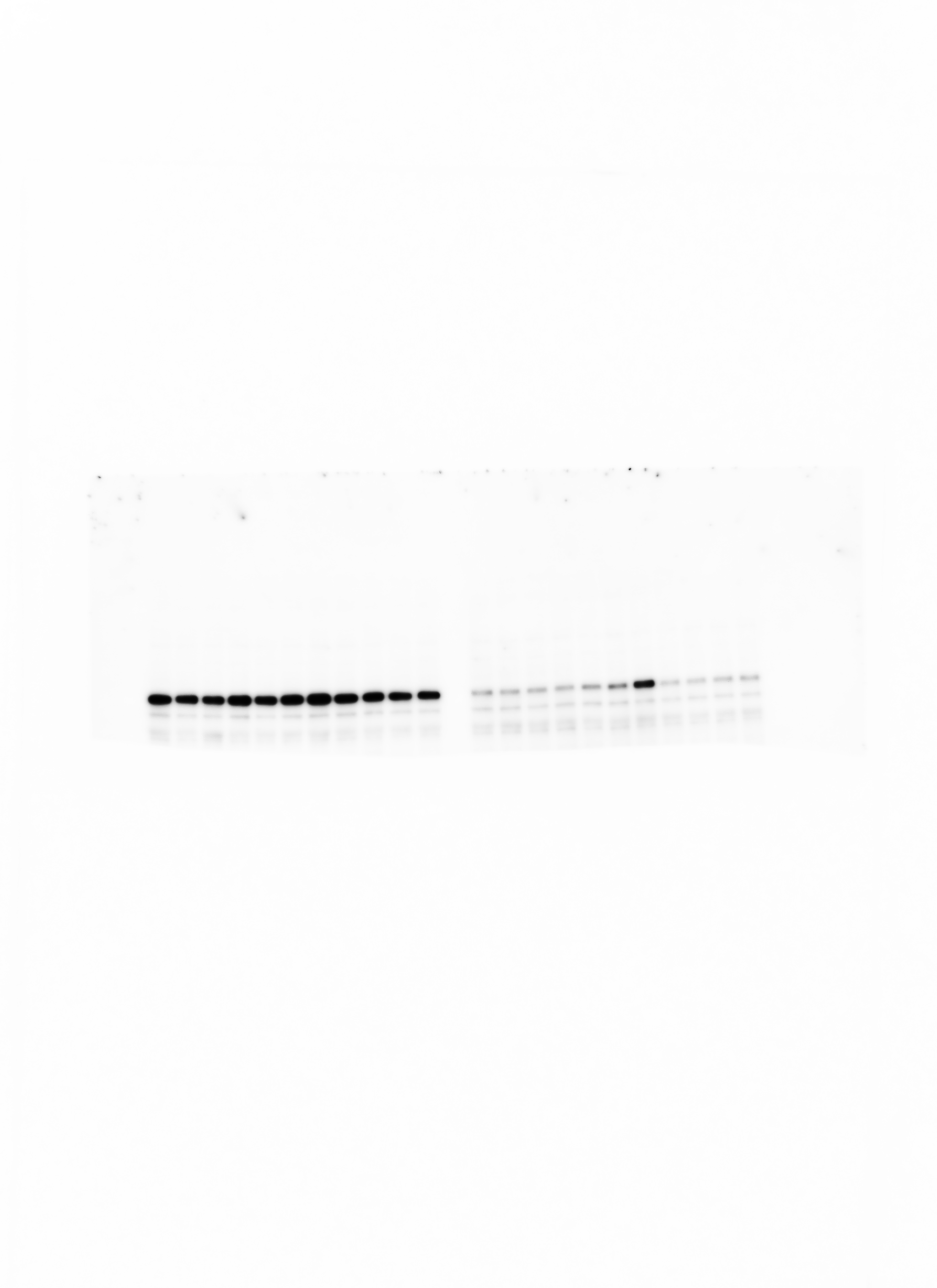

Supplement: Figure 5—figure supplement 1—source data 3. [file elife-81573-fig5-figsupp1-data3.zip › Figure 5-supplement 1-source data 3/Figure 5-supplement 1-source data 3_raw files/ws2 parental sun2 2022.09.15_17.20.58-12_Ch/ws2 parental sun2 2022.09.15_17.20.58-12_Ch_editted.tif]

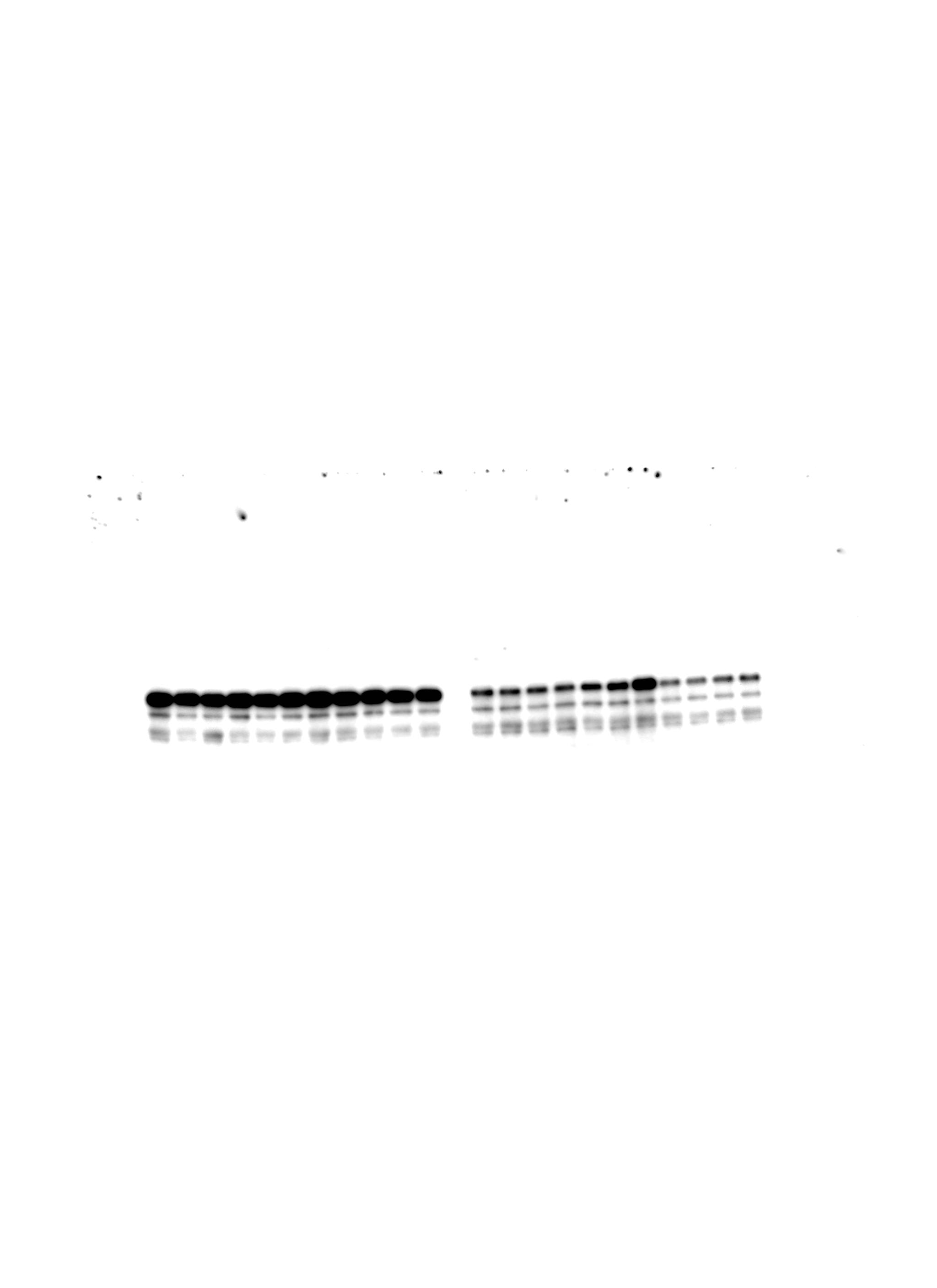

Supplement: Figure 5—figure supplement 1—source data 3. [file elife-81573-fig5-figsupp1-data3.zip › Figure 5-supplement 1-source data 3/Figure 5-supplement 1-source data 3_raw files/ws2 parental sun2 2022.09.15_17.20.58-12_Ch/ws2 parental sun2 2022.09.15_17.20.58-12_Ch.jpg]

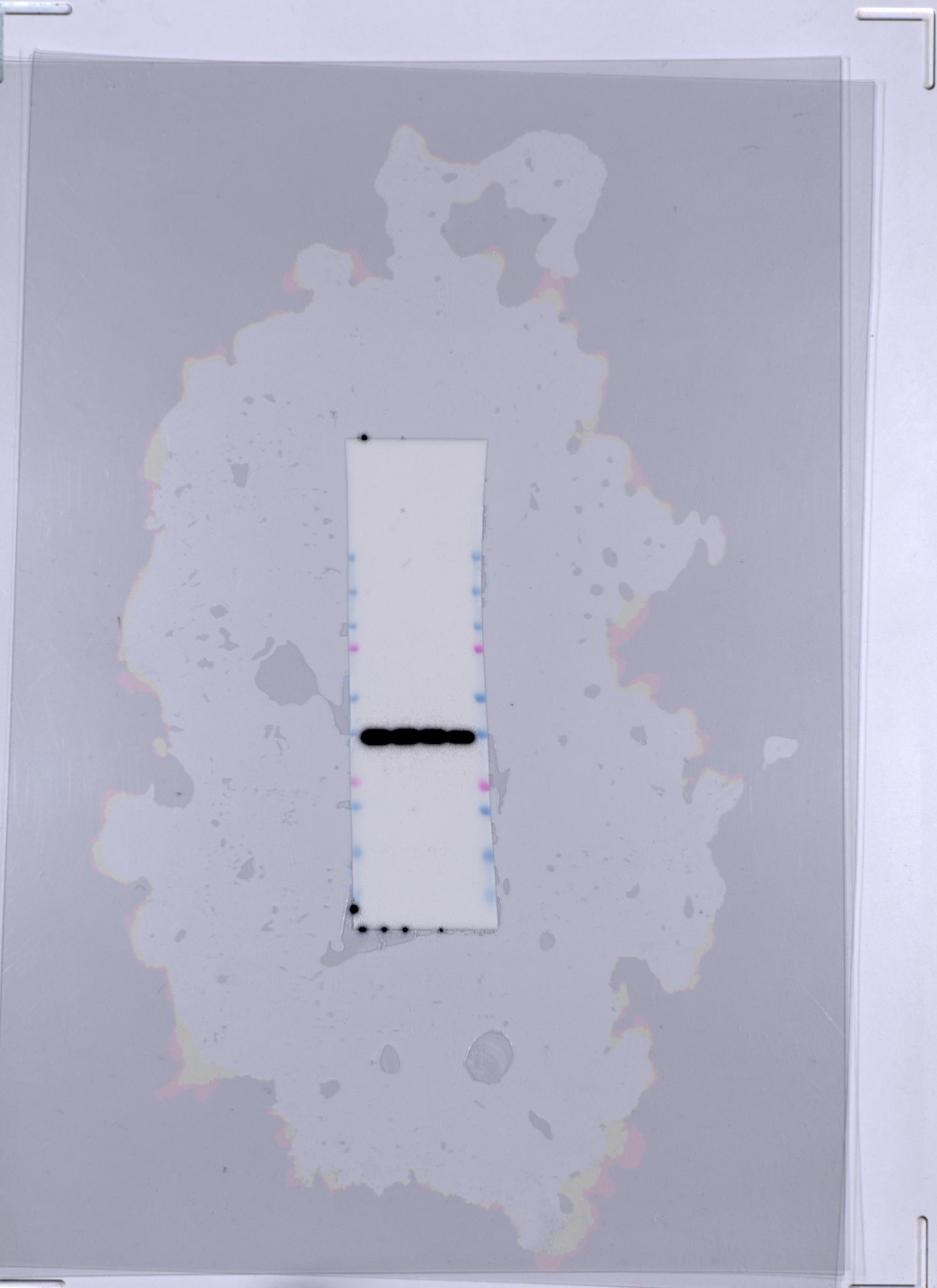

Supplement: Figure 5—figure supplement 1—source data 4. [file elife-81573-fig5-figsupp1-data4.zip › Figure 5-supplement 1-source data 4/Figure 5-supplement 1-source data 4_raw files/LK220618 ck2i GAPDH 2022.06.18_16.34.11_Ch+Marker.jpg]

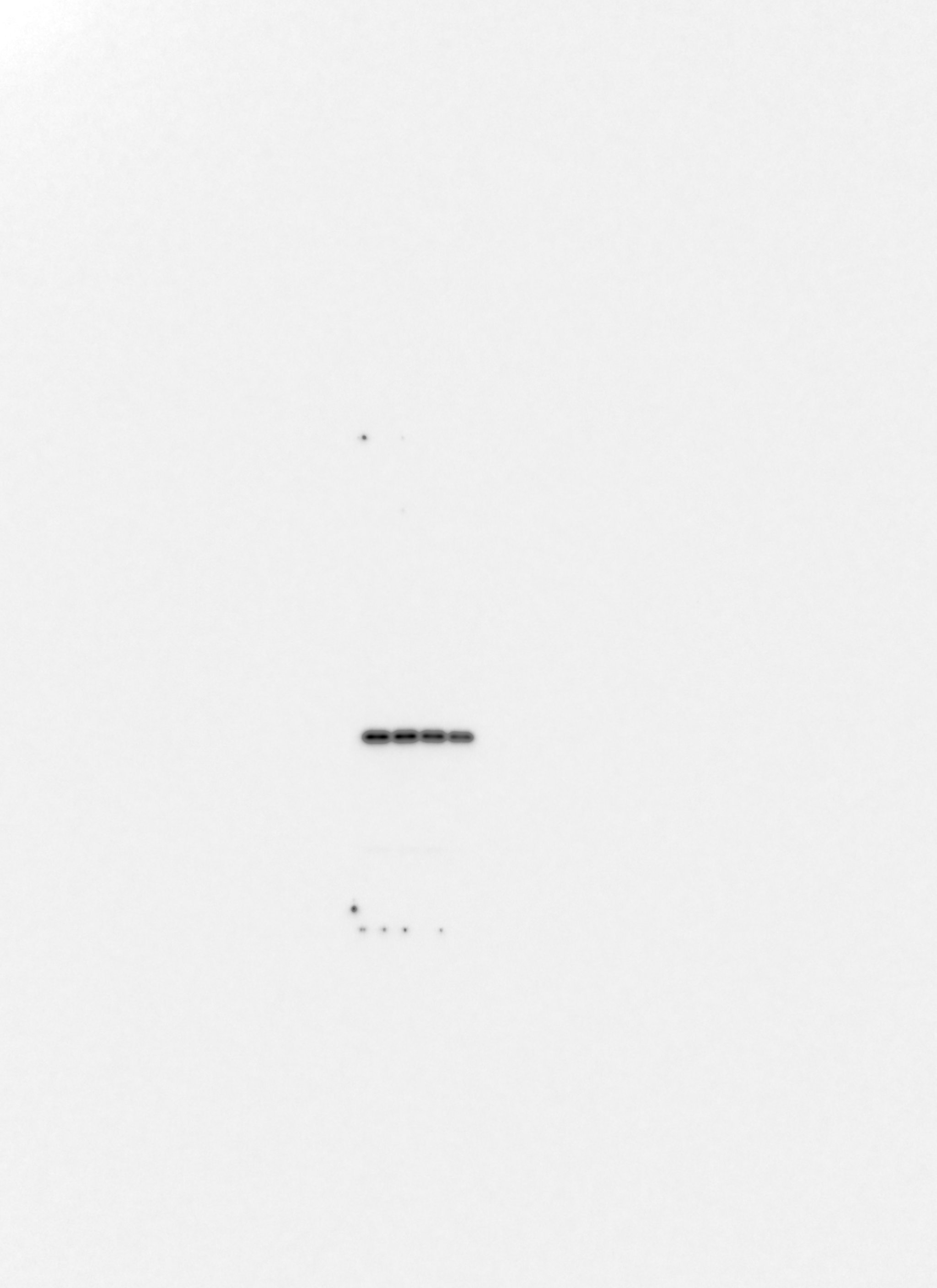

Supplement: Figure 5—figure supplement 1—source data 4. [file elife-81573-fig5-figsupp1-data4.zip › Figure 5-supplement 1-source data 4/Figure 5-supplement 1-source data 4_raw files/LK220618 ck2i GAPDH 2022.06.18_16.36.01-08_Ch.tif]

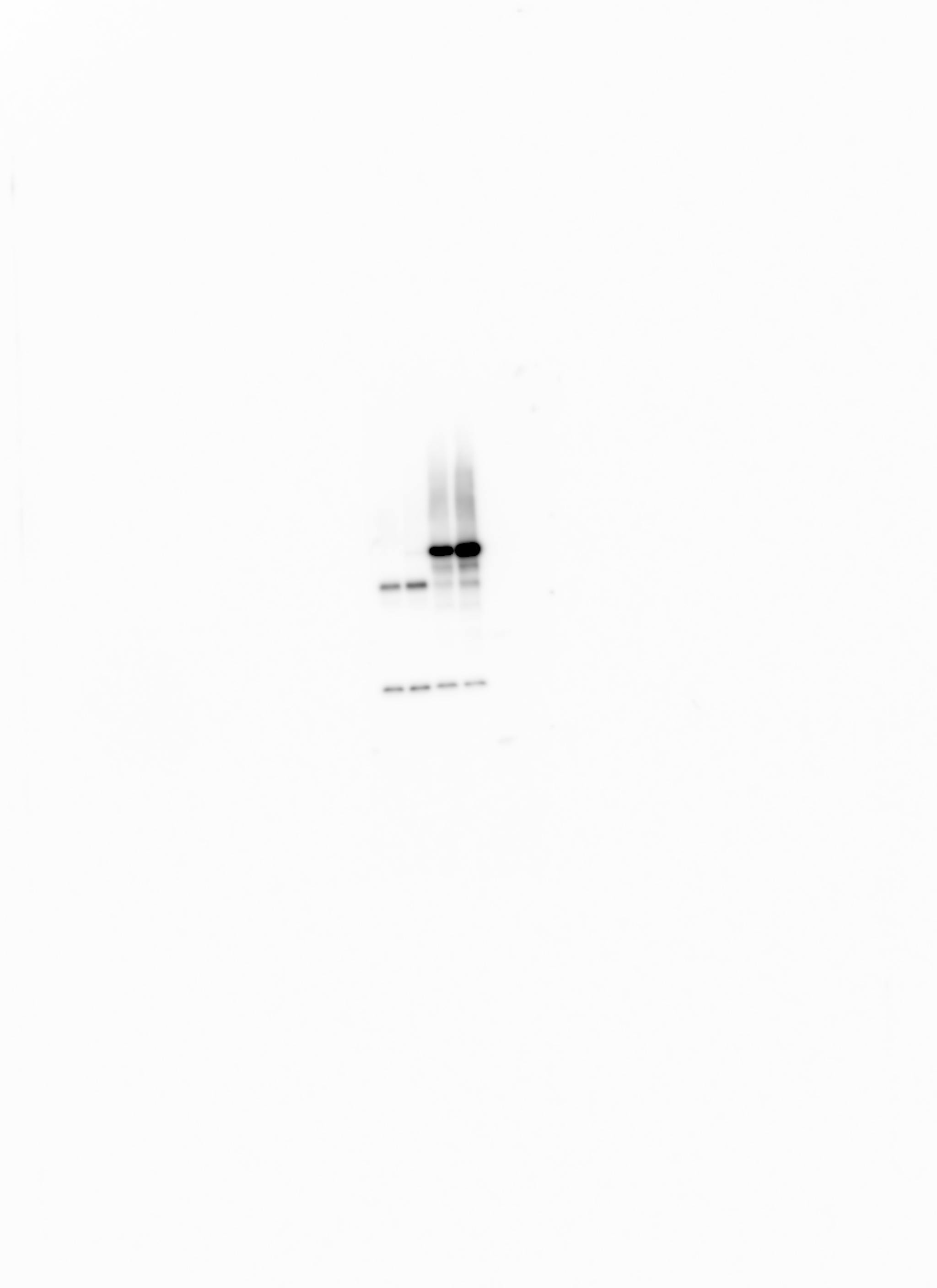

Supplement: Figure 5—figure supplement 1—source data 4. [file elife-81573-fig5-figsupp1-data4.zip › Figure 5-supplement 1-source data 4/Figure 5-supplement 1-source data 4_raw files/LK220617 ck2i Sun2 2022.06.17_15.58.09-12_Ch.tif]

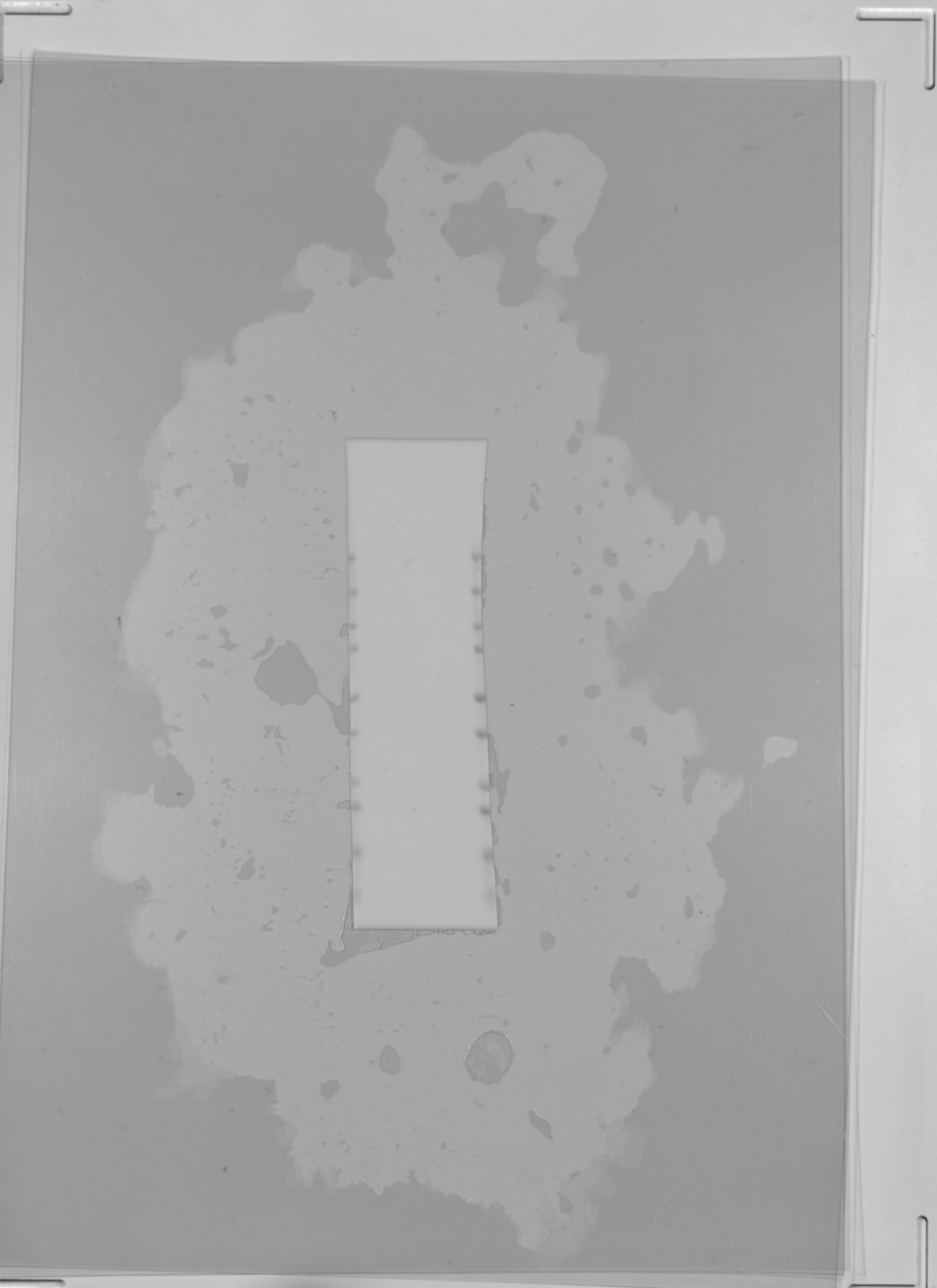

Supplement: Figure 5—figure supplement 1—source data 4. [file elife-81573-fig5-figsupp1-data4.zip › Figure 5-supplement 1-source data 4/Figure 5-supplement 1-source data 4_raw files/LK220618 ck2i GAPDH 2022.06.18_16.34.11_Ch-Marker.tif]

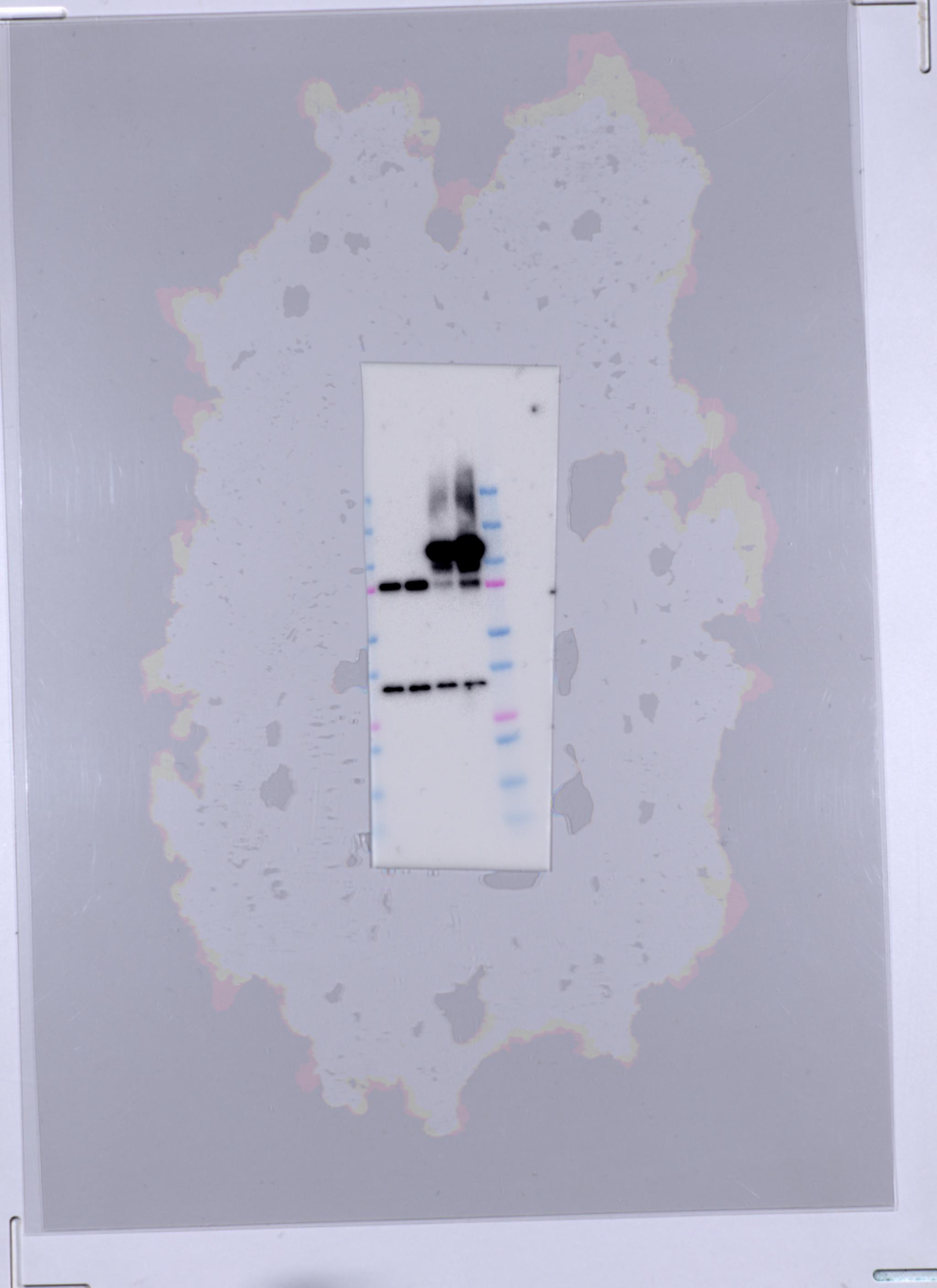

Supplement: Figure 5—figure supplement 1—source data 4. [file elife-81573-fig5-figsupp1-data4.zip › Figure 5-supplement 1-source data 4/Figure 5-supplement 1-source data 4_raw files/LK220617 ck2i Sun2 2022.06.17_15.38.42_Ch+Marker.jpg]

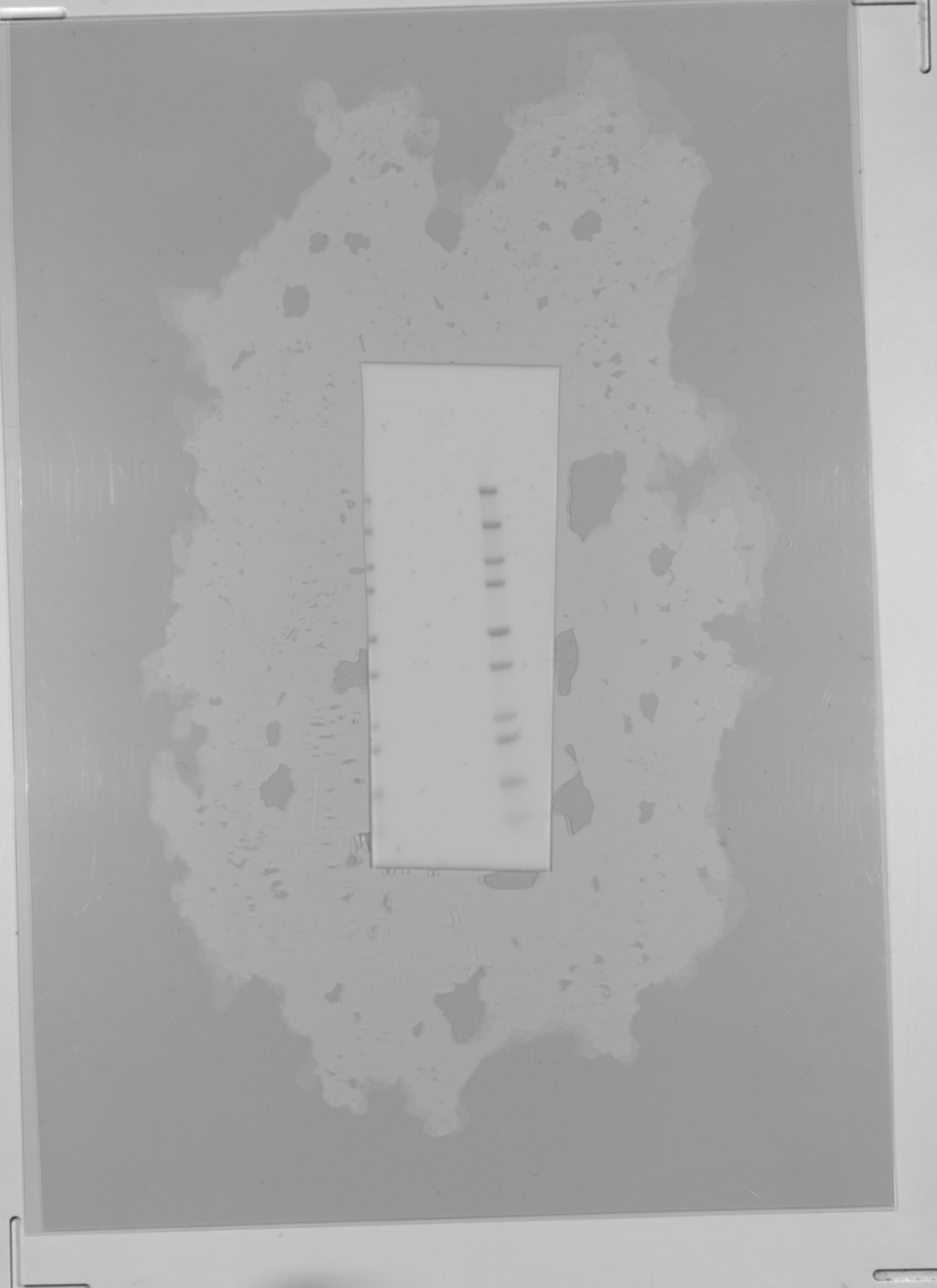

Supplement: Figure 5—figure supplement 1—source data 4. [file elife-81573-fig5-figsupp1-data4.zip › Figure 5-supplement 1-source data 4/Figure 5-supplement 1-source data 4_raw files/LK220617 ck2i Sun2 2022.06.17_15.38.42_Ch-Marker.tif]
